# Extending the Scope of the New Variant of the Castagnoli–Cushman Cyclocondensation onto *o*-Methyl Benzoic Acids Bearing Various Electron-Withdrawing Groups in the α-Position

**DOI:** 10.3390/molecules27217211

**Published:** 2022-10-25

**Authors:** Natalia Guranova, Lyudmila Yakovleva, Olga Bakulina, Dmitry Dar’in, Mikhail Krasavin

**Affiliations:** 1 Institute of Chemistry, Saint Petersburg State University, 26 Universitetskii Prospect, Peterhof 198504, Russia; 2 Institute for Medicine and Life Sciences, Immanuel Kant Baltic Federal University, Kaliningrad 236041, Russia

**Keywords:** cyclocondensation, isoquionolone, imine, homophthalic, Castagnoli-Cushman reaction

## Abstract

Based on the previously reported involvement of homophthalic acid monoesters in the Castagnoli–Cushman reaction-type cyclocondensation with imines, we tested a number of other *o*-methyl benzoic acids bearing various electron-withdrawing groups in the α-position. The majority of these substrates delivered the expected tetrahydroisoquinolone adducts on activation with CDI or acetic anhydride. Homophthalic acid mononitriles displayed the highest promise as substrates for the new reaction, both in terms of scope and product yields. Homophthalic acid monoamides either gave low yields or failed to react with imines. Sulfonyl-substituted substrates gave the desired (and hitherto unknown) type of tetrahydroisoquinolines. Despite the low yields, this approach to sulfonyl-substituted tetrahydroisoquinolines appears practical as alternative syntheses based on the traditional, carboxylic acid CCR adducts would presumably be cumbersome and multistep. The azido- and nitro-substituted *o*-methyl benzoic acids failed to react with imines.

## 1. Introduction

**Figure 1:**
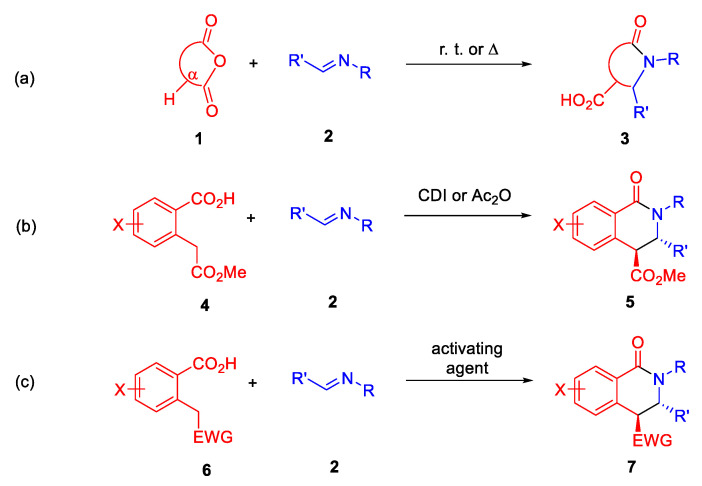
(**a**) The Castagnoli–Cushman reaction; (**b**) the recently developed CCR of homophthalic acid monoesters; (**c**) the reaction scope extended to electron-withdrawing groups other than methoxycarbonyl (this work).

**Figure 2:**
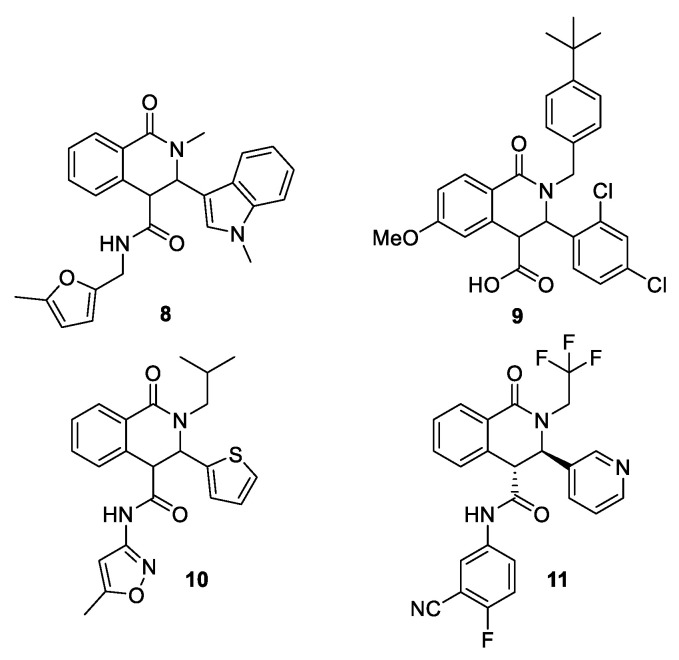
Examples of diversely biologically active tetrahydroisoquinolones.

The Castagnoli–Cushman reaction (CCR) [1,2,3,4,5,6] is a distinctly versatile [4+2]-type cyclocondensation of α-C-H-acidic cyclic anhydrides **1** with imines **2** leading, depending on the specific anhydride employed [7], to skeletally diverse [8] polysubstituted lactams **3**, often in diastereoselective fashion. Although in some instances the cyclic anhydride can be generated from the respective dicarboxylic acid in situ [9], hydrolytically prone cyclic anhydrides remain the traditional reagent input for the CCR, which hinders the use of this reaction for large combinatorial arrays. Moreover, in some instances the carboxylic acid resulting from the initial CCR has a tendency to decarboxylate [10] and/or requires in situ esterification before it can be conveniently isolated by chromatography. With this obstacle in mind, we developed a novel variant of the CCR, which relies upon monoesters of homophthalic acid **4** (whose anhydride is one of the most popular reagents for the CCR [7]) being activated towards the condensation with imines **2** by either CDI (1,1’-carbonyldiimidazole) or acetic anhydride [11]. Not only did this variant of the CCR deliver easily isolable esters **5** directly and alleviate the need to use hydrolytically prone homophthalic anhydrides, it also opened a potential prospect of extending this reactivity to other *o*-methyl benzoic acids bearing various electron-withdrawing groups in the α-position (**6**). Besides the fundamental interest towards realizing such a novel reactivity of readily available substrates in practice, the opportunity to obtain tetrahydroisoquinolones **7** bearing other groups, in lieu of the carboxylic acid, was viewed as a significant advantage from the medicinal chemistry perspective. Indeed, varying the electron-withdrawing group in **7**, depending on the substrate **6,** could afford a direct access to various carboxylic acid congeners or replacements with no need for multistep reaction sequences (Figure 1). Considering the medicinal chemistry significance of the tetrahydroisoquinolone (THIQ) scaffold as the core of adrenocorticotropic hormone receptor modulator **8** [12], apoptosis regulator **9** [13], trypanocidal cysteine protease inhibitor **10** [14] as well as antimalarial **11** [15] (to mention only a few examples, Figure 2), validating substrates **6** for the CCR clearly represents a worthy undertaking. Herein, we present the results obtained in the course of investigating the new reagent space for the recently discovered variant of the CCR.

## 2. Results and Discussion

Initially, we began to explore the reaction of homophthalic mononitriles **12a**–**c**. Electron-donating and electron-withdrawing group substitution was expected to have a bearing on the CH_2_-acidity of these substrates and, therefore, on the facility of the CCR-type cyclocondensation as understood within the suggested mechanism of the reaction (*vide infra*).

**Scheme 1:**
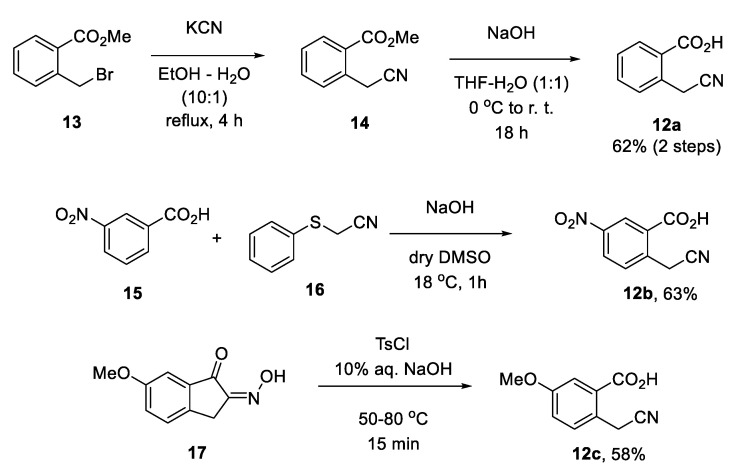
Preparation of homophthalic mononitriles **12a**–**c**.

Unsubstituted homophthalic mononitrile **12a** was prepared from methyl *o*-(bromomethyl)benzoate (**13**) as described in the literature [16]. The initial nucleophilic substitution product **14**, without purification, was subjected to basic hydrolysis to give **12a** in 62% yield over two steps. The nitro-substituted substrate **12b** was prepared by the vicarious nucleophilic substitution of hydrogen in *m*-nitrobenzoic acid (**15**) by deprotonated *S*-(cyanomethyl) benzene thiol **16** [17]. Finally, the methoxy-substituted substrate **12c** was prepared from indane-1,2-dione monoxime **17** via tandem dehydration/hydrolysis [18] (Scheme 1).

**Scheme 2:**
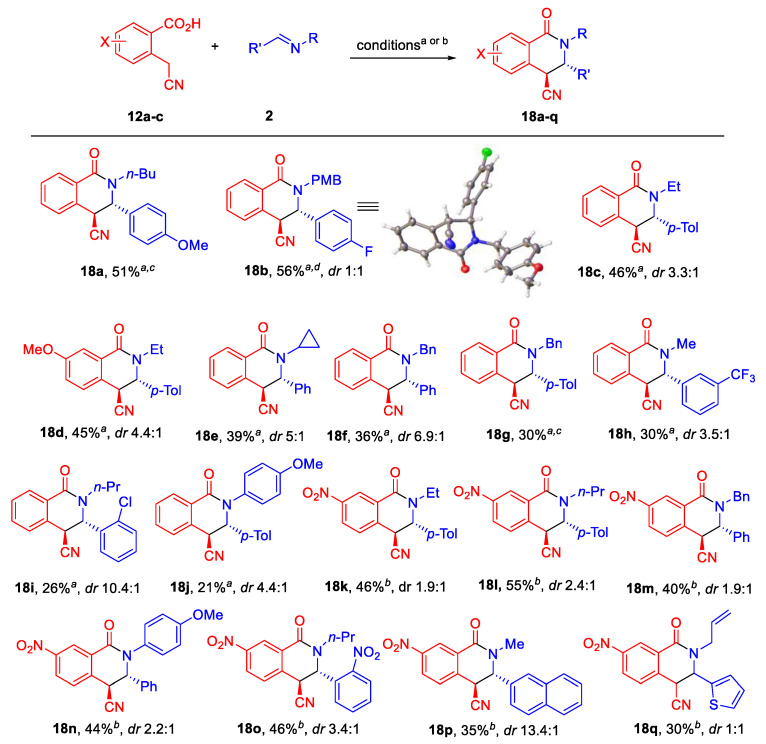
Synthesis of 4-cyanotetrahydroisoquinolones **18a**–**q** (*^a^*CDI, PhCl, 130 °C, 16 h; *^b^*Ac_2_O, PhCl, 60 °C, 2 h; *^c^*single diastereomer; *^d^*individual isomers were isolated).

With the substrates **12a**–**c** at hand, we proceeded to explore their modified CCR with various imines **2** using either CDI (1,1’-carbonyldiimidazole) or acetic anhydride (Ac_2_O) [11] as the carboxylic group activator (Scheme 2). To our delight, the reaction delivered the expected 4-cyanotetrahydroisoquinolones **18a**–**q** in moderate yields and displayed either no diastereoselectivity (*cf.* **18b** or **18q**) or gave exclusively one diastereomer (*cf.* **18a** or **18g**). The relative trans-stereochemistry was assigned to the major diastereomer obtained in the majority of cases by correlation of their spectral properties (specifically, the *^3^J* coupling constants between the methine protons) with those of the trans-**18b** whose stereochemistry was unequivocally confirmed by the single-crystal X-ray analysis (CCDC2192610). This isomer of **18b** (with X-ray data) revealed the signal of H-3 methine atom as doublet with *J* = 2.6 Hz (for details see ESI, Table S3), while the other (cis) isomer displayed the coupling constant of *J* = 5.9 Hz. For all other products **18** the corresponding coupling constants were found to be in the range 1.7–2.7 Hz for major isomers and 5.6–6.3 Hz for the minor. The reactivity of unsubstituted (**12a**) or methoxy-substituted (**12c**) substrates was markedly lower compared to their nitro-substituted (**12b**) counterpart. While in the former case, prolonged heating at 130 ℃ with CDI as the activating agent was needed, in the latter case, only two hours at 60 ℃ were sufficient to complete the reaction with Ac_2_O as the carboxylic acid activator.

**Scheme 3:**
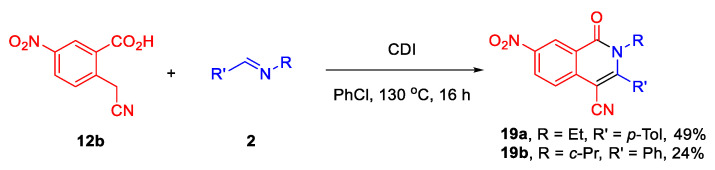
Formation of 4-cyanodihydroisoquinolones **19a**,**b** from substrate **12b** activated by CDI.

Acetic anhydride was used to activate substrate **12b** as with CDI, 4-cyanodihydroisoquinolones **19a**,**b** (Scheme 3) were exclusively obtained in moderate yield, presumably, via the oxidation of the respective α-carbanion (formed, in turn, from the initial tetrahydroisoquinolone adduct due to the basic character of imidazole generated from CDI). Interestingly, thorough exclusion of air from the reaction medium by argon purging did not suppress this process, which is likely due to the possibility of nitro compounds self-oxidizing [19]. Likewise, variation in such reaction parameters as temperature, reaction duration and concentration in compound **19a** synthesis did not dramatically alter the outcome of the reaction with CDI. However, switching to acetic anhydride as the carboxylic acid activator allowed conditions to be quickly identified (60 ℃, 2 h) for the formation of this compound’s unoxidized version, i.e., compound **18k**, in a respectable 46% yield (see Table S1 in Supplementary Materials for the reaction optimization towards **19a** and **18k**).

**Scheme 4:**
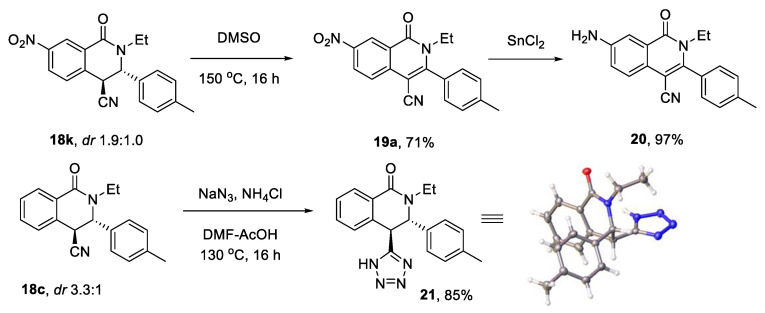
Postcondensational manipulation of 4-cyanotetrahydrisoquinolones **18k** and **18c**.

While compounds **18** present a novel substitution pattern around the tetrahydroisoquinolone scaffold accessible by the CCR-type cyclocondensation, these compounds can be manipulated further via a range of post-condensational modifications. For instance, we have shown that compound **18k** could be intentionally oxidized to its dihydroisoquinolone counterpart **19a** by heating at 150 °C in DMSO (dimethylsulfoxide). In the resulting compound **19a**, the nitro group can be reduced by stannous chloride to give dihydroisoquinolone compound **20** with an amino group, i.e., an additional site for further modification. The nitrile functionality is a versatile precursor to various heterocyclic moieties including 1,3-oxazole [20] and tetrazole [21], the latter being a well-established bioisostere of the carboxylic acid functionality for drug design [22]. Thus, compound **18c** (taken as a 3.3:1.0 diastereomeric mixture) underwent a formal [3+2] cycloaddition with sodium azide in the presence of ammonium chloride to give tetrazole **21** isolated chromatographically as a single trans-isomer. The configuration of the latter was confirmed by the single-crystal X-ray analysis (CCDC2203753) (Scheme 4).

**Scheme 5:**
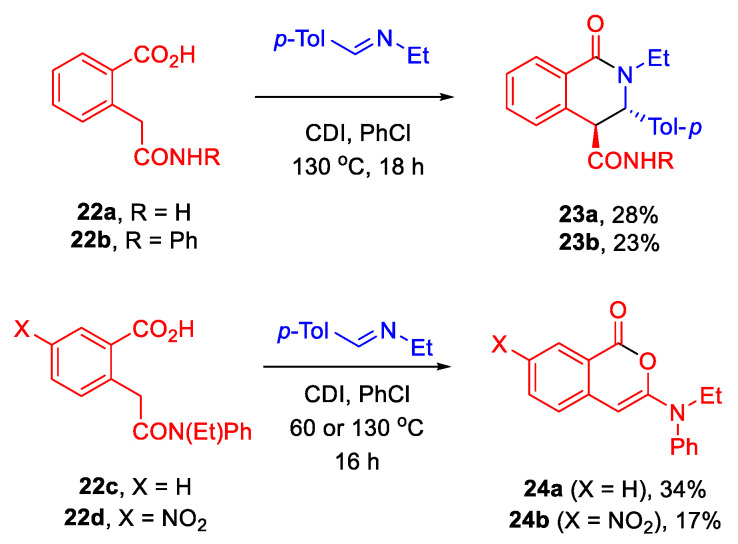
Homophthalic acid monoamides **22a**–**d** tested in the new type of CCR.

While the traditional carboxylic acid Castagnoli–Cushman adducts obtainable from homophthalic anhydride can be amidated directly [23], we were curious to see if the amide functionality can be installed in lieu of the nitrile function in compounds **18** and serve as a sufficiently electron-withdrawing group to enable the new variant of the CCR-type cyclocondensation. Homophthalic monoamides **22a**–**d** (obtainable from the respective homophthalic anhydrides and ammonia or anilines [24]) were tested in the reaction with one exemplary imine (**2a**), on activation with CDI (Scheme 5). To our delight, primary amide **22a** and anilide **22b** gave the expected cycloadducts **23a** and **23b**, respectively, in excellent diastereoselectivity (trans-diastereomers as judged from the *^3^J* coupling constants between the methine protons Table S3, ESI) albeit in low yield. Disappointingly, tertiary anilides **22c**,**d** gave no reaction with the imine component. Instead, isochromenones **24a** and **24b** were isolated, also in mediocre yields. This result signifies the higher propensity of the less C-H-acidic and more *O*-nucleophilic anilides **22c**,**d** to undergo the known [25] intramolecular cyclodehydration, rather than to interact, intermolecularly, with the imine component. Overall, it can be concluded that the involvement of monoamides **22** in the new type of the CCR discussed herein is possible but appears less practical compared to the same reaction of nitriles **12** or the amidation of the traditional carboxylic acid CCR products.

**Scheme 6:**
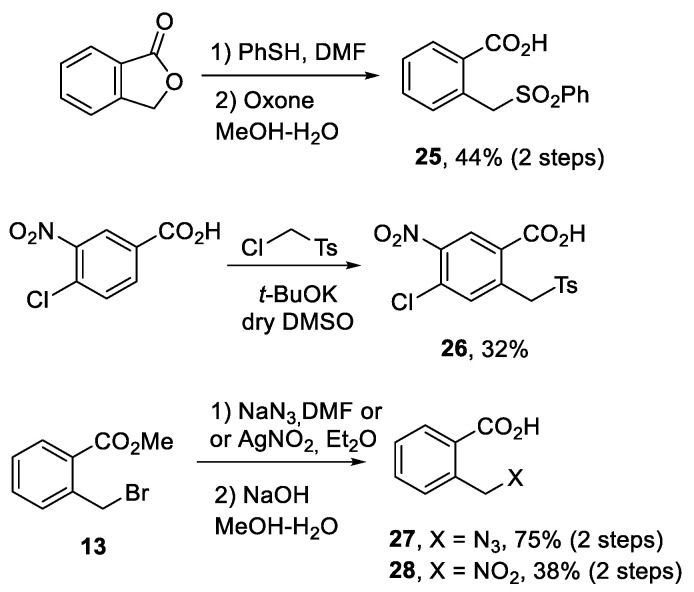
Synthesis of sulfone (**25** [26]–**26** [17]), azide (**27** [27]) and nitro (**28** [28]) compounds as potential substrates for the new type of the CCR.

**Scheme 7:**
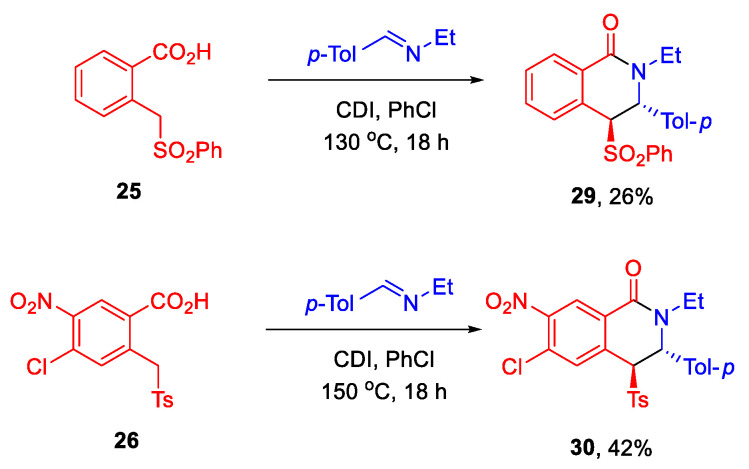
Sulfones **25** and **26** in the new type of CCR.

To our delight, sulfones **25** and **26** proved to be valid substrates for the new type of CCR under investigation (Scheme 6). The hitherto unknown CCR-derived sulfones **29** and **30** were obtained in low yield as single (trans) diastereomers as judged from the *^3^J* coupling constants between the methine protons (Scheme 7 and Table S3, ESI). However, despite the moderate yields of the sulfone adducts, in our view, this approach carries much practicality as synthesizing such Castagnoli–Cushman-type sulfones from the traditional carboxylic acid CCR adducts would presumably require a sequence of several steps, including decarboxylation, which may deliver these compounds in an even lower yield, if any.

Unfortunately, all attempts to react azidoalkyl (**27**) and nitroalkyl (**28**) substrates (Scheme 6) with imines in the Castagnoli–Cushman fashion failed, thereby signifying clear limitations of this variant of CCR at the level of electron-withdrawing groups in the α-position of *o*-methyl benzoic acid substrates.

**Scheme 8:**
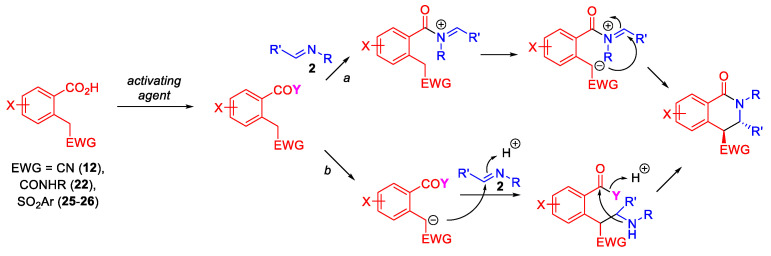
Plausible mechanism for the new type of CCR of substrates **12**, **22**, **25** and **26**.

Mechanistically, the new variant of the CCR of substrates **12**, **22**, **25** and **26** (which successfully delivered tetrahydroisoquinolone adducts) is thought to involve the initial activation of the carboxylic function by either CDI or acetic anhydride. The activated substrate can then interact with the imine via two possible pathways [29]: (a) via an *N*-acylation of the imine component followed by the deprotonation of the α-C-H position and intramolecular Mannich-type cyclization; or (b) via the deprotonation of the α-C-H position first intermolecular Mannich-type reaction with the imine component, followed by intramolecular *N*-acylation of the resulting secondary amine intermediate by the activated carboxylic acid function (Scheme 8).

## 3. Materials and Methods

### 3.1. General Information

NMR spectroscopic data were recorded with a 400 MHz (400.13 MHz for ^1^H and 100.61 MHz for ^13^C{1H}, and 376 MHz for ^19^F{1H}) or 500 MHz (500.03 MHz for ^1^H{1H} and 125.73 MHz for ^13^C{1H}) spectrometer (Bruker Avance III, Billerica, MA, USA) in CDCl_3_ and DMSO-*d_6_* and were referenced to residual solvent proton signals (δ_H_ = 7.26, and δ_H_ = 2.50, respectively) and solvent carbon signals (δ_C_ = 77.0, δC = 39.5, respectively). Mass spectra were recorded with a HRMS-ESI-qTOF spectrometer Nexera LCMS-9030 (electrospray ionization mode) (Shimadzu Europa, Duisburg, Germany). Chlorobenzene (PhCl) was distilled from P_2_O_5_ and dried over molecular sieves 4Å (>24 h). Column chromatography was carried out on silica gel grade 60 (0.040−0.063 mm) 230−400 mesh. Kieselgel on aluminum was used for TLC (UV 254). All imines were prepared in our laboratory according to common protocol for condensation of equimolar amounts of amine with aldehyde in dichloromethane (DCM) (overnight, r.t.) in the presence of anhydrous MgSO_4_.

### 3.2. Synthesis of Compounds ***12a**–**c***

#### 3.2.1. 2-(Cyanomethyl)benzoic Acid (**12a**)

Prepared according to [16]. White powder. Yield 0.63 g (62%). ^1^H NMR (400 MHz, DMSO-*d_6_*): δ = 13.29 (s, 1H), 7.99 (dd, *J* = 7.8, 1.5 Hz, 1H), 7.63 (td, *J* = 7.5, 1.5 Hz, 1H), 7.56 (d, *J* = 7.0 Hz, 1H), 7.49 (td, *J* = 7.5, 1.4 Hz, 1H), 4.28 ppm (s, 2H). ^13^C NMR (101 MHz, DMSO-*d*_6_) δ 167.6, 132.8, 132.4, 131.1, 130.6, 129.6, 128.3, 118.9, 22.3. HRMS(ESI): *m*/*z* calcd for C_9_H_7_NO_2_ + Na^+^: 184.0369 [M + Na]^+^; found: 184.0368.

#### 3.2.2. 2-(Cyanomethyl)-5-nitrobenzoic Acid (**12b**)

Prepared according to modified procedure from [17]. First, 3-nitrobenzoic acid (0.835 g, 5 mmol) was added to an intensively stirred suspension of powdered NaOH (1.0 g, 25 mmol) in 10 mL of dry DMSO, while the temperature was kept at 18 °C. After 5 min, 2-(phenylthio)acetonitrile (0.750 g, 5 mmol) in 10 mL of DMSO was added dropwise. The mixture was stirred at 18 °C for 50 min, poured over ice (50 g) and HCl conc. (10 mL in 90 mL of H_2_O) and extracted with ethyl acetate (3 × 50 mL). The organic layer was washed with sat. aq. K_2_CO_3_ (2 × 50 mL), the resulting water layer was extracted with DCM (3 × 25 mL), acidified with HCl conc. to pH = 2 and extracted again with EtOAc (3 × 50 mL), washed with water (100 mL) and dried over Na_2_SO_4_. The solvent was removed under reduced pressure and the product was crystallized from EtOAc/hexane mixture.

Yellow powder. Yield 0.65 g (63%).^1^H NMR (400 MHz, DMSO-*d*_6_): δ = 14.01 (s, 1H), 8.66 (d, *J* = 2.6 Hz, 1H), 8.48 (dd, *J* = 8.5, 2.6 Hz, 1H), 7.87 (d, *J* = 8.5 Hz, 1H), 4.47 ppm (s, 2H).^13^C NMR (101 MHz, DMSO-*d_6_*):δ = 166.3, 147.5, 139.9, 132.7, 131.5, 127.5, 126.0, 118.6, 22.9 ppm. HRMS(ESI): *m*/*z* calcd forC_9_H_6_N_2_O_4_ + Na^+^: 229.0220 [M + Na]^+^; found: 229.0218.

#### 3.2.3. 2-(Cyanomethyl)-5-methoxybenzoic Acid (**12c**)

Prepared according to modified procedure from [18]. First, 6-Methoxy indenone oxime (116 mg, 0.61 mmol) was added to 10% aq. NaOH (5 mL) and the mixture was heated at 50 °C. Tosyl chloride (139 mg, 0.73 mmol) was added in portions and the reaction was heated at 80 °C for 15 min. Then it was allowed to cool down. The resulting precipitate was filtered off and the filtrate was acidified with HCl conc. to pH = 2, followed by extraction with EtOAc (3 × 10 mL). The combined organic extracts were dried over Na_2_SO_4_, filtered and concentrated under reduced pressure to give yellow solid.

Yield 67 mg (58%). ^1^H NMR (400 MHz, DMSO-*d_6_*): δ = 12.60 (s, 1H), 7.53 (d, *J* = 8.3 Hz, 1H), 7.32 (dd, *J* = 8.4, 2.6 Hz, 1H), 7.21 (d, *J* = 2.5 Hz, 1H), 3.83 (s, 3H), 3.69 ppm (s, 2H). ^13^C NMR (126 MHz, CDCl_3_): δ = 194.3, 164.4, 160.1, 144.9, 143.9, 133.4, 129.5, 110.8, 60.8, 32.8 ppm. HRMS (ESI): *m*/*z* calcd for C_10_H_9_NO_3_ + Na^+^: 214.0475 [M + Na]^+^; found: 214.0477.

### 3.3. General Procedure for the Synthesis of Tetrahydroisoquinolone Carbonitriles ***18a**–**j*** and Dihydroisoquinolines ***19a**,**b***

Corresponding 2-(cyanomethyl)benzoic acid **12a**–**c** ( 0.25 mmol) was dissolved in PhCl (1.25 mL). CDI (49.0 mg, 1.2 eq) and corresponding imine (0.25 mmol) were added, the vial was flushed with argon and heated at 130 °C overnight. The solvent was evaporated. The residue was purified by flash column chromatography on SiO_2_, using gradient of acetone in hexane from 1:6 to 1:2.

#### 3.3.1. (±)-(3S,4S)-2-Butyl-3-(4-methoxyphenyl)-1-oxo-1,2,3,4-tetrahydroisoquinoline-4-carbonitrile (**18a**)

Pale yellow oil. Yield 42 mg (51%, dr > 20:1). ^1^H NMR (400 MHz, CDCl_3_): δ = 8.22 (dd, *J* = 7.4, 1.6 Hz, 1H), 7.46 (dtd, *J* = 17.0, 7.5, 1.5 Hz, 2H), 7.13 (dd, *J* = 7.4, 1.4 Hz, 1H), 6.99–6.95 (m, 2H), 6.79–6.70 (m, 2H), 5.04 (d, *J* = 2.5 Hz, 1H), 4.23 (ddd, *J* = 13.6, 8.8, 7.1 Hz, 1H), 4.13 (d, *J* = 2.5 Hz, 1H), 3.72 (s, 3H), 2.78 (ddd, *J* = 13.9, 8.7, 5.5 Hz, 1H), 1.79–1.57 (m, 2H), 1.49–1.32 (m, 2H), 0.93 ppm (t, *J* = 7.3 Hz, 3H).^13^C NMR (101 MHz, CDCl_3_): δ = 163.0, 159.8, 132.8, 129.8, 129.1, 128.9, 128.7, 128.2, 127.8, 127.7, 118.3, 114.4, 61.4, 55.3, 46.1, 37.4, 30.0, 20.2, 13.8 ppm. HRMS(ESI): *m*/*z* calcd for C_21_H_22_N_2_O_2_ + H^+^: 335.1760 [M + H]^+^; found: 335.1756.

#### 3.3.2. (±)-3-(4-Fluorophenyl)-2-(4-methoxybenzyl)-1-oxo-1,2,3,4-tetrahydroisoquinoline-4-carbonitrile (**18b**)

Yellow oil. Yield 54 mg (56%, dr 1:1). HRMS(ESI): *m*/*z* calcd for C_24_H_19_FN_2_O_2_+H^+^: 387.1509 [M + H]^+^; found: 387.1506. Pure isomers were isolated after second column chromatography. *Trans-isomer, (±)-(3S,4S)*: ^1^H NMR (400 MHz, CDCl_3_): δ = 8.33 (dd, *J* = 7.6, 1.6 Hz, 1H), 7.54 (dtd, *J* = 19.9, 7.5, 1.5 Hz, 2H), 7.29–7.08 (m, 3H), 7.03–6.81 (m, 6H), 5.71 (d, *J* = 14.8 Hz, 1H), 4.95 (d, *J* = 2.6 Hz, 1H), 4.05 (d, *J* = 2.6 Hz, 1H), 3.82 (s, 3H), 3.73 ppm (d, *J* = 14.7 Hz, 1H); ^13^C NMR (101 MHz, CDCl_3_):δ (trans) = 163.1, 162.7 (d, *J* = 248.9 Hz), 159.5, 133.2, 131.8 (d, *J* = 3.2 Hz), 130.0, 129.3, 128.6 (d, *J* = 17.0 Hz), 128.4 (d, *J* = 8.4 Hz), 127.8, 127.6, 117.7, 116.2 (d, *J* = 21.7 Hz), 114.2, 59.4, 55.3, 48.0, 37.1 ppm; ^19^F NMR (376 MHz, CDCl_3_): δ = −112.30 ppm;

Crystal Data for C_24_H_19_FN_2_O_2_ (*M* = 386.41 g/mol): monoclinic, space group P2_1_/n (no. 14), *a* = 8.51030(10) Å, *b* = 8.6727(2) Å, *c* = 26.2561(5) Å, *β* = 94.418(2), *V* = 1932.13(6) Å^3^, *Z* = 4, *T* = 100.15 K, μ(CuKα) = 0.750 mm^−1^, *Dcalc* = 1.328 g/cm^3^, 28288 reflections measured (6.754° ≤ 2Θ ≤ 160.042°), 4123 unique (*R*_int_ = 0.0501, R_sigma_ = 0.0296), which were used in all calculations. The final *R*_1_ was 0.1293 (I > 2σ(I)) and *wR*_2_ was 0.3425 (all data). Deposition Number 2192610 contains the supplementary crystallographic data for this paper. These data are provided free of charge by the joint Cambridge Crystallographic Data Centre (http://www.ccdc.cam.ac.uk/structures, accessed on 22 September 2022) and Fachinformationszentrum Karlsruhe Access Structures service.

Cis-isomer, (±)-(3S,4R): ^1^H NMR (400 MHz, CDCl_3_): δ = 8.33–8.26 (m, 1H), 7.61–7.55 (m, 2H), 7.38 (ddd, *J* = 4.8, 2.8, 1.2 Hz, 1H), 7.25–7.20 (m, 2H), 7.08–7.01 (m, 2H), 7.00–6.87 (m, 4H), 5.67 (d, *J* = 14.6 Hz, 1H), 4.83 (d, *J* = 5.9 Hz, 1H), 4.72 (dd, *J* = 5.9, 1.1 Hz, 1H), 3.85 (s, 3H), 3.58 ppm (d, *J* = 14.7 Hz, 1H). ^13^C NMR (101 MHz, CDCl_3_) δ = 163.1 (d, *J* = 248.9 Hz), 162.9, 159.6, 133.2, 129.8, 129.7 (d, *J* = 8.4 Hz), 129.5, 128.8, 128.4, 128.1 (d, *J* = 6.6 Hz), 126.3, 116.1, 116.0, 115.7, 114.4, 58.3, 55.4, 47.9, 37.7 ppm. ^19^F NMR (376 MHz, CDCl_3_): δ = −112.05 ppm.

#### 3.3.3. (±)-(3S,4S)-2-Ethyl-1-oxo-3-(p-tolyl)-1,2,3,4-tetrahydroisoquinoline-4-carbonitrile (**18c**)

Yellow oil. Yield 67 mg (46%, dr 3.3:1), performed at 0.5 mmol of **12a**. 

^1^H NMR (400 MHz, CDCl_3_): δ = 8.23 (dd, *J* = 7.5, 1.7 Hz, 1H), 7.61–7.48 (m, 2H), 7.48–7.40 (m, 1H), 7.18–7.10 (m, 1H), 7.05 (d, *J* = 9.7 Hz, 2H), 6.96 (d, *J* = 7.9 Hz, 2H), 5.04 (d, *J* = 2.7 Hz, 1H), 4.24 (dq, *J* = 14.4, 7.2 Hz, 1H), 4.14 (d, *J* = 2.7 Hz, 1H), 2.95 (dq, *J* = 14.1, 7.1 Hz, 1H), 2.27 (s, 3H), 1.28 ppm (t, *J* = 7.1 Hz, 3H). ^13^C NMR (126 MHz, CDCl_3_) δ 162.8, 138.8, 133.5, 132.8, 129.8, 129.3, 128.9, 128.6, 128.4, 127.7, 126.3, 118.2, 61.6, 41.3, 37.5, 21.0, 13.0 ppm. HRMS(ESI): *m*/*z* calcd for C_19_H_18_N_2_O + Na^+^: 313.1311 [M + Na]^+^; found: 313.1294.

#### 3.3.4. (±)-(3S,4S)-2-Ethyl-7-methoxy-1-oxo-3-(p-tolyl)-1,2,3,4-tetrahydroisoquinoline-4-carbonitrile (**18d**)

Yellow oil. Yield 36 mg (45%, dr 4.4:1). ^1^H NMR (400 MHz, CDCl_3_): δ (major isomer) *=* 7.75 (d, *J* = 2.7 Hz, 1H), 7.07 (t, *J* = 7.3 Hz, 2H), 6.97 (td, *J* = 5.6, 2.6 Hz, 4H), 5.04 (d, *J* = 2.4 Hz, 1H), 4.25 (dq, *J* = 14.3, 7.2 Hz, 1H), 4.11 (d, *J* = 2.5 Hz, 1H), 3.88 (s, 3H), 2.97 (dq, *J* = 14.2, 7.2 Hz, 1H), 2.29 (d, *J* = 2.4 Hz, 3H), 1.30 ppm (t, *J* = 7.2 Hz, 3H); δ (minor isomer)*=* 7.75 (d, *J* = 2.7 Hz, 1H), 7.07 (t, *J* = 7.3 Hz, 2H), 6.97 (td, *J* = 5.6, 2.6 Hz, 4H),4.91 (d, *J* = 5.8 Hz, 1H), 4.85 (dd, *J* = 5.7, 1.1 Hz, 1H), 4.03 (dt, *J* = 13.8, 7.0 Hz, 1H), 3.90 (s, 3H), 3.10 (dq, *J* = 14.1, 7.1 Hz, 1H), 2.28 (s, 3H), 1.21 ppm (t, *J* = 7.2 Hz, 3H).^13^C NMR (101 MHz, CDCl_3_): δ (both isomers) = 162.7, 160.6, 160.3, 139.0, 138.6, 133.6, 131.3, 130.4, 129.8, 129.7, 129.6, 129.3, 129.0, 127.7, 127.6, 126.3, 125.8, 120.5, 120.3, 119.8, 119.3, 118.5, 116.7, 112.5, 61.7, 60.6, 55.6, 55.6, 41.9, 41.5, 37.5, 36.8, 21.06, 21.0, 19.7, 14.1, 13.4, 13.0 ppm. HRMS(ESI): *m*/*z* calcd for C_20_H_20_N_2_O_2_ + H^+^: 321.1603 [M + H]^+^; found: 321.1600.

#### 3.3.5. (±)-(3S,4S)-2-Cyclopropyl-1-oxo-3-phenyl-1,2,3,4-tetrahydroisoquinoline-4-carbonitrile (**18e**)

Light yellow powder. Yield 28 mg (39%, dr 5:1). ^1^H NMR (400 MHz, CDCl_3_): δ (major isomer)= 8.26 (dd, *J* = 7.4, 1.9 Hz, 1H), 7.56–7.40 (m, 1H), 7.31–7.19 (m, 4H), 7.14–7.10 (m, 3H), 5.19 (d, *J* = 1.9 Hz, 1H), 4.19 (d, *J* = 1.9 Hz, 1H), 2.87–2.79 (m, 1H), 1.16 (dtd, *J* = 8.3, 7.3, 5.7 Hz, 1H), 1.09–0.99 (m, 1H), 0.84–0.75 ppm (m, 2H); δ (minor isomer) =8.26 (dd, *J* = 7.4, 1.9 Hz, 1H), 7.56–7.40 (m, 1H), 7.31–7.19 (m, 4H), 7.14–7.10 (m, 3H), 5.06 (d, *J* = 5.7 Hz, 1H), 4.91 (dd, *J* = 5.8, 1.2 Hz, 1H), 2.78–2.71 (m, 1H), 0.93–0.84 (m, 2H), 0.69–0.62 ppm (m, 2H).^13^C NMR (101 MHz, CDCl_3_):δ (both isomers) = 164.8, 164.7, 136.7, 134.5, 133.1, 133.1, 129.8, 129.2, 129.2, 129.1, 129.0, 128.9, 128.7, 128.6, 128.6, 128.5, 128.4, 128.3, 127.9, 127.6, 126.2, 126.1, 118.2, 116.4, 63.6, 62.3, 38.1, 37.3, 30.3, 30.2, 29.3, 9.2, 8.5, 6.8, 6.4 ppm. HRMS(ESI): *m*/*z* calcd for C_19_H_16_N_2_O + H^+^: 289.1335[M + H]^+^; found: 289.1338.

#### 3.3.6. (±)-(3S,4S)-2-Benzyl-1-oxo-3-phenyl-1,2,3,4-tetrahydroisoquinoline-4-carbonitrile (**18f**)

Yellow oil. Yield 30 mg (36%, dr 6.9:1). ^1^H NMR (400 MHz, CDCl_3_) δ (major isomer) = 8.34 (dd, *J* = 7.7, 1.6 Hz, 1H), 7.55 (td, *J* = 7.5, 2.2 Hz, 1H), 7.50 (td, *J* = 7.5, 1.5 Hz, 1H), 7.36 (qd, *J* = 6.7, 2.4 Hz, 5H), 7.29 (dd, *J* = 5.0, 2.1 Hz, 3H), 7.16 (d, *J* = 7.4 Hz, 1H), 7.03 (dd, *J* = 6.7, 2.8 Hz, 2H), 5.83 (d, *J* = 14.9 Hz, 1H), 4.97 (d, *J* = 2.5 Hz, 1H), 4.11 (d, *J* = 2.5 Hz, 1H), 3.75 ppm (d, *J* = 14.9 Hz, 1H); δ (minor isomer) = 8.32–8.30 (m, 1H), 7.85–7.80 (m, 1H), 7.11–7.05 (m, 2H), 5.77 (d, *J* = 14.9 Hz, 1H), 4.85 (d, *J* = 6.0 Hz, 1H), 4.78 (d, *J* = 5.9 Hz, 1H), 3.62 ppm(d, *J* = 14.8 Hz, 1H).^13^C NMR (101 MHz, CDCl_3_): δ (both isomers) = 163.3, 163.1, 136.7, 136.0, 135.9, 133.6, 133.2, 133.1, 129.9, 129.4, 129.3, 129.3, 129.2, 129.0, 128.9, 128.8, 128.7, 128.6, 128.4, 128.1, 128.0, 127.9, 127.9, 127.0, 126.6, 126.3, 117.9, 116.2, 60.3, 59.2, 48.7, 48.5, 37.7, 37.0, 30.9 ppm. HRMS(ESI): *m*/*z* calcd for C_23_H_18_N_2_O + Na^+^: 361.1311[M + Na]^+^; found: 361.1314.

#### 3.3.7. (±)-(3S,4S)-2-Benzyl-1-oxo-3-(p-tolyl)-1,2,3,4-tetrahydroisoquinoline-4-carbonitrile (**18g**)

Yellow oil. Yield 26 mg (30%, dr > 20:1). ^1^H NMR (400 MHz, CDCl_3_): δ = 8.34 (dd, *J* = 7.5, 1.6 Hz, 1H), 7.53 (dtd, *J* = 21.5, 7.5, 1.5 Hz, 2H), 7.43–7.30 (m, 5H), 7.16 (dd, *J* = 7.5, 1.3 Hz, 1H), 7.09 (d, *J* = 7.9 Hz, 2H), 6.92 (d, *J* = 7.9 Hz, 2H), 5.83 (d, *J* = 14.9 Hz, 1H), 4.93 (d, *J* = 2.5 Hz, 1H), 4.08 (d, *J* = 2.5 Hz, 1H), 3.72 (d, *J* = 14.9 Hz, 1H), 2.31 ppm (s, 3H).^13^C NMR (101 MHz, CDCl_3_):δ = 163.3, 138.9, 136.0, 133.1, 132.9, 129.8, 129.2, 128.8, 128.8, 128.8, 128.6, 128.0, 127.9, 126.5, 117.9, 60.1, 48.5, 37.1, 21.0 ppm. HRMS(ESI): *m*/*z* calcd for C_24_H_20_N_2_O + H^+^: 353.1654 [M + H]^+^; found: 353.1649.

#### 3.3.8. (±)-(3S,4S)-2-Methyl-1-oxo-3-(3-(trifluoromethyl)phenyl)-1,2,3,4-tetrahydroisoquinoline-4-carbonitrile (**18h**)

Yellow oil. Yield 28 mg (34%, dr 3.5:1).^1^H NMR (400 MHz, CDCl_3_): δ (major isomer) = 8.25 (td, *J* = 7.5, 2.5 Hz, 1H), 7.62–7.48 (m, 2H), 7.48–7.36 (m, 3H), 7.30–7.17 (m, 2H), 5.12 (d, *J* = 3.2 Hz, 1H), 4.22 (d, *J* = 3.2 Hz, 1H), 3.19 ppm (s, 3H); δ (minor isomer) = 8.25 (td, *J* = 7.5, 2.5 Hz, 1H), 7.62–7.48 (m, 2H), 7.48–7.36 (m, 3H), 7.30–7.17 (m, 2H), 5.05 (d, *J* = 5.9 Hz, 1H), 5.00 (d, *J* = 5.9 Hz, 1H), 3.15 ppm (s, 3H).^13^C NMR (101 MHz, CDCl_3_):δ (both isomers) = 163.2, 163.2, 137.4, 134.9, 133.2, 133.1, 132.0, 131.7, 131.4 (d, *J* = 7.8 Hz), 130.8, 130.1, 129.9, 129.6, 129.6, 129.4, 129.1, 128.5, 128.3 (d, *J* = 15.3 Hz), 128.1, 128.0 (d, *J* = 15.4 Hz, 126.3, 126.3, 126.0 (q, *J* = 3.6 Hz), 124.8, 124.7 (d, *J* = 3.7 Hz), 123.4 (q, *J* = 3.8 Hz), 122.1, 117.8, 115.9, 64.0, 62.8, 37.3, 37.1, 34.7, 34.4 ppm.^19^F NMR (376 MHz, CDCl_3_): δ (major isomer) = −62.82 ppm; δ (minor isomer) = −62.93 ppm. HRMS(ESI): *m*/*z* calcd for C_18_H_13_F_3_N_2_O + H^+^: 331.1058 [M + H]^+^; found: 331.1054.

#### 3.3.9. (±)-(3S,4S)-3-(2-Chlorophenyl)-1-oxo-2-propyl-1,2,3,4-tetrahydroisoquinoline-4-carbonitrile (**18i**)

White amorphous powder. Yield 21 mg (26%, dr 10.4:1). ^1^H NMR (400 MHz, CDCl_3_): δ = 8.26 (dd, *J* = 7.6, 1.6 Hz, 1H), 7.56–7.38 (m, 3H), 7.24 (td, *J* = 7.7, 1.7 Hz, 1H), 7.16–7.04 (m, 2H), 6.84 (dd, *J* = 7.8, 1.6 Hz, 1H), 5.46 (d, *J* = 1.7 Hz, 1H), 4.33 (d, *J* = 1.7 Hz, 1H), 4.23 (dt, *J* = 13.5, 7.9 Hz, 1H), 2.73 (dt, *J* = 13.9, 7.1 Hz, 1H), 1.79 (h, *J* = 7.5 Hz, 2H), 1.04 ppm (t, *J* = 7.4 Hz, 3H).^13^C NMR (101 MHz, CDCl_3_): δ = 163.4, 133.3, 133.0, 132.6, 130.4, 130.1, 129.9, 129.0, 128.8, 128.4, 128.1, 127.8, 127.3, 118.0, 59.2, 48.3, 34.4, 21.3, 11.4 ppm. HRMS(ESI): *m*/*z* calcd for C_19_H_17_ClN_2_O + H^+^: 325.1103 [M + H]^+^; found: 325.1103.

#### 3.3.10. (±)-(3S,4S)-2-(4-Methoxyphenyl)-1-oxo-3-(p-tolyl)-1,2,3,4-tetrahydroisoquinoline-4-carbonitrile (**18j**)

Yellow amorphous powder. Yield 19 mg (21%, dr 4.4:1).^1^H NMR (400 MHz, CDCl_3_): δ (both isomers) = 8.31 (dd, *J* = 7.2, 1.9 Hz, 1H), 8.29 (d, *J* = 2.2 Hz, 1H), 7.54 (pd, *J* = 7.6, 1.6 Hz, 2H), 7.29–7.23 (m, 2H), 7.20 (dd, *J* = 7.1, 1.6 Hz, 4H), 7.09–7.02 (m, 5H), 6.91–6.87 (m, 2H), 5.37 (d, *J* = 2.4 Hz, 1H), 5.23 (d, *J* = 5.6 Hz, 1H), 5.19 (d, *J* = 5.5 Hz, 1H), 4.22 (d, *J* = 2.4 Hz, 1H), 3.79 (s, 3H), 3.80 (s, 3H), 2.30 (s, 3H), 2.28 ppm (s, 3H).^13^C NMR (101 MHz, CDCl_3_):δ (both isomers) =163.1, 163.0, 158.7, 139.0, 138.7, 134.4, 133.8, 133.3, 133.2, 129.9, 129.7, 129.4, 129.3, 129.1, 128.9, 127.9, 127.8, 127.8, 126.5, 126.4, 124.3, 118.4, 114.6, 114.5, 65.9, 64.6, 55.5, 38.5, 37.8, 21.10, 21.0 ppm. HRMS (ESI): *m*/*z* calcd for C_24_H_20_N_2_O_2_ + H^+^: 369.1598 [M + H]^+^; found: 369.1599.

#### 3.3.11. (±)-(3S,4S)-2-Ethyl-7-nitro-1-oxo-3-(p-tolyl)-1,2-dihydroisoquinoline-4-carbonitrile (**19a**)

White powder. Yield 41 mg (49%). ^1^H NMR (400 MHz, CDCl_3_): δ = 9.32 (d, *J* = 2.4 Hz, 1H), 8.59 (dd, *J* = 8.8, 2.4 Hz, 1H), 8.02 (d, *J* = 8.8 Hz, 1H), 7.44 (d, *J* = 7.9 Hz, 2H), 7.37 (d, *J* = 8.1 Hz, 2H), 4.02 (q, *J* = 7.0 Hz, 2H), 2.51 (s, 3H), 1.22 ppm (t, *J* = 7.1 Hz, 3H). ^13^C NMR (126 MHz, CDCl_3_): δ = 160.5, 156.5, 146.9, 141.5, 138.1, 130.1, 129.1, 128.0, 127.7, 125.8, 124.7, 124.4, 115.2, 91.9, 42.6, 21.6, 14.1 ppm. HRMS (ESI): *m*/*z* calcd for C_19_H_15_N_3_O_3_ + H^+^: 334.1192 [M + H]^+^; found: 334.1190.

#### 3.3.12. (±)-(3S,4S)-2-Cyclopropyl-7-nitro-1-oxo-3-phenyl-1,2-dihydroisoquinoline-4-carbonitrile (**19b**)

White powder. Yield 12 mg (24%) from 0.15 mmol.^1^H NMR (400 MHz, CDCl_3_):δ = 9.28 (d, *J* = 2.4 Hz, 1H), 8.59 (dd, *J* = 8.8, 2.4 Hz, 1H), 8.04 (d, *J* = 8.8 Hz, 1H), 7.66–7.54 (m, 5H), 3.02 (tt, *J* = 7.1, 4.2 Hz, 1H), 0.97–0.86 (m, 2H), 0.61–0.51 ppm (m, 2H). ^13^C NMR (126 MHz, CDCl_3_):δ = 162.2, 157.5, 147.0, 138.1, 132.4, 131.0, 129.0, 128.9, 128.7, 127.7, 126.0, 124.8, 124.5, 115.3, 91.8, 31.9, 11.3 ppm.HRMS (ESI): *m*/*z* calcd for C_19_H_13_N_3_O_3_ + Na^+^: 354.0849 [M + Na]^+^; found: 354.0845.

### 3.4. General Procedure for the Synthesis of 7-Nitro Tetrahydroisoquinolone Carbonitriles (***18k**−**q**)*

2-(Cyanomethyl)-5-nitrobenzoic acid **12b** (51.2 mg, 0.25 mmol) was dissolved in PhCl (1.25 mL). Ac_2_O (31.0 mg, 1.2 eq) and corresponding imine (0.25 mmol) were added, the vial was flushed with argon and heated at 60 °C with occasional TLC monitoring of the reaction. After 2 h, the starting material was fully consumed. The solvent was evaporated. The residue was purified by flash column chromatography on SiO_2_, using a gradient of acetone in hexane from 1:10 to 1:3.

#### 3.4.1. (±)-(3S,4S)-2-Ethyl-7-nitro-1-oxo-3-(p-tolyl)-1,2,3,4-tetrahydroisoquinoline-4-carbonitrile (**18k**)

Yellow oil. Yield 39 mg (46%, dr 1.9:1). ^1^H NMR (400 MHz, CDCl_3_): δ (major isomer) = 9.06 (dd, *J* = 4.1, 2.4 Hz, 1H), 8.31 (dd, *J* = 8.3, 2.5 Hz, 1H), 7.40 (d, *J* = 8.3 Hz, 1H), 7.10 (dd, *J* = 11.2, 8.0 Hz, 2H), 6.97 (dd, *J* = 8.2, 6.5 Hz, 2H), 5.16 (d, *J* = 2.7 Hz, 1H), 4.33 (d, *J* = 2.7 Hz, 1H), 4.27 (dt, *J* = 14.3, 7.2 Hz, 1H), 3.04 (dq, *J* = 14.2, 7.1 Hz, 1H), 2.29 (s, 3H), 1.32 ppm (t, *J* = 7.2 Hz, 3H);δ (minor isomer) = 9.06 (dd, *J* = 4.1, 2.4 Hz, 1H), 8.37 (dd, *J* = 8.4, 2.5 Hz, 1H),7.60 (dd, *J* = 8.4, 1.1 Hz, 1H),7.10 (dd, *J* = 11.2, 8.0 Hz, 2H), 6.97 (dd, *J* = 8.2, 6.5 Hz, 2H),5.05 (d, *J* = 5.8 Hz, 1H), 4.98 (dd, *J* = 5.8, 1.2 Hz, 1H), 4.04 (dq, *J* = 14.4, 7.2 Hz, 1H), 3.18 (dq, *J* = 14.3, 7.2 Hz, 1H),2.29 (s, 3H), 1.24 ppm (t, *J* = 7.2 Hz, 3H). ^13^C NMR (101 MHz, CDCl_3_):δ (both isomers) = 160.7, 160.7, 149.1, 148.8, 139.7, 139.3, 135.0, 134.9, 132.4, 130.9, 130.3, 130.3, 130.1, 129.6, 129.3, 127.9, 127.4, 127.2, 126.1, 124.1, 123.5, 116.9, 115.4, 61.4, 60.3, 42.2, 41.7, 38.4, 37.4, 21.1, 21.0, 13.3, 12.9 ppm. HRMS(ESI): *m*/*z* calcd for C_19_H_17_N_3_O_3_ + H^+^: 336.1348 [M + H]^+^; found: 336.1344.

#### 3.4.2. (±)-(3S,4S)-7-Nitro-1-oxo-2-propyl-3-(p-tolyl)-1,2,3,4-tetrahydroisoquinoline-4-carbonitrile (**18l**)

Yellow oil. Yield 18 mg (55%, dr 2.4:1) from 0.1 mmol.^1^H NMR (400 MHz, CDCl_3_):δ (both isomers) = 9.07 (d, *J* = 2.4 Hz, 1H), 9.06 (d, *J* = 2.5 Hz, 1H), 8.37 (dd, *J* = 8.4, 2.5 Hz, 1H), 8.31 (dd, *J* = 8.3, 2.4 Hz, 1H), 8.05–7.96 (m, 1H), 7.60 (dd, *J* = 8.4, 1.2 Hz, 1H), 7.39 (d, *J* = 8.3 Hz, 1H), 7.10 (t, *J* = 8.4 Hz, 3H), 6.98–6.92 (m, 3H), 5.16 (d, *J* = 2.5 Hz, 1H), 5.03 (d, *J* = 5.8 Hz, 1H), 4.97 (dd, *J* = 5.8, 1.2 Hz, 1H), 4.31 (d, *J* = 2.5 Hz, 1H), 4.24 (dt, *J* = 13.6, 7.9 Hz, 1H), 4.02 (ddd, *J* = 13.6, 9.0, 6.6 Hz, 1H), 2.97 (ddd, *J* = 13.6, 9.0, 5.8 Hz, 1H), 2.86 (dt, *J* = 13.9, 6.9 Hz, 1H), 2.30 (d, *J* = 2.4 Hz, 4H), 1.77 (p, *J* = 7.4 Hz, 2H), 1.72–1.60 (m, 2H), 1.03 (t, *J* = 7.4 Hz, 3H), 0.98 ppm (t, *J* = 7.4 Hz, 3H). ^13^C NMR (101 MHz, CDCl_3_):δ (both isomers) = 161.0, 161.0, 149.1, 148.8, 139.7, 139.3, 134.9, 132.4, 130.9, 130.4, 130.2, 130.1, 130.1, 129.6, 129.3, 129.2, 127.8, 127.4, 127.2, 126.1, 124.2, 123.6, 116.9, 115.4, 61.6, 60.6, 48.8, 48.4, 38.3, 37.2, 31.6, 22.6, 21.4, 21.2, 21.1, 21.0, 14.1, 11.4, 11.4 ppm. HRMS(ESI): *m*/*z* calcd for C_20_H_19_N_3_O_3_ + H^+^: 350.1499 [M + H]^+^; found: 350.1502.

#### 3.4.3. (±)-(3S,4S)-2-Benzyl-7-nitro-1-oxo-3-phenyl-1,2,3,4-tetrahydroisoquinoline-4-carbonitrile (**18m**)

Yellow oil. Yield 38 mg (40%, dr 1.9:1). ^1^H NMR (400 MHz, CDCl_3_):δ (major isomer) = 9.18 (d, *J* = 2.4 Hz, 1H), 8.36 (dd, *J* = 8.3, 2.4 Hz, 1H), 7.44–7.30 (m, 8H), 7.05–6.98 (m, 2H), 5.83 (d, *J* = 14.8 Hz, 1H), 5.04 (d, *J* = 2.6 Hz, 1H), 4.23 (d, *J* = 2.6 Hz, 1H), 3.82 ppm (d, *J* = 14.9 Hz, 1H); δ (minor isomer) = 9.15 (d, *J* = 2.4 Hz, 1H), 8.40 (dd, *J* = 8.4, 2.4 Hz, 1H), 7.61 (dd, *J* = 8.4, 1.1 Hz, 1H), 7.44 - 7.30 (m, 9H),7.07 -7.05 (m, 1H), 5.77 (d, *J* = 14.8 Hz, 1H), 4.94 (d, *J* = 5.9 Hz, 1H), 4.81 (dd, *J* = 6.0, 1.1 Hz, 1H), 3.68 ppm (d, *J* = 14.8 Hz, 1H).^13^C NMR (101 MHz, CDCl_3_):δ (both isomers)= 161.3, 161.1, 149.2, 148.9, 136.0, 135.2, 135.0, 134.9, 132.7, 130.5, 129.9, 129.8, 129.5, 129.4, 129.3, 129.2, 129.1, 129.0, 128.9, 128.7, 128.5, 128.4, 128.3, 128.0, 127.8, 127.7, 127.5, 126.3, 124.5, 123.9, 116.6, 115.2, 60.0, 59.0, 49.0, 48.9, 38.0, 37.0 ppm. HRMS (ESI): *m*/*z* calcd for C_23_H_17_N_3_O_3_ + H^+^: 384.1348[M + H]^+^; found: 384.1348. 

#### 3.4.4. (±)-(3S,4S)-2-(4-Methoxyphenyl)-7-nitro-1-oxo-3-phenyl-1,2,3,4-tetrahydroisoquinoline-4-carbonitrile (**18n**)

Yellow viscous oil. Yield 44 mg (44%, dr 2.2:1). ^1^H NMR (400 MHz, CDCl_3_):δ (both isomers) = 9.11 (d, *J* = 2.4 Hz, 1H), 9.10 (d, *J* = 2.4 Hz, 1H), 8.41 (dd, *J* = 8.4, 2.4 Hz, 1H), 8.35 (dd, *J* = 8.3, 2.4 Hz, 1H), 7.65 (dd, *J* = 8.5, 1.2 Hz, 1H), 7.43 (d, *J* = 8.3 Hz, 2H), 7.39 (d, *J* = 9.0 Hz, 2H), 7.32–7.28 (m, 3H), 7.26 (d, *J* = 8.9 Hz, 2H), 7.19–7.14 (m, 3H), 7.10 (d, *J* = 8.9 Hz, 1H), 6.94–6.88 (m, 2H), 6.88–6.81 (m, 2H), 5.50 (d, *J* = 2.4 Hz, 1H), 5.36 (d, *J* = 5.6 Hz, 1H), 5.28 (d, *J* = 5.5 Hz, 1H), 4.41 (d, *J* = 2.5 Hz, 1H), 3.80 (s, 3H), 3.79 (s, 3H), 2.14 ppm (s, 3H).^13^C NMR (101 MHz, CDCl_3_):δ (both isomers) = 168.4, 161.1, 161.0, 159.0, 159.0, 156.4, 149.2, 148.8, 135.7, 135.4, 135.3, 133.7, 133.5, 133.4, 131.1, 131.1, 130.5, 129.6, 129.6, 129.4, 129.2, 129.0, 128.0, 127.7, 127.7, 127.6, 127.6, 126.4, 124.5, 124.0, 121.9, 117.2, 115.3, 114.8, 114.7, 114.1, 65.8, 64.5, 55.5, 55.5, 38.6, 37.6, 24.3 ppm. HRMS (ESI): *m*/*z* calcd for C_24_H_19_N_3_O_4_ + H^+^: 414.1448 [M + H]^+^; found: 414.1450.

#### 3.4.5. (±)-(3S,4S)-7-Nitro-3-(2-nitrophenyl)-1-oxo-2-propyl-1,2,3,4-tetrahydroisoquinoline-4-carbonitrile (**18o**)

Orange oil. Yield 44 mg (46%, dr 3.4:1). ^1^H NMR (400 MHz, CDCl_3_):δ (major isomer) = 9.08 (d, *J* = 2.4 Hz, 1H), 8.33 (dd, *J* = 8.3, 2.4 Hz, 1H), 8.17 (dd, *J* = 8.1, 2.1 Hz, 1H), 8.08 (d, *J* = 2.0 Hz, 1H), 7.50 (td, *J* = 8.0, 5.5 Hz, 1H), 7.38 (dd, *J* = 22.5, 7.9 Hz, 2H), 5.30 (d, *J* = 2.3 Hz, 1H), 4.36 (d, *J* = 2.3 Hz, 1H), 4.29 (dt, *J* = 13.7, 7.9 Hz, 1H), 2.90–2.80 (m, 1H), 1.84–1.72 (m, 2H), 1.03 ppm (t, *J* = 7.4 Hz, 3H); δ (minor isomer) = 9.08 (d, *J* = 2.4 Hz, 1H),8.40 (dd, *J* = 8.4, 2.5 Hz, 1H), 8.22–8.19 (m, 1H), 8.04 (t, *J* = 2.0 Hz, 1H), 7.60 (dd, *J* = 8.4, 1.1 Hz, 1H), 7.51–7.47 (m, 2H), 5.17 (d, *J* = 5.8 Hz, 1H), 5.04 (d, *J* = 5.7 Hz, 1H), 4.04 (ddd, *J* = 13.7, 9.1, 6.5 Hz, 1H), 2.98 (ddd, *J* = 14.2, 9.1, 5.7 Hz, 1H), 1.72–1.62 (m, 2H), 0.97 ppm (t, *J* = 7.4 Hz, 3H).^13^C NMR (101 MHz, CDCl_3_):δ (both isomers) = 160.8, 149.4, 148.8, 137.9, 135.8, 134.0, 133.9, 133.0, 131.8, 130.7, 130.5, 130.3, 130.0, 129.4, 128.0, 127.6, 124.7, 124.4, 124.3, 123.9, 122.9, 121.6, 116.3, 114.9, 61.1, 60.1, 49.0, 48.6, 37.8, 36.9, 21.4, 21.2, 11.34, 11.3 ppm. HRMS (ESI): *m*/*z* calcd for C_19_H_16_N_4_O_5_ + H^+^:381.1199[M + H]^+^; found: 381.1198.

#### 3.4.6. (±)-(3S,4S)-2-Methyl-3-(naphthalen-2-yl)-7-nitro-1-oxo-1,2,3,4-tetrahydroisoquinoline-4-carbonitrile (**18p**)

Yellow oil. Yield 31 mg (35%, dr 13.4:1). ^1^H NMR (400 MHz, CDCl_3_): δ = 9.16 (d, *J* = 2.4 Hz, 1H), 8.28 (dd, *J* = 8.4, 2.4 Hz, 1H), 8.04–7.96 (m, 2H), 7.85 (d, *J* = 8.3 Hz, 1H), 7.76 (ddd, *J* = 8.4, 6.9, 1.4 Hz, 1H), 7.65 (ddd, *J* = 8.1, 6.9, 1.1 Hz, 1H), 7.37–7.27 (m, 2H), 6.99 (d, *J* = 7.2 Hz, 1H), 5.94 (d, *J* = 2.4 Hz, 1H), 4.56 (d, *J* = 2.5 Hz, 1H), 3.31ppm (s, 3H).^13^C NMR (101 MHz, CDCl_3_):δ = 161.7, 149.1, 135.0, 134.4, 130.2, 130.1, 130.0, 129.6, 129.1, 127.9, 127.3, 126.6, 125.3, 124.3, 124.2, 121.0, 117.1, 61.4, 35.1, 35.1 ppm. HRMS (ESI): *m*/*z* calcd for C_21_H_15_N_3_O_3_ + H^+^: 358.1192[M + H]^+^; found: 358.1192.

#### 3.4.7. (±)-(3S,4S)-2-Allyl-7-nitro-1-oxo-3-(thiophen-2-Yl)-1,2,3,4-tetrahydroisoquinoline-4-carbonitrile (**18q**)

Yellow oil. Yield 25 mg (30%, dr 1:1). ^1^H NMR (400 MHz, CDCl_3_):δ (both isomers) 9.08 (d, *J* = 2.4 Hz, 1H), 9.05 (d, *J* = 2.4 Hz, 1H), 8.46 (dd, *J* = 8.4, 2.5 Hz, 1H), 8.41 (dd, *J* = 8.3, 2.5 Hz, 1H), 7.74 (dd, *J* = 8.4, 1.2 Hz, 1H), 7.52 (d, *J* = 8.3 Hz, 1H), 7.20 (td, *J* = 5.2, 1.3 Hz, 2H), 7.02 (dd, *J* = 3.6, 1.2 Hz, 1H), 6.95 (dd, *J* = 5.1, 3.6 Hz, 1H), 6.91 (dd, *J* = 5.1, 3.6 Hz, 1H), 6.86 (d, *J* = 3.6 Hz, 1H), 6.02–5.80 (m, 2H), 5.47–5.30 (m, 7H), 5.10 (ddt, *J* = 15.1, 3.7, 1.7 Hz, 1H), 5.02–4.95 (m, 1H), 4.94 (dd, *J* = 5.4, 1.2 Hz, 1H), 4.39 (d, *J* = 2.3 Hz, 1H), 3.50 ppm (td, *J* = 14.6, 8.2 Hz, 2H).^13^C NMR (101 MHz, CDCl_3_): δ (both isomers) = 160.1, 160.1,149.3, 149.0, 138.6, 136.0, 135.3, 135.2, 132.0, 131.7, 130.5, 129.9, 129.6, 128.8, 128.2, 127.5, 127.5, 127.2, 127.1, 127.0, 126.9, 126.6, 124.6, 124.1, 120.6, 120.1, 116.1, 115.1, 56.4, 55.7, 48.0, 38.5, 37.1 ppm. HRMS(ESI): *m*/*z* calcd for C_17_H_13_N_3_O_3_S + Na^+^: 362.0570 [M + Na]^+^; found: 362.0572.

### 3.5. Post-Condensational Modifications

#### 3.5.1. Oxidation Protocol (Scheme 4)

Tetrahydroisoquinoline-4-carbonitrile **18k** (0.04 mmol, 14 mg) was dissolved in DMSO (0.6 mL), the solution was heated at 150 °C for 2 days. The reaction mixture was poured over the top of a SiO_2_ flash column. The product was eluted with acetone:hexane, 1:5 to afford 10 mg (71%) of compound **19a**.

#### 3.5.2. Reduction Protocol (Scheme 4). 7-Amino-2-ethyl-1-oxo-3-(p-tolyl)-1,2-dihydroisoquinoline-4-carbonitrile (**20**)

One portion of tin (II) chloride (11 mg, 0.5 mmol) was added to a stirred solution of compound **19a** (36 mg, 0.11 mmol) in ethanol (6 mL) in a screw-cap vial equipped with a magnetic stir bar. After heating for 24 h at 80 °C (oil bath temperature), the reaction mixture was cooled to room temperature and partitioned between ethyl acetate (30 mL) and 1M aq. NaOH (5 mL). The organic phase was separated, washed with water, dried over Na_2_SO_4_ and concentrated to give the pure title compound.

Yield 32 mg (97%). Beige foam. ^1^H NMR (400 MHz, CDCl_3_): δ = 7.86–7.56 (m, 2H), 7.45–7.29 (m, 4H), 7.14 (dd, *J* = 8.5, 2.6 Hz, 1H), 4.07 (s, 2H), 3.94 (q, *J* = 7.0 Hz, 1H), 2.46 (s, 3H), 1.14 ppm (t, *J* = 7.0 Hz, 3H). ^13^C NMR (101 MHz, CDCl_3_): δ = 161.3, 149.1, 146.8, 140.4, 130.2, 129.8, 128.8, 125.8, 125.6, 125.0, 122.2, 116.5, 110.7, 92.6, 41.8, 21.5, 14.2 ppm. HRMS (ESI): *m*/*z* calcd for C_19_H_17_N_3_O + H^+^: 304.1444 [M + H]^+^; found: 304.1446.

#### 3.5.3. Tetrazole Synthesis. (3S,4S)-2-Ethyl-4-(1H-tetrazol-5-Yl)-3-(p-tolyl)-3,4-dihydroisoquinolin-1(2H)-one (**21**)

Sodium azide (65 mg, 1 mmol) and ammonium chloride (53 mg, 1 mmol) were added in one portion to a stirred solution of compound **18c** (60 mg, 0.2 mmol) in dimethyl formamide-acetic acid (DMF-AcOH) mixture (10:1, 5.5 mL) in a screw-cap vial equipped with a magnetic stir bar. After heating for 24 h at 130 °C (metal heating block temperature), the reaction mixture was cooled to room temperature and partitioned between ethyl acetate (30 mL) and water (10 mL). The organic layer was washed with brine (3×), separated, dried over Na_2_SO_4_, filtered and concentrated to give crude product, which was purified by flash chromatography in DCM-MeOH to give the pure title compound as beige solid.

Yield 57 mg (85%, dr > 20:1). ^1^H NMR (500 MHz, CDCl_3_): δ = 7.75 (d, *J* = 7.8 Hz, 1H), 7.39 (td, *J* = 7.5, 1.3 Hz, 1H), 7.21 (td, *J* = 7.7, 1.2 Hz, 1H), 7.13 (dd, *J* = 7.6, 1.2 Hz, 1H), 7.06 (d, *J* = 8.1 Hz, 2H), 7.01 (d, *J* = 8.2 Hz, 2H), 5.05 (d, *J* = 1.6 Hz, 1H), 4.95 (d, *J* = 1.6 Hz, 1H), 3.93 (dq, *J* = 14.4, 7.2 Hz, 1H), 2.87 (dq, *J* = 14.3, 7.2 Hz, 1H), 2.26 (s, 3H), 0.84 ppm (t, *J* = 7.2 Hz, 3H). ^13^C NMR (126 MHz, CDCl_3_) δ 164.6, 157.2, 138.5, 134.4, 133.2, 132.4, 129.8, 129.4, 129.0, 129.0, 128.4, 126.3, 63.7, 42.3, 41.7, 40.9, 21.1, 12.6 ppm. HRMS (ESI): *m*/*z* calcd for C_19_H_19_N_5_O + H^+^: 334.1662 [M + H]^+^; found: 334.1661.

Crystal Data for C_19_H_19_N_5_O (*M* = 333.39 g/mol): monoclinic, space group P2_1_/c (no. 14), *a* = 9.37370(10) Å, *b* = 21.3889(2) Å, *c* = 8.67650(10) Å, *β* = 99.5170(10), *V* = 1715.64(3) Å^3^, *Z* = 4, *T* = 102.15 K, μ(CuKα) = 0.674 mm^−1^, *Dcalc* = 1.291 g/cm^3^, 27,122 reflections measured (8.268° ≤ 2Θ ≤ 160.55°), 3678 unique (*R*_int_ = 0.0312, R_sigma_ = 0.0177) which were used in all calculations. The final *R*_1_ was 0.0358 (I > 2σ(I)) and *wR*_2_ was 0.0942 (all data). Deposition Number 2203753 contains the supplementary crystallographic data for this paper. These data are provided free of charge by the joint Cambridge Crystallographic Data Centre (http://www.ccdc.cam.ac.uk/structures, accessed on 22 September 2022) and Fachinformationszentrum Karlsruhe Access Structures service.

### 3.6. 2-(2-Amino-2-oxoethyl)benzoic Acid (***22a***)

Prepared according to ref [30]. White powder. ^1^H NMR (400 MHz, DMSO-*d_6_*): δ = 12.98 (s, 1H), 7.81 (d, *J* = 7.7 Hz, 1H), 7.46 (t, *J* = 7.4 Hz, 1H), 7.36–7.28 (m, 3H), 6.86 (s, 1H), 3.81 ppm (s, 2H).^13^C NMR (101 MHz, DMSO-*d_6_*): δ = 172.8, 169.2, 137.4, 132.2, 132.0, 131.8, 130.5, 127.0, 40.9 ppm. HRMS (ESI): *m*/*z* calcd for C_9_H_9_NO_3_ + Na^+^: 202.0472 [M + Na]^+^; found: 202.0475.

### 3.7. Synthesis of Amides ***22b**–**d***

Corresponding amine (1.2 mmol) was added to a suspension of anhydride (1.2 mmol) in dry DCM (25 mL) and the mixture stirred at room temperature overnight. The solvent was removed under reduced pressure. The crude product was triturated with diethyl ether and hexane. The resulting powder was filtered off and air-dried.

#### 3.7.1. 2-(2-Oxo-2-(phenylamino)ethyl)benzoic Acid (**22b**)

White powder. Yield 263 mg (86%). ^1^H NMR (400 MHz, DMSO-*d_6_*):δ = 12.82 (s, 1H), 10.05 (s, 1H), 7.96–7.83 (m, 1H), 7.58 (d, *J* = 8.0 Hz, 2H), 7.52 (td, *J* = 7.5, 1.5 Hz, 1H), 7.38 (t, *J* = 8.1 Hz, 2H), 7.28 (t, *J* = 7.8 Hz, 2H), 7.02 (t, *J* = 7.4 Hz, 1H), 4.09 ppm (s, 2H).^13^C NMR (101 MHz, DMSO-*d_6_*): δ = 169.6, 169.0, 139.9, 137.3, 132.7, 132.1, 131.5, 130.7, 129.1, 127.3, 123.3, 119.4, 42.3 ppm. HRMS (ESI) *m*/*z*: calcd for C_15_H_13_NO_3_ + H^+^: 256.0968[M + H]^+^; found: 256.0971.

#### 3.7.2. 2-(2-(Ethyl(phenyl)amino)-2-oxoethyl)benzoic Acid (**22c**)

Yield 237 mg (68%). White powder. ^1^H NMR (400 MHz, CDCl_3_): δ = 13.15 (s, 1H), 7.94 (dd, *J* = 7.6, 1.6 Hz, 1H), 7.57 (dd, *J* = 8.3, 6.7 Hz, 2H), 7.52–7.46 (m, 1H), 7.42 (td, *J* = 7.5, 1.7 Hz, 1H), 7.39–7.30 (m, 3H), 6.98 (d, *J* = 7.4 Hz, 1H), 3.82 (q, *J* = 7.2 Hz, 2H), 3.69 (s, 2H), 1.18 ppm (t, *J* = 7.1 Hz, 3H). ^13^C NMR (101 MHz, CDCl_3_): δ = 171.9, 170.7, 141.6, 135.3, 132.1, 132.0, 131.6, 131.0, 130.1, 128.6, 128.4, 127.4, 44.9, 39.6, 12.6 ppm. HRMS(ESI): *m*/*z* calcd for C_17_H_17_NO_3_ + Na^+^:306.1101[M + Na]^+^; found: 306.1104.

#### 3.7.3. 2-((Ethyl(phenyl)amino)-2-oxoethyl)-5-nitrobenzoic Acid (**22d**)

Yellow powder. Yield 298 mg (76%).^1^H NMR (400 MHz, DMSO-*d*_6_): δ = 8.57 (d, *J* = 2.6 Hz, 1H), 8.30 (dd, *J* = 8.4, 2.6 Hz, 1H), 7.55–7.51 (m, 3H), 7.41 (d, *J* = 7.8 Hz, 3H), 3.65 (q, *J* = 7.2 Hz, 2H), 1.03 ppm (t, *J* = 7.1 Hz, 3H).^13^C NMR (101 MHz, DMSO-*d_6_*):δ = 168.8, 166.9, 146.6, 145.9, 142.4, 134.5, 132.5, 130.2, 129.0, 128.4, 126.2, 125.1, 43.8, 13.4ppm. HRMS(ESI): *m*/*z* calcd for C_17_H_16_N_2_O_5_ + Na^+^: 351.0951 [M + Na]^+^; found: 351.0956.

### 3.8. Synthesis of Tetrahydroisoquinolonecarboamides ***23a**,**b***

2-Amido benzoic acid **22a** or **22b** (0.25 mmol) was dissolved in PhCl (1.25 mL). CDI (49.0 mg, 1.2 eq) and *N*-ethyl-1-(p-tolyl)methanimine (37 mg, 0.25 mmol) were added, the vial was flushed with argon and heated at 130 °C overnight. The solvent was evaporated. The residue was purified by flash column chromatography on SiO_2_, using a gradient of acetone in hexane from 1:4 to 1:1. 

#### 3.8.1. (±)-(3S,4S)-2-Ethyl-1-oxo-3-(p-tolyl)-1,2,3,4-tetrahydroisoquinoline-4-carboxamide (**23a**)

White powder. Yield 23 mg (28%, dr > 20:1). ^1^H NMR (400 MHz, CDCl_3_): δ = 8.25 (dd, *J* = 7.1, 1.9 Hz, 1H), 7.47 (pd, *J* = 7.4, 1.6 Hz, 2H), 7.12 (dd, *J* = 6.9, 1.8 Hz, 1H), 7.04 (d, *J* = 8.1 Hz, 2H), 6.98 (d, *J* = 8.2 Hz, 2H), 5.71 (s, 1H), 5.48 (d, *J* = 1.5 Hz, 1H), 5.20 (s, 1H), 4.17 (dq, *J* = 14.3, 7.2 Hz, 1H), 3.79 (d, *J* = 1.6 Hz, 1H), 2.94 (dq, *J* = 14.2, 7.1 Hz, 1H), 2.26 (s, 3H), 1.21 ppm (t, *J* = 7.2 Hz, 3H). ^13^C NMR (101 MHz, CDCl_3_):δ = 172.4, 163.1, 137.5, 136.0, 132.8, 132.5, 129.8, 129.4, 129.1, 128.8, 128.6, 126.0, 6.12, 53.4, 41.6, 20.9, 12.8 ppm. HRMS (ESI): *m*/*z* calcd for C_19_H_20_N_2_O_2_ + H^+^:309.1598 [M + H]^+^; found: 309.1602.

#### 3.8.2. (±)-(3S,4S)-2-Ethyl-1-oxo-N-phenyl-3-(p-tolyl)-1,2,3,4-tetrahydroisoquinoline-4-carboxamide (**23b**)

Yellow amorphous solid. Yield 22 mg (23%, dr > 20:1).^1^H NMR (400 MHz, CDCl_3_): δ = 8.32 (dd, *J* = 7.2, 2.0 Hz, 1H), 7.54 (ddt, *J* = 11.8, 7.4, 3.6 Hz, 2H), 7.32–7.29 (m, *J* = 7.0 Hz, 4H), 7.22–7.18 (m, 1H), 7.12 (tt, *J* = 5.5, 2.4 Hz, 1H), 7.08–7.00 (m, 4H), 6.82 (s, 1H), 5.59 (d, *J* = 1.7 Hz, 1H), 4.21 (dq, *J* = 14.2, 7.2 Hz, 1H), 3.95 (d, *J* = 1.7 Hz, 1H), 2.94 (dq, *J* = 14.1, 7.1 Hz, 1H), 2.28 (s, 3H), 1.23–1.17 ppm (m, 3H). ^13^C NMR (101 MHz, CDCl_3_): δ = 168.3, 163.0, 137.5, 136.9, 136.0, 132.7, 132.2, 130.0, 129.5, 129.4, 129.3, 129.1, 129.0, 129.0, 126.1, 125.1, 120.2, 61.3, 54.6, 41.6, 21.0, 13.0 ppm. HRMS(ESI): *m*/*z* calcd for C_25_H_24_N_2_O_2_ + H^+^:385.1911[M + H]^+^; found: 385.1912.

### 3.9. Synthesis of Isochromenones ***24***

#### 3.9.1. 3-(Ethyl(phenyl)amino)-1H-isochromen-1-one (**24a**)

Compound **22c** (48 mg, 0.17 mmol) was dissolved in PhCl (1.25 mL). CDI (33.0 mg, 1.2 eq) and *N*-ethyl-1-(*p*-tolyl)methanimine (0.17 mmol) were added, the vial was flushed with argon and heated at 130 °C overnight. The solvent was evaporated. The residue was purified by flash column chromatography on SiO_2_, using a gradient of acetone in hexane from 1:6 to 1:2. 

Yellow oil. Yield 25 mg (34%) from 0.17 mmol.^1^H NMR (400 MHz, CDCl_3_): δ = 8.17–8.04 (m, 1H), 7.51–7.43 (m, 3H), 7.34–7.27 (m, 3H), 7.14 (ddd, *J* = 8.1, 7.0, 1.1 Hz, 1H), 7.05 (dt, *J* = 8.0, 0.8 Hz, 1H), 5.23 (s, 1H), 3.88 (q, *J* = 7.2 Hz, 2H), 1.28 ppm (t, *J* = 7.1 Hz, 3H). ^13^C NMR (101 MHz, CDCl_3_):δ = 161.9, 155.7, 142.9, 141.6, 134.7, 129.8, 129.7, 129.6, 128.3, 126.8, 126.4, 123.6, 123.5, 115.2, 81.7, 45.3, 13.8 ppm. HRMS (ESI): *m*/*z* calcd for C_17_H_15_NO_2_ + H^+^: 266.1176; found: 266.1175.

#### 3.9.2. 3-(Ethyl(phenyl)amino)-7-nitro-1H-isochromen-1-one (**24b**)

Compound **22d** (48 mg, 0.17 mmol) was dissolved in PhCl (1.25 mL). CDI (33.0 mg, 1.2 eq) and corresponding imine (0.17 mmol) were added, the vial was flushed with argon and heated at 60 °C overnight. The solvent was evaporated. The residue was purified by flash column chromatography on SiO_2_, using a gradient of acetone in hexane from 1:6 to 1:2. 

Orange amorphous powder. Yield 9 mg (17%). ^1^H NMR (400 MHz, CDCl_3_): δ = 8.95 (d, *J* = 2.5 Hz, 1H), 8.19 (dd, *J* = 8.9, 2.4 Hz, 1H), 7.54 (dd, *J* = 8.4, 7.0 Hz, 2H), 7.49–7.41 (m, 1H), 7.35–7.24 (m, 2H), 7.01 (d, *J* = 9.0 Hz, 1H), 5.11 (s, 1H), 3.93 (q, *J* = 7.1 Hz, 2H), 1.32 ppm (t, *J* = 7.1 Hz, 3H). ^13^C NMR (101 MHz, CDCl_3_): δ = 159.8, 158.6, 146.9, 142.3, 141.1, 130.3, 128.8, 128.3, 127.9, 126.9, 123.9, 112.9, 79.9, 46.0, 13.8 ppm. HRMS(ESI): *m*/*z* calcd for C_17_H_14_N_2_O_4_ + H^+^: 311.1032[M + H]^+^; found:311.1031.

### 3.10. Synthesis of 4-Chloro-5-nitro-2-(tosylmethyl)benzoic Acid (***26***)

Prepared according to modified procedure from [17]. First, 3-nitro-4-chloro benzoic acid (1.0 g, 5 mmol) was added to a thoroughly stirred suspension of *t*-BuOK (2.8 g, 25 mmol) in DMSO (10 mL), while the temperature was kept at 18 °C. After 5 min, 1-((chloromethyl)sulfonyl)-4-methylbenzene (1.0 g, 5 mmol) was added. The mixture was stirred at 18 °C for 50 min, poured over ice (50 g) mixed with 10% aq. HCl (100 mL), extracted with EtOAc (3 × 50 mL). The combined organic extracts were washed with sat. aq. K_2_CO_3_ (2 × 50 mL), the resulting aqueous layer was separated and extracted with DCM (3 × 25 mL), acidified with HCl conc. to pH = 2 and extracted again with EtOAc (3 × 50 mL), washed with brine (20 mL), dried over Na_2_SO_4_. The solvent was removed under reduced pressure and the crude product was crystallized from EtOAc/hexane.

Yellow powder. Yield 0.58 g (32%). ^1^H NMR (400 MHz, DMSO-*d_6_*): δ = 8.47 (s, 1H), 7.61 (s, 1H), 7.53(d, *J* = 8.3 Hz, 2H), 7.43 (d, *J* = 8.1 Hz, 2H), 5.29 (s, 2H), 2.41 ppm (s, 3H). ^13^C NMR (101 MHz, DMSO-*d_6_*): δ = 166.0, 147.1, 145.5, 136.7, 135.6, 135.4, 132.4, 130.3, 128.6, 128.3, 128.2, 57.5, 21.6 ppm. HRMS(ESI): *m*/*z* calcd for C_15_H_12_ClNO_6_S + Na^+^: 391.9966 [M + Na]^+^; found: 391.9965.

### 3.11. Synthesis of 3-Aryl Sulphonyl Tetrahydroisoquinolones ***29*** and ***30***

2-(Arylsulfonyl)methyl)benzoic acid (0.1 or 0.25 mmol) was dissolved in PhCl (1.25 mL). CDI (1.2 equiv.) and *N*-ethyl-1-(*p*-tolyl)methanimine (1 equiv.) were added, the vial was flushed with argon and heated at 130 °C overnight. The solvent was evaporated. The residue was purified by flash column chromatography on SiO_2_, using a gradient of acetone in hexane from 1:5 to 1:2. 

#### 3.11.1. (±)-(3R,4S)-2-Ethyl-4-(phenylsulfonyl)-3-(p-tolyl)-3,4-dihydroisoquinolin-1(2H)-one (**29**) 

White powder. Yield 26 mg (26%, dr > 20:1) from 0.25 mmol of compound **25**. ^1^H NMR (400 MHz, CDCl_3_):δ = 8.05 (dd, *J* = 7.8, 1.4 Hz, 1H), 7.59 (tt, *J* = 6.9, 1.8 Hz, 1H), 7.48 (td, *J* = 7.6, 1.3 Hz, 1H), 7.43–7.34 (m, 5H), 7.07–7.01 (m, 3H), 6.97–6.91 (m, 2H), 5.60 (s, 1H), 4.38 (d, *J* = 1.1 Hz, 1H), 3.72 (dq, *J* = 14.3, 7.2 Hz, 1H), 3.17 (dq, *J* = 14.4, 7.3 Hz, 1H), 2.27 (s, 3H), 1.17 ppm (t, *J* = 7.2 Hz, 3H).^13^C NMR (101 MHz, CDCl_3_): δ = 161.9, 138.3, 135.7, 135.3, 134.3, 131.7, 130.5, 130.4, 129.9, 129.7, 129.2, 128.7, 128.1, 126.9, 126.1, 71.3, 59.1, 42.4, 20.9, 12.9 ppm. HRMS(ESI): *m*/*z* calcd for C_24_H_23_NO_3_S + H^+^: 406.1477 [M + H]^+^; found: 406.1476.

#### 3.11.2. (±)-(3R,4S)-6-Chloro-2-ethyl-7-nitro-3-(p-tolyl)-4-tosyl-3,4-dihydroisoquinolin-1(2H)-one (**30**)

White amorphous solid. Yield 21 mg (42%, dr > 20:1) from 0.1 mmol of compound **26**. ^1^H NMR (400 MHz, CDCl_3_):δ = 8.56 (s, 1H), 7.45–7.38 (m, 2H), 7.30 (d, *J* = 8.1 Hz, 2H), 7.10 (d, *J* = 7.9 Hz, 2H), 7.04 (s, 1H), 6.93–6.88 (m, 2H), 5.61 (s, 1H), 4.32 (d, *J* = 1.2 Hz, 1H), 3.80 (dq, *J* = 14.3, 7.2 Hz, 1H), 3.18 (dq, *J* = 14.3, 7.2 Hz, 1H), 2.47 (s, 3H), 2.30 (s, 3H), 1.21 ppm (t, *J* = 7.2 Hz, 3H). ^13^C NMR (126 MHz, CDCl_3_):δ = 159.3, 148.7, 146.5, 139.0, 134.3, 133.5, 132.3, 132.1, 130.3, 130.1, 130.0, 129.8, 129.2, 125.8, 125.2, 70.1, 58.7, 42.7, 21.8, 21.0, 12.8 ppm. HRMS(ESI): *m*/*z* calcd forC_25_H_23_ClN_2_O_5_S + Na^+^: 521.908 [M + Na]^+^; found: 521.0912.

## 4. Conclusions

Encouraged by our previous success involving homophthalic acid monoesters in the CCR-like cyclocondensation with imines, we tested a number of other *o*-methyl benzoic acids bearing various electron-withdrawing groups in the α-position. The majority of these substrates, being activated either by CDI or acetic anhydride, did deliver the expected tetrahydroisoquinolone adducts. The homophthalic acid mononitriles displayed a high promise as substrates for the new reaction, offering the largest scope, generally higher yields and modest to high diastereoselectivity. Homophthalic acid monoamides, such as primary amide or anilide, gave the Castagnoli–Cushman adducts, albeit in low yields; tertiary anilides failed to condense with the imine component. Sulfonyl-substituted substrates also gave the desired (and hitherto unknown) type of tetrahydroisoquinolines. Despite the low yields, this approach to sulfonyl-substituted tetrahydroisoquinolines is deemed practical as an alternative synthesis based on the traditional, carboxylic acid CCR adducts appears cumbersome and multistep. An azido- and nitro-substituted *o*-methyl benzoic acids failed to react with imines. Collectively, these results clearly expand the scope of the new type of Castagnoli–Cushman reaction, as well as set certain apparent limitations.

## Data Availability

Data available from the corresponding authors upon reasonable request.

## Supplementary Materials

The following supporting information can be downloaded at: https://www.mdpi.com/article/10.3390/molecules27217211/s1; Figure S1: ORTEP representation of compound **18b** drawn at 50% probability level; Figure S2: ORTEP representation of compound **21** drawn at 50% probability level; Table S1: Optimization studies for synthesis of compound 18k; Table S2: Crystal data and structure refinement for **18b**; Table S3: Selected ^1^H NMR data for compounds 18 used for relative configuration assignment. Refs. [31, 32, 33] are cited in Supplementary Materials.

## Sample Availability

Samples of the compounds are available from the authors.
