# Supplementary material for: Extending the Scope of the New Variant of the Castagnoli–Cushman Cyclocondensation onto o-Methyl Benzoic Acids Bearing Various Electron-Withdrawing Groups in the α-Position
Source: Molecules. 2022 Oct 25;27(21):7211. doi: 10.3390/molecules27217211 (PMC9659198; doi:10.3390/molecules27217211)

ELECTRONIC SUPPORTING INFORMATION FOR

Extending the scope of the new variant of the Castagnoli-Cushman cyclocondensation onto *o*-methyl benzoic acids bearing various electron-withdrawing groups in the α-position

Natalia Guranova[a], Lyudmila Yakovleva[a], Olga Bakulina[a], Dmitry Dar'in[a] and Mikhail Krasavin*[,a,b]

* Corresponding author.

[a]Saint Petersburg State University, 26 Universitetskii prospect, Peterhof 198504 Russian Federation

[b]Immanuel Kant Baltic Federal University, Kaliningrad 236041 Russian Federation

E-mail: m.krasavin@spbu.ru, URL: http://krasavin-group.org

Table of contents

Table S1	S2
Crystallographic data, Table S2	S3
Table S3	S4
References	S4
Copies of NMR spectra	S5

Table S1. Optimization studies for synthesis of compound 18k

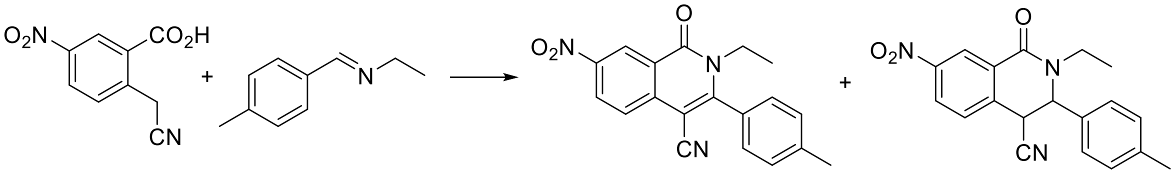

19a 18k

Activator	Equivalents	Temperature	Duration	Molarity (Solvent quantity)	Scale, mmol	Yield 19a, %	Yield 18k, %	Entry
**CDI**	1.2	30°C	overnight	0.2M (0.15/0.75 or 0.25/1.25)	0.15	**38**	-	1
		60°C				24	9	2
		110°C				**42**	-	3
		130 °C			0.25	**49**	-	4
		60°C	2h	0.2M	0.25	**42**	-	5
				0.05M (5mL)		24	20	6
				0.025M (10 mL)		30	-	7
	0.6	60°C	2h	0.2M		19		8
	1.2	150°C	overnight			**44**	-	9
**Ac_2O**	1.2	20°C	overnight	0.2M	0.25	-	39	10
		40°C				-	**44**	11
		60°C	2h			-	31	12
	5.0	60°C				-	**46**	13
	1.2	60°C		0.05M (5 mL)		-	30	14
		60°C		0.5M (0.5 mL)		-	**46**	15
		110°C	overnight	0.2M		-	27	16
		150°C				-	-	17
**HATU**	1.2	60°C	2h	0.2M	0.25	-	21	18
**T3P**	1.2	60°C	2h	0.2M	0.25	-	25	19

Optimization on solvents

Solvent	Ac2O	CDI
Dioxane-1,4	18k (44%)	19a (33%), 18k (12%)
MeCN	18k (20%)	19a (31%), 18k (12%)

Crystallographic data

Single crystal X-ray data were obtained using Rigaku XtaLAB Synergy diffractometer. The crystals were kept at 100 K during data collection. Using Olex2 [1], the structure was solved with the SHELXT [2] structure solution program using Intrinsic Phasing and refined with the SHELXL [3] refinement package using Least Squares minimisation.

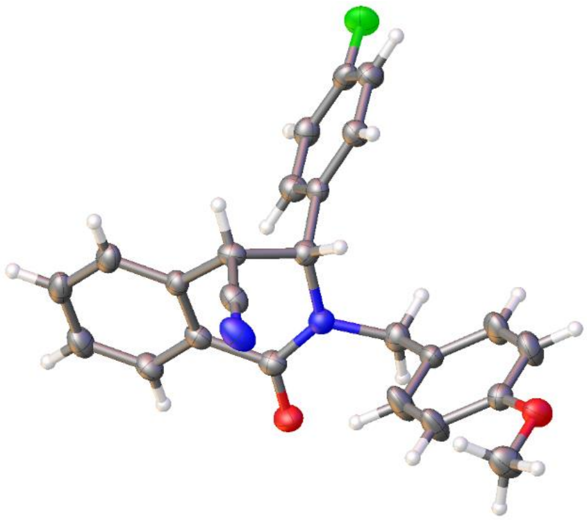

Figure S1. ORTEP representation of compound **18b** drawn at 50% probability level

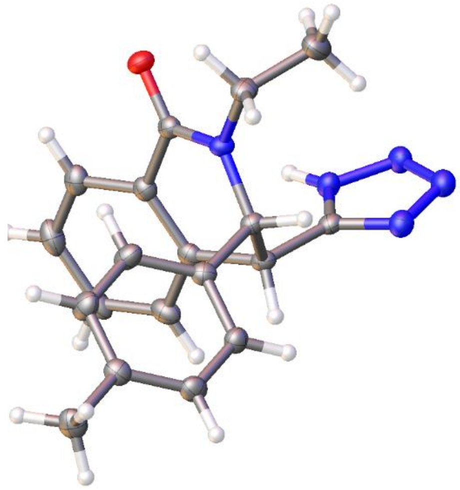

Figure S2. ORTEP representation of compound **21** drawn at 50% probability level

Table S2. Crystal data and structure refinement for **18b**

Identification code	**18b**	**21**
Empirical formula	$C_{24}H_{19}FN_2O_2$	$C_{19}H_{19}N_5O$
Formula weight	386.41	333.39
Temperature/K	100.15	102.15
Crystal system	monoclinic	monoclinic
Space group	$P2_1/n$	$P2_1/c$
a/Å	8.51030(10)	9.37370(10)
b/Å	8.6727(2)	21.3889(2)
c/Å	26.2561(5)	8.67650(10)
α/°	90	90
β/°	94.418(2)	99.5170(10)
γ/°	90	90
Volume/Å^3	1932.13(6)	1715.64(3)
Z	4	4
ρ_{calc}g/cm^3	1.328	1.291
μ/mm^{-1}	0.750	0.674
F(000)	808.0	704.0
Crystal size/mm^3	0.1 × 0.08 × 0.04	0.22 × 0.2 × 0.12
Radiation	CuKα (λ = 1.54184)	CuKα (λ = 1.54184)
2Θ range for data collection/°	6.754 to 160.042	8.268 to 160.55
Index ranges	$-10 \le h \le 8, -11 \le k \le 10, -33 \le l \le 33$	$-11 \le h \le 11, -27 \le k \le 26, -11 \le l \le 9$
Reflections collected	28288	27122
Independent reflections	4123 [R_{int} = 0.0501, R_{sigma} = 0.0296]	3678 [R_{int} = 0.0312, R_{sigma} = 0.0177]

Data/restraints/parameters	4123/0/264	3678/0/232
Goodness-of-fit on F^2	1.101	1.069
Final R indexes [I>=2σ (I)]	R_1 = 0.1293, wR_2 = 0.3415	R_1 = 0.0358, wR_2 = 0.0928
Final R indexes [all data]	R_1 = 0.1330, wR_2 = 0.3425	R_1 = 0.0374, wR_2 = 0.0942
Largest diff. peak/hole / e $Å^{-3}$	0.77/-0.53	0.28/-0.23
CCDC	2192610	2203753

Table S3. Selected ^{1}H NMR data for compounds 18 used for relative configuration assignment

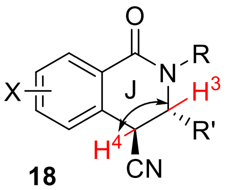

18

Entry	Compound	H^3 signal (isomer 1[a]): δ ppm/J Hz	H^3 signal (isomer 2): δ ppm, J Hz
1	**18a**	5.04/2.5	-
2	**18b**	4.95/2.6 (*trans*- according to X-ray)	4.83/5.9
3	**18c**	5.04/2.7	4.93/5.8
4	**18d**	5.04/2.4	4.91/5.8
5	**18e**	5.19/2.0	5.06/5.7
6	**18f**	4.97/2.5	4.85/6.0
7	**18g**	4.93/2.5	-
8	**18h**	5.12/3.2	5.05/5.9
9	**18i**	5.46/1.7	4.98/6.5
10	**18j**	5.37/2.3	5.23/5.6
11	**18k**	5.16/2.7	5.05/5.8
12	**18l**	5.16/2.5	5.03/5.8
13	**18m**	5.04/2.6	4.94/5.9
14	**18n**	5.50/2.4	5.36/5.7
15	**18o**	5.30/2.4	5.17/5.8
16	**18p**	5.94/2.4	6.12/6.3
17	**18q**	4.39[b]/2.3	-
18	**23a**	5.48/1.5	-
19	**23b**	5.59/1.6	-
20	**29**	5.60<1	-
21	**30**	5.50<1	-

[a] Major isomer for all isolated compounds except for 18b and 18q. [b]H^4 signal

References

1. Dolomanov, O.V., Bourhis, L.J., Gildea, R.J, Howard, J.A.K. & Puschmann, H. (2009), J. Appl. Cryst. 42, 339-341.
2. Sheldrick, G.M. (2015). Acta Cryst. A71, 3-8.
3. Sheldrick, G.M. (2015). Acta Cryst. C71, 3-8.

^{1}H NMR spectrum of compound 12a

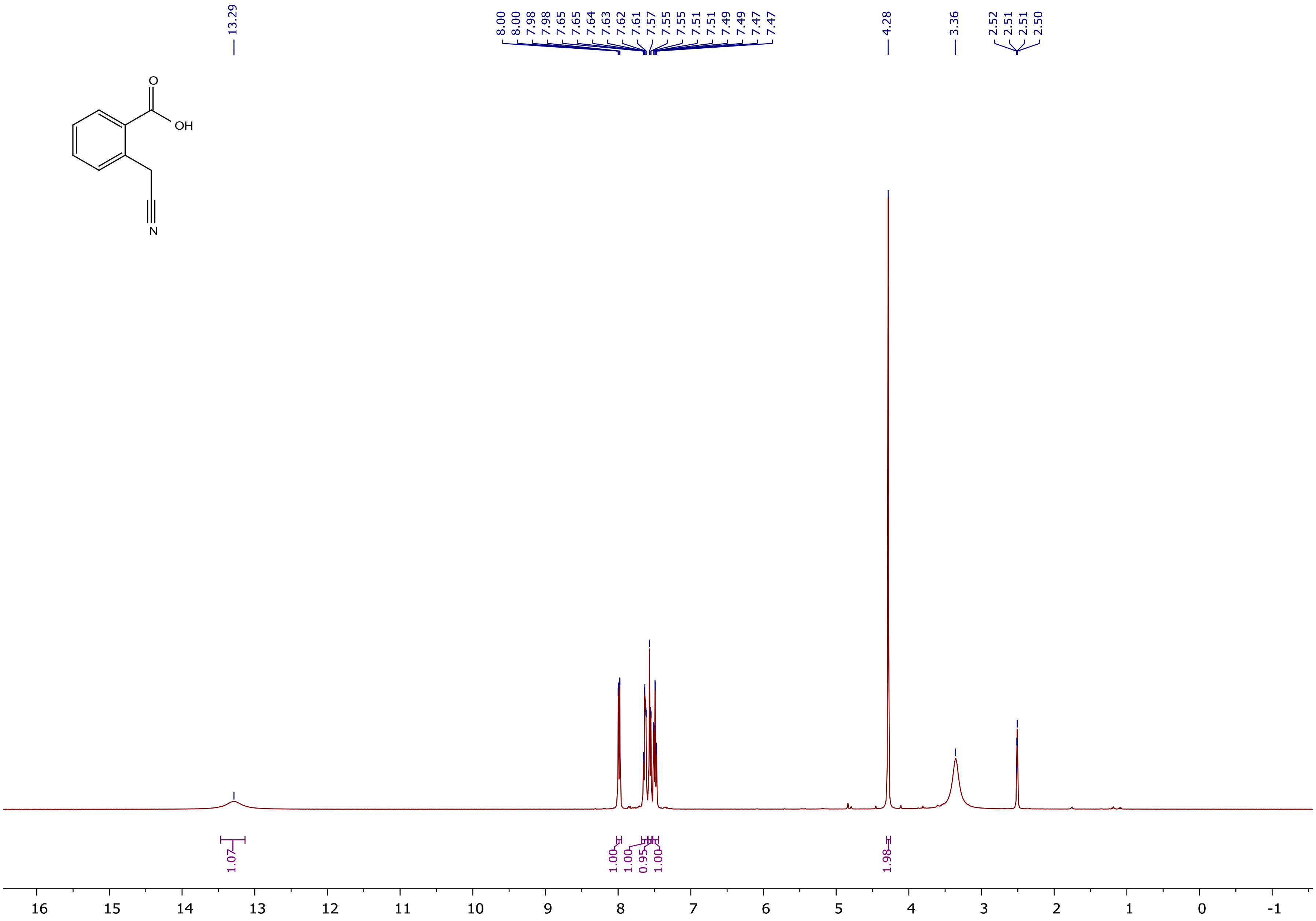

^{13}C NMR spectrum of compound 12a

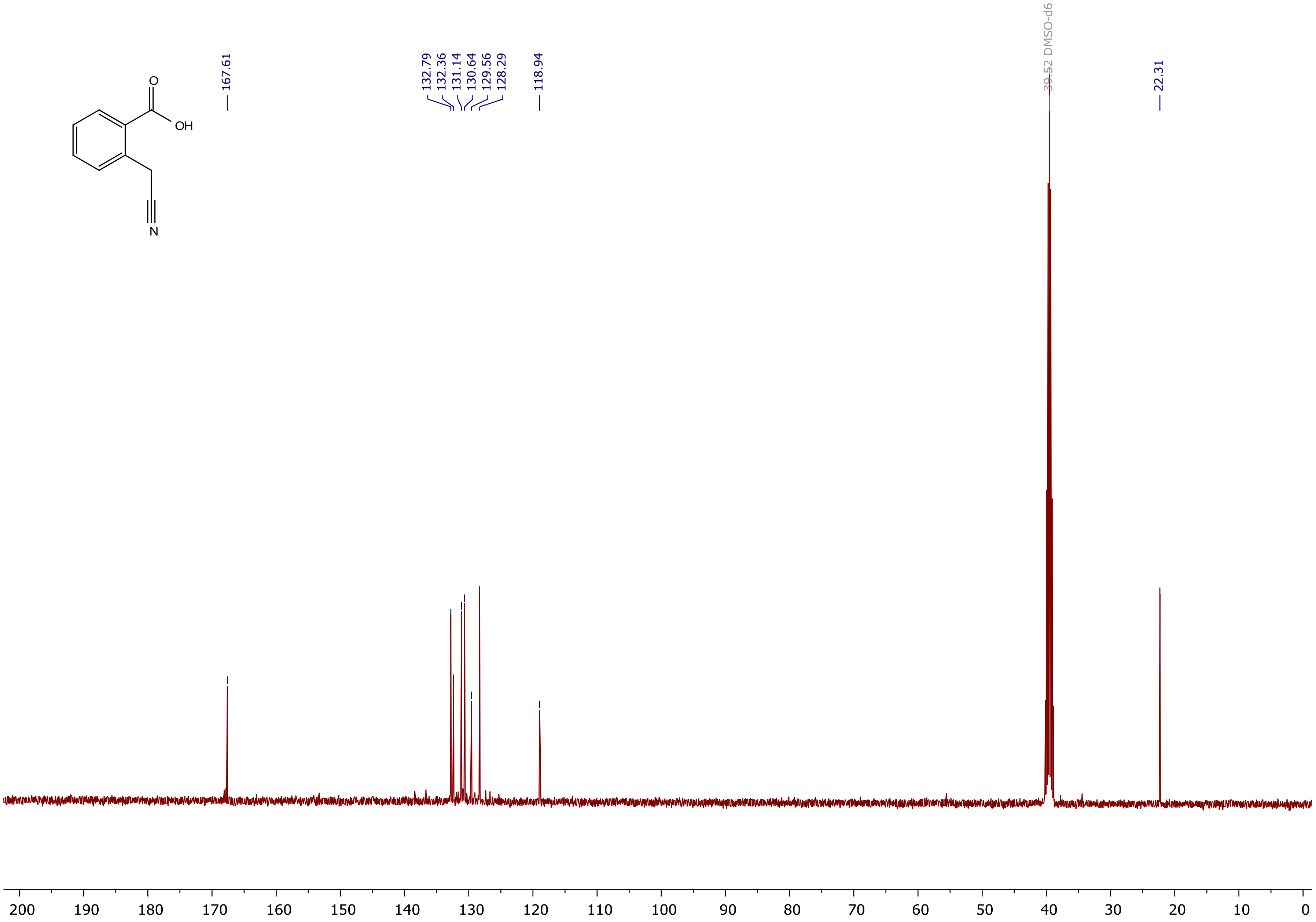

^{1}H NMR spectrum of compound 12b

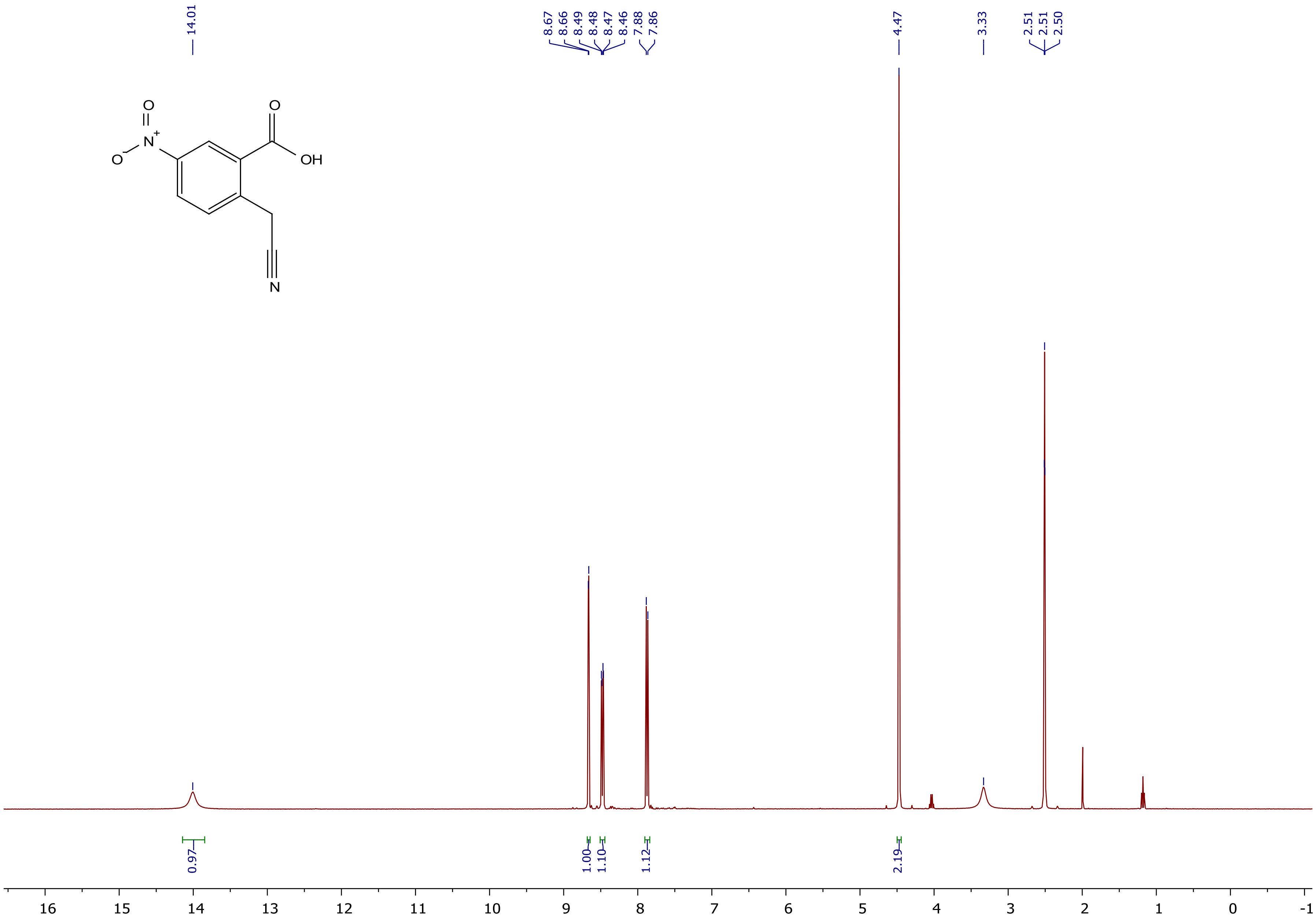

^{13}C NMR spectrum of compound 12b

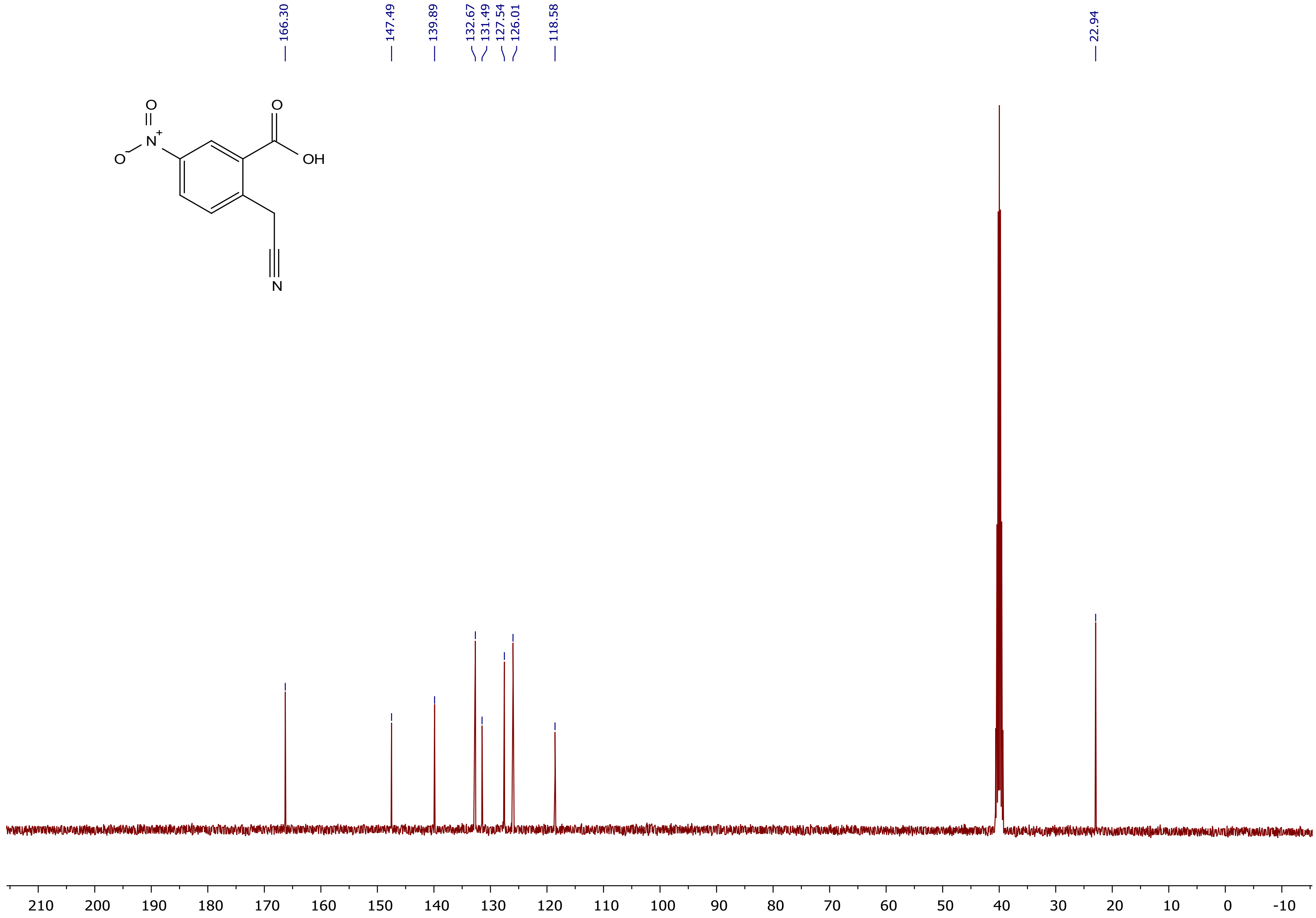

^{1}H NMR spectrum of compound 12c

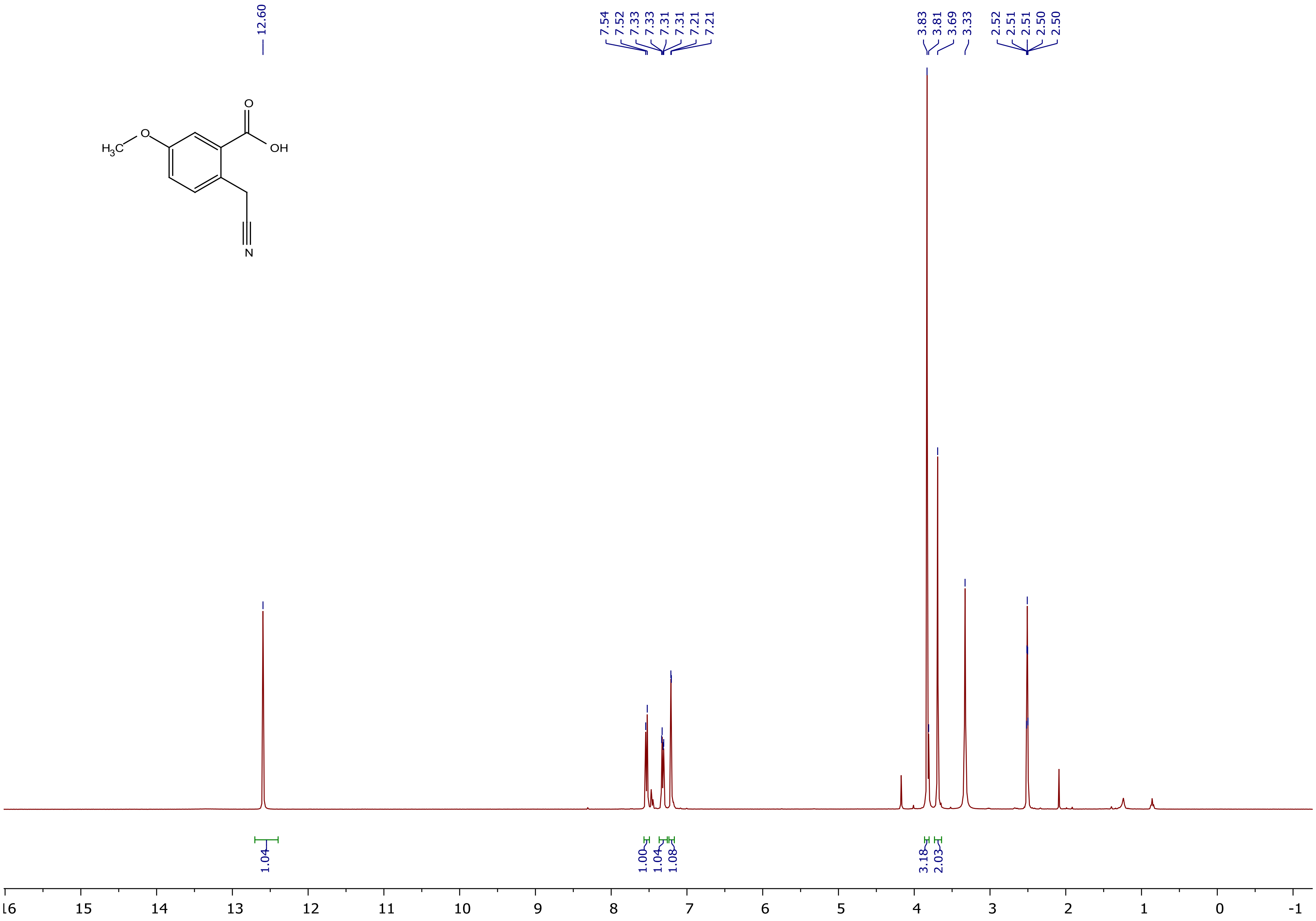

^{13}C NMR spectrum of compound 12c

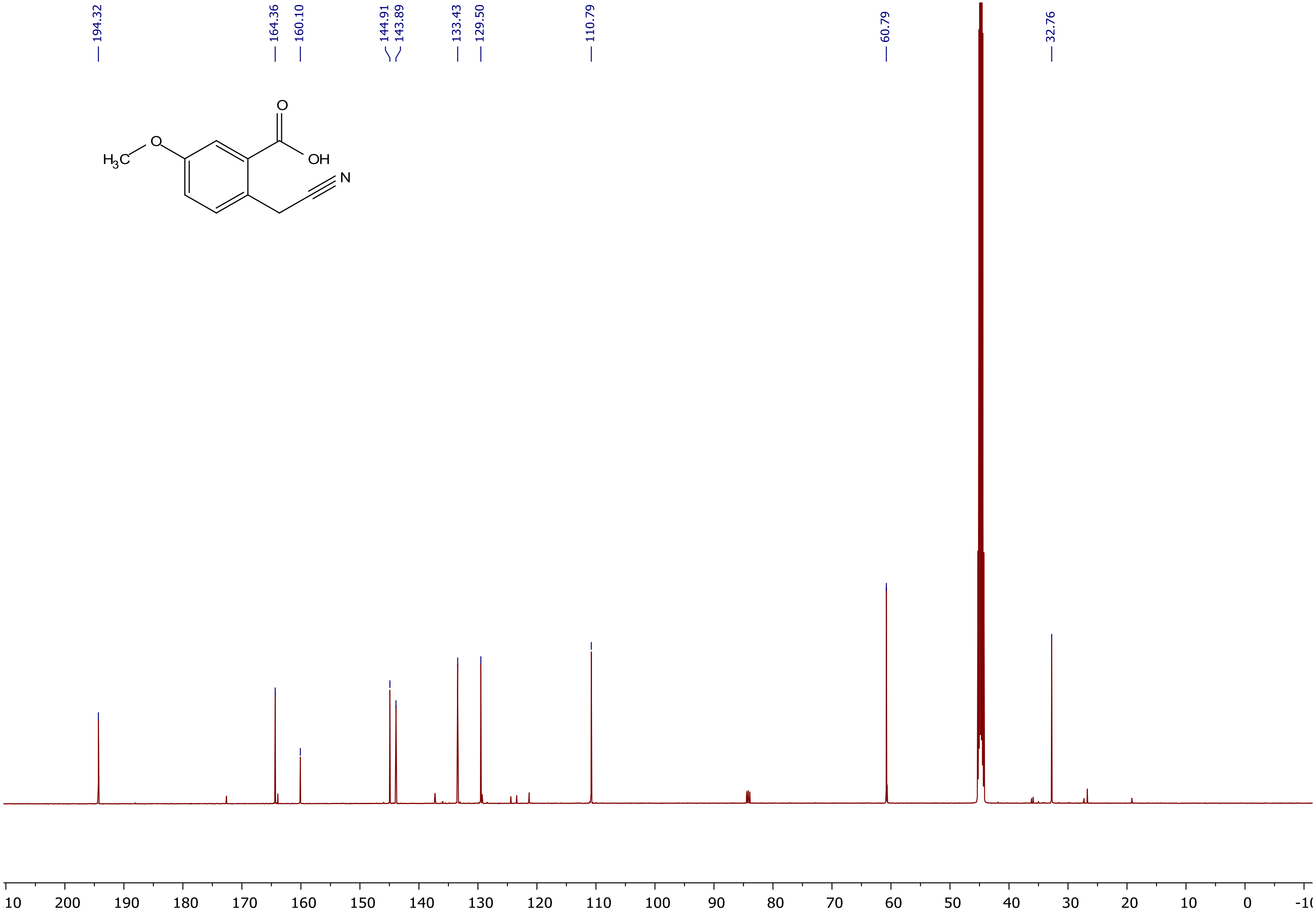

^{1}H NMR spectrum of compound 18a

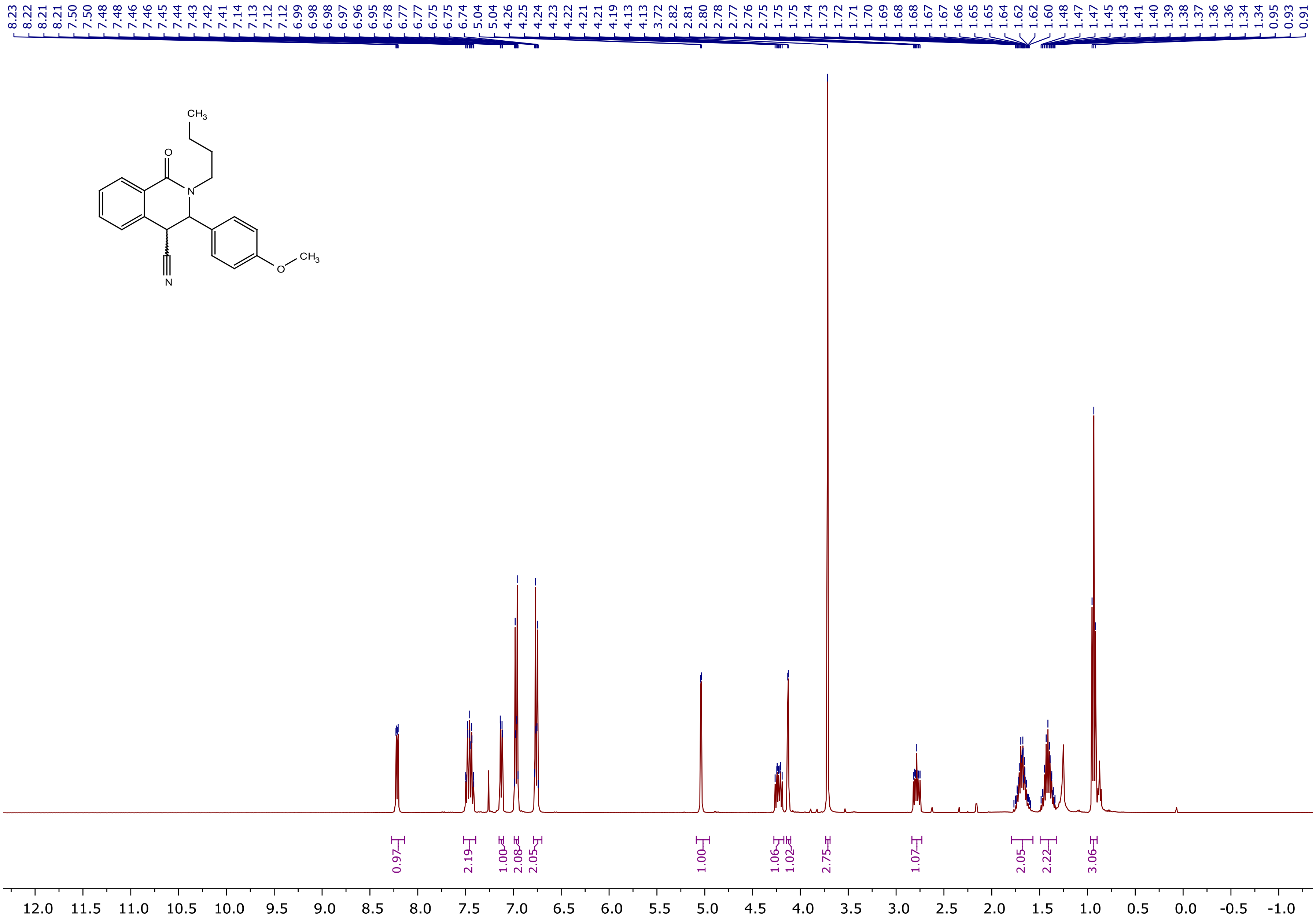

^{13}C NMR spectrum of compound 18a

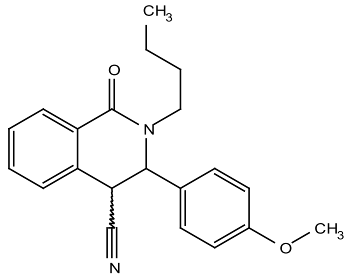

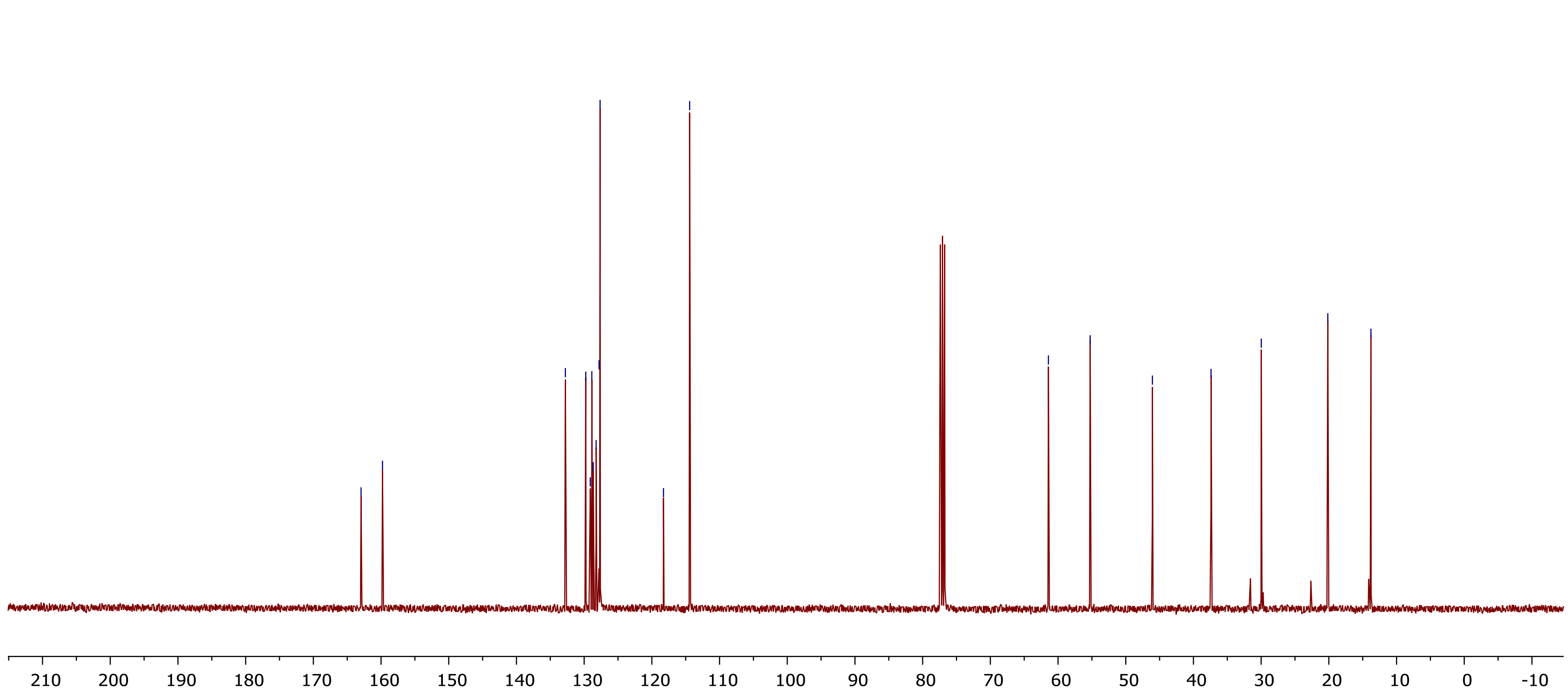

^{1}H NMR spectrum of compound 18b, trans-isomer

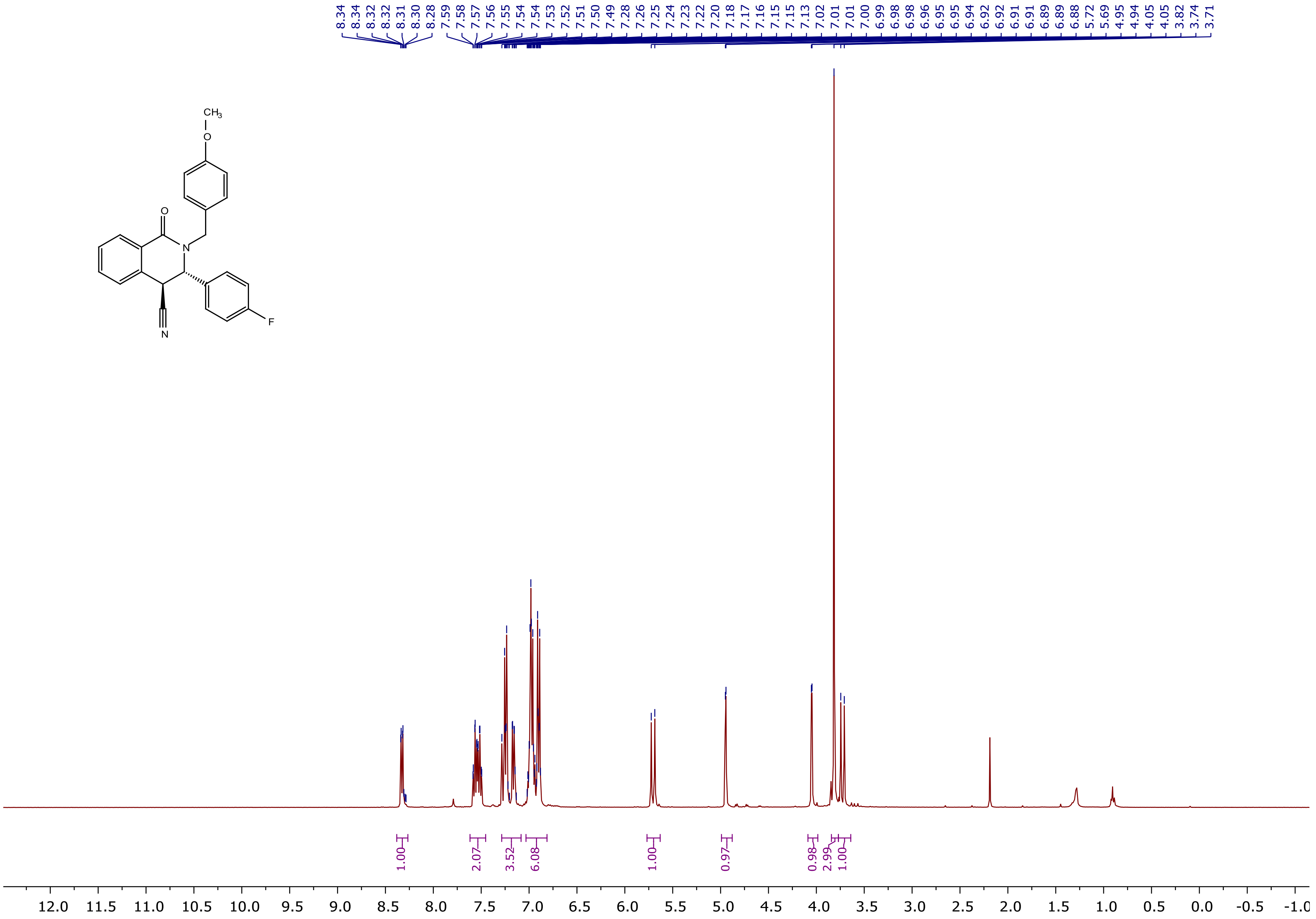

^{13}C NMR spectrum of compound 18b, trans-isomer

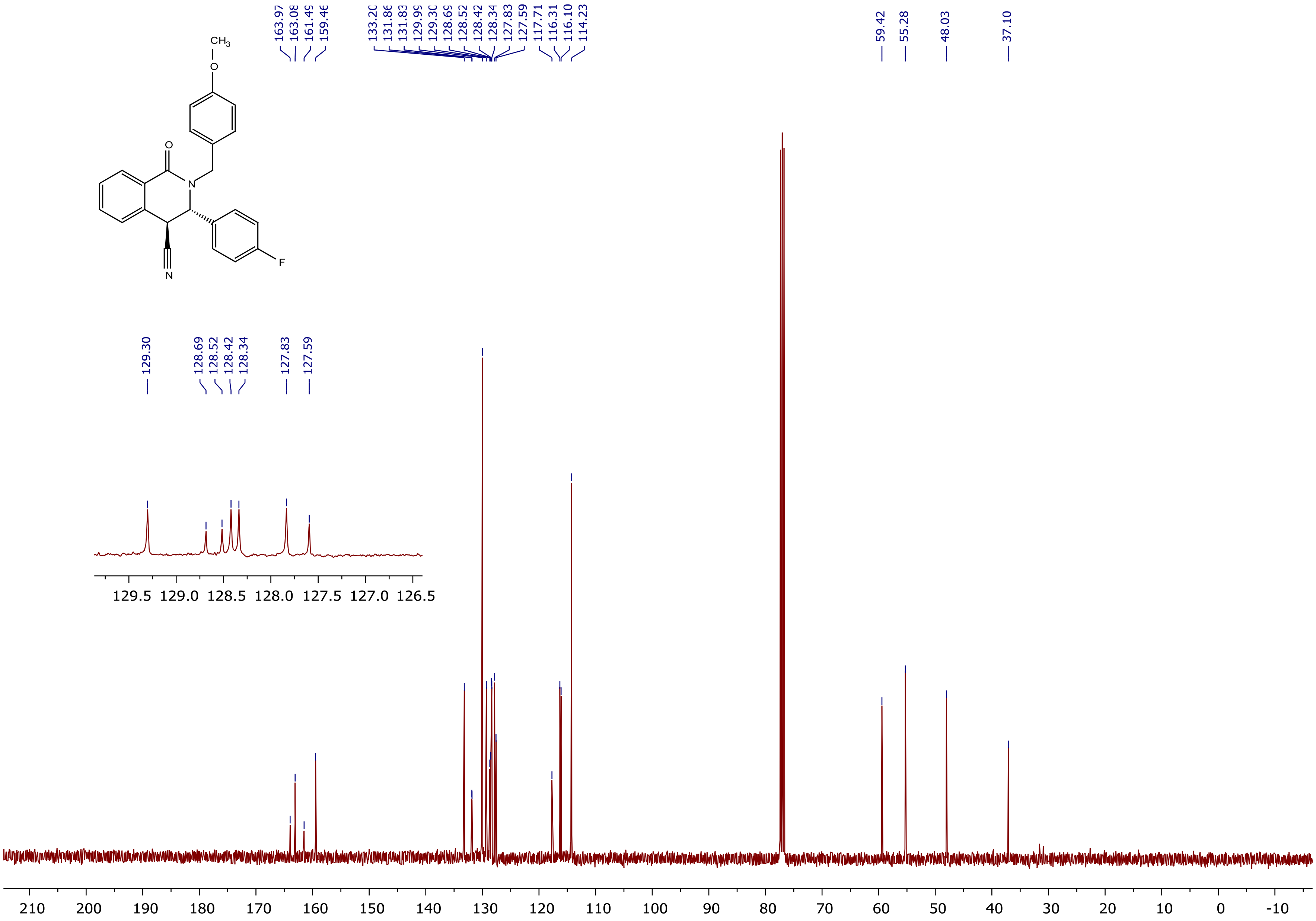

^{19}F NMR spectrum of compound 18b, trans-isomer

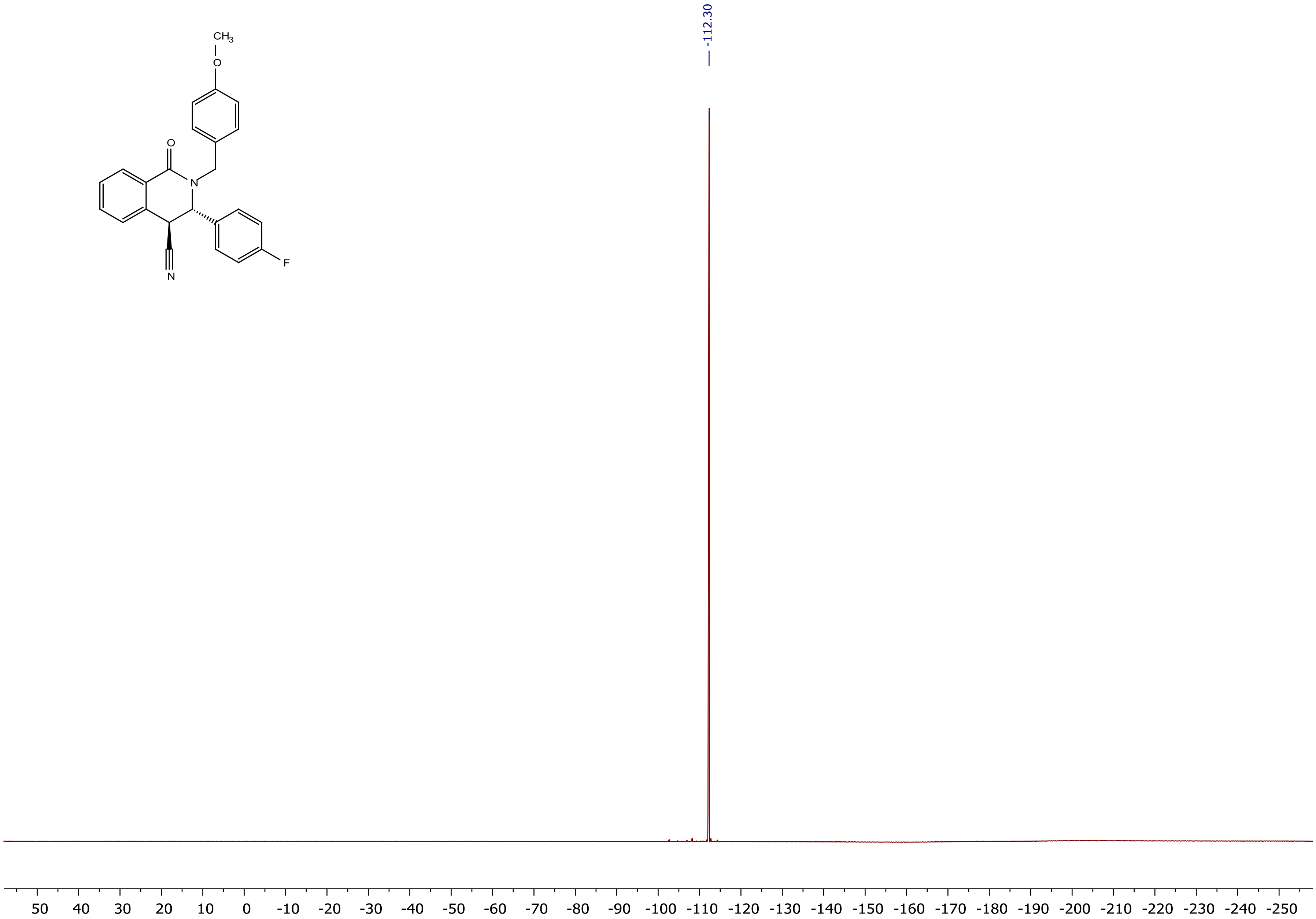

^{1}H NMR spectrum of compound compound 18b, cis-isomer

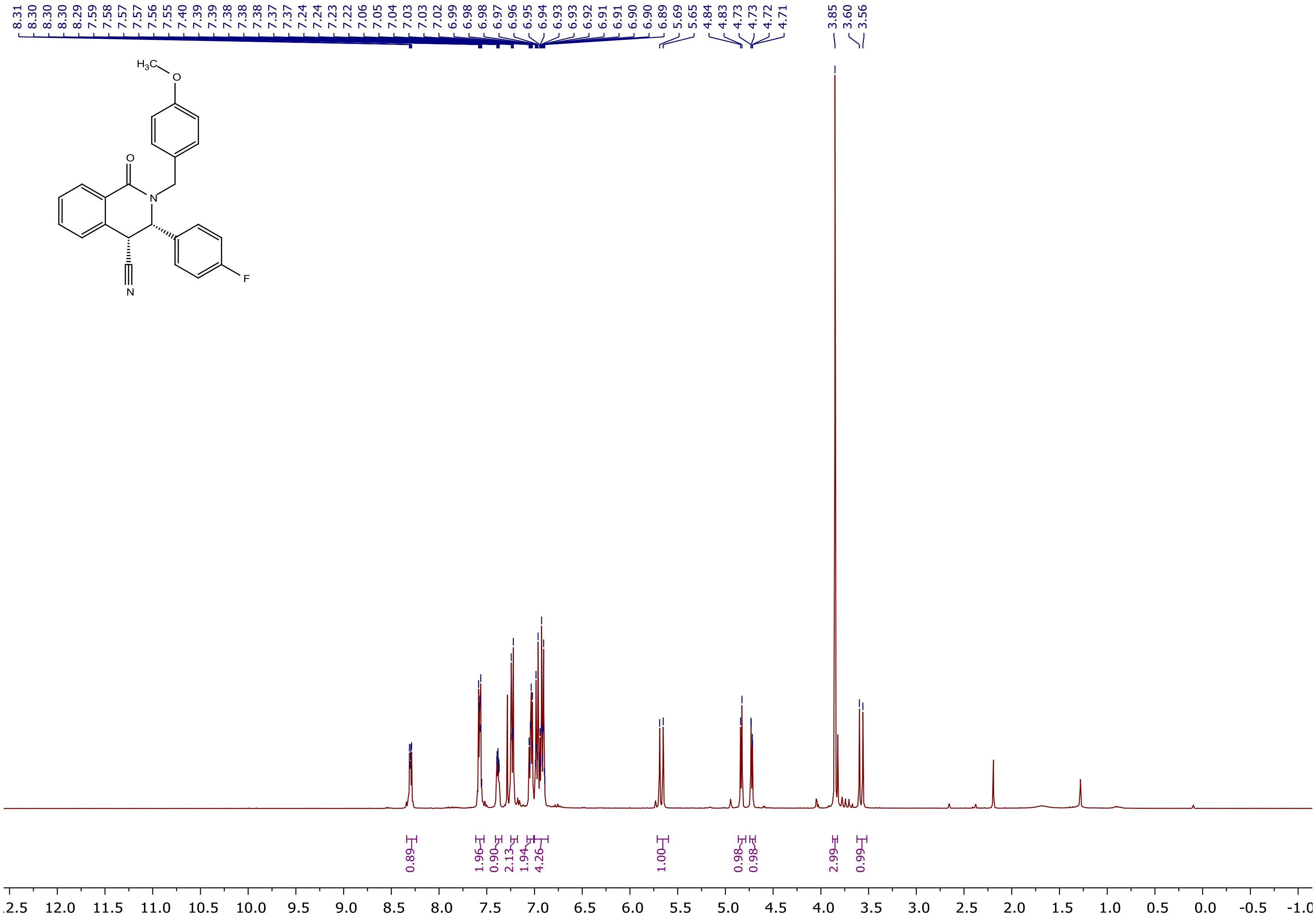

^{13}C NMR spectrum of compound 18b, cis-isomer

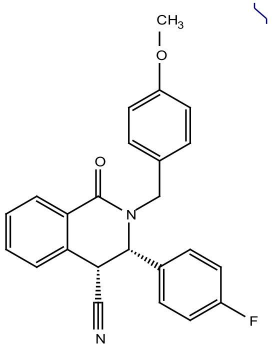

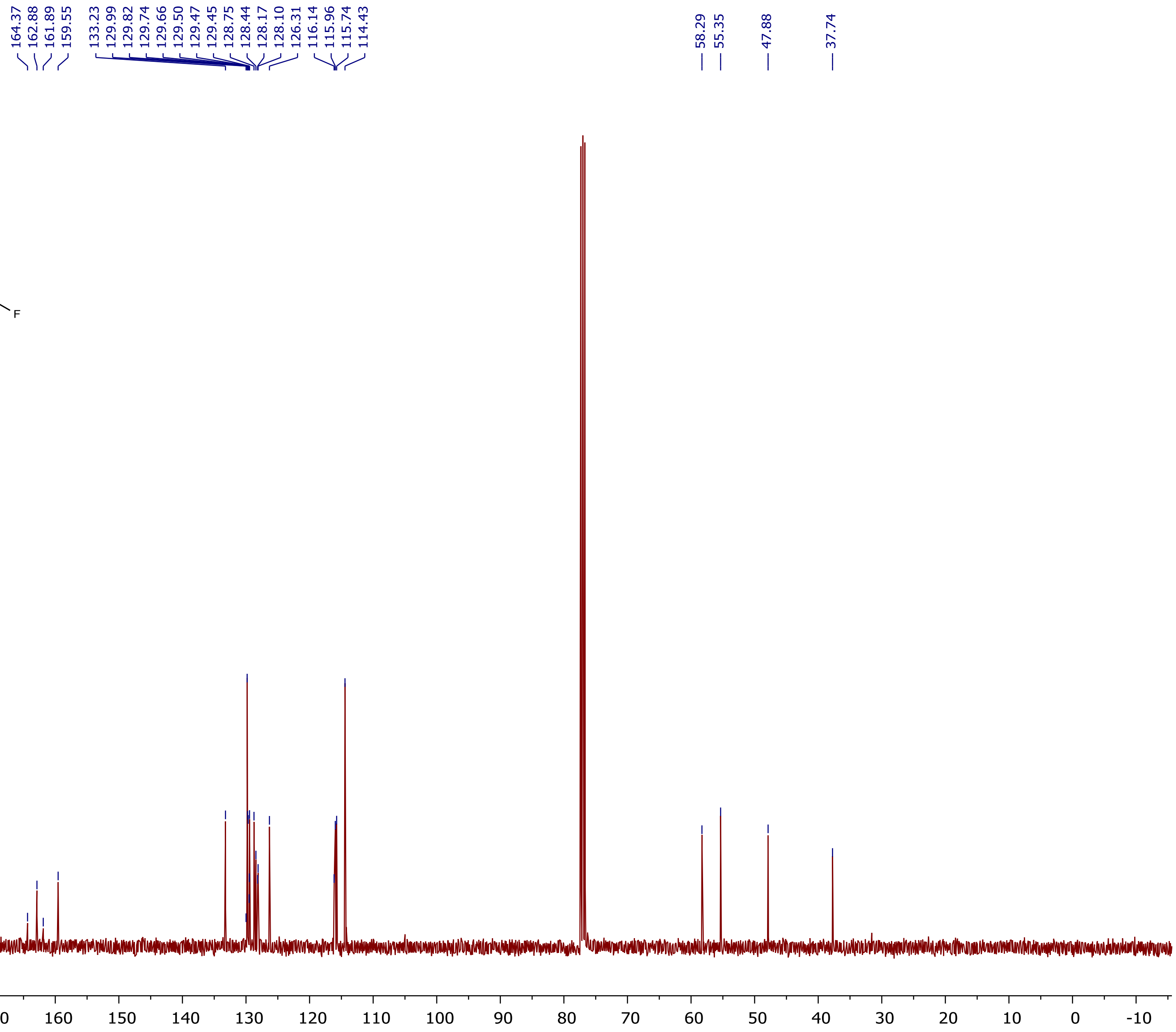

^{19}F NMR spectrum of compound 18b, cis-isomer

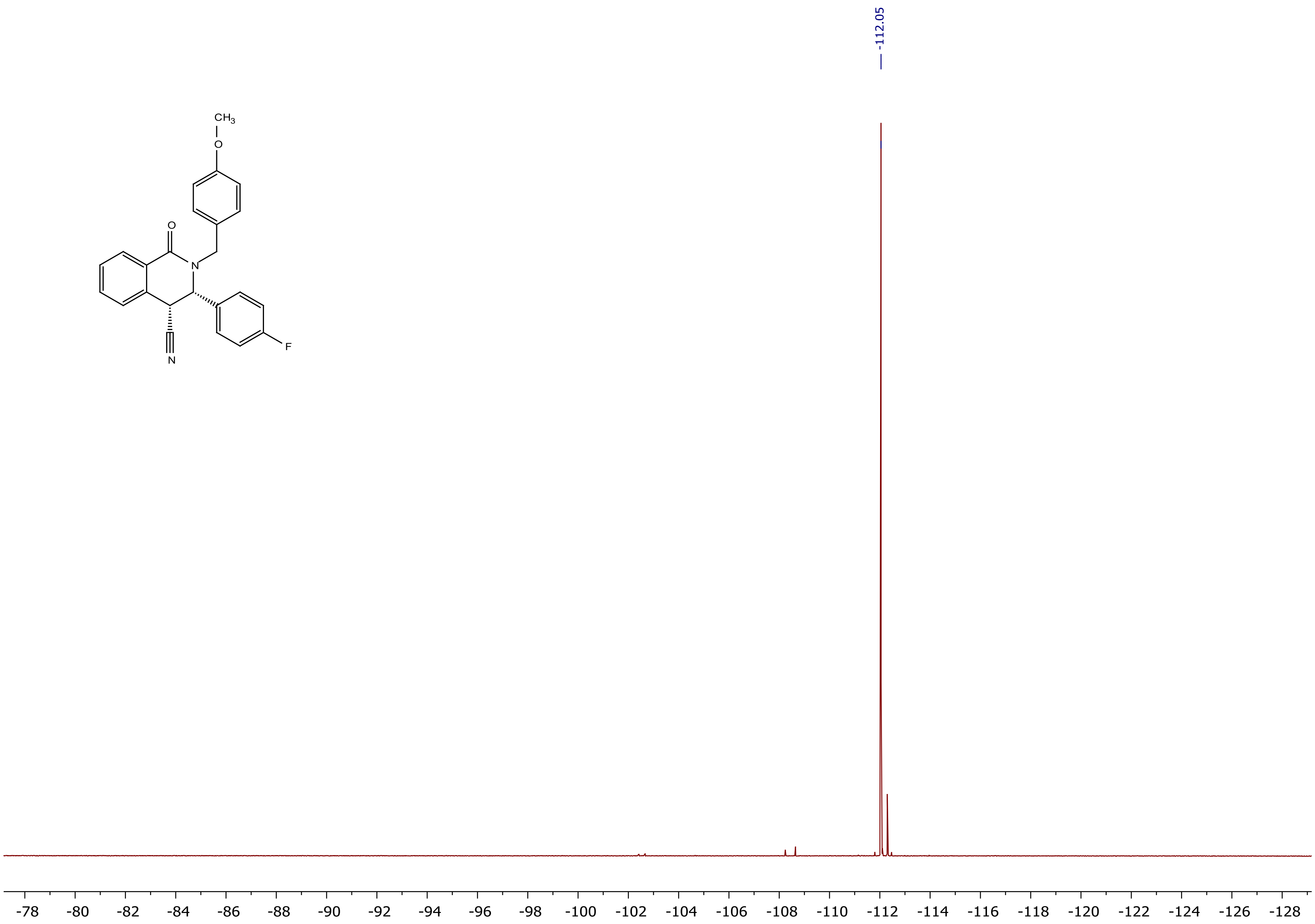

^{1}H NMR spectrum of compound 18c

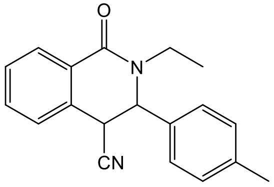

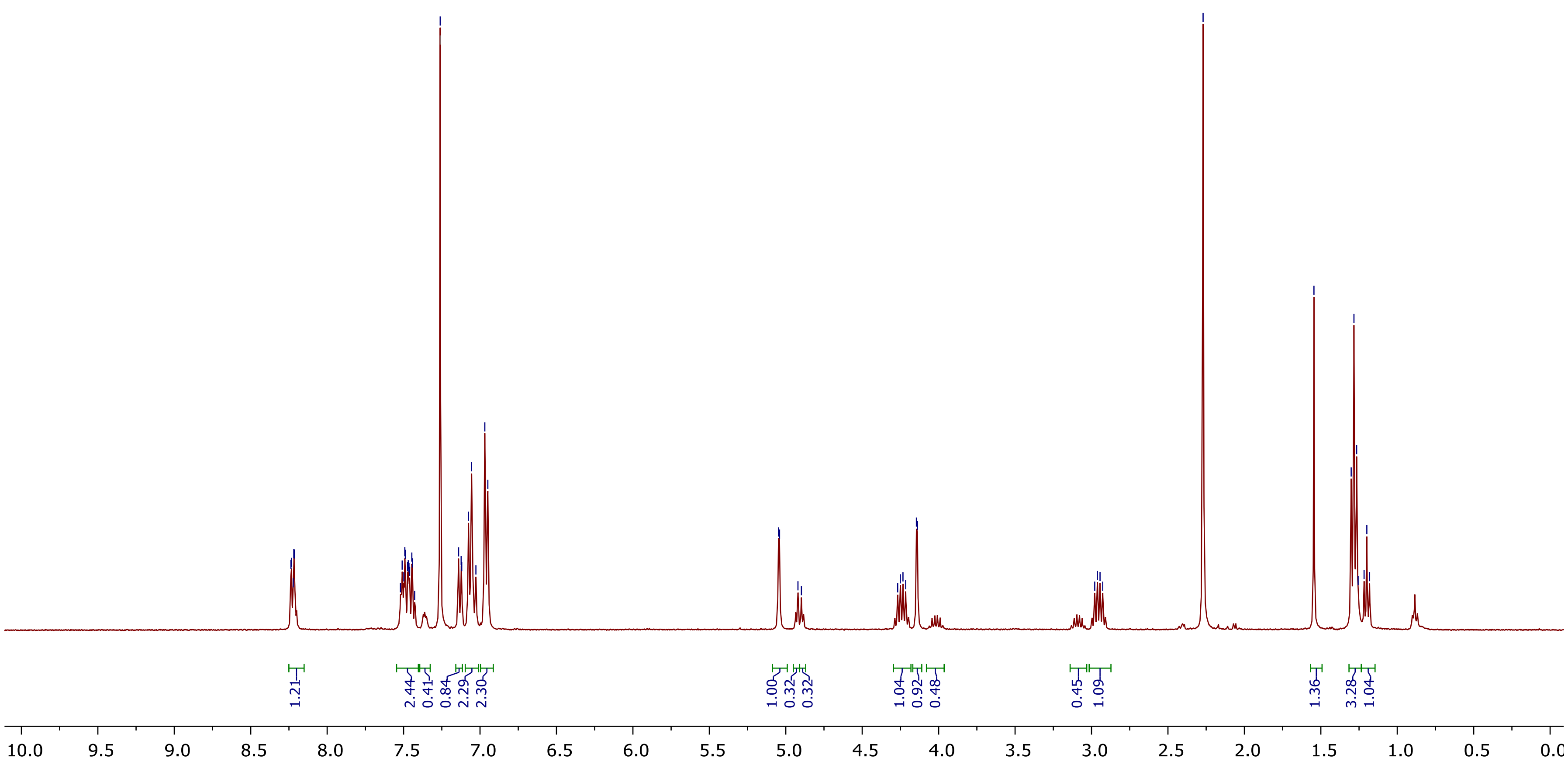

[1]13C NMR spectrum of compound 18c

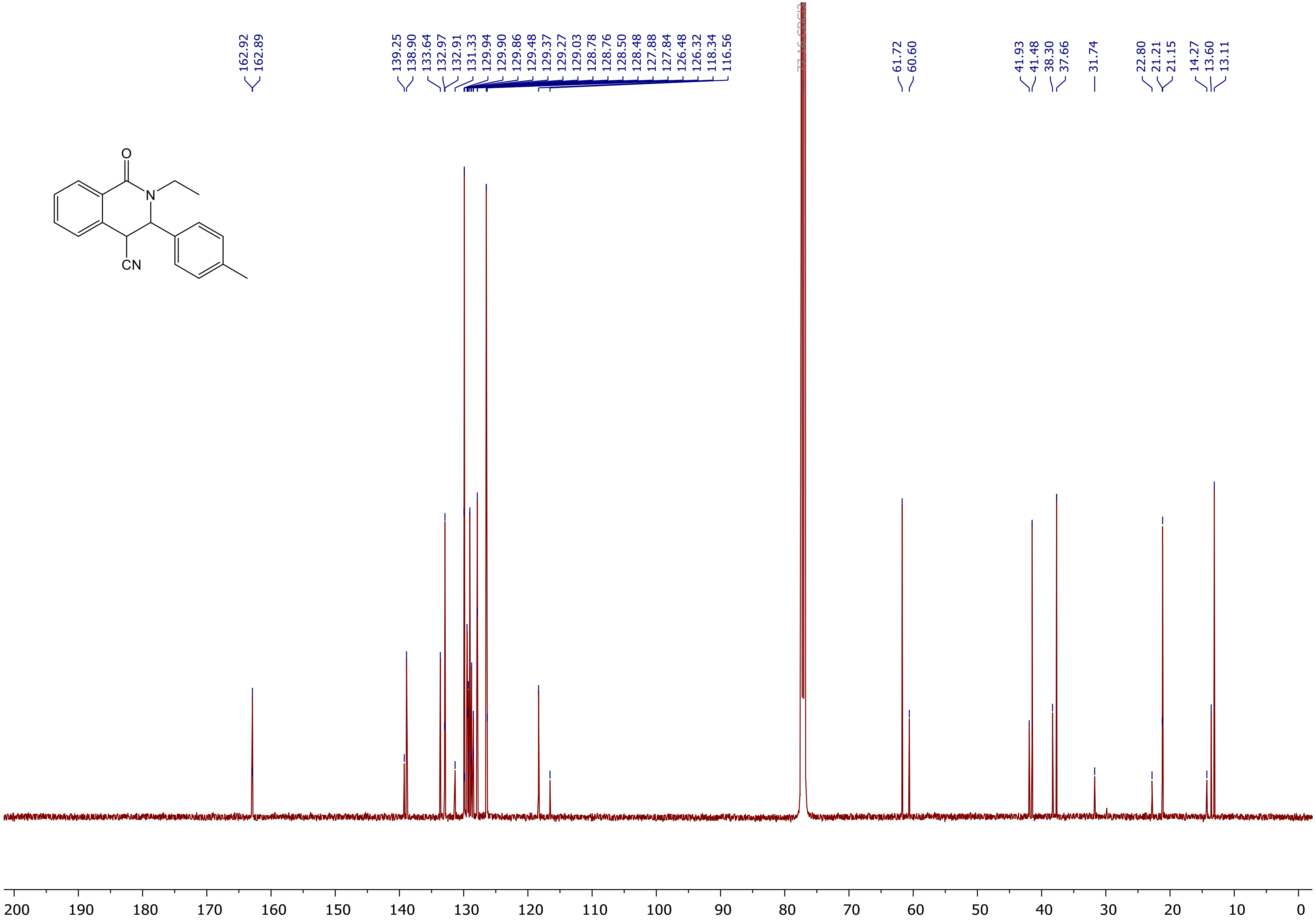

^{1}H NMR spectrum of compound 18d

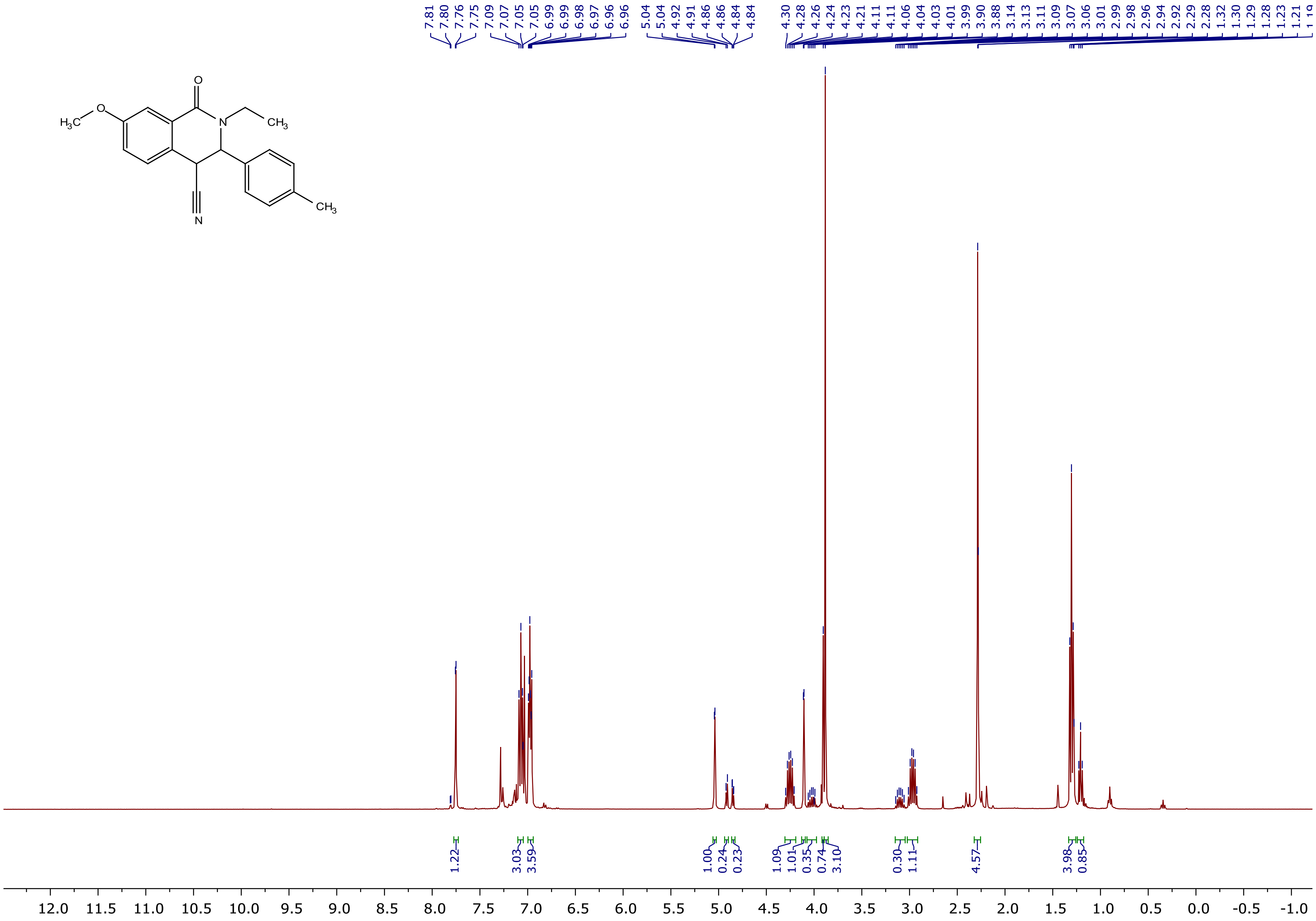

^{13}C NMR spectrum of compound 18d

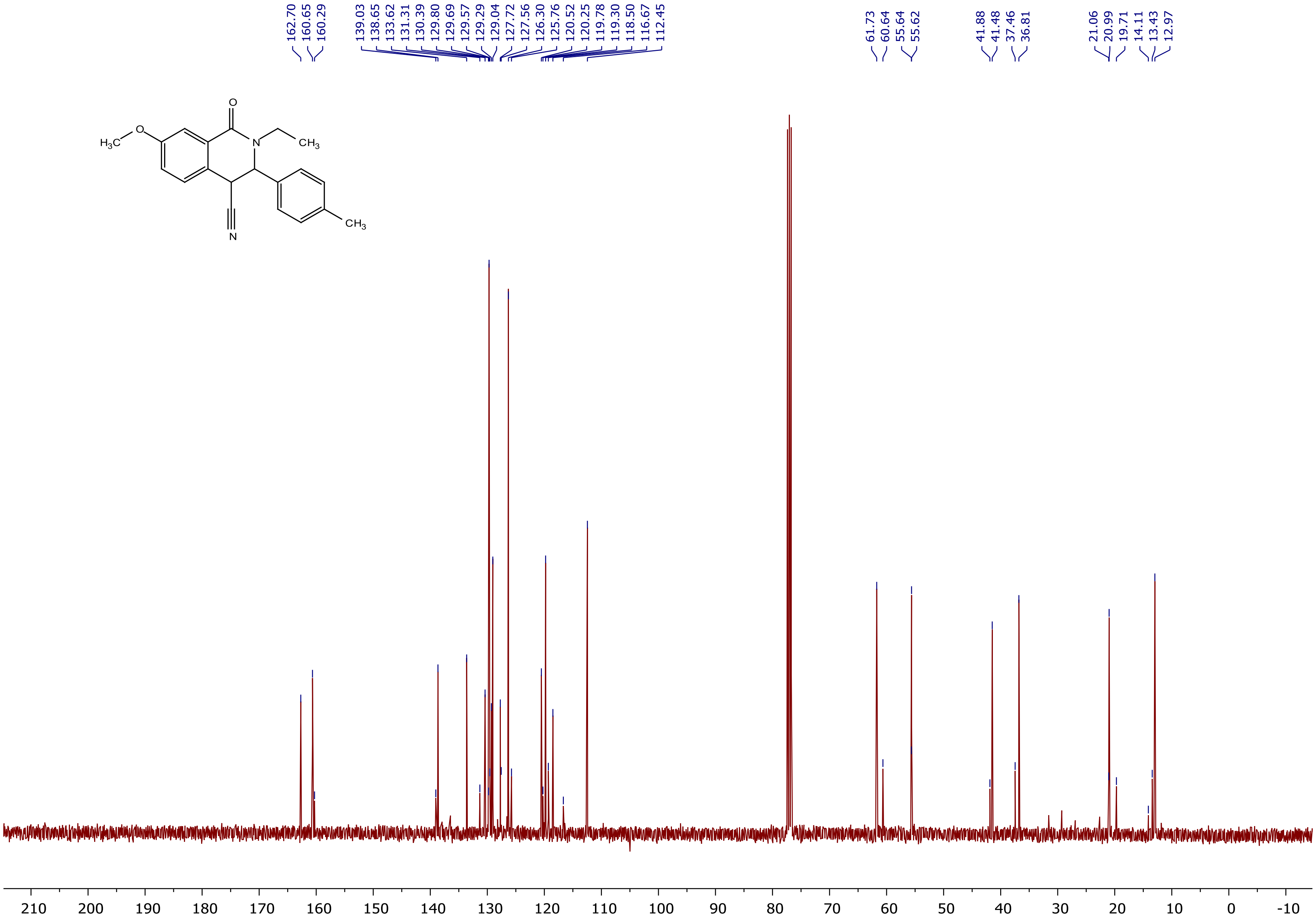

^{1}H NMR spectrum of compound 18e

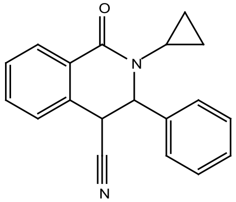

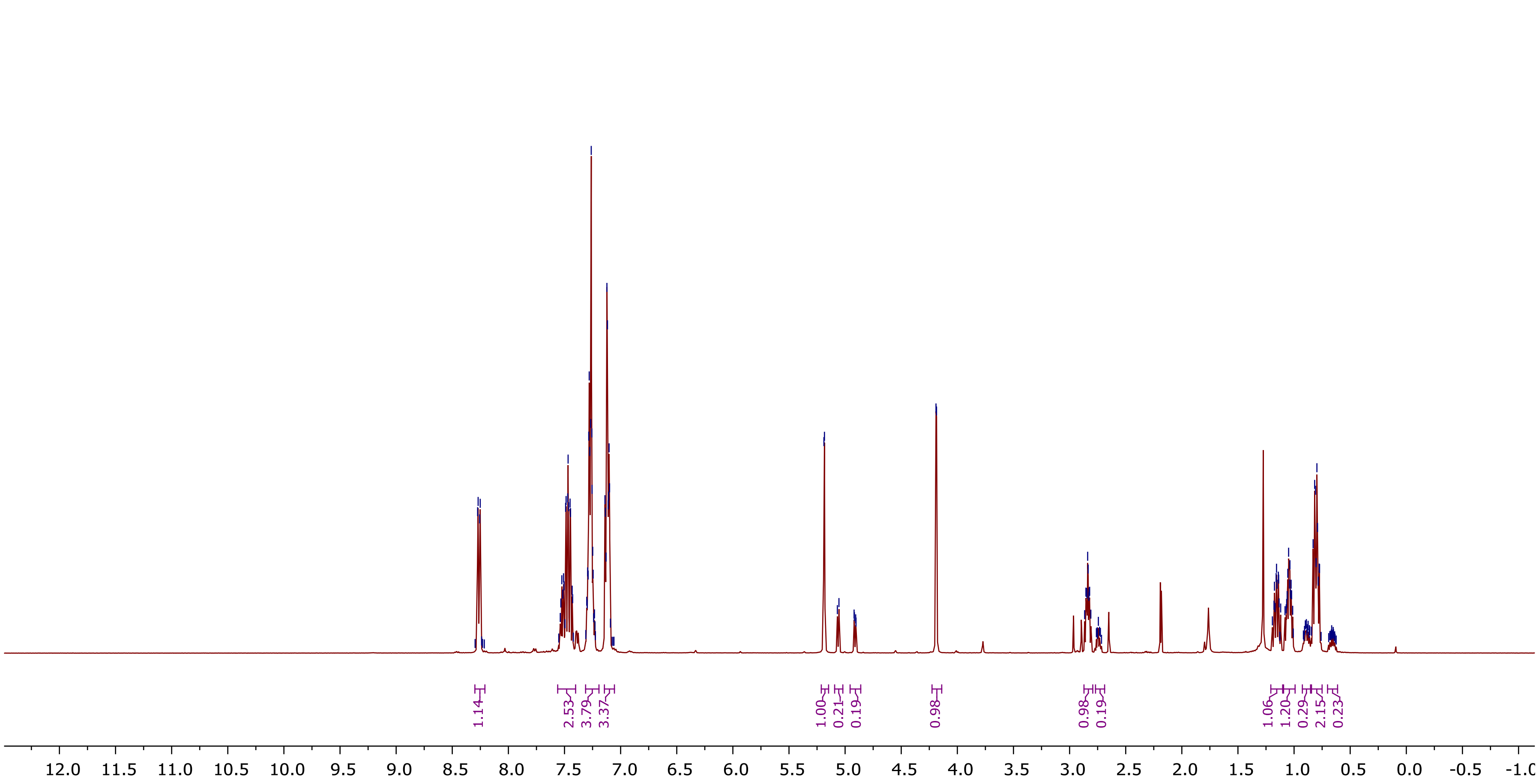

^{13}C NMR spectrum of compound 18e

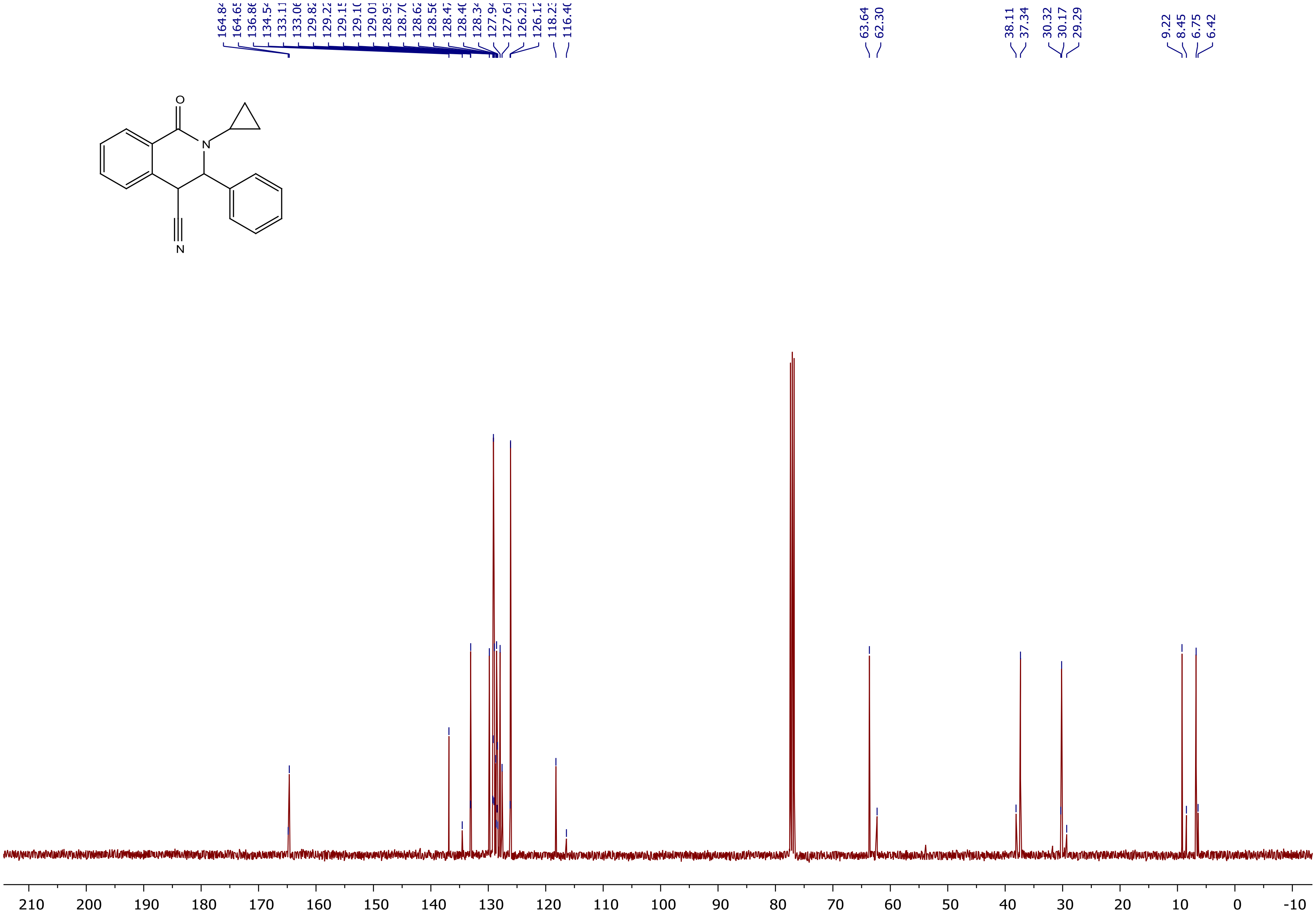

^{1}H NMR spectrum of compound 18f

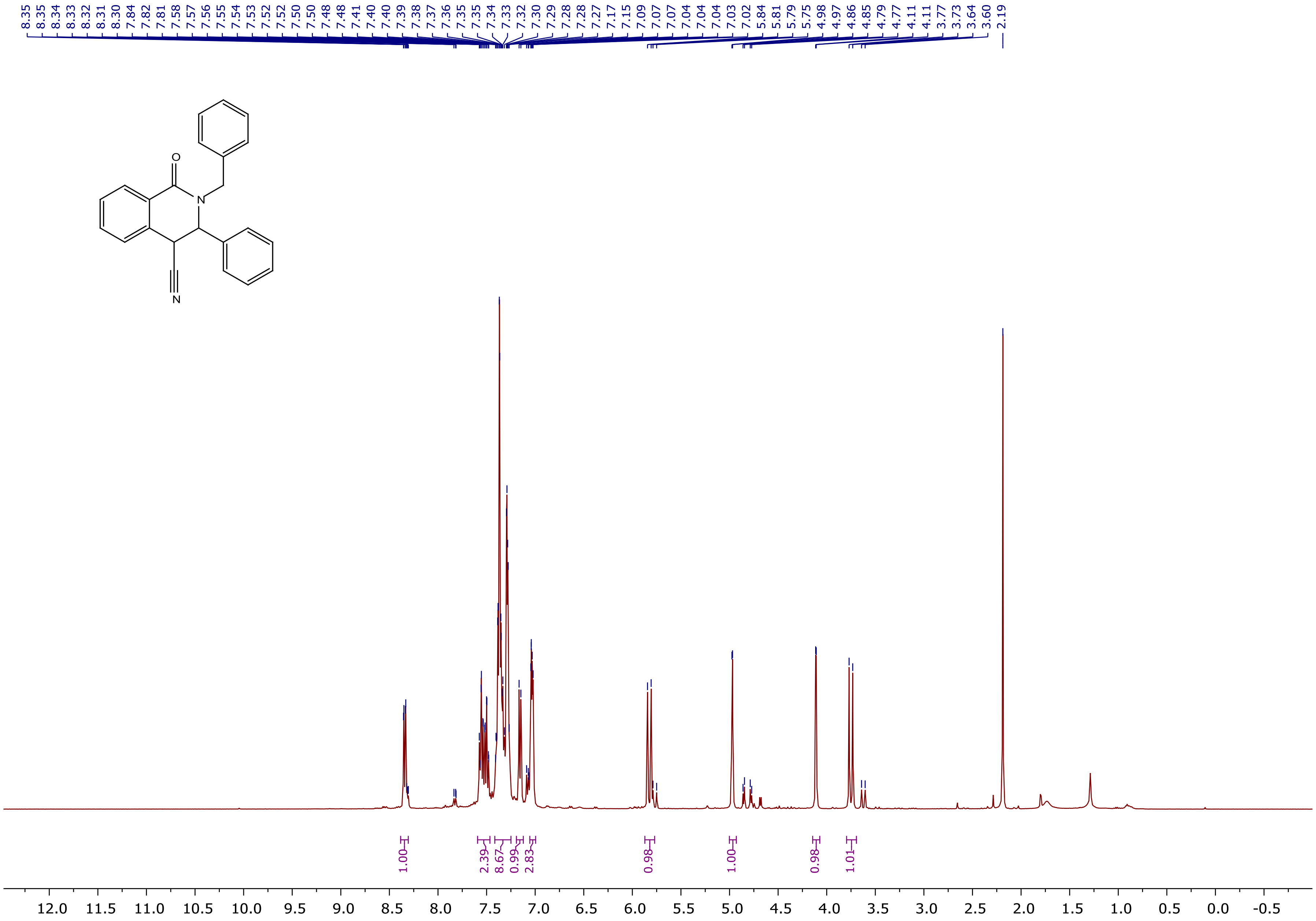

^{13}C NMR spectrum of compound 18f

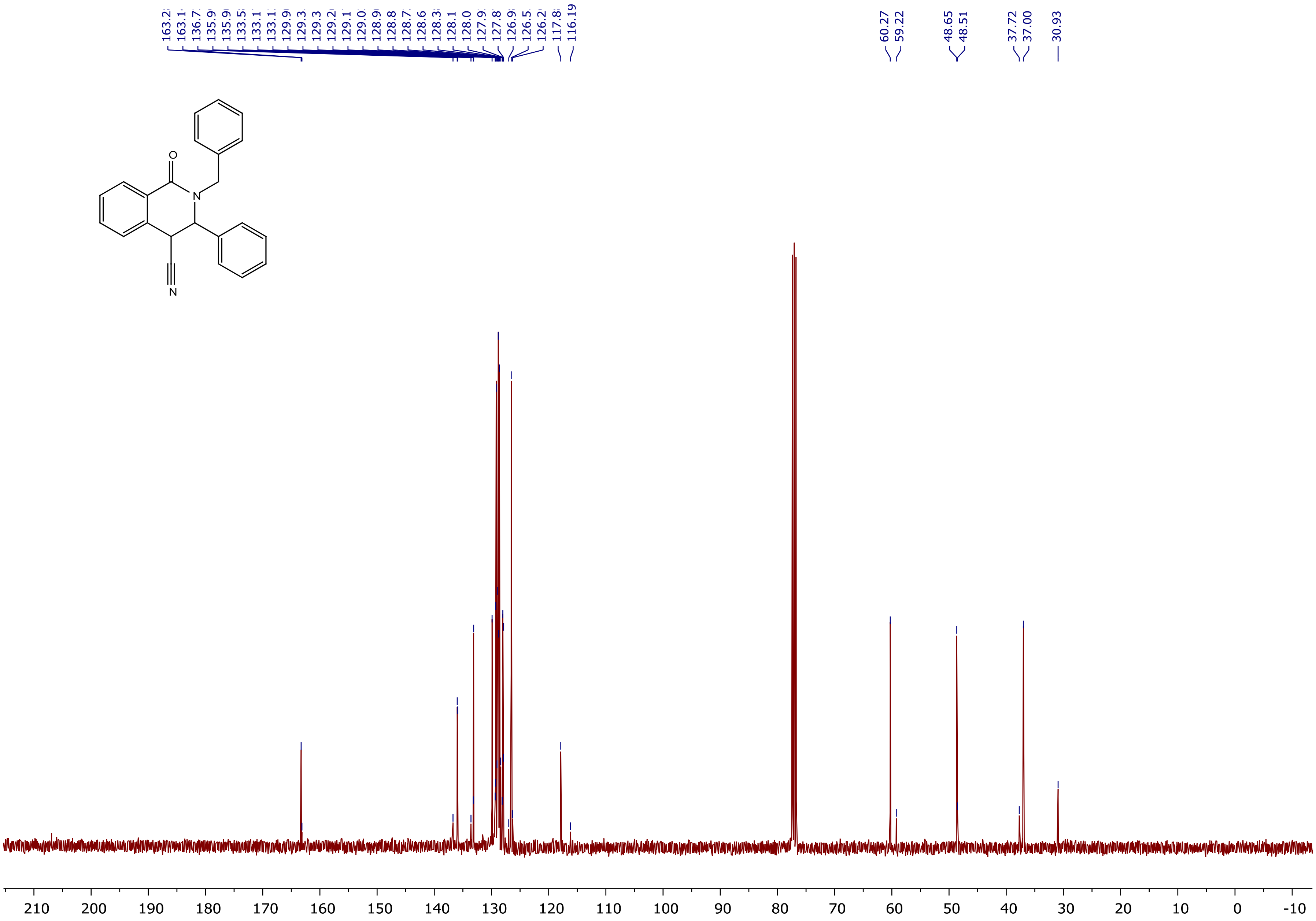

^{1}H NMR spectrum of compound 18g

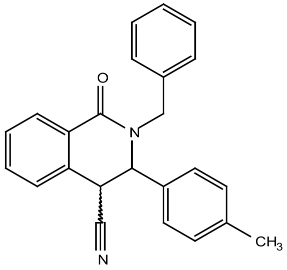

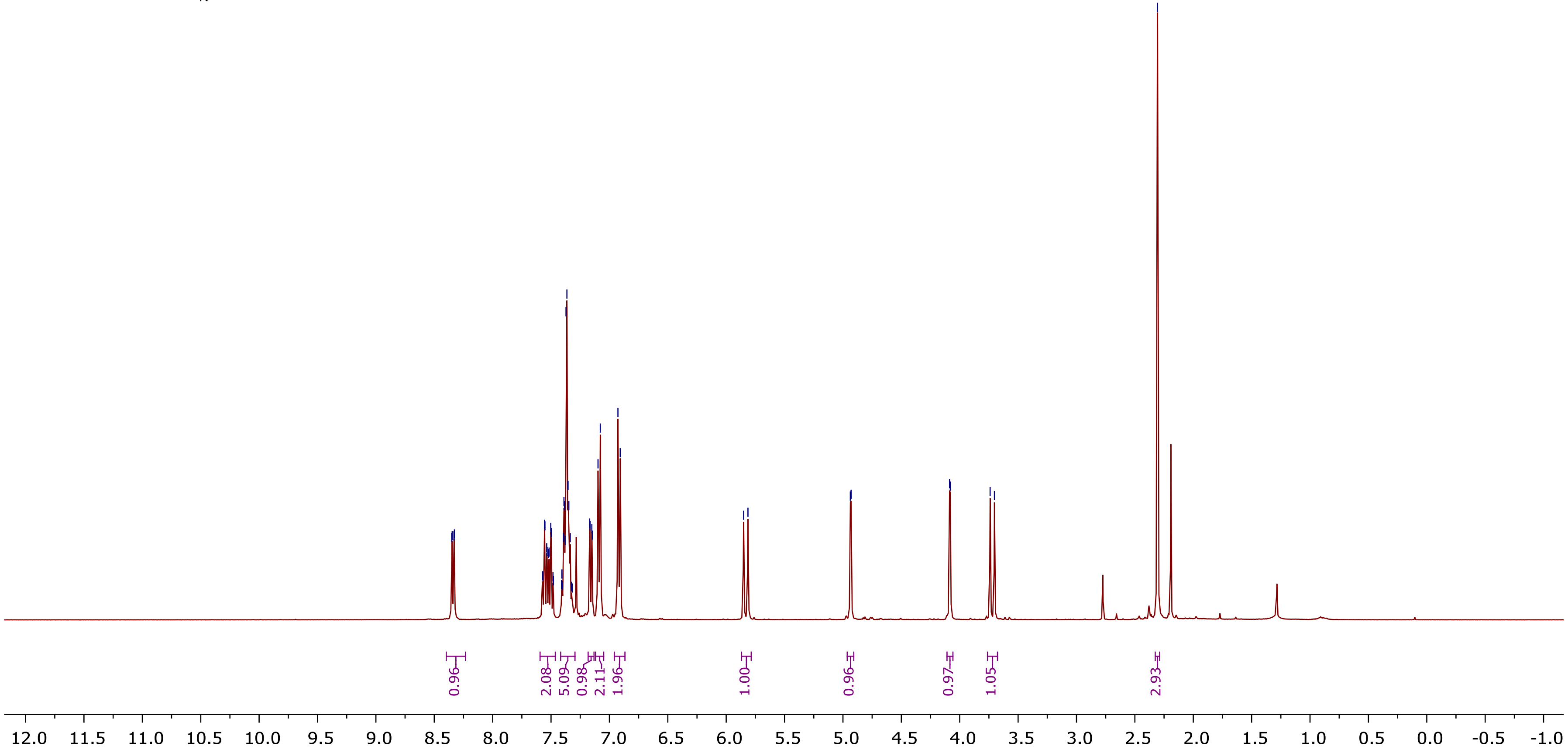

^{13}C NMR spectrum of compound 18g

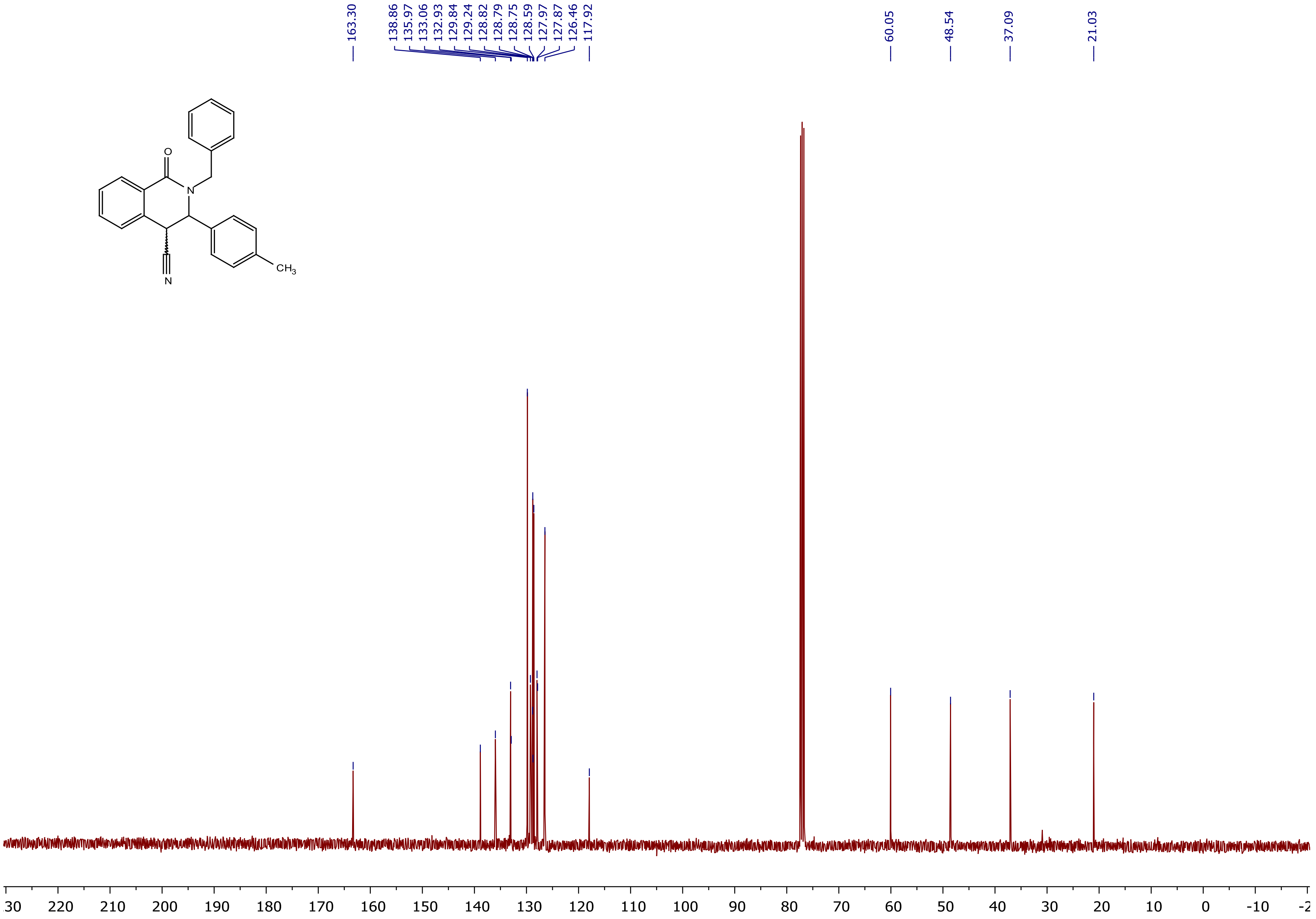

^{1}H NMR spectrum of compound 18h

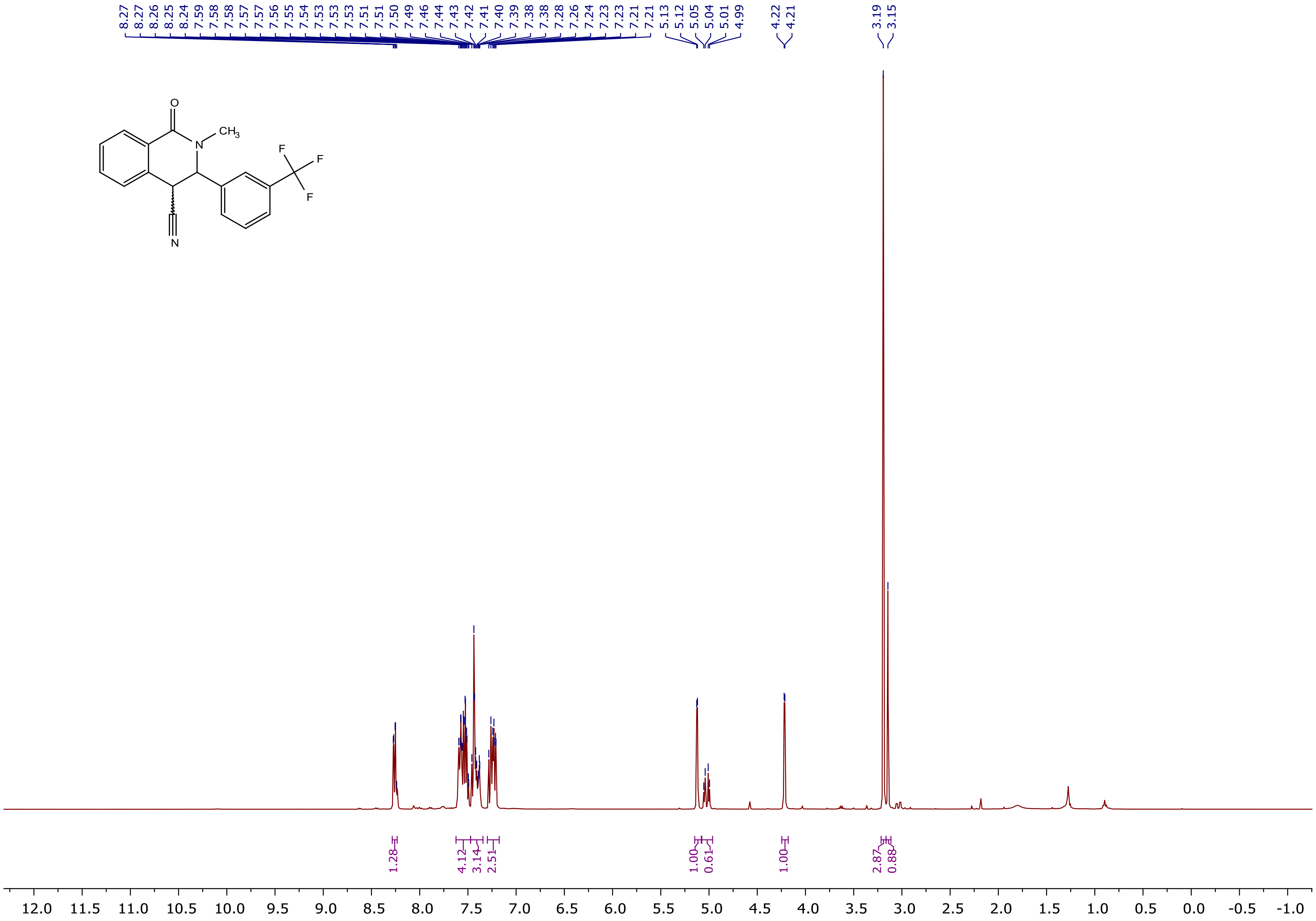

^{13}C NMR spectrum of compound 18h

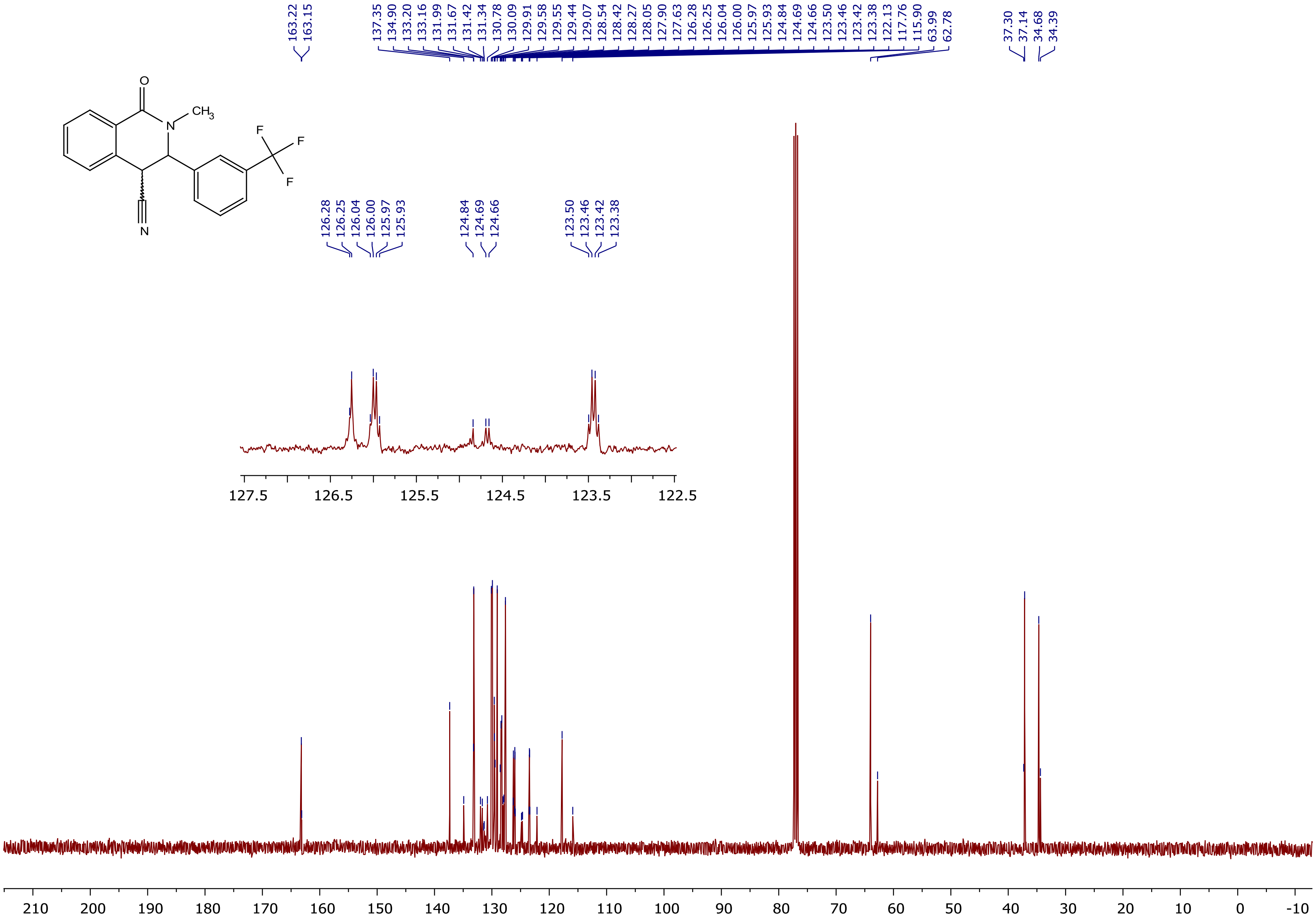

^{19}F NMR spectrum of compound 18h

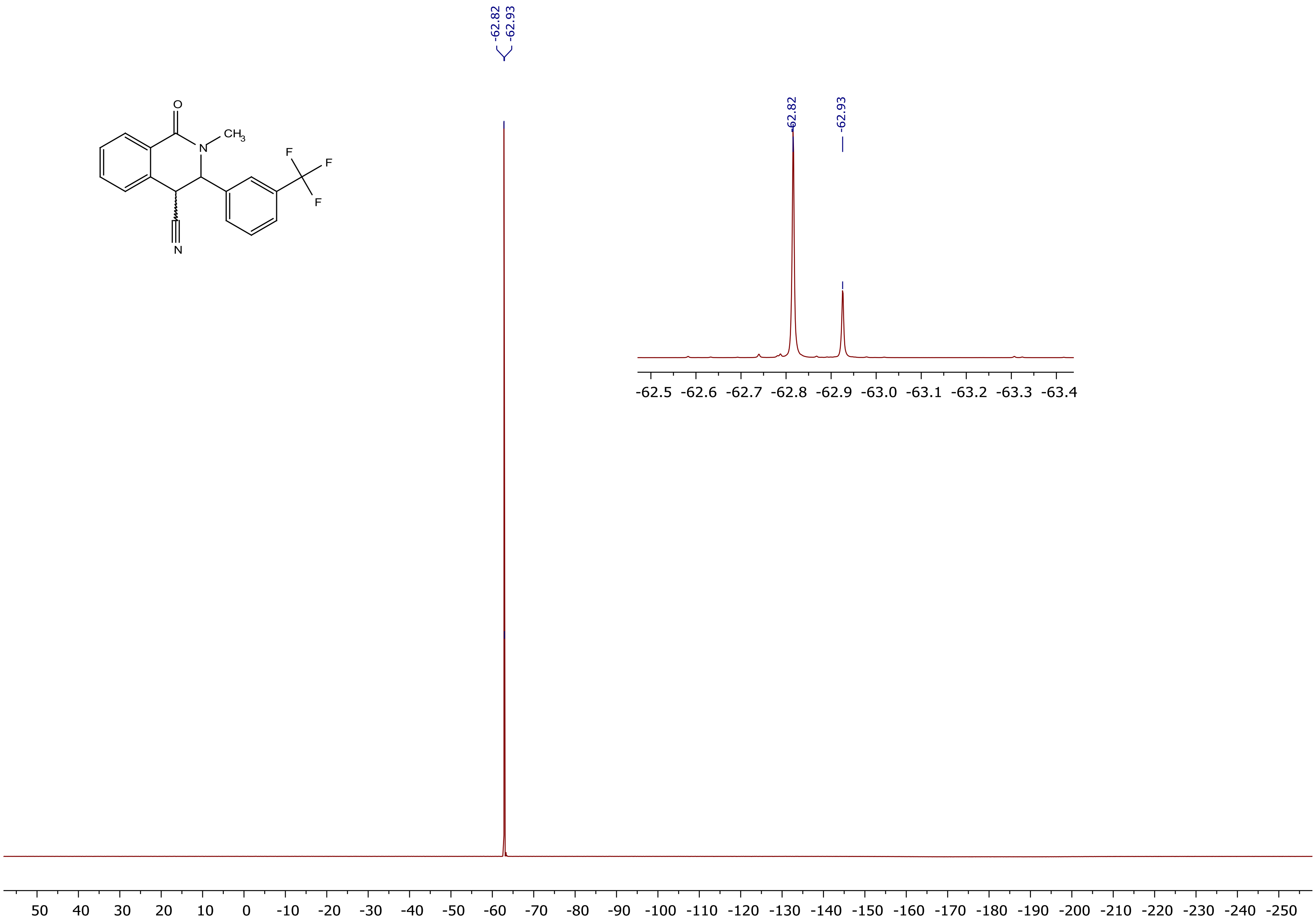

^{1}H NMR spectrum of compound 18i

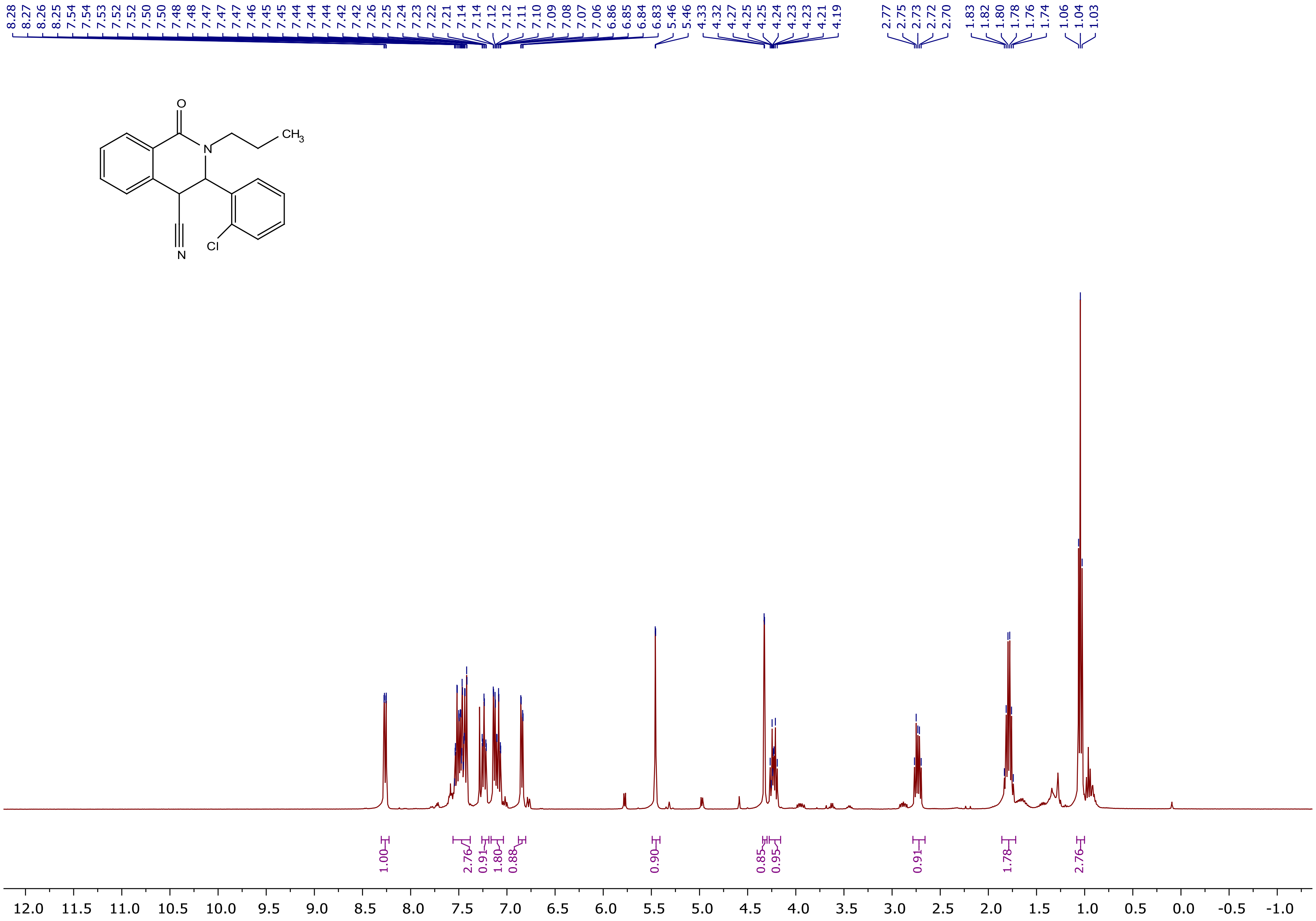

^{13}C NMR spectrum of compound 18i

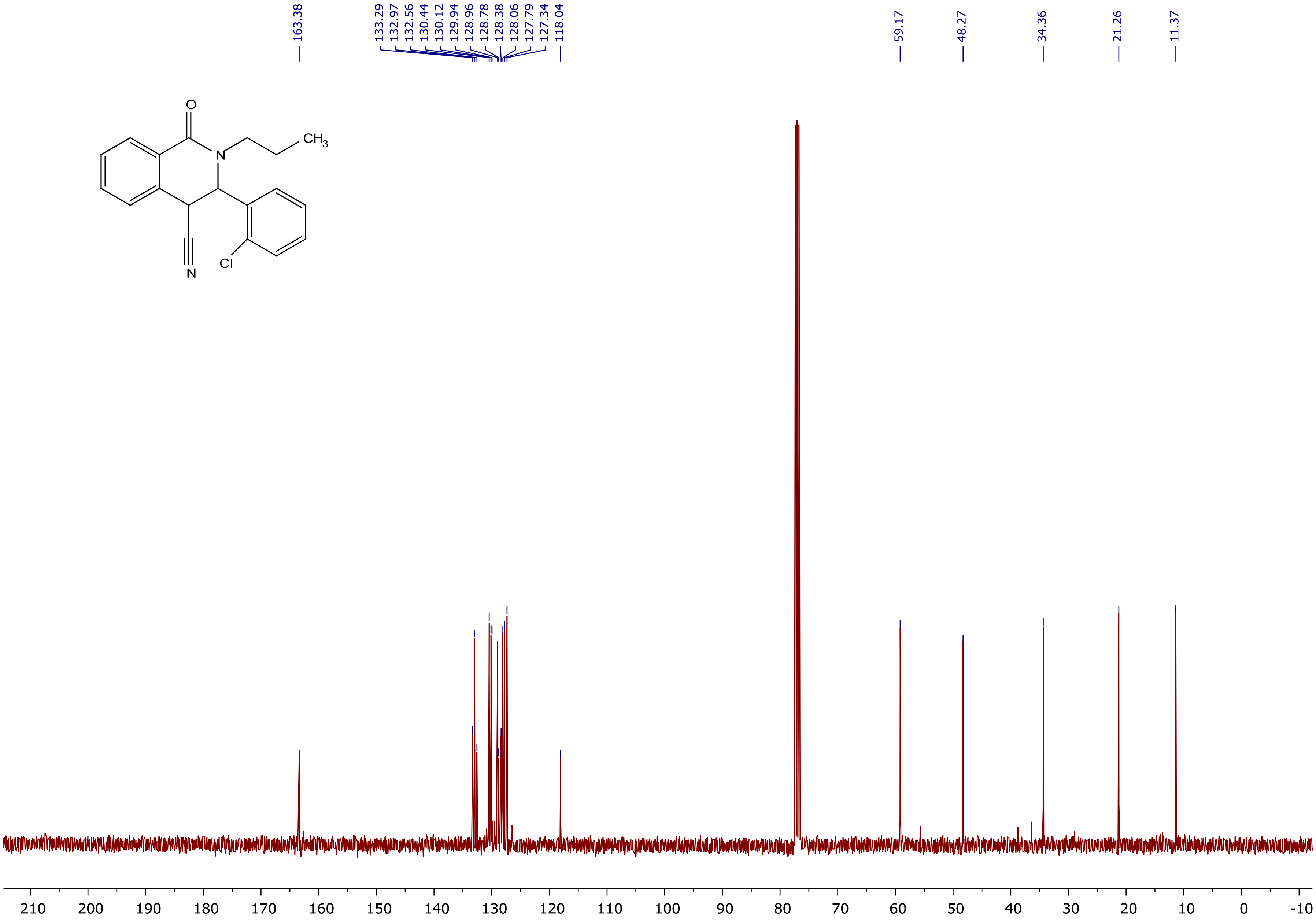

^{1}H NMR spectrum of compound 18j

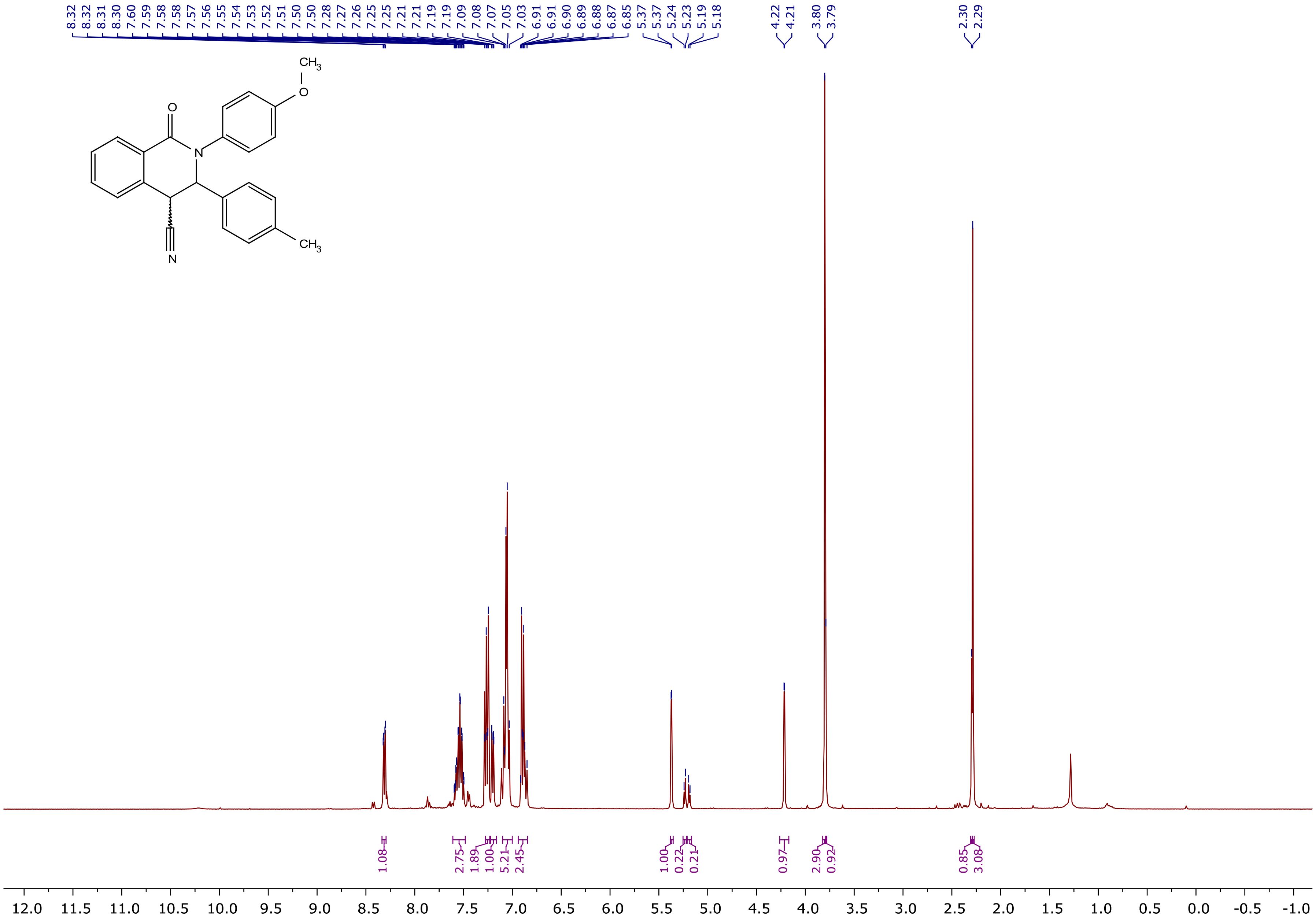

^{13}C NMR spectrum of compound 18j

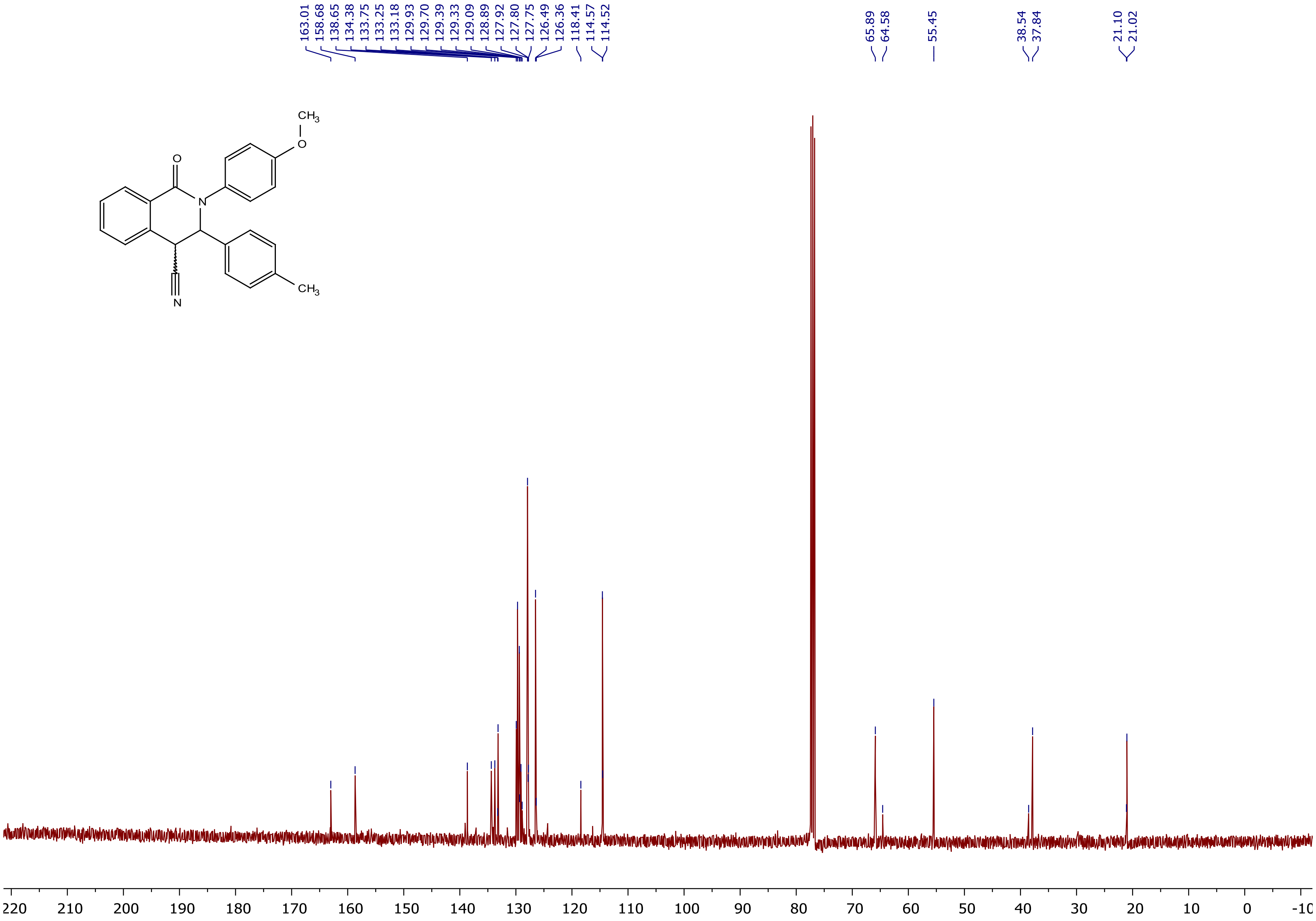

^{1}H NMR spectrum of compound 18k

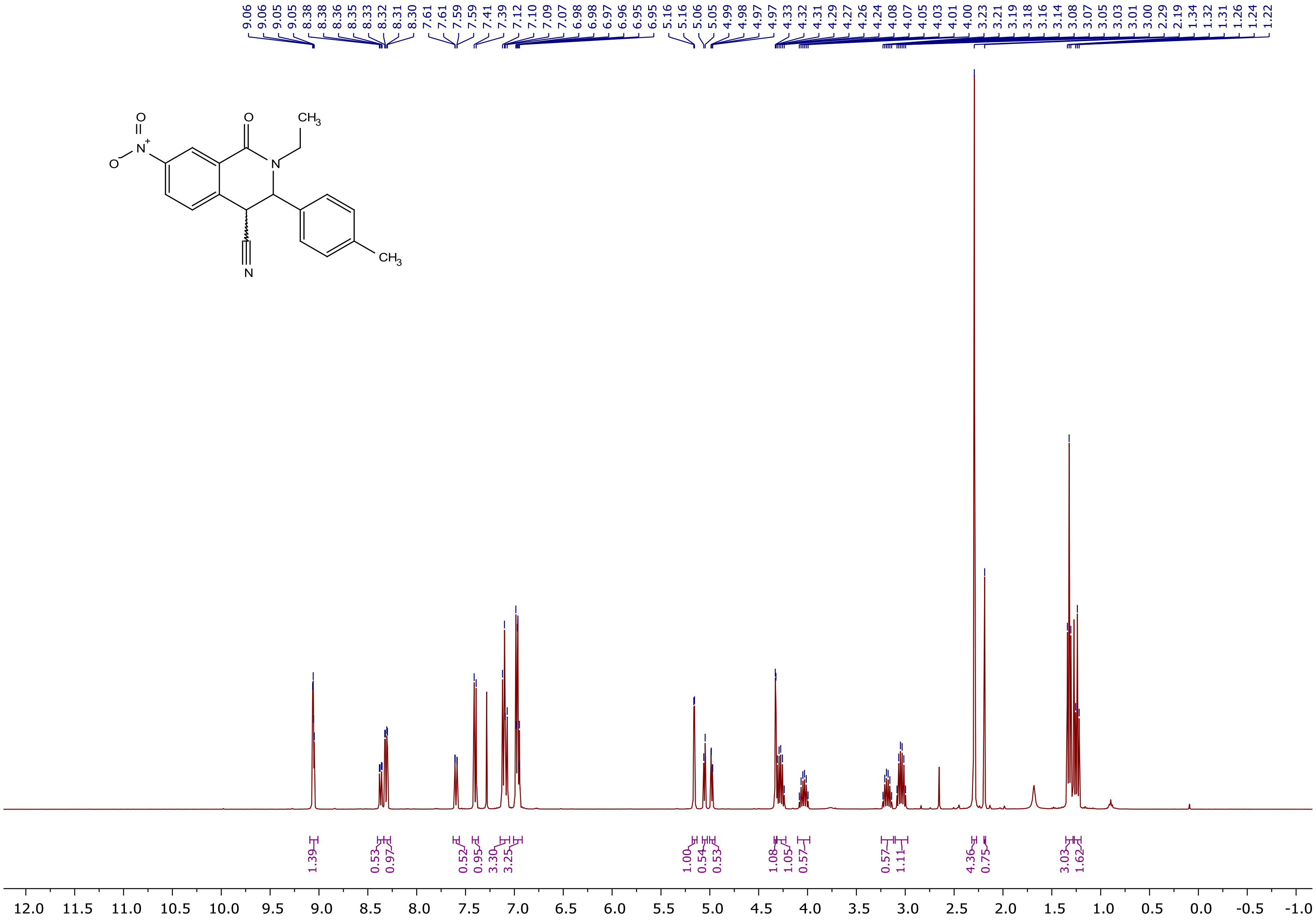

^{13}C NMR spectrum of compound 18k

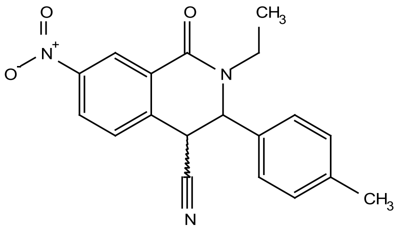

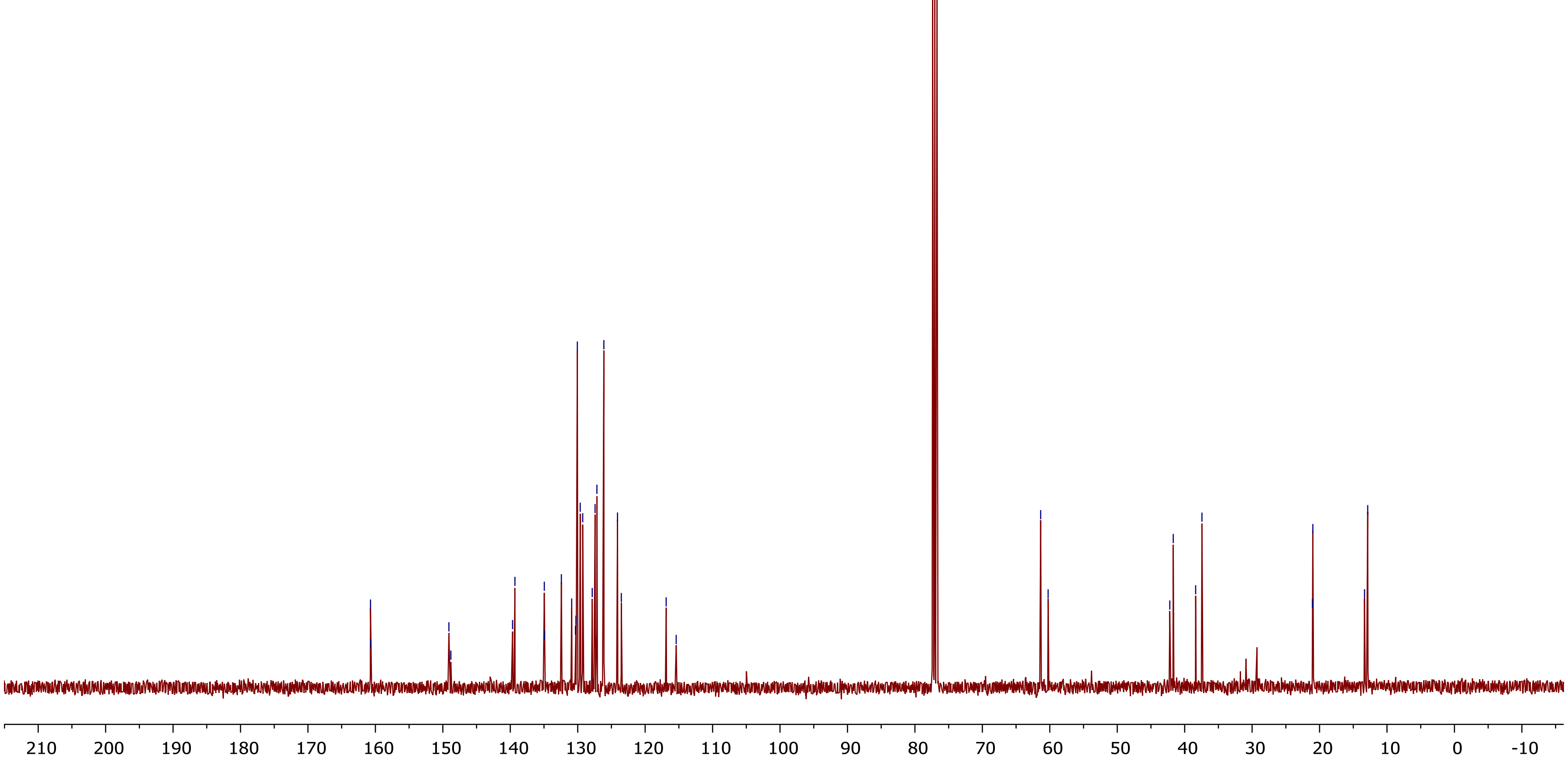

^{1}H NMR spectrum of compound 18l

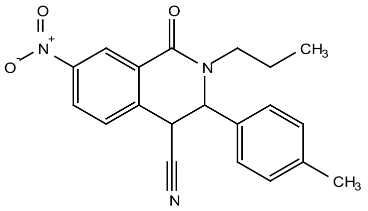

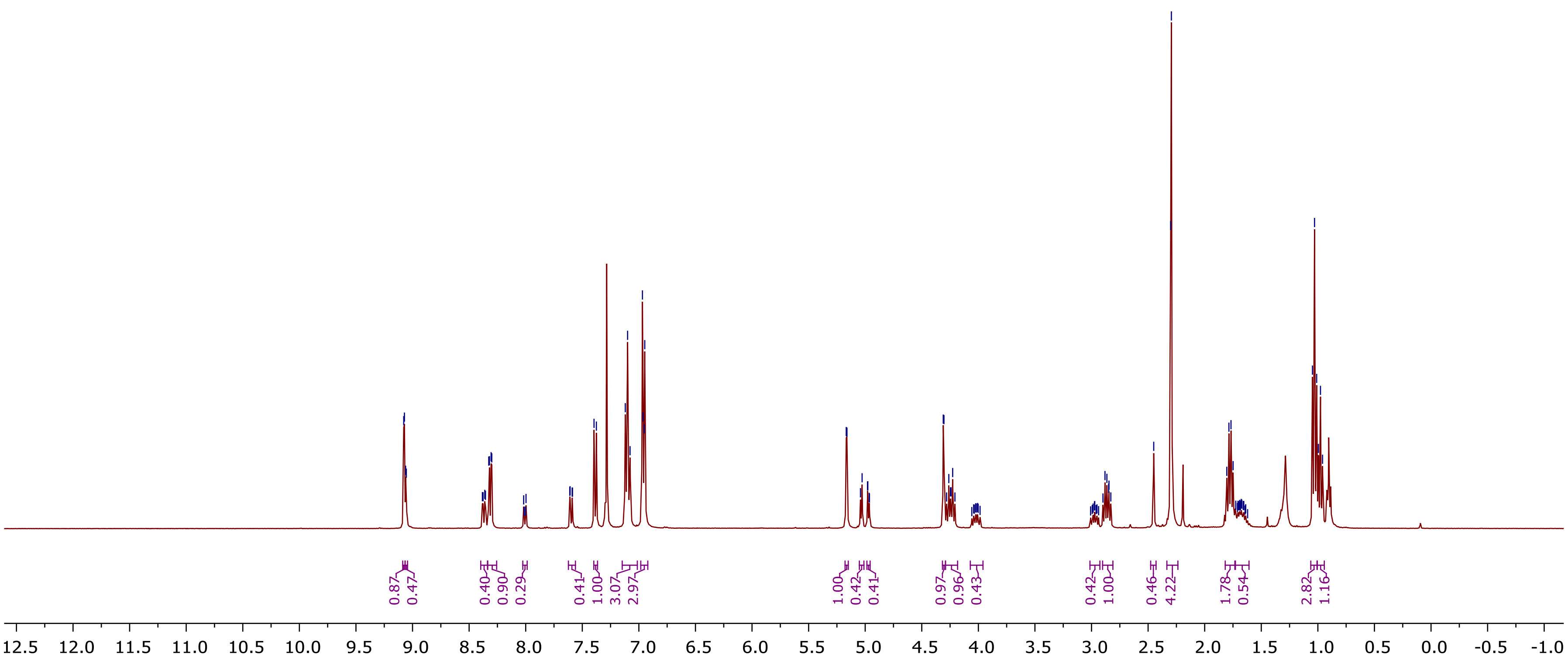

^{13}C NMR spectrum of compound 18l

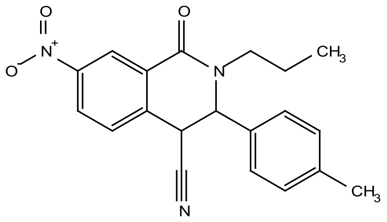

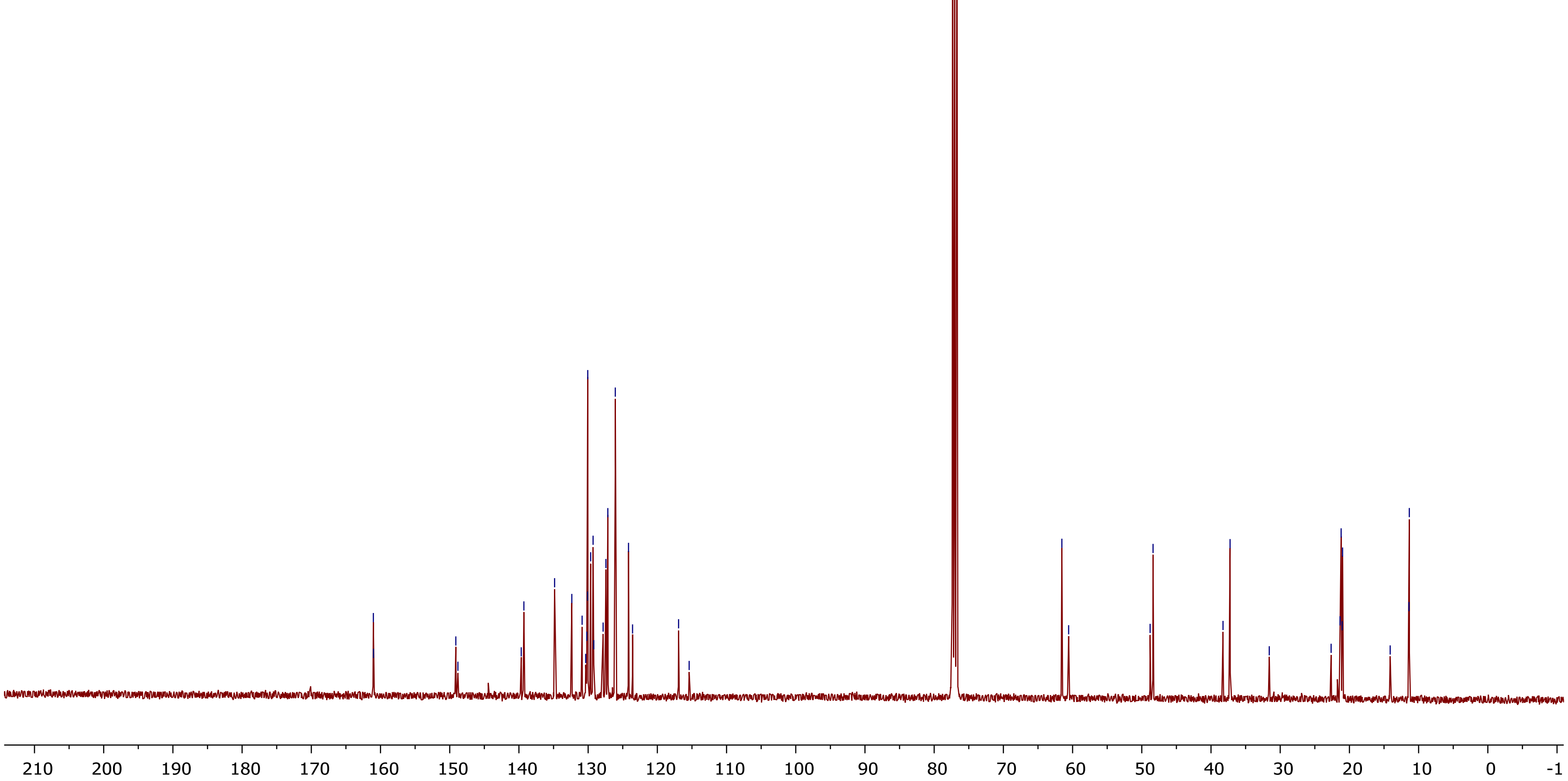

^{1}H NMR spectrum of compound 18m

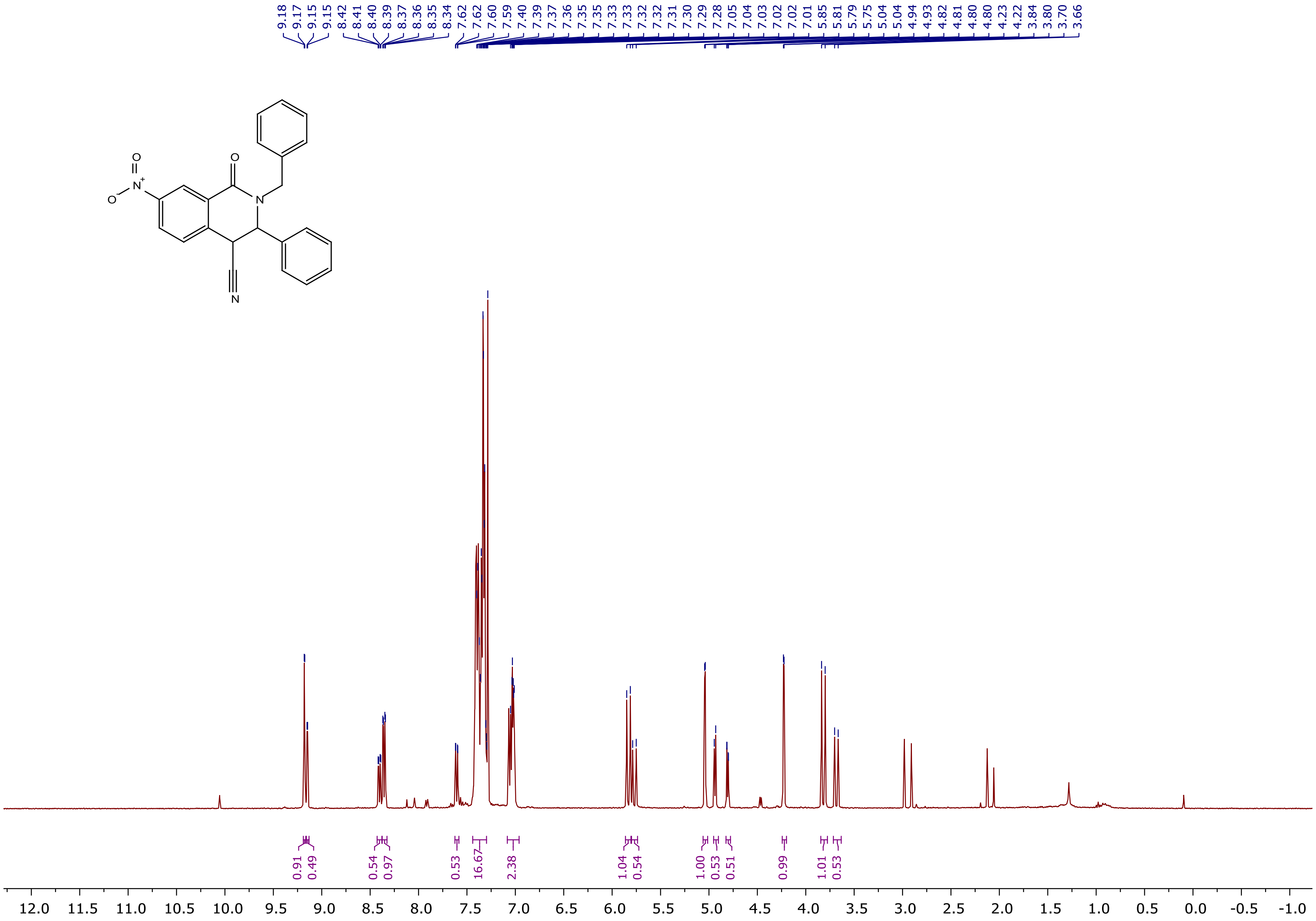

^{13}C NMR spectrum of compound 18m

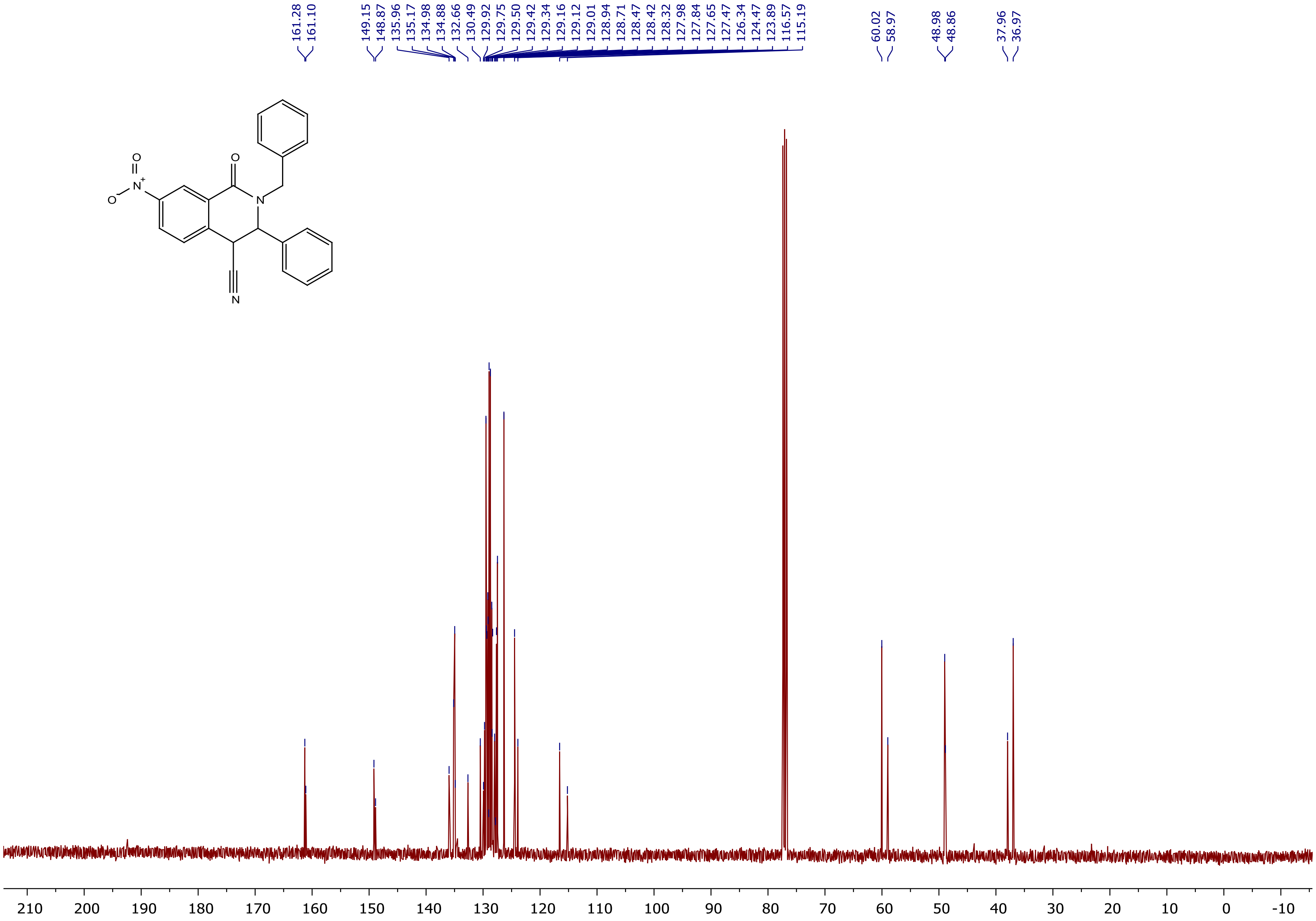

^{1}H NMR spectrum of compound 18n

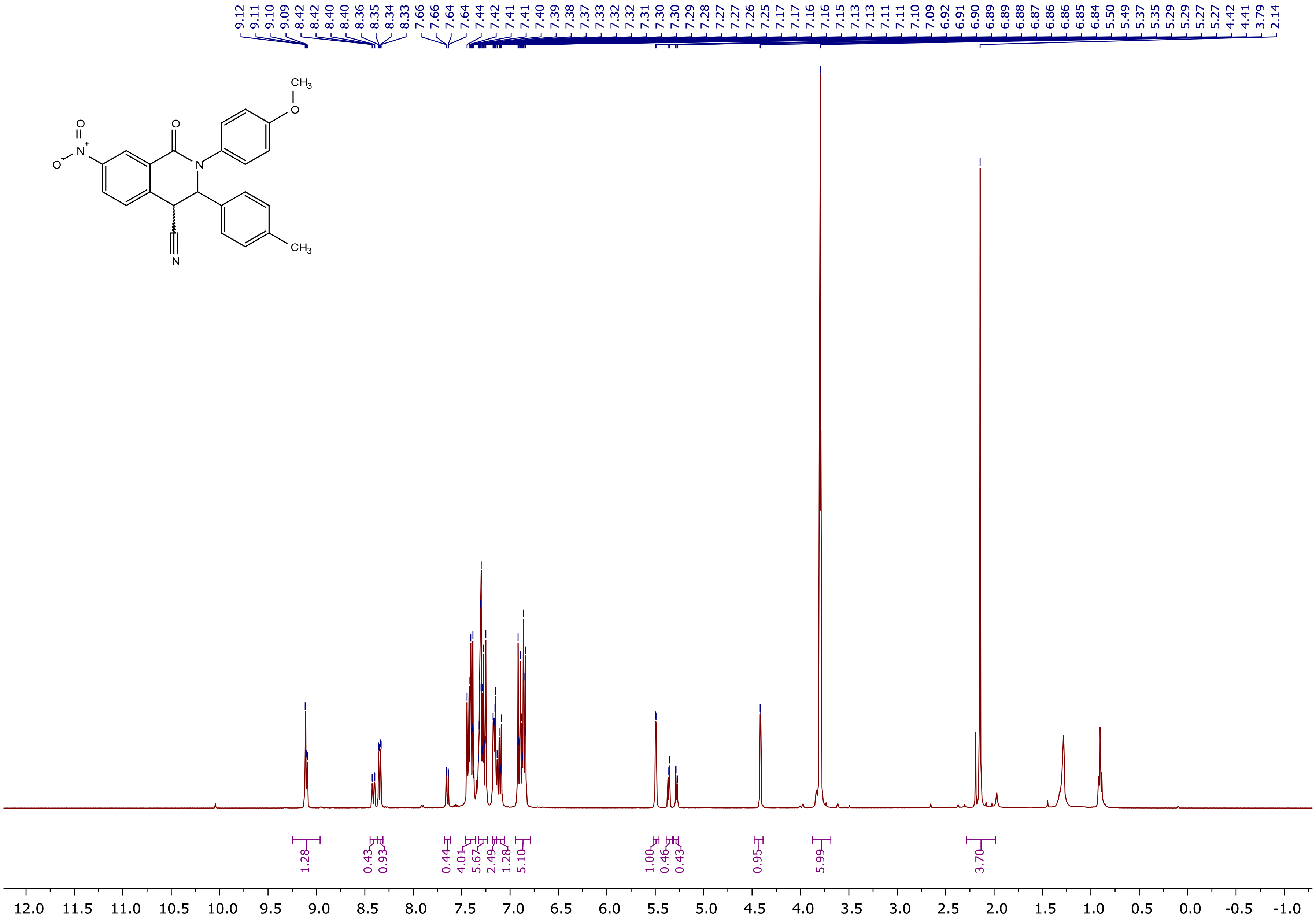

^{13}C NMR spectrum of compound 18n

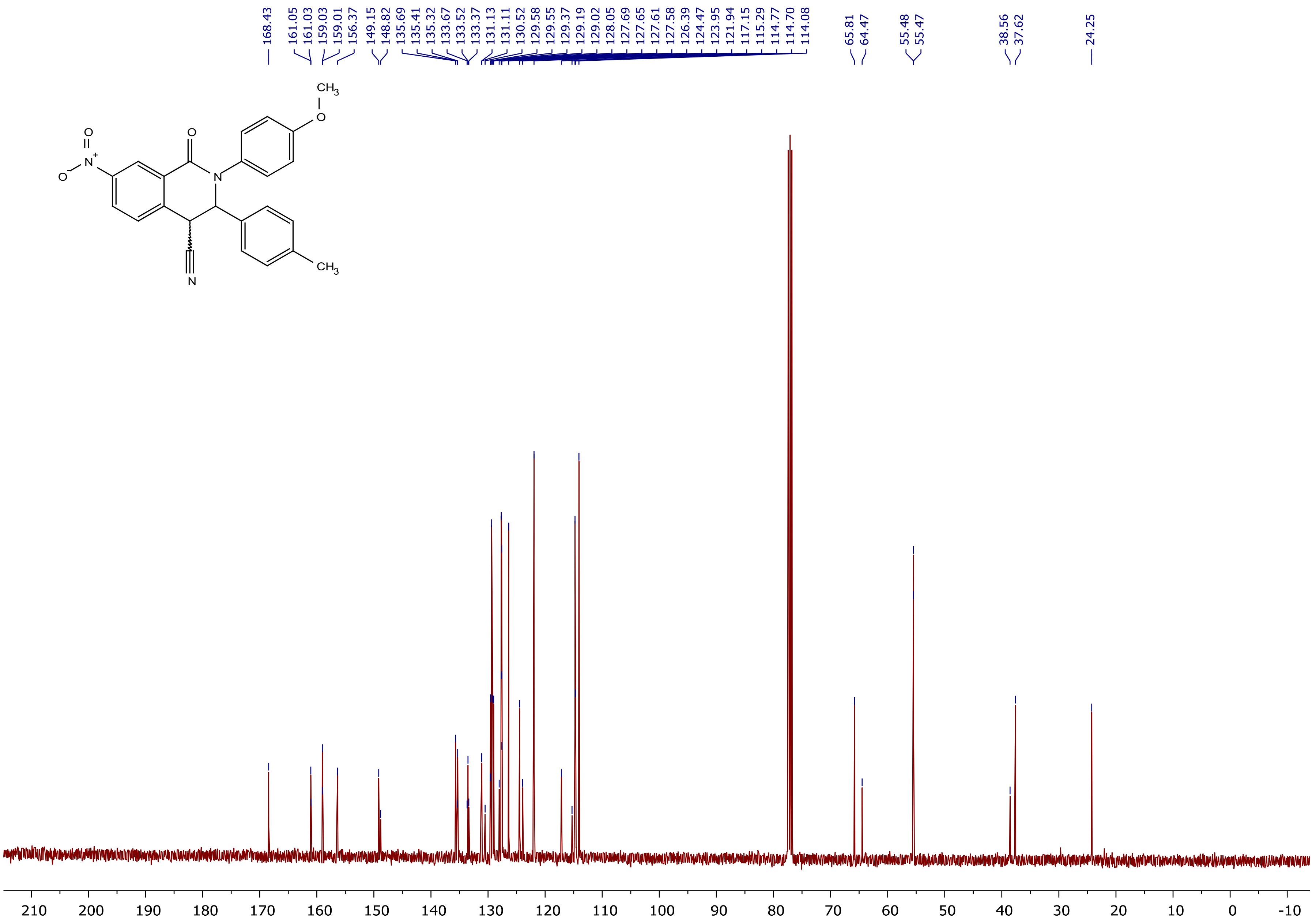

^{1}H NMR spectrum of compound 18o

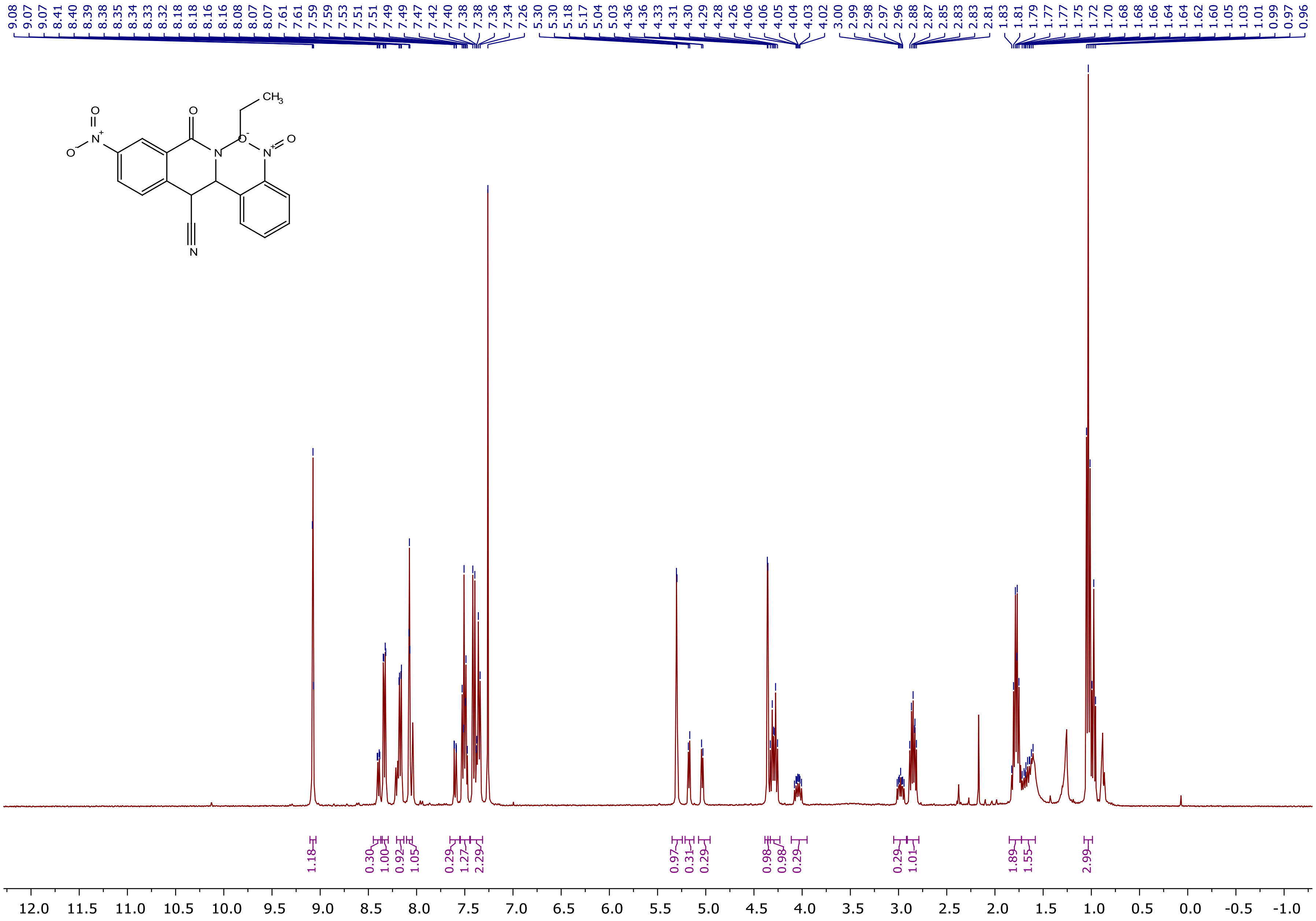

^{13}C NMR spectrum of compound 18o

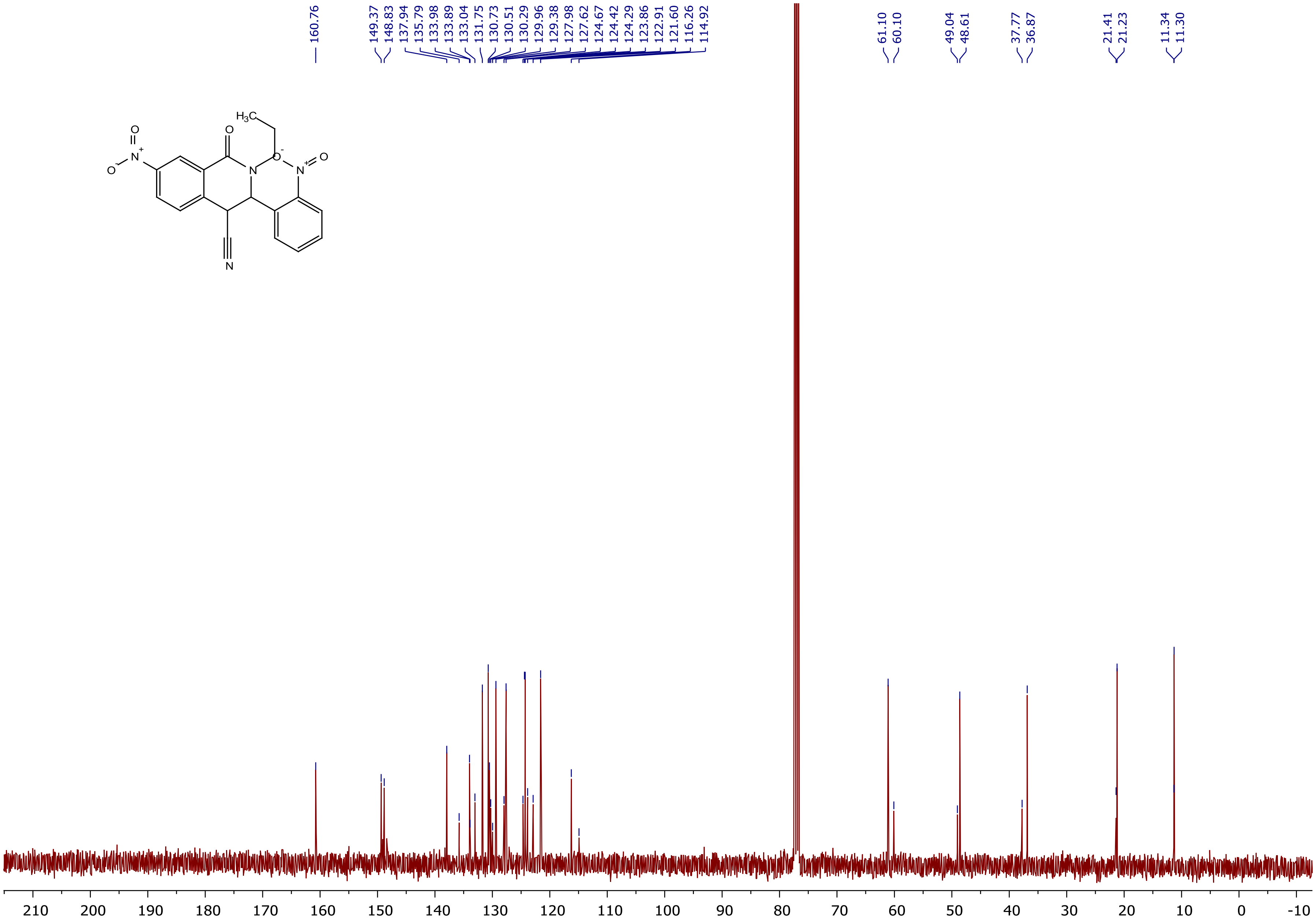

^{1}H NMR spectrum of compound 18p

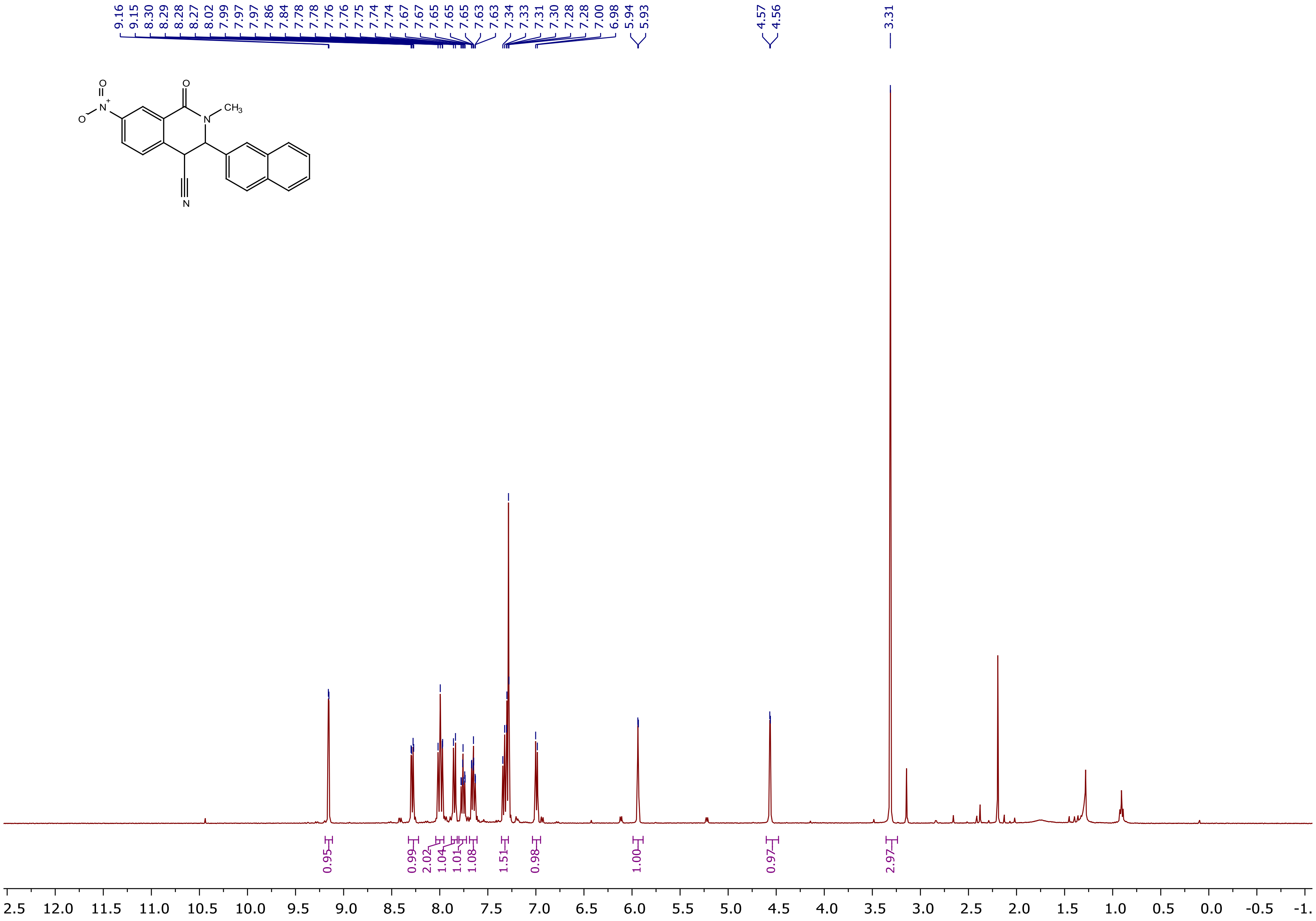

^{13}C NMR spectrum of compound 18p

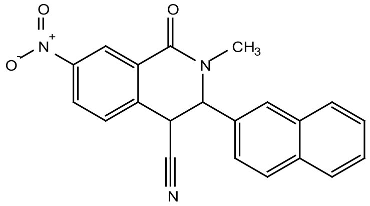

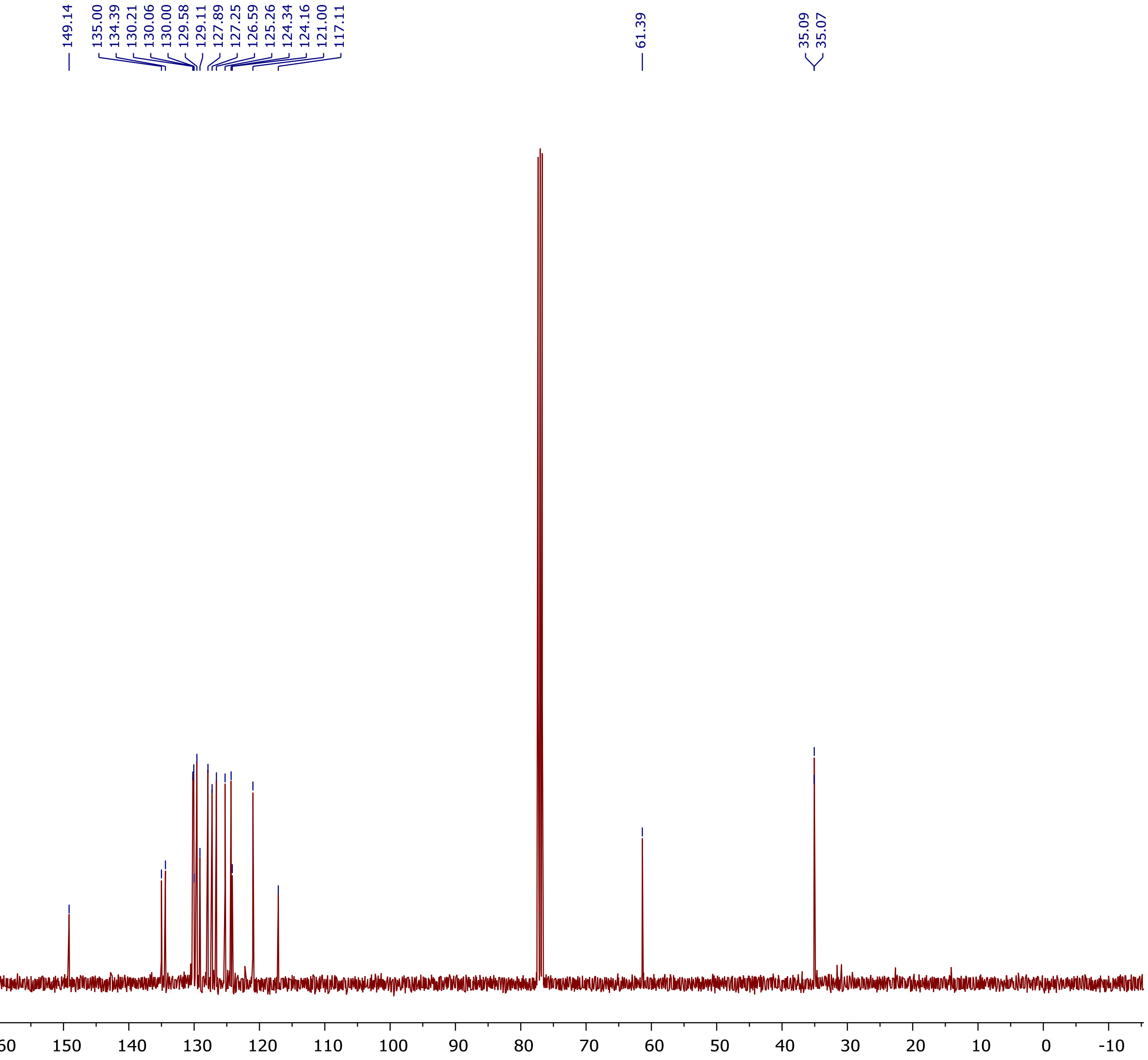

^{1}H NMR spectrum of compound 18q

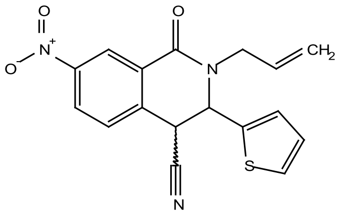

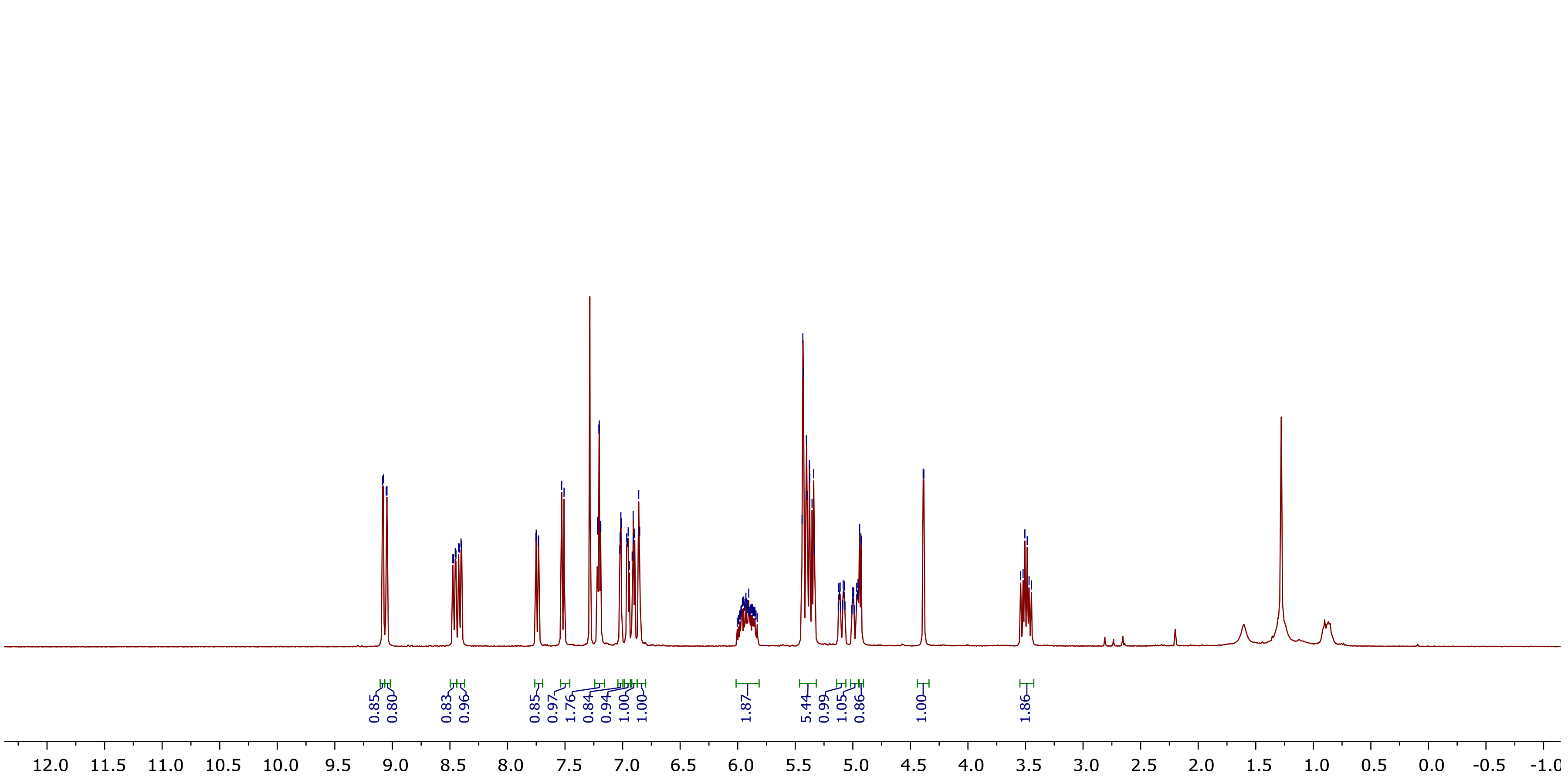

^{13}C NMR spectrum of compound 18q

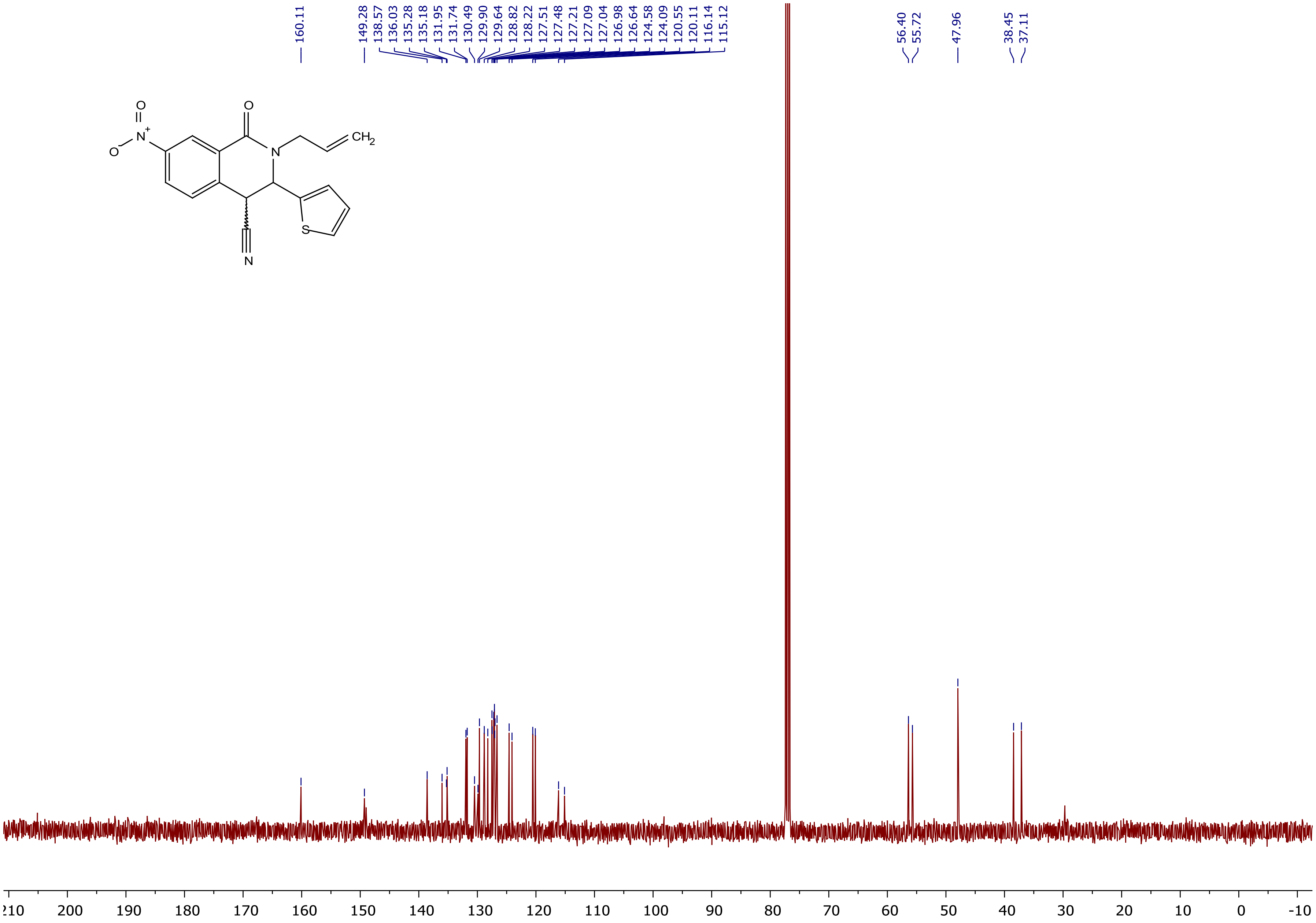

^{1}H NMR spectrum of compound 19a

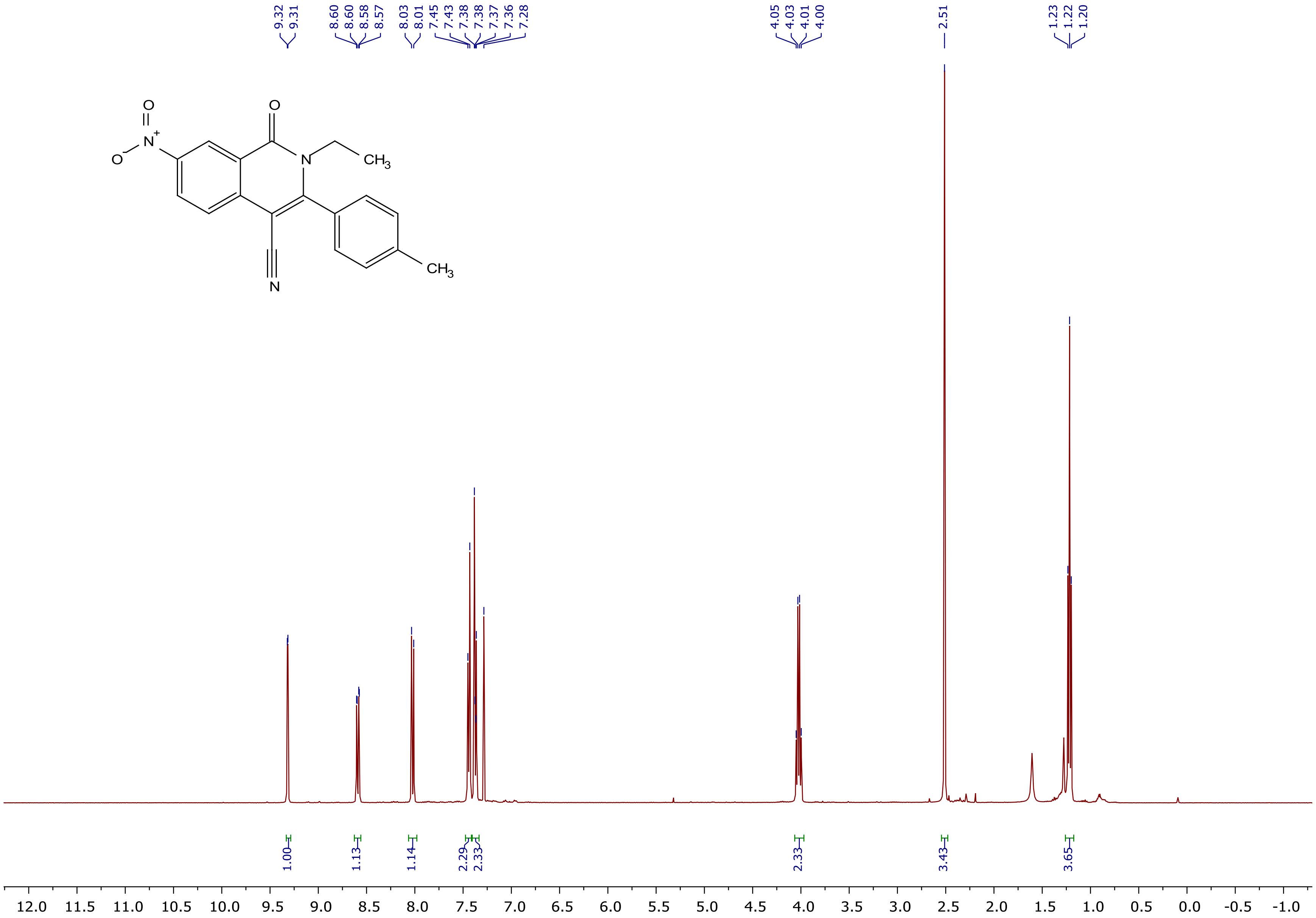

^{13}C NMR spectrum of compound 19a

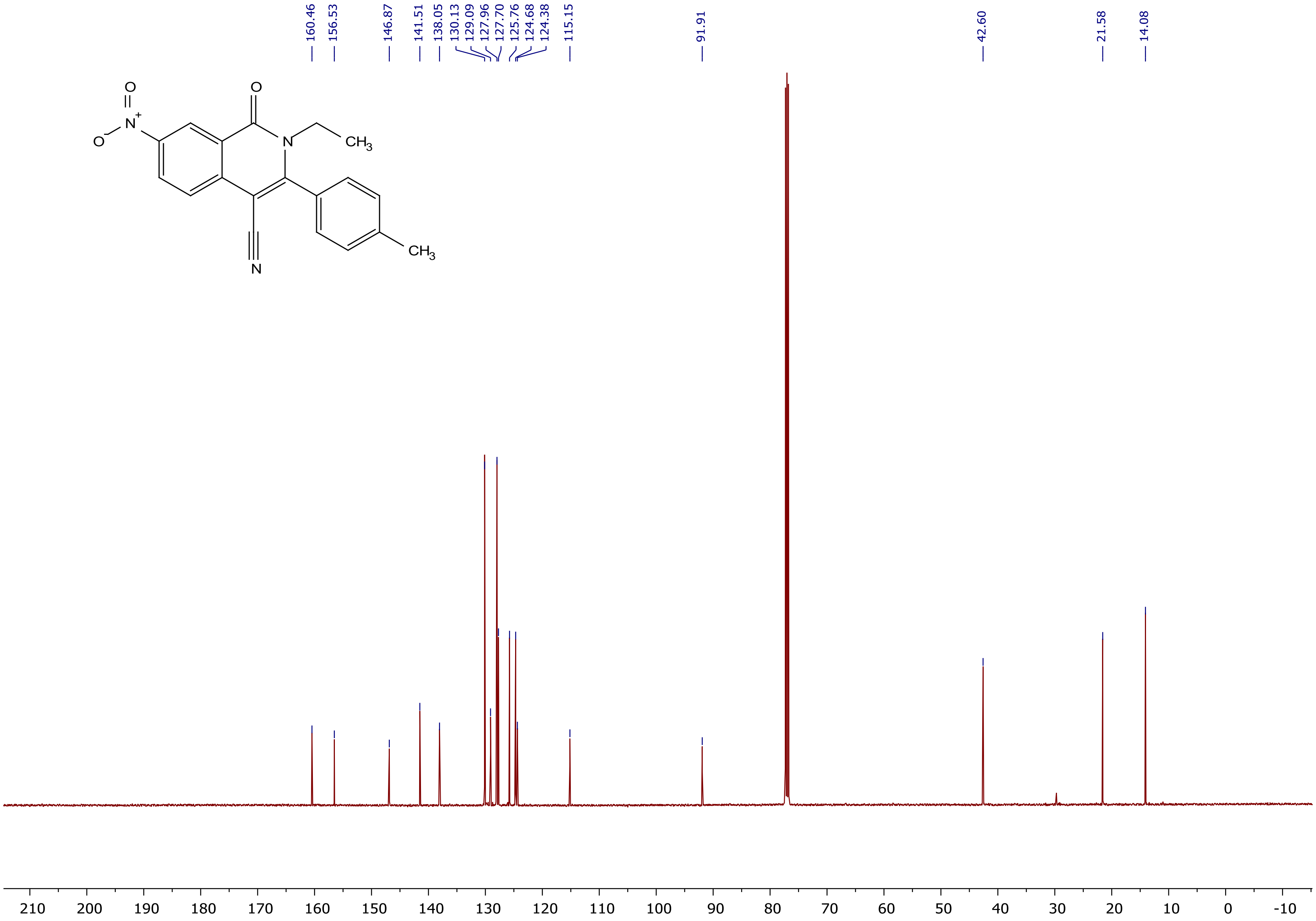

^{1}H NMR spectrum of compound 19b

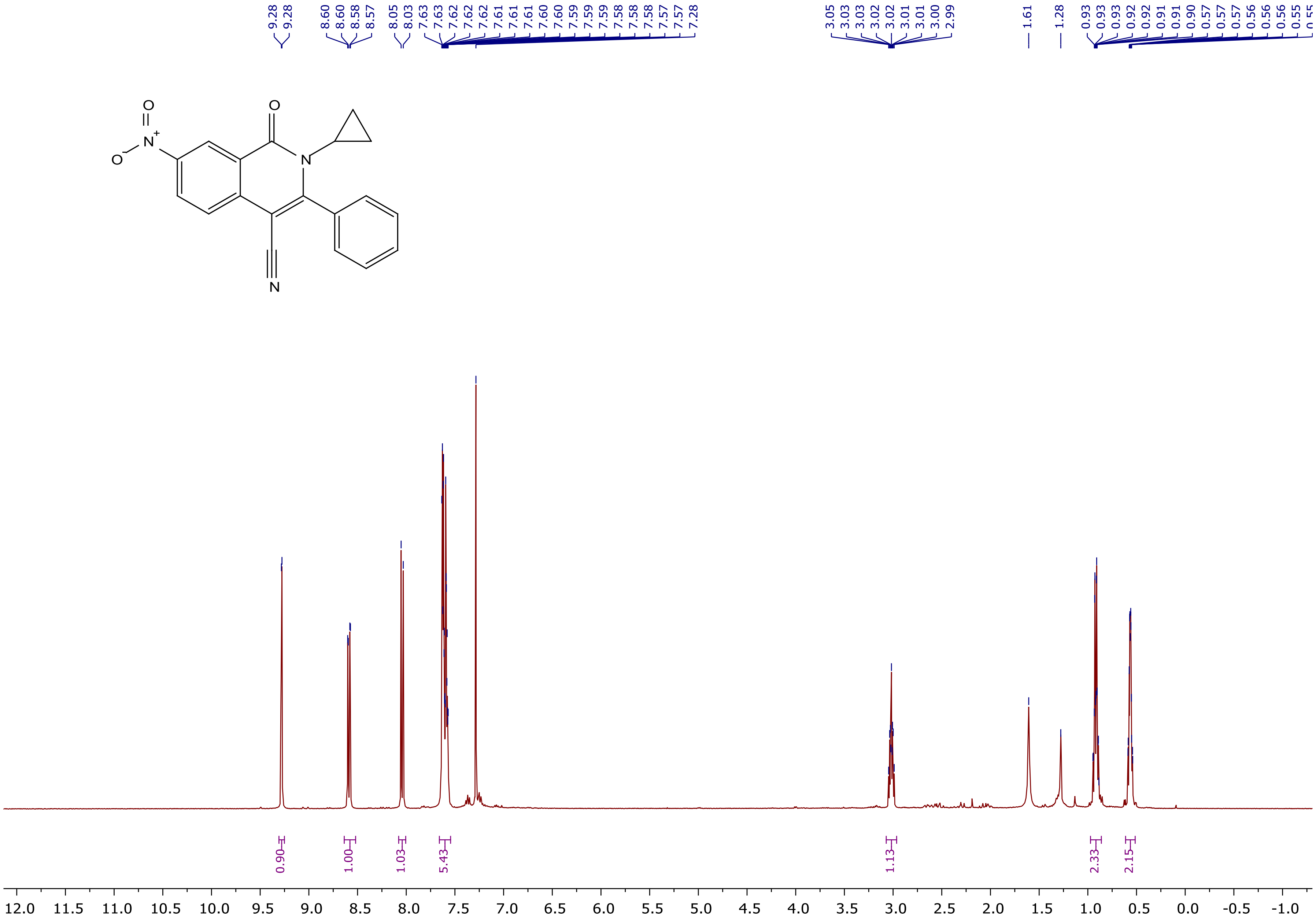

^{13}C NMR spectrum of compound 19b

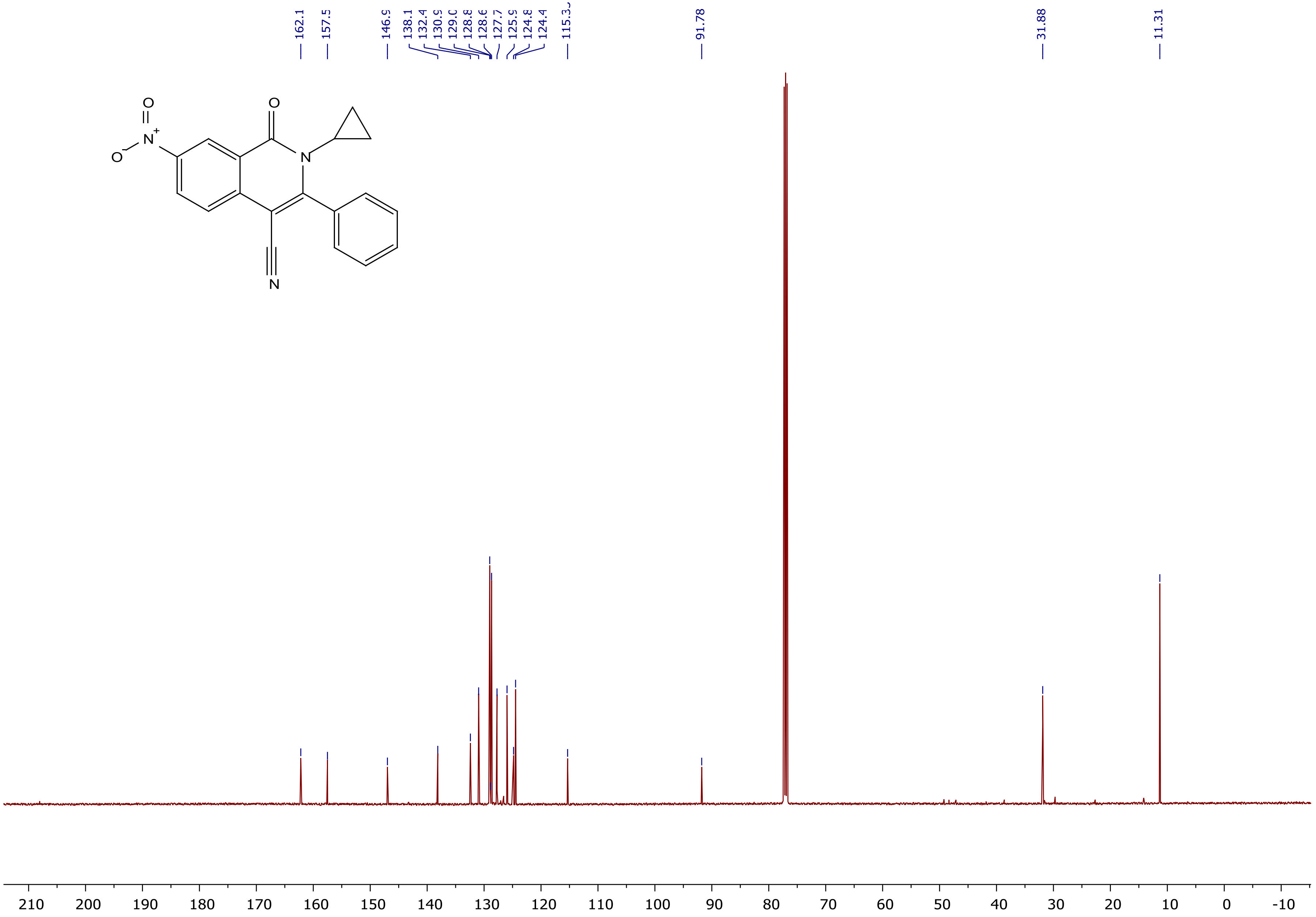

^{1}H NMR spectrum of compound 20

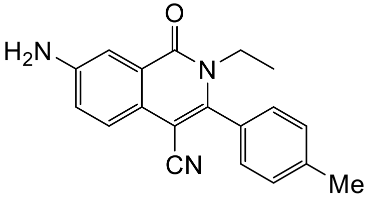

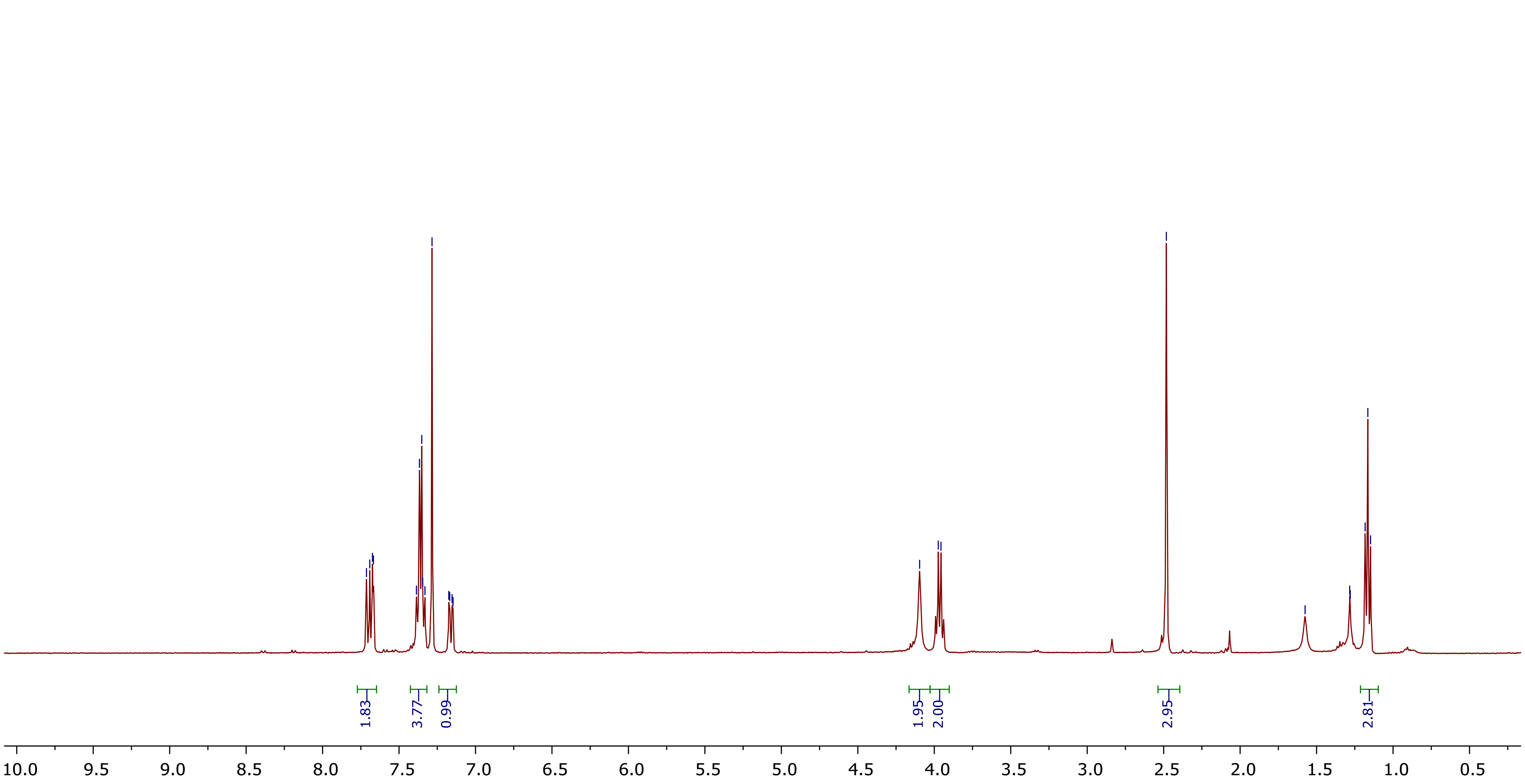

^{13}C NMR spectrum of compound 20

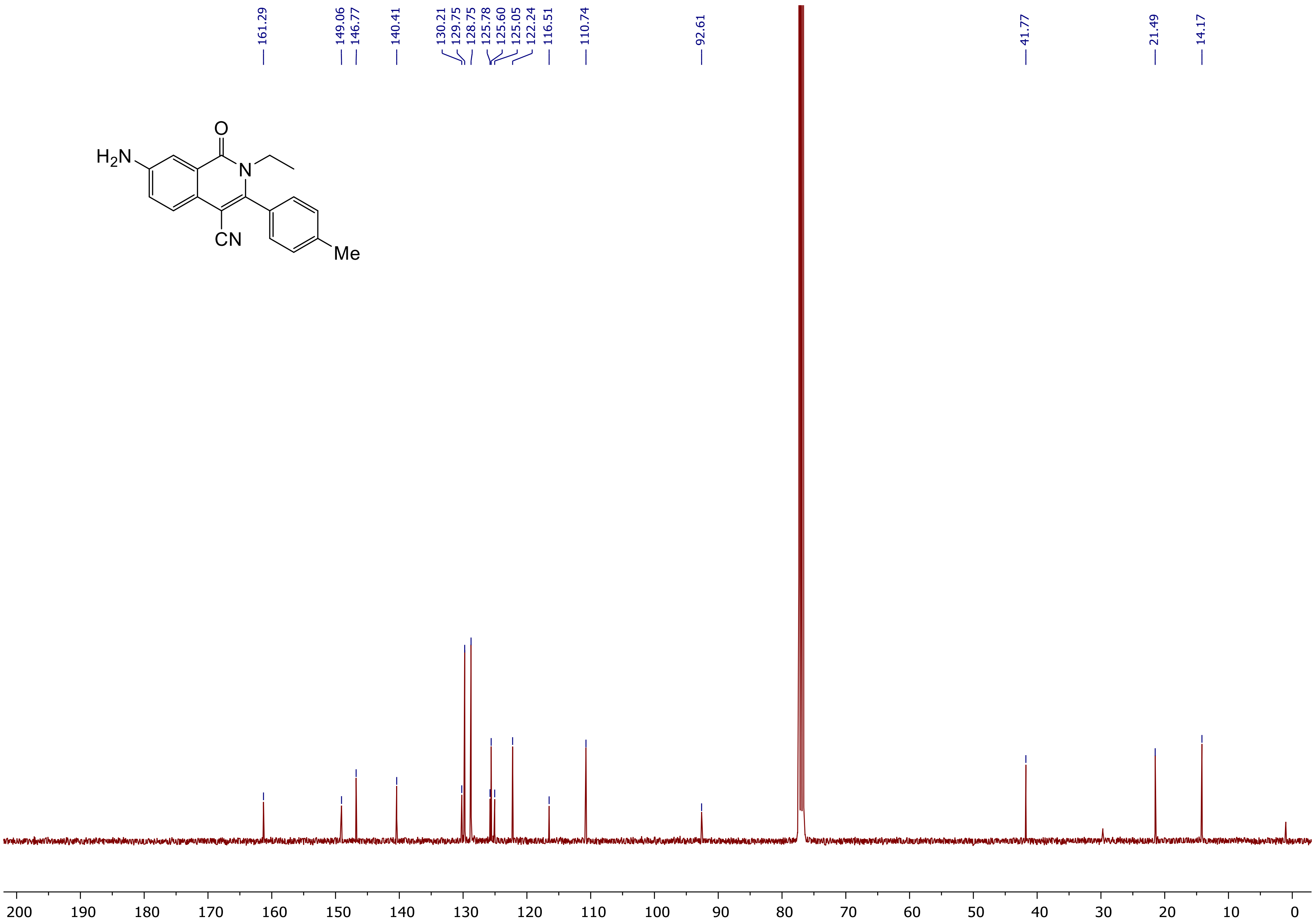

^{1}H NMR spectrum of compound 21

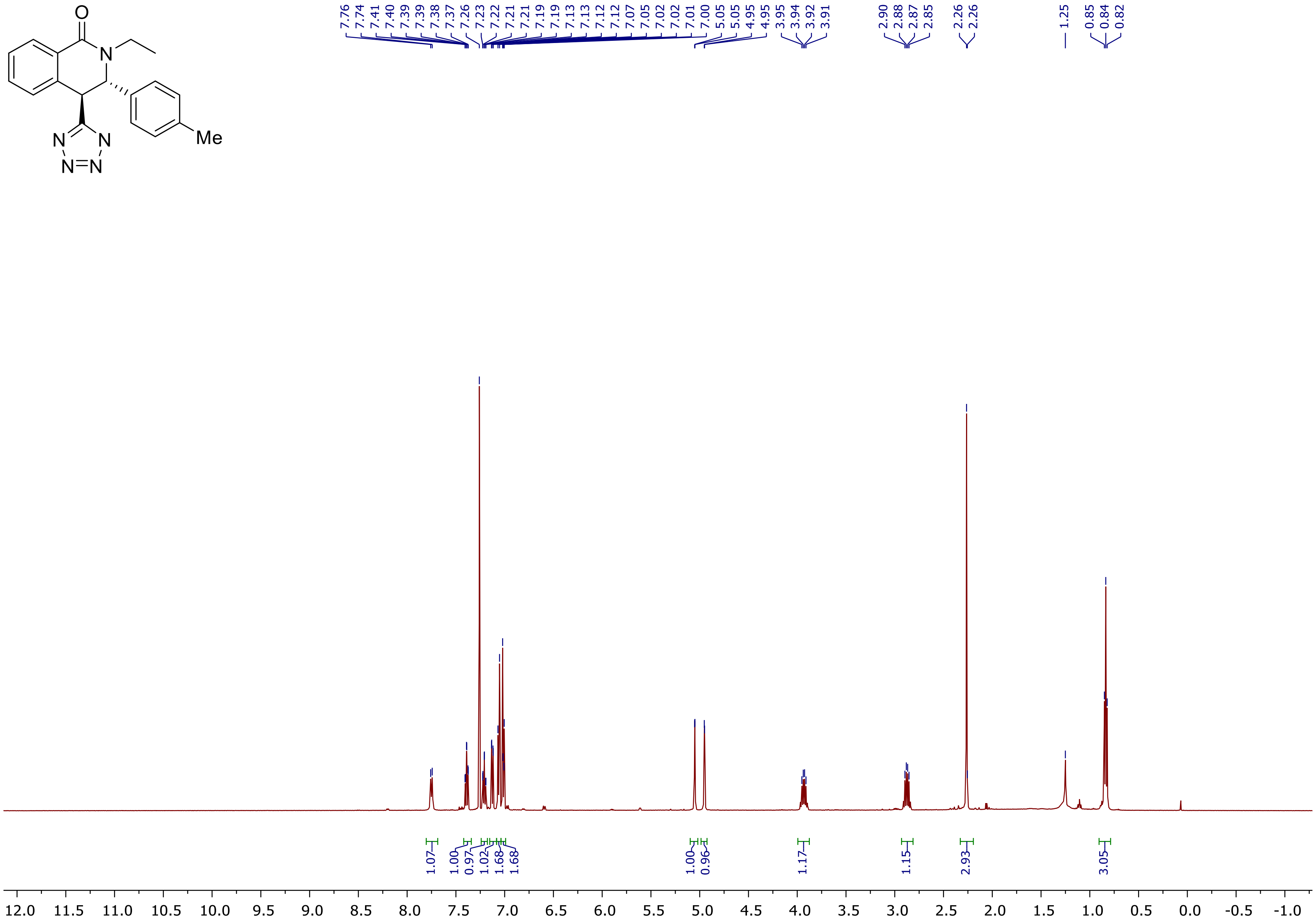

^{13}C NMR spectrum of compound 21

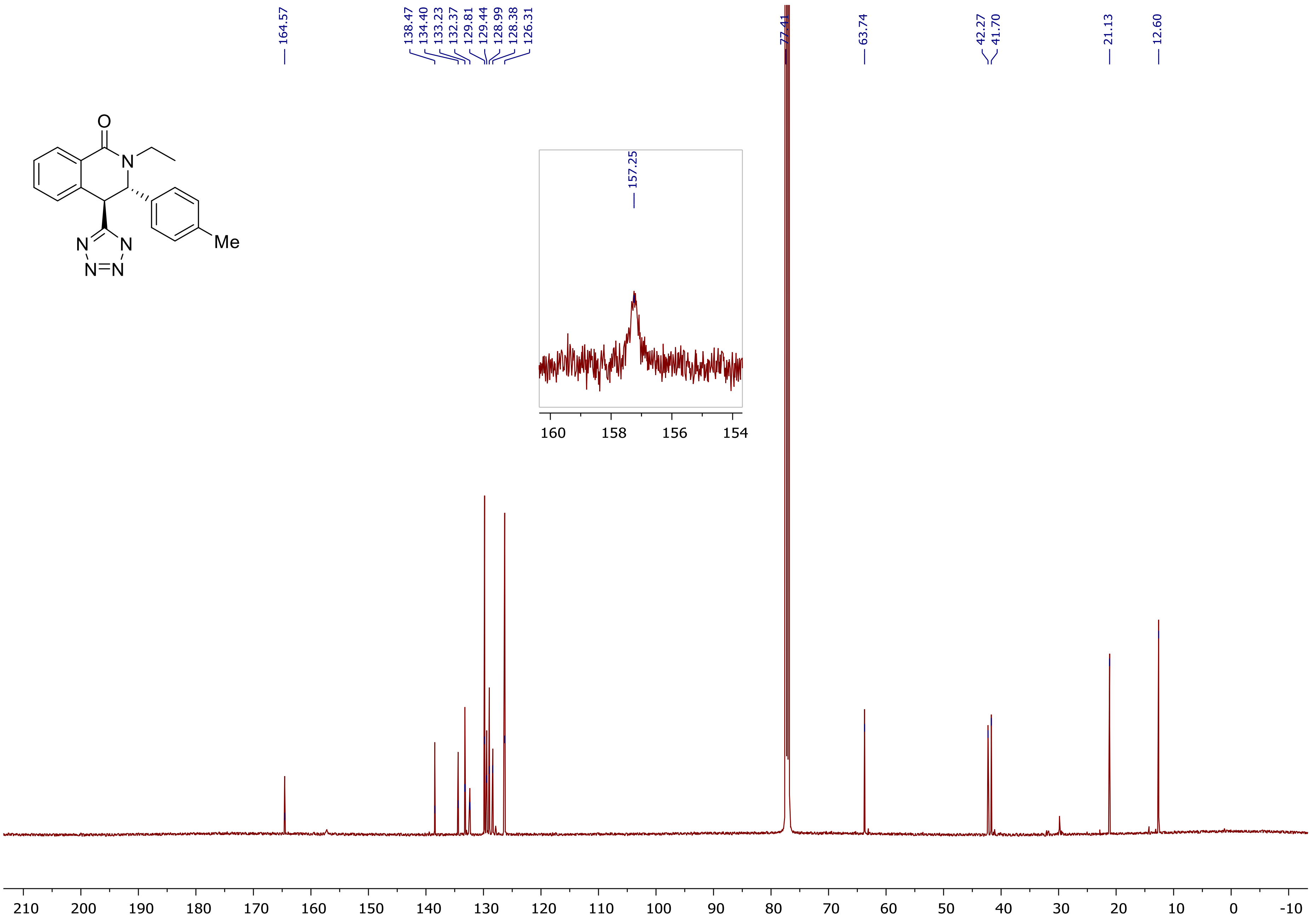

^1H NMR spectrum of compound 22a

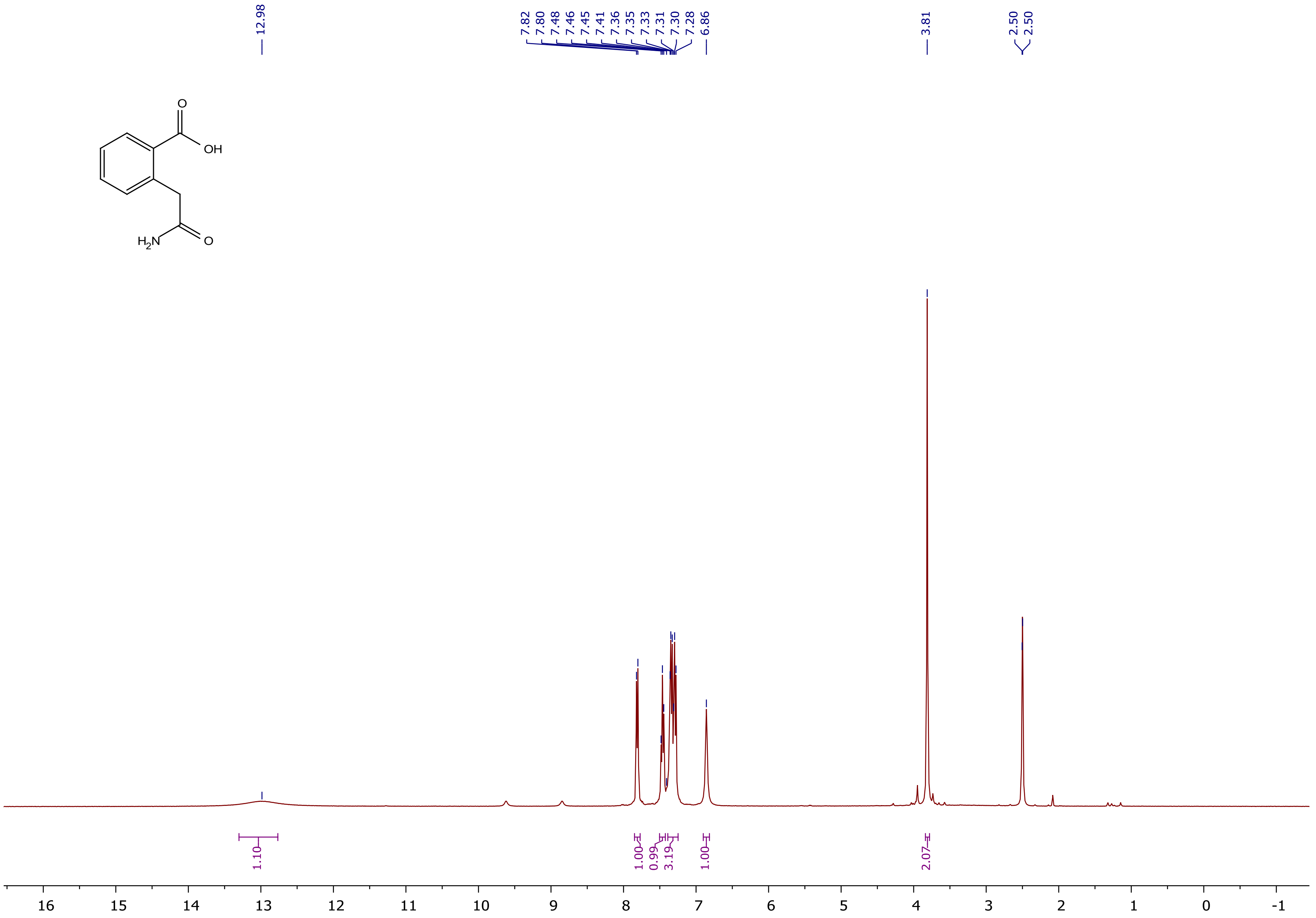

^{13}C NMR spectrum of compound 22a

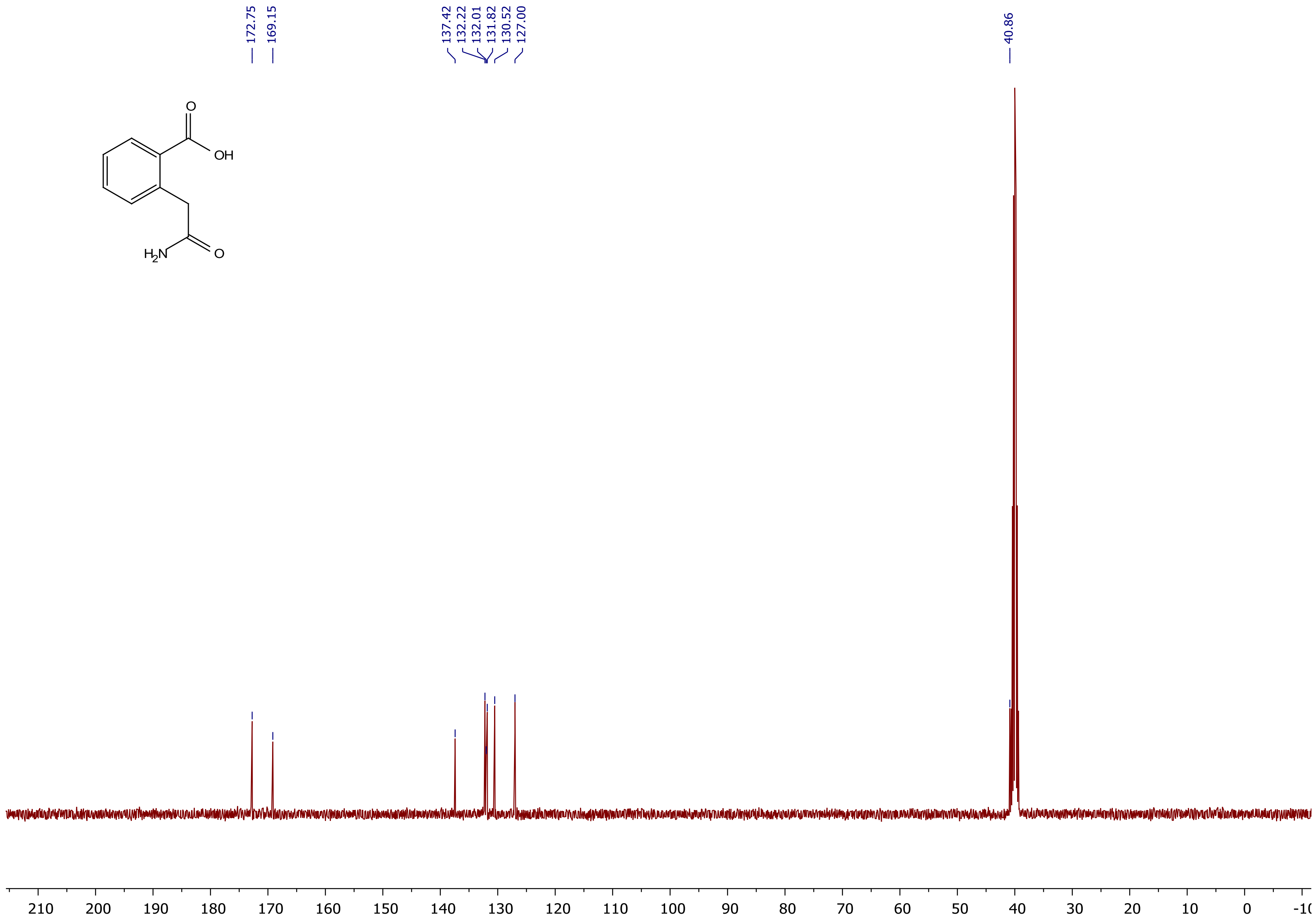

^{1}H NMR spectrum of compound 22b

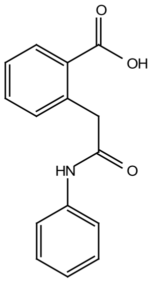

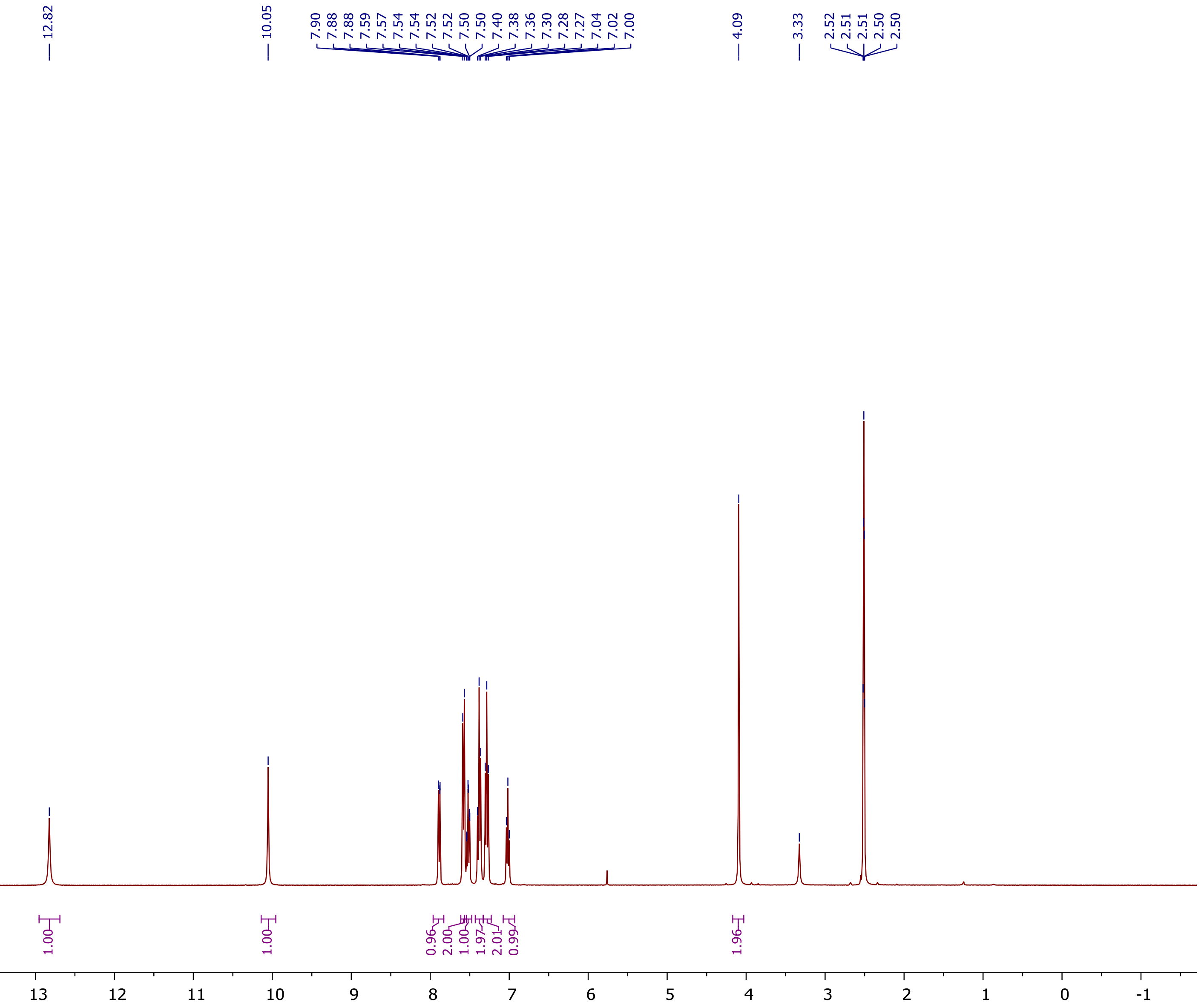

^{13}C NMR spectrum of compound 22b

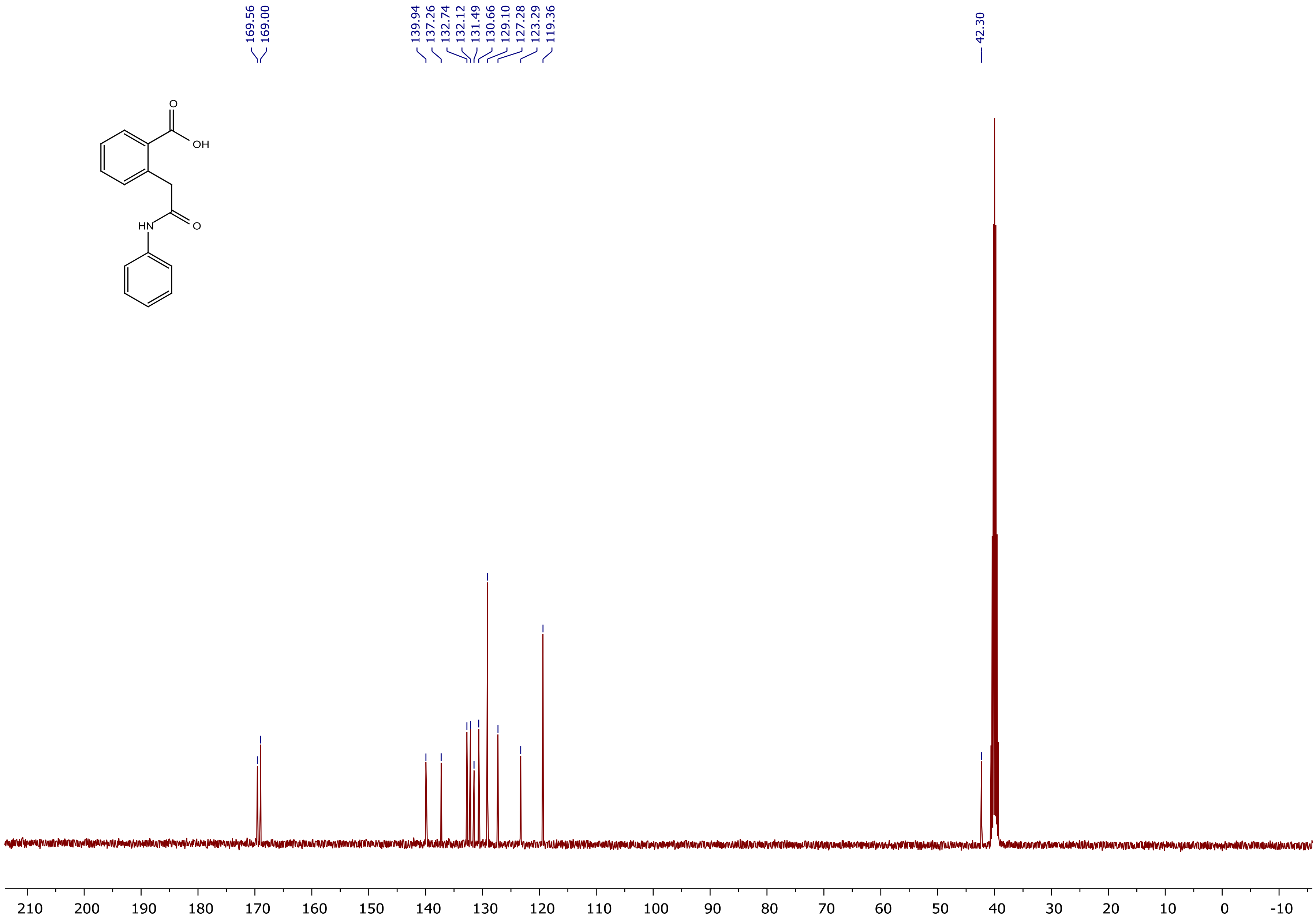

^{1}H NMR spectrum of compound 22c

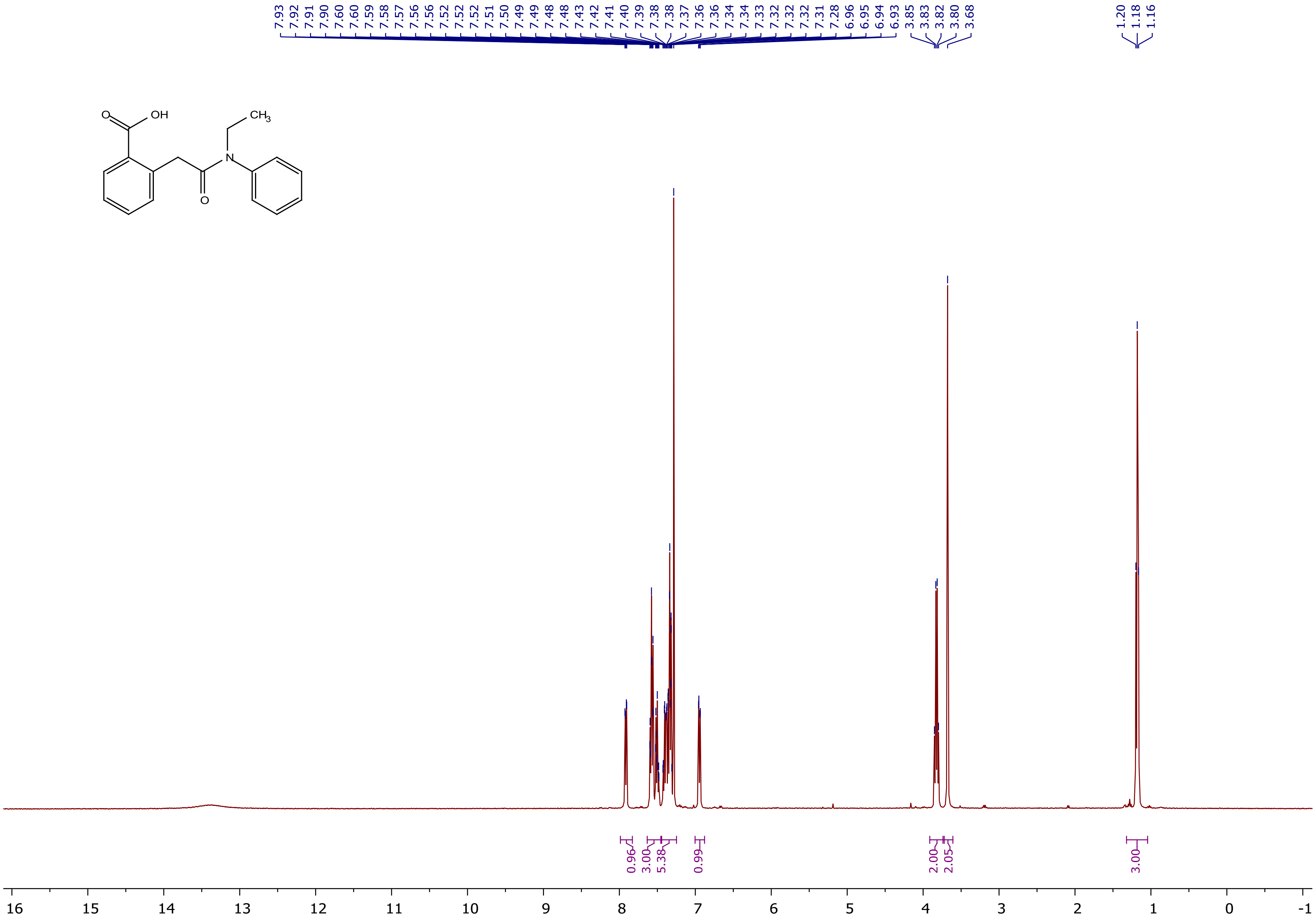

^{13}C NMR spectrum of compound 22c

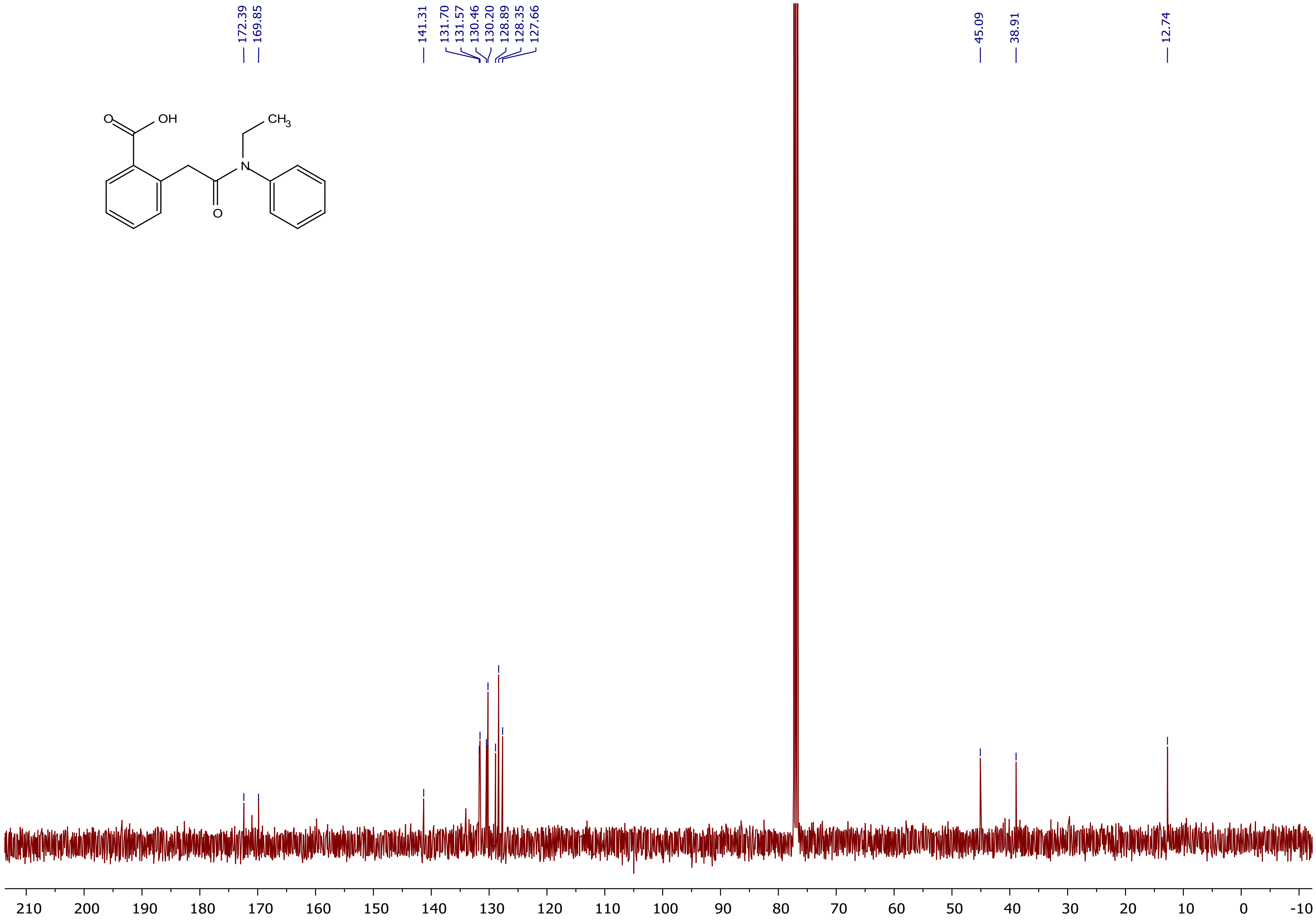

^{1}H NMR spectrum of compound 22d

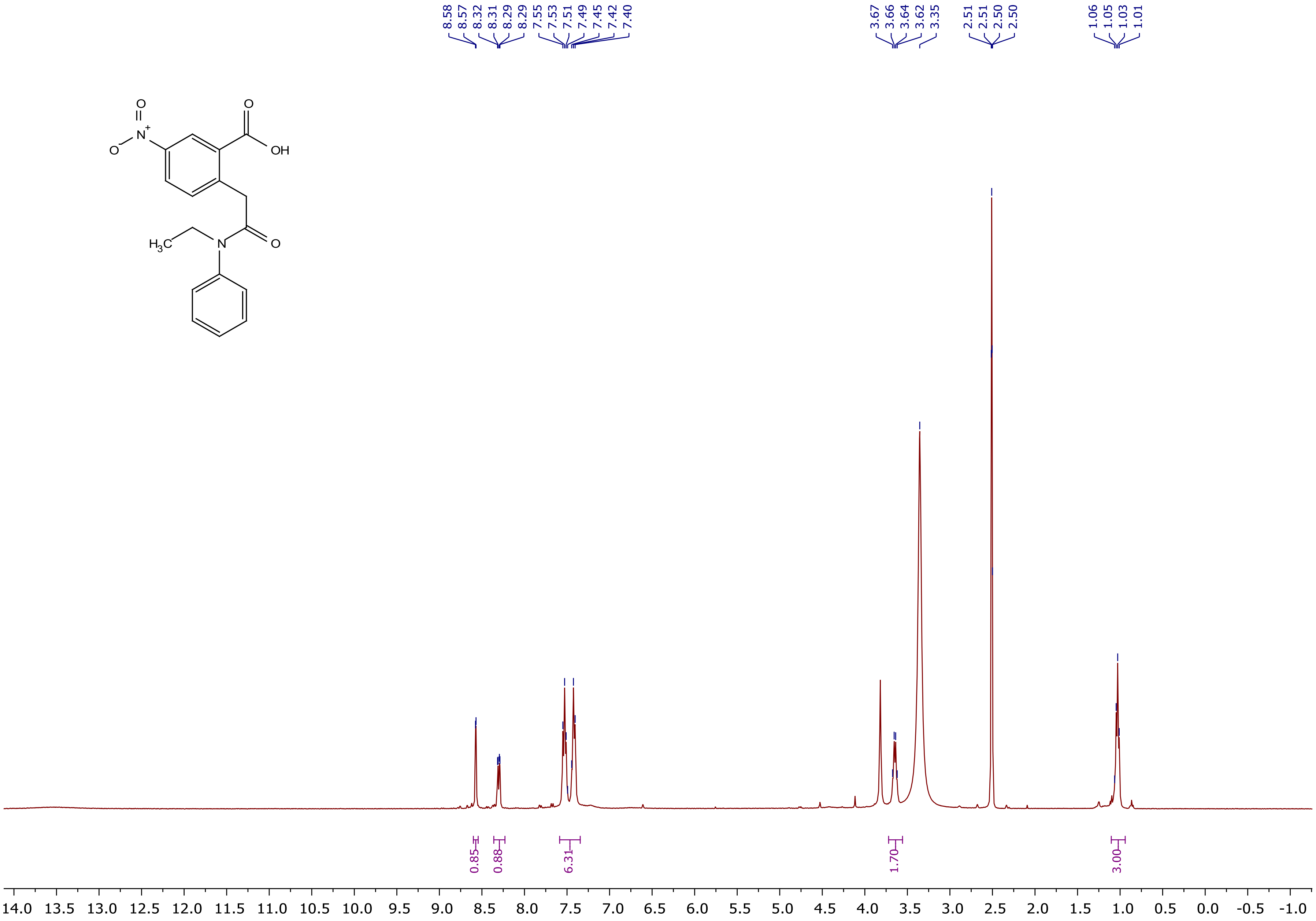

^{13}C NMR spectrum of compound 22d

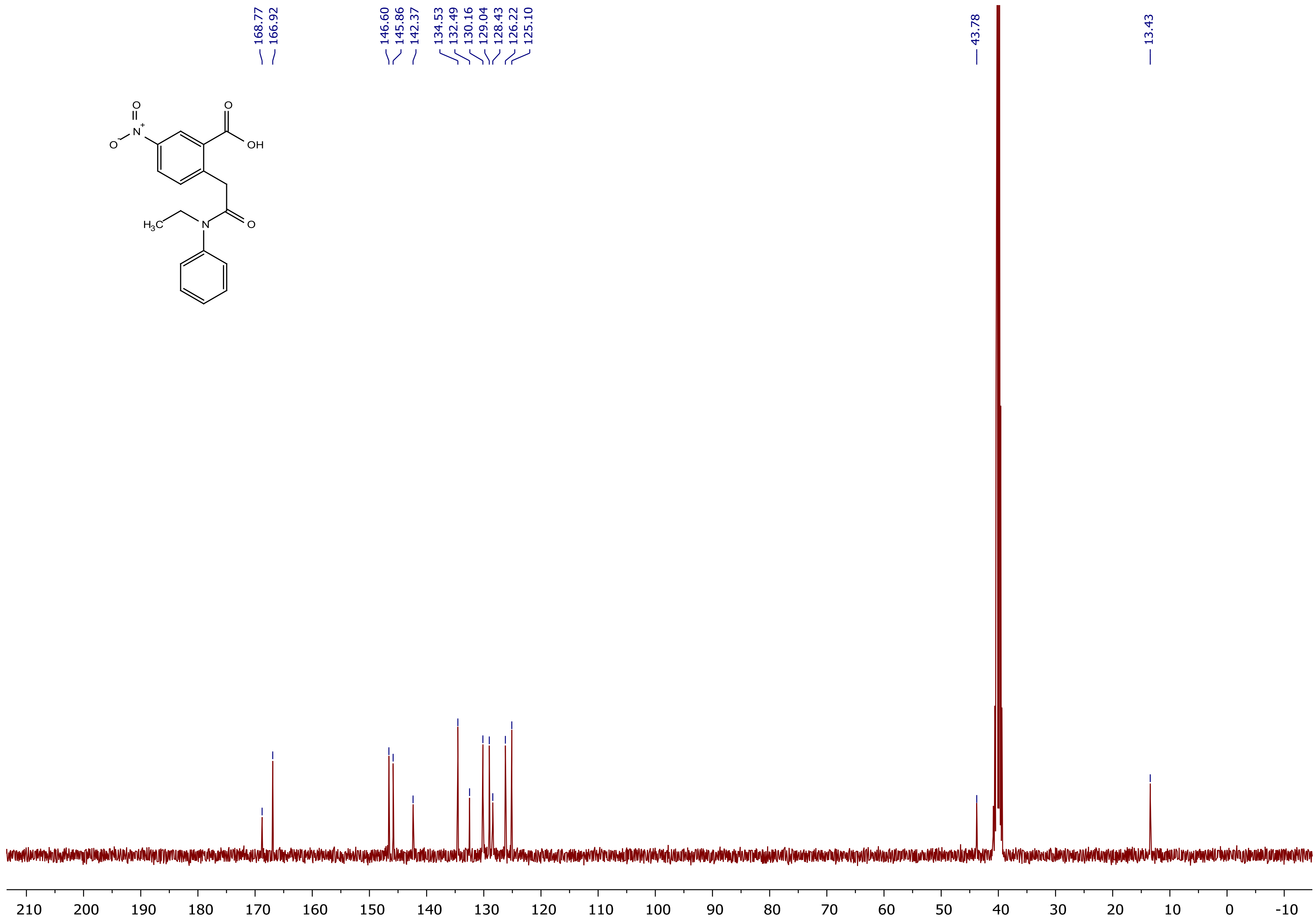

^{1}H NMR spectrum of compound 23a

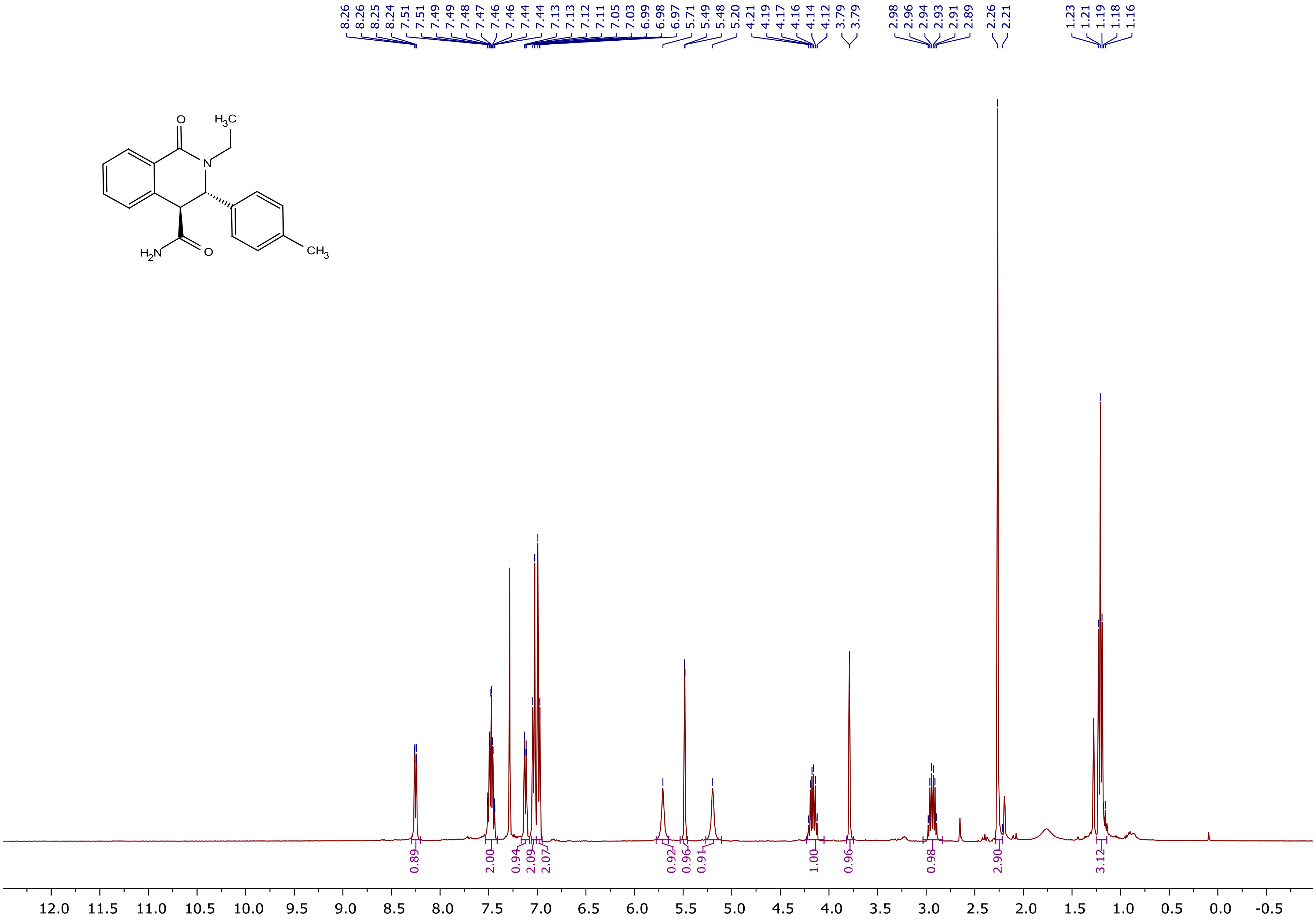

^{13}C NMR spectrum of compound 23a

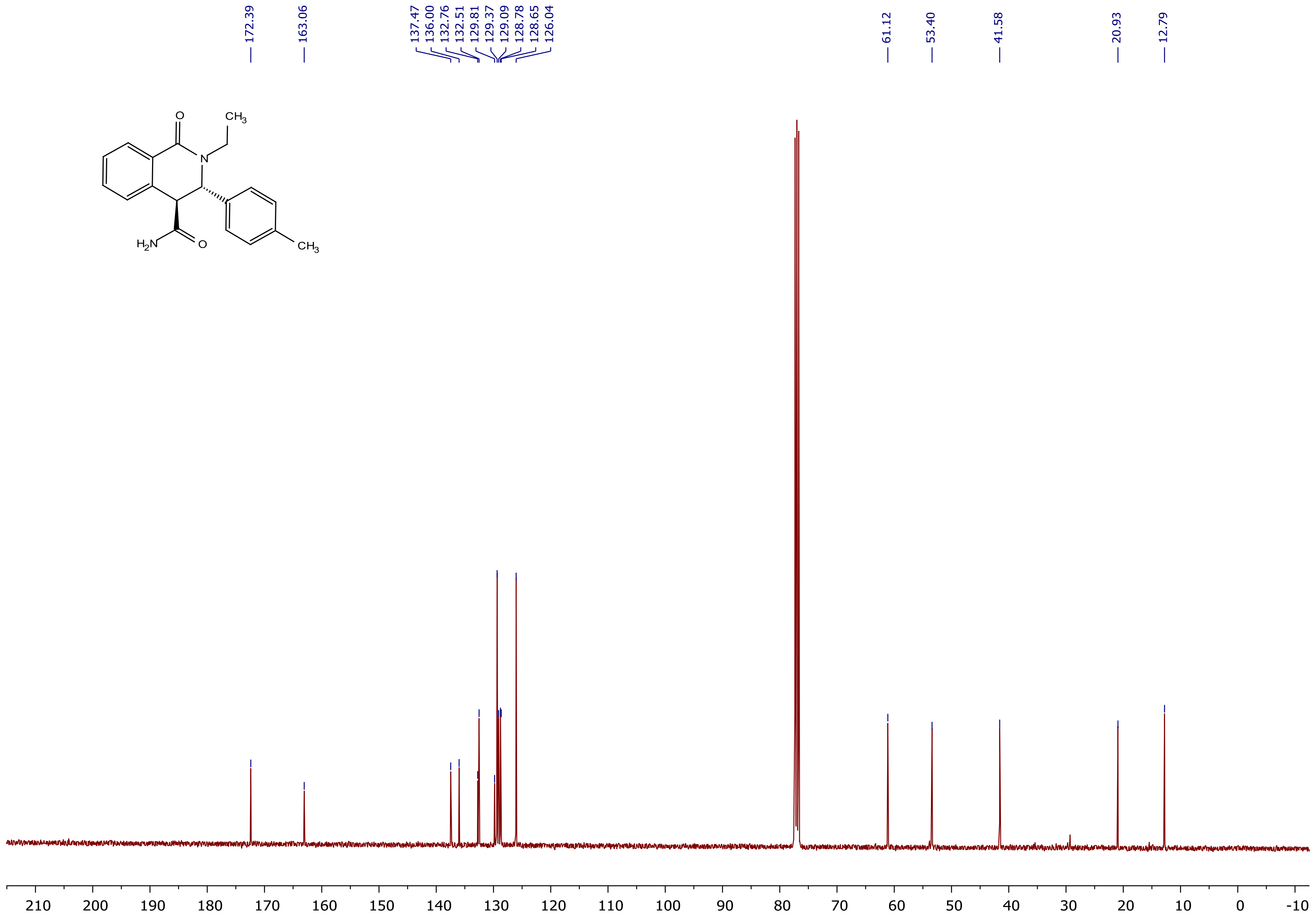

^{1}H NMR spectrum of compound 23b

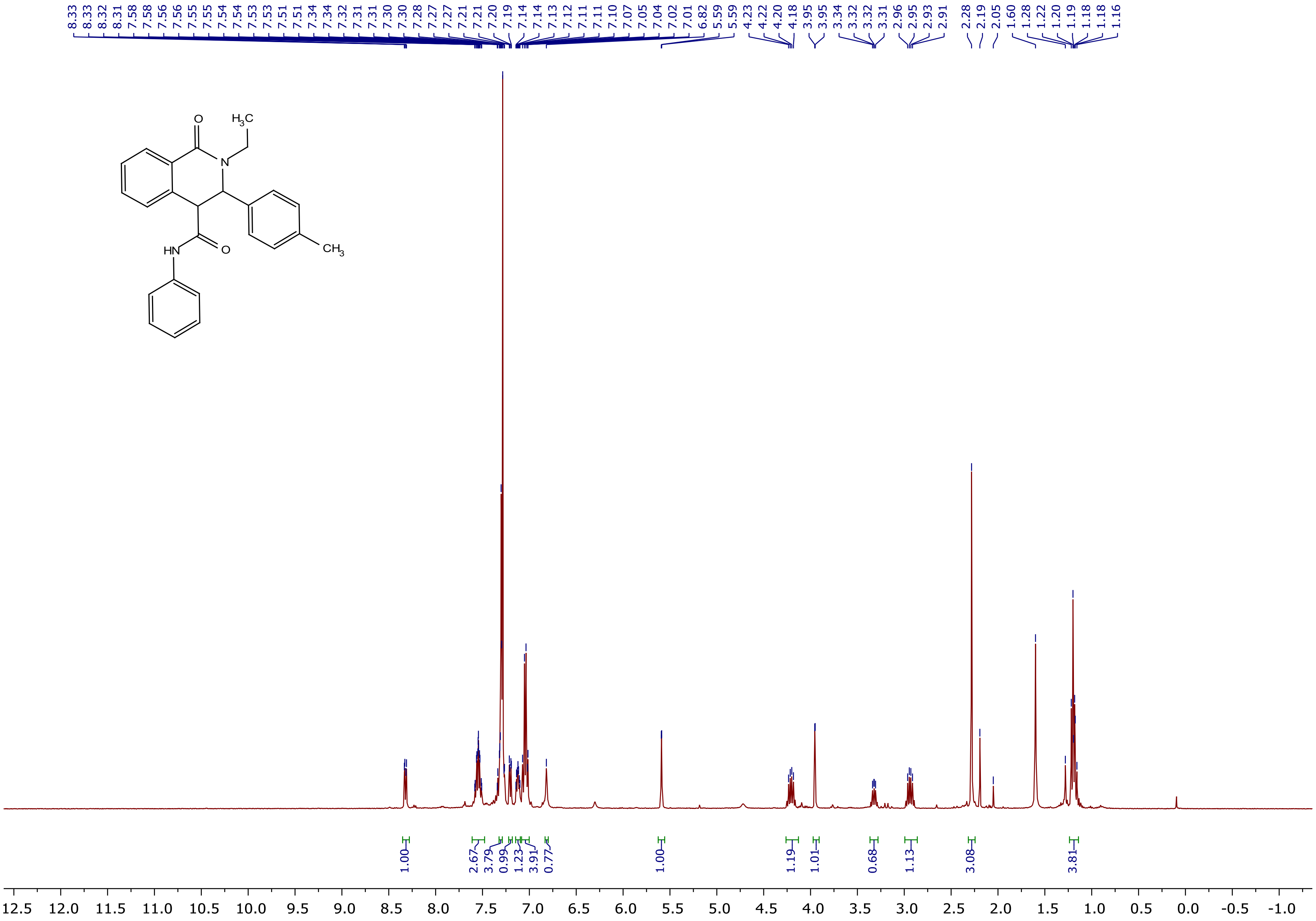

^{13}C NMR spectrum of compound 23b

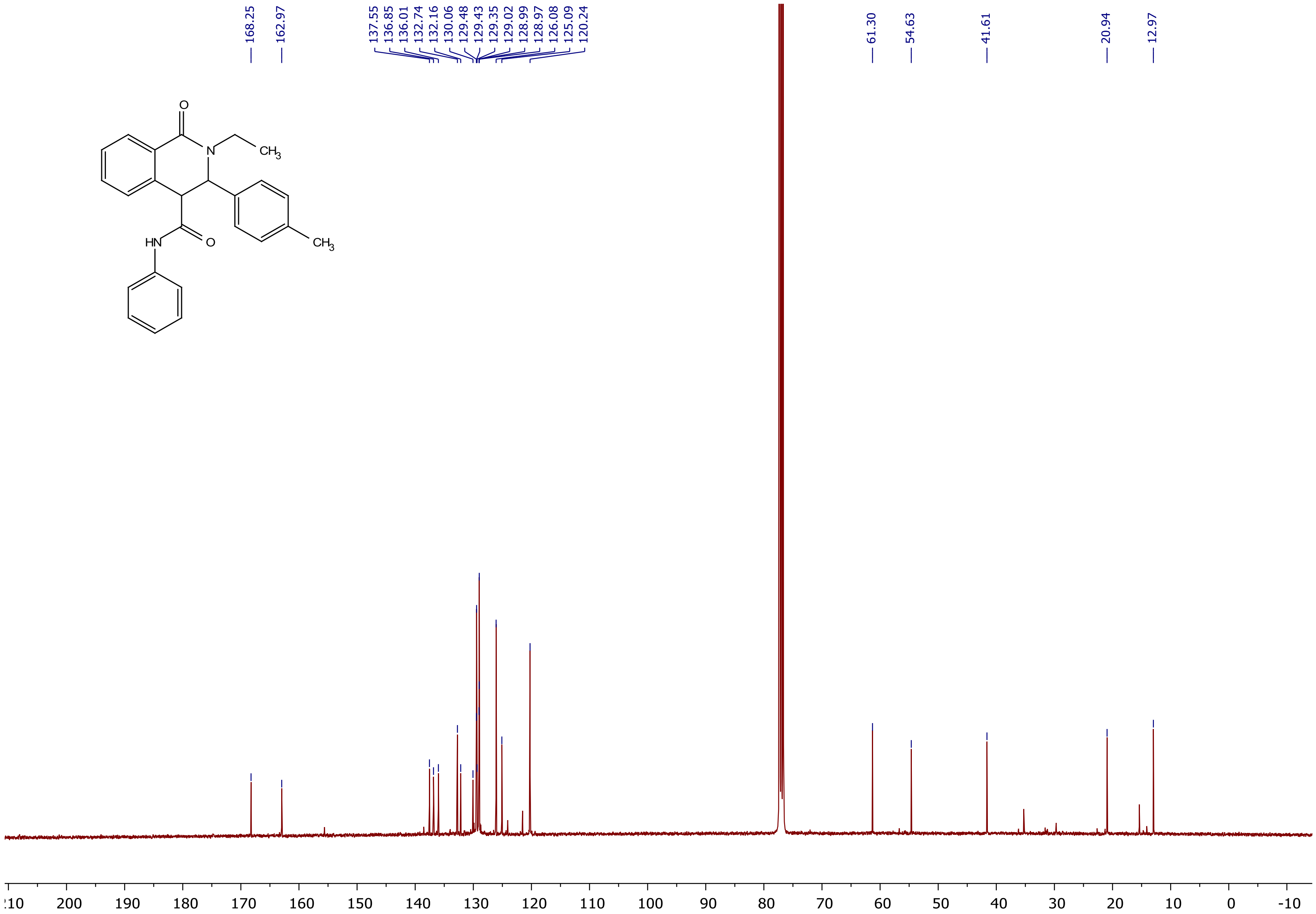

^{1}H NMR spectrum of compound 24a

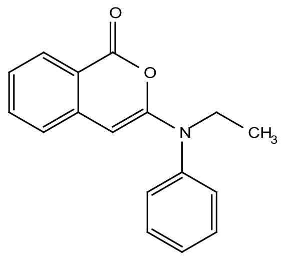

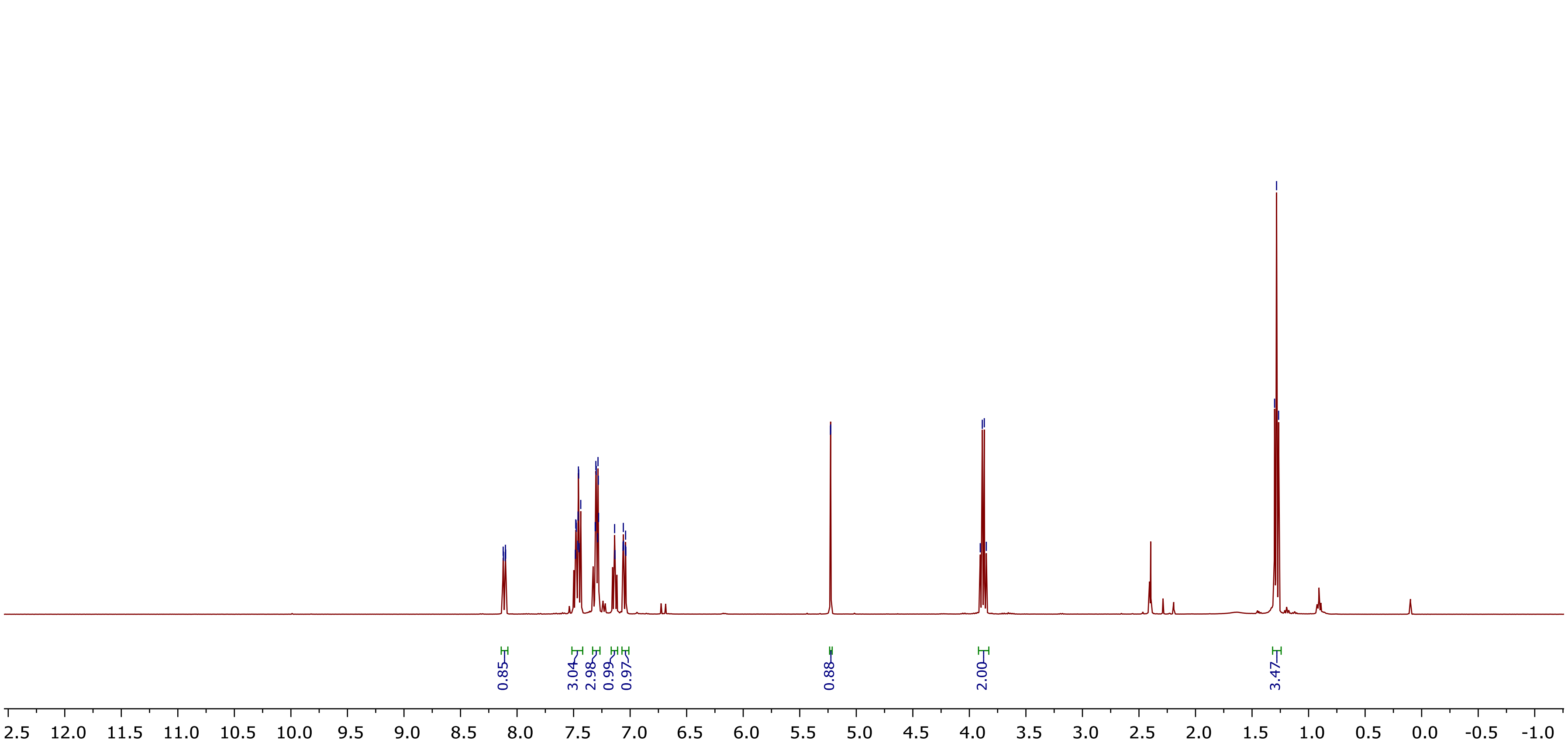

^{13}C NMR spectrum of compound 24a

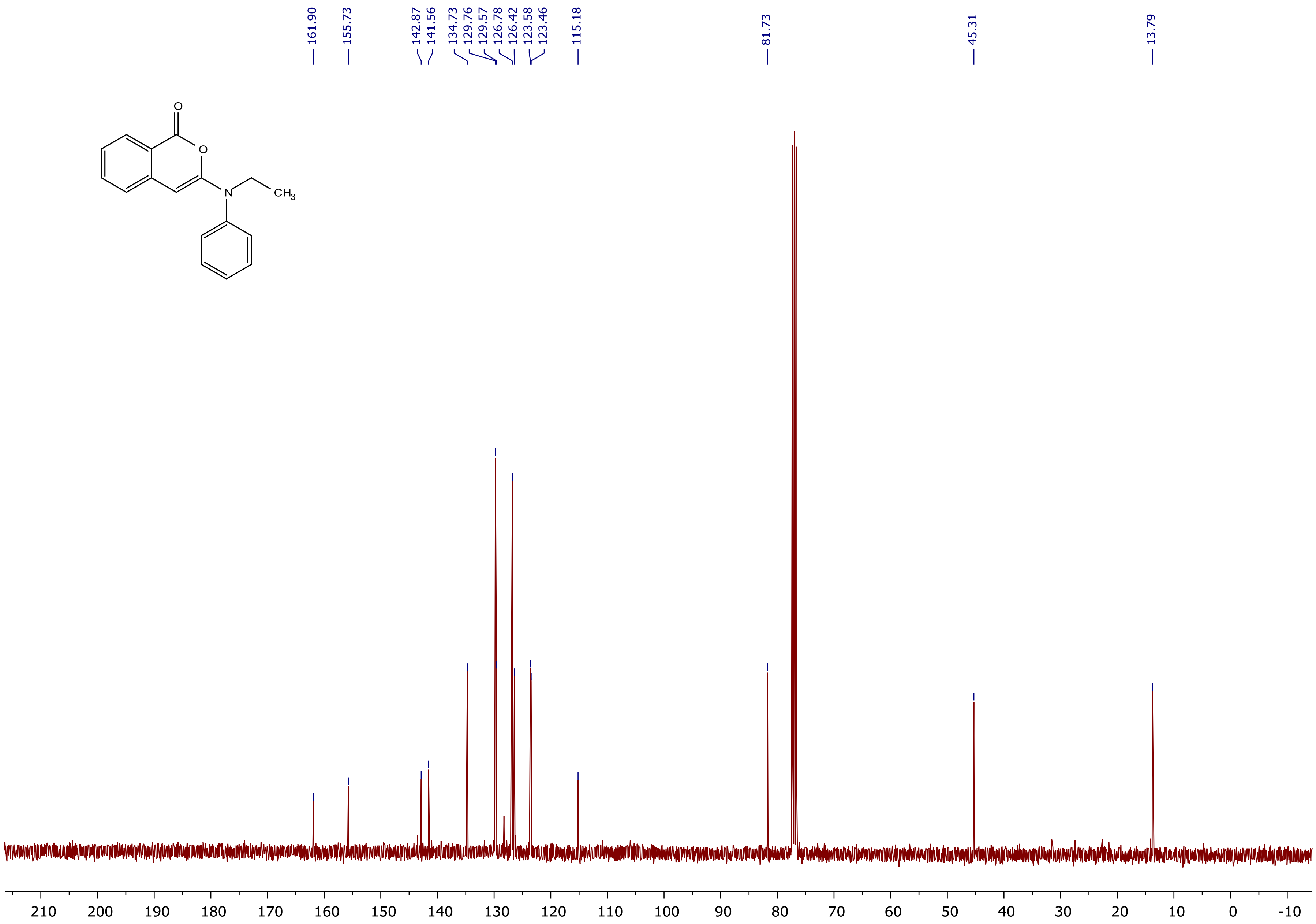

^{1}H NMR spectrum of compound 24b

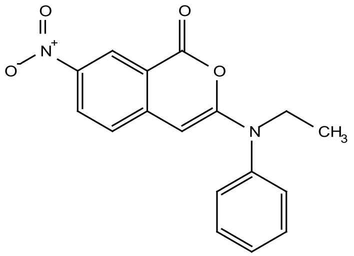

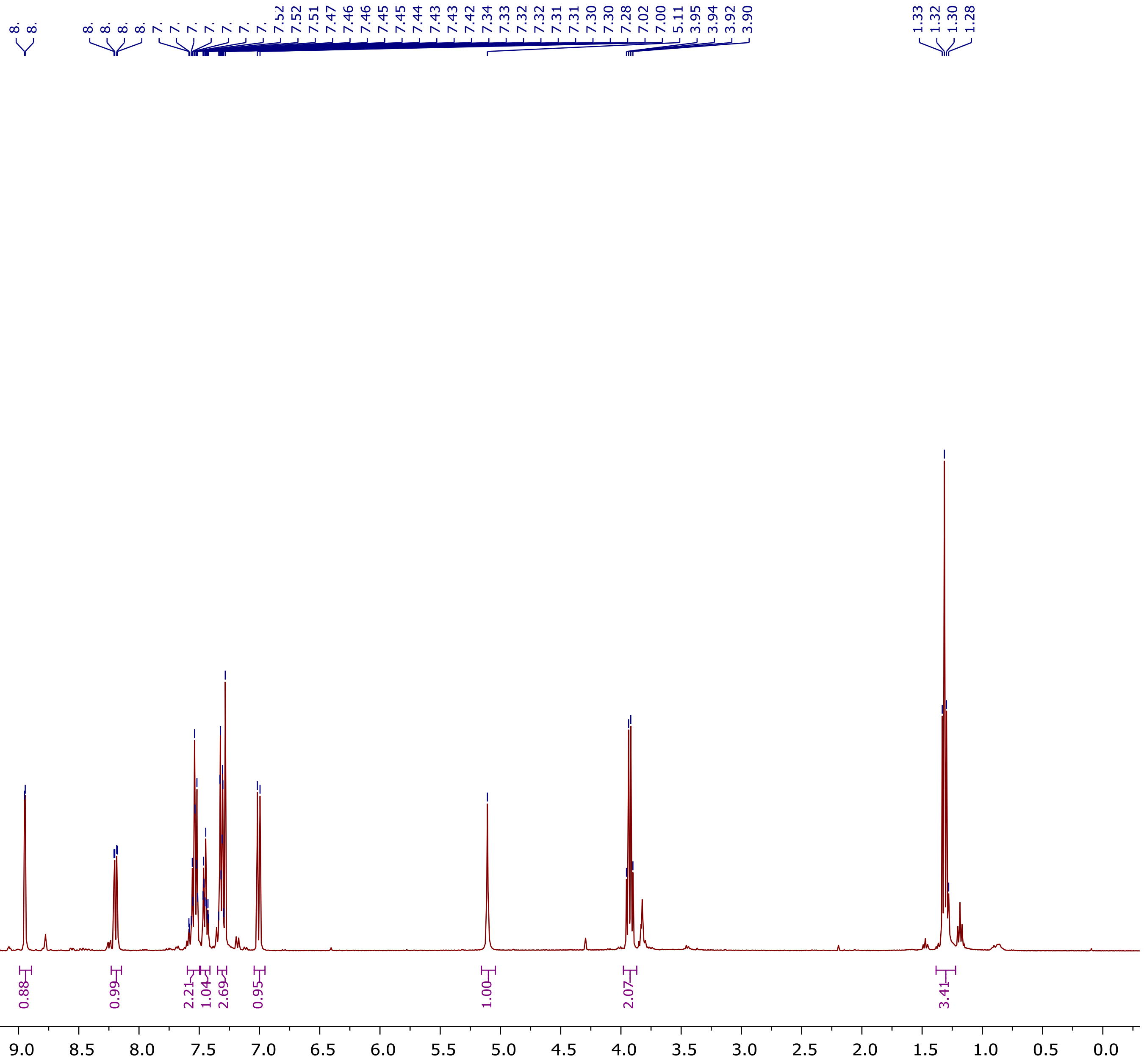

^{13}C NMR spectrum of compound 24b

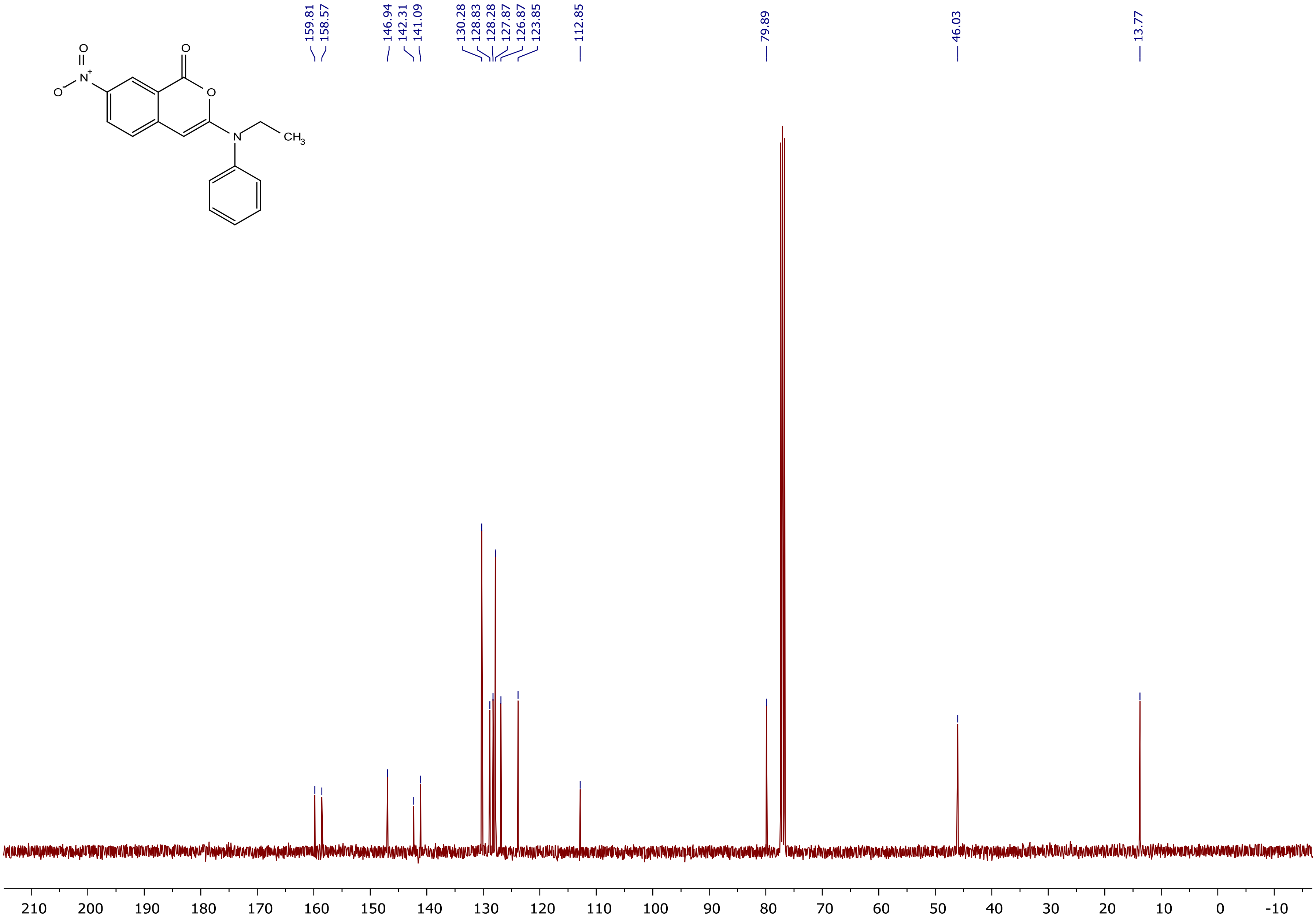

^{1}H NMR spectrum of compound 26

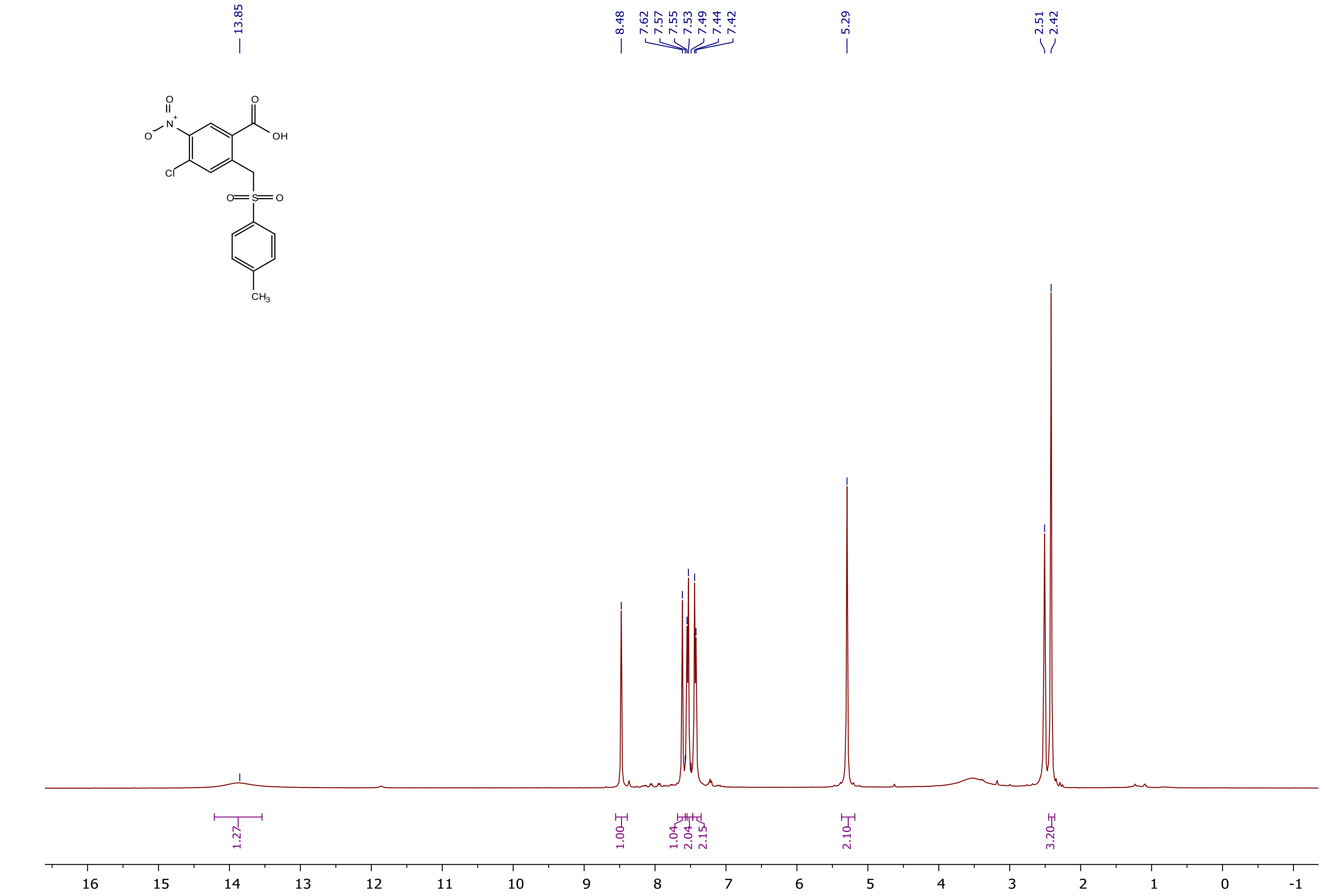

^{13}C NMR spectrum of compound 26

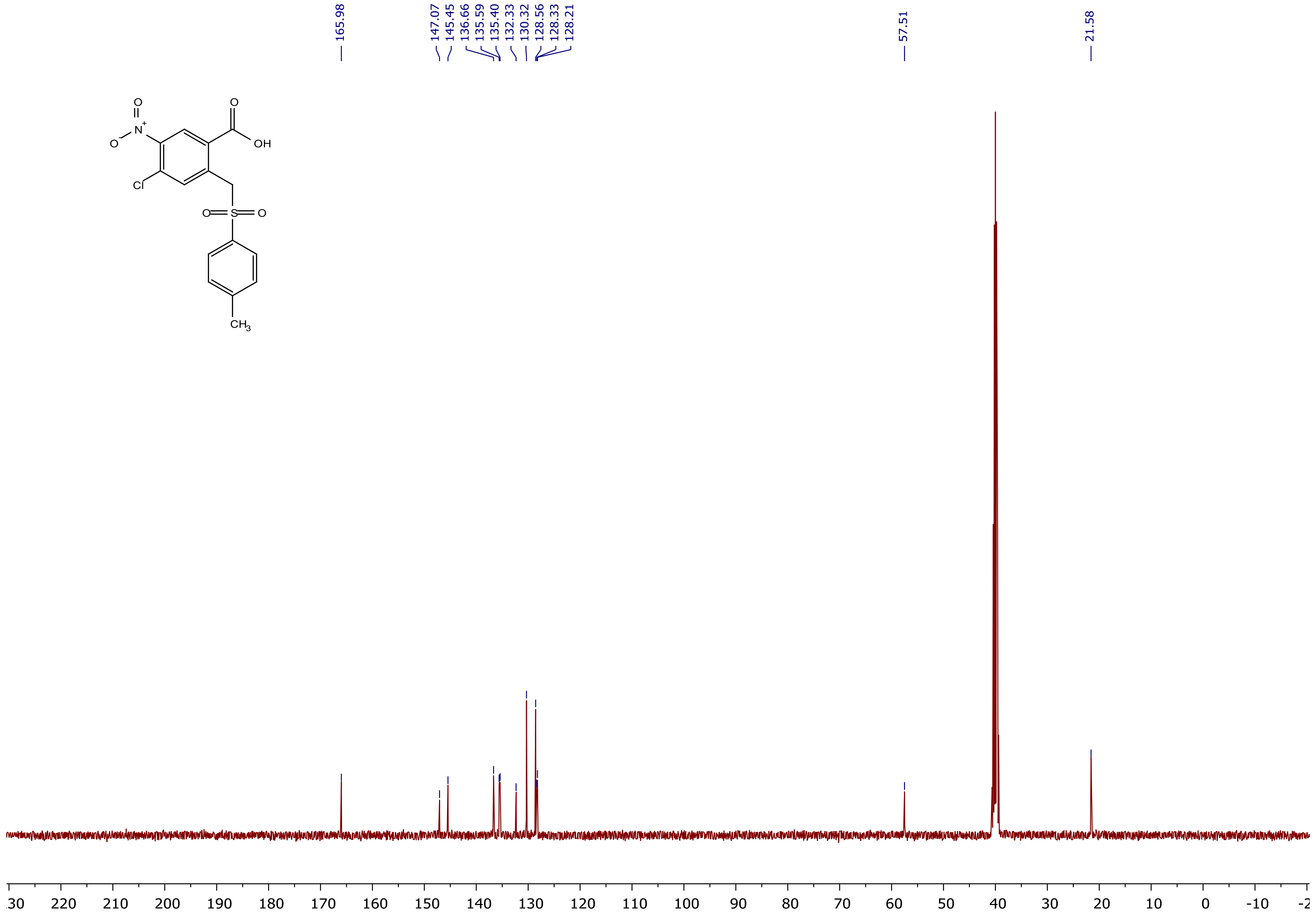

^{13}C NMR spectrum of compound 12a

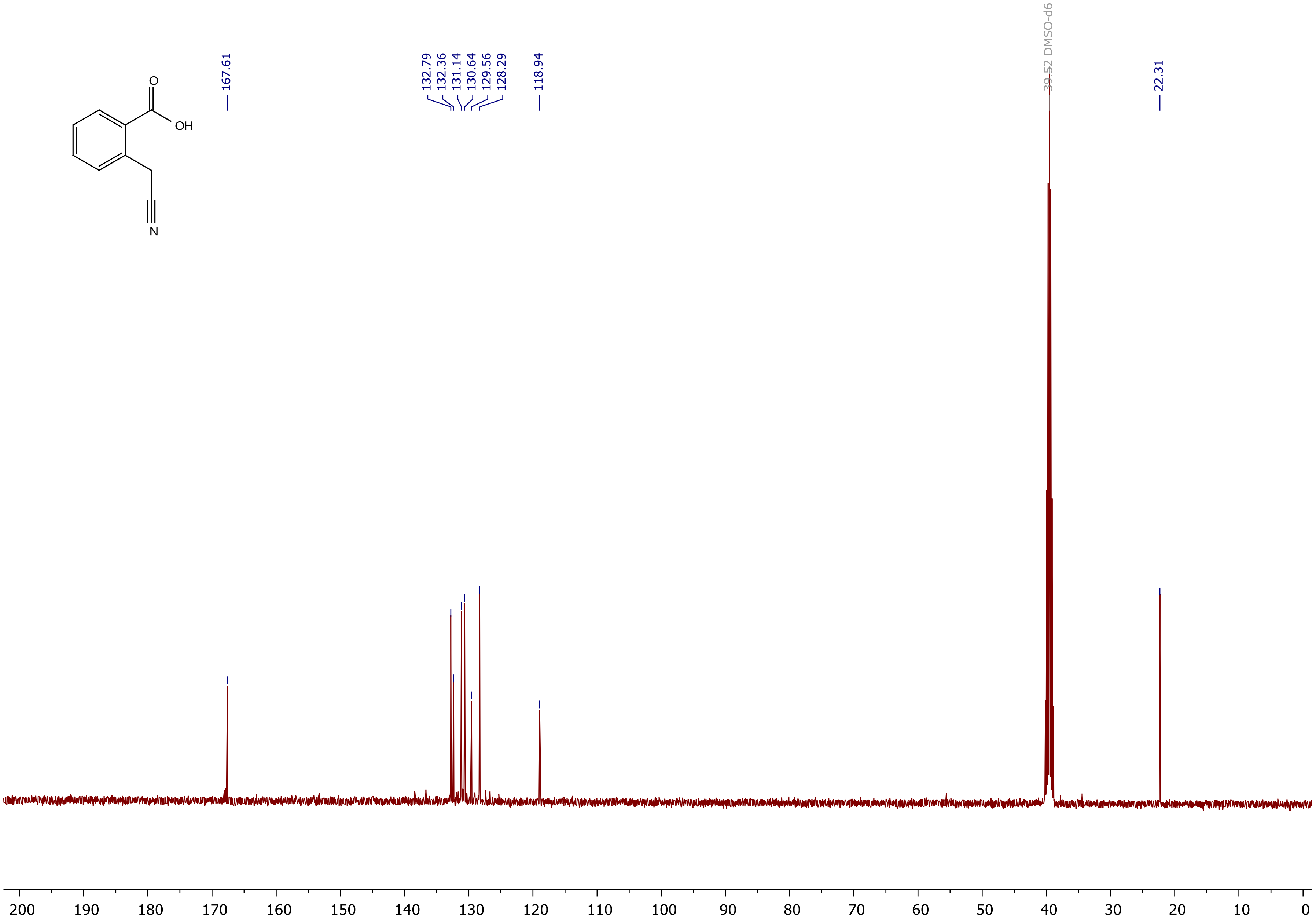

^{1}H NMR spectrum of compound 12b

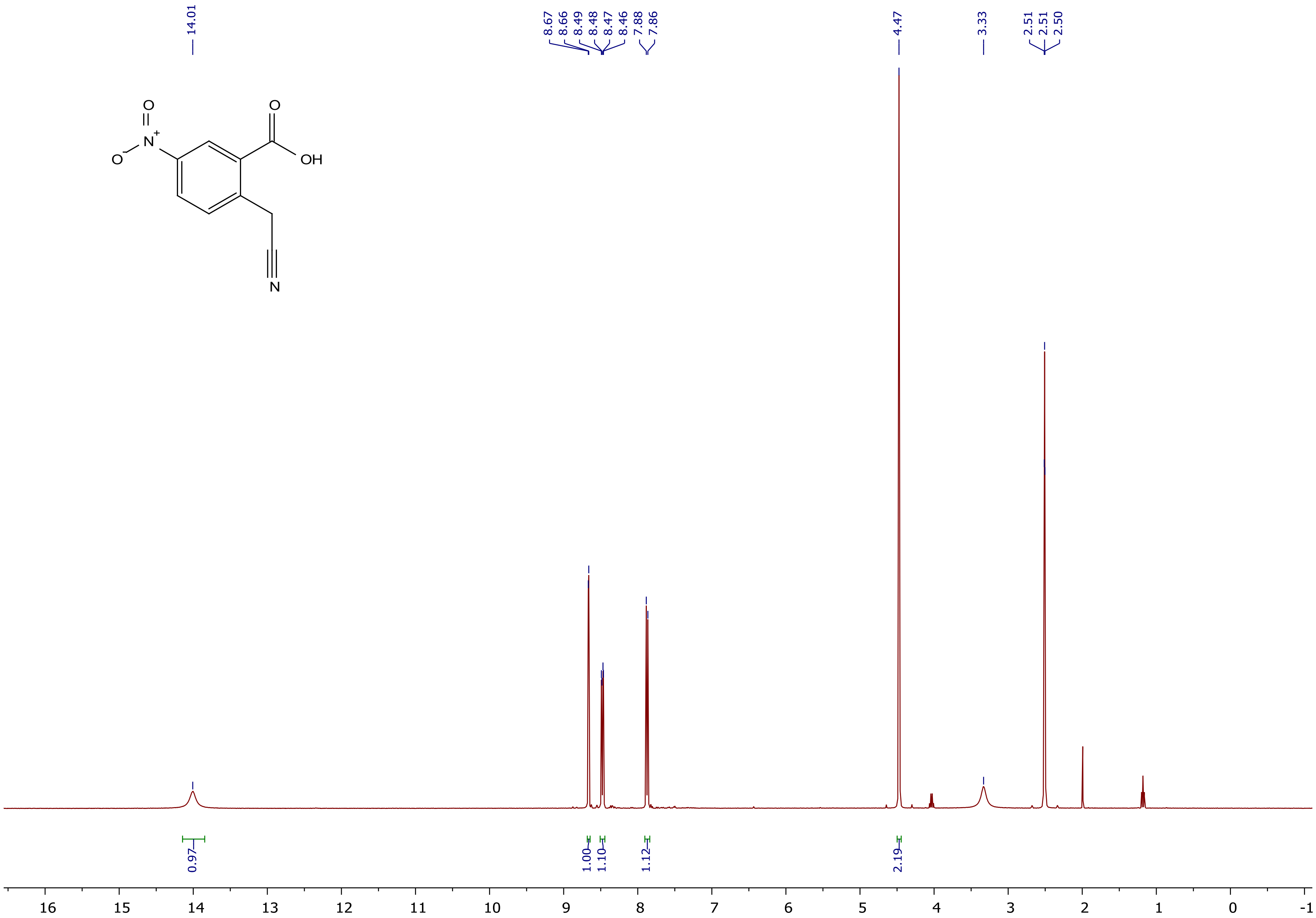

^{13}C NMR spectrum of compound 12b

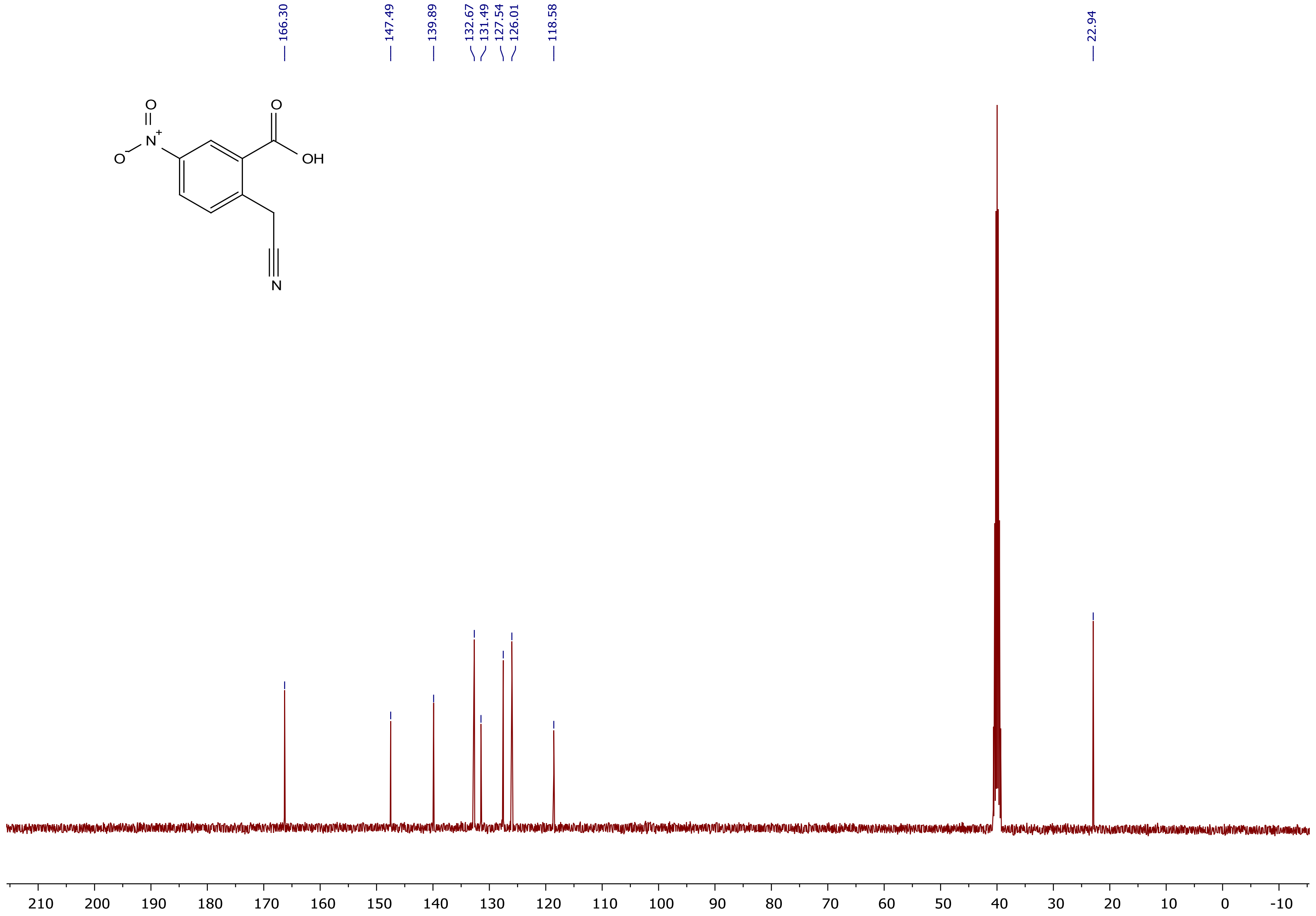

^{1}H NMR spectrum of compound 12c

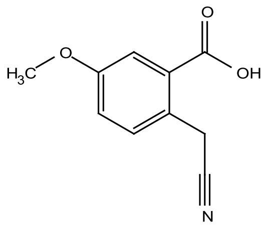

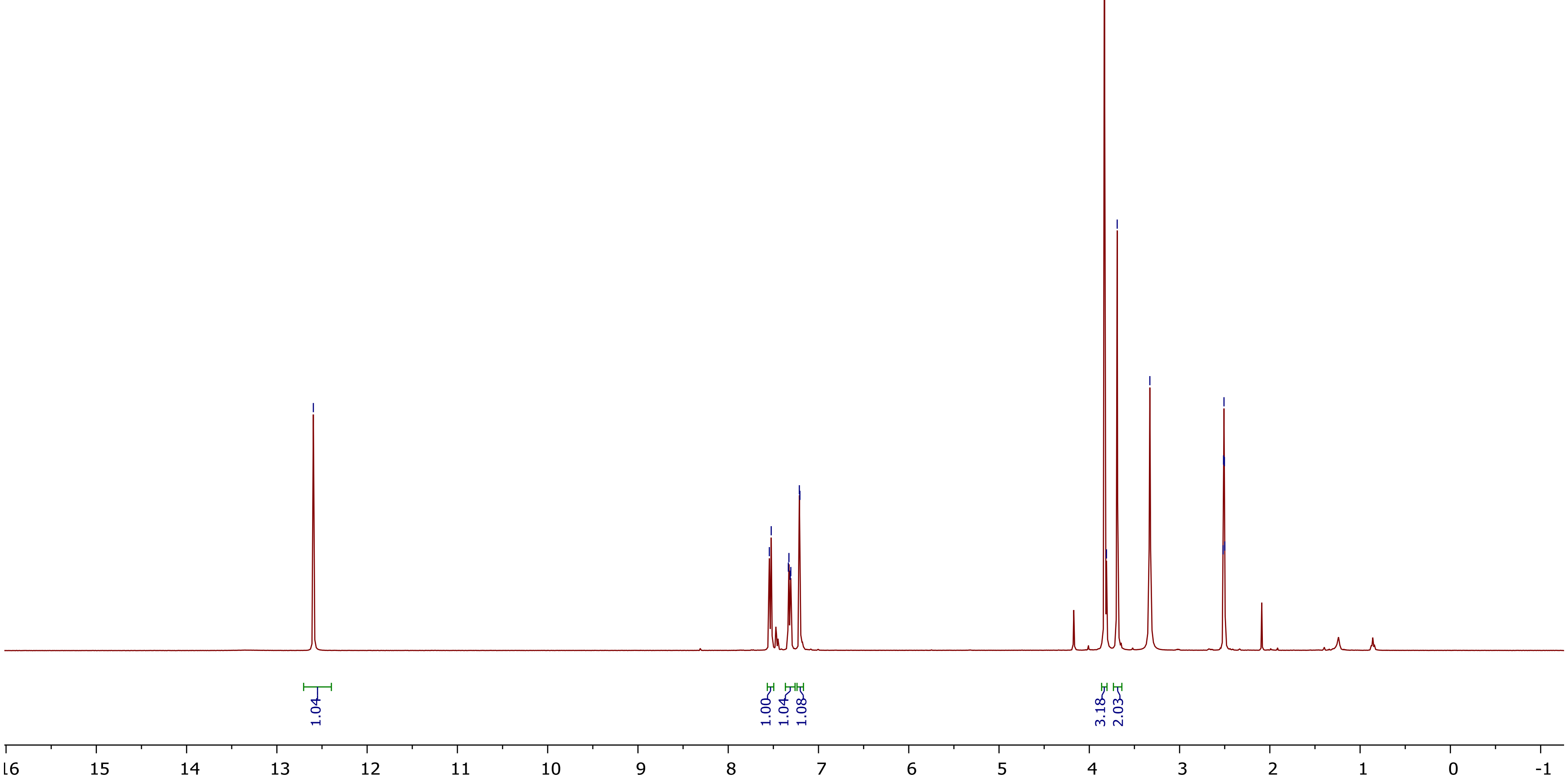

^{13}C NMR spectrum of compound 12c

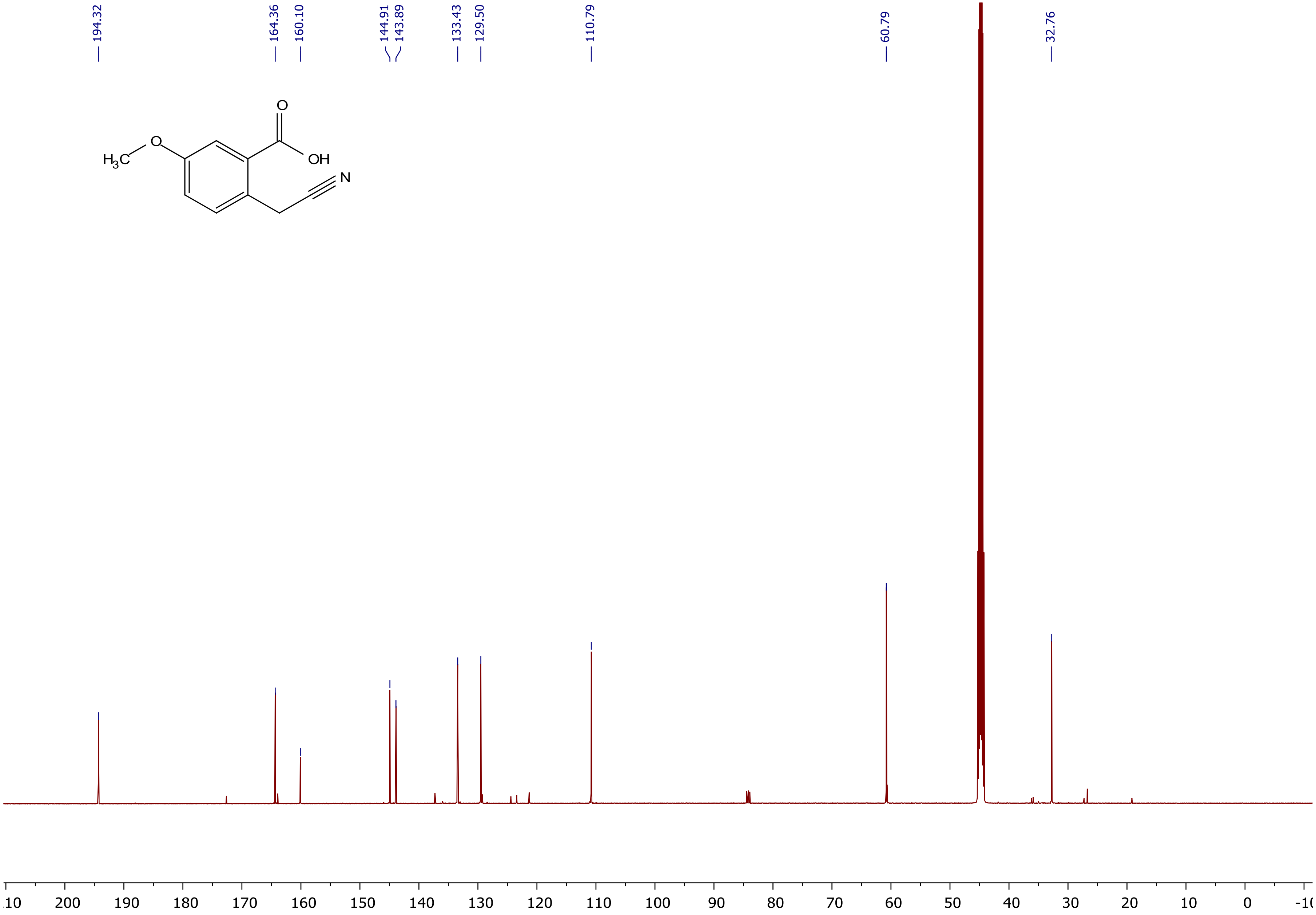

^{1}H NMR spectrum of compound 18a

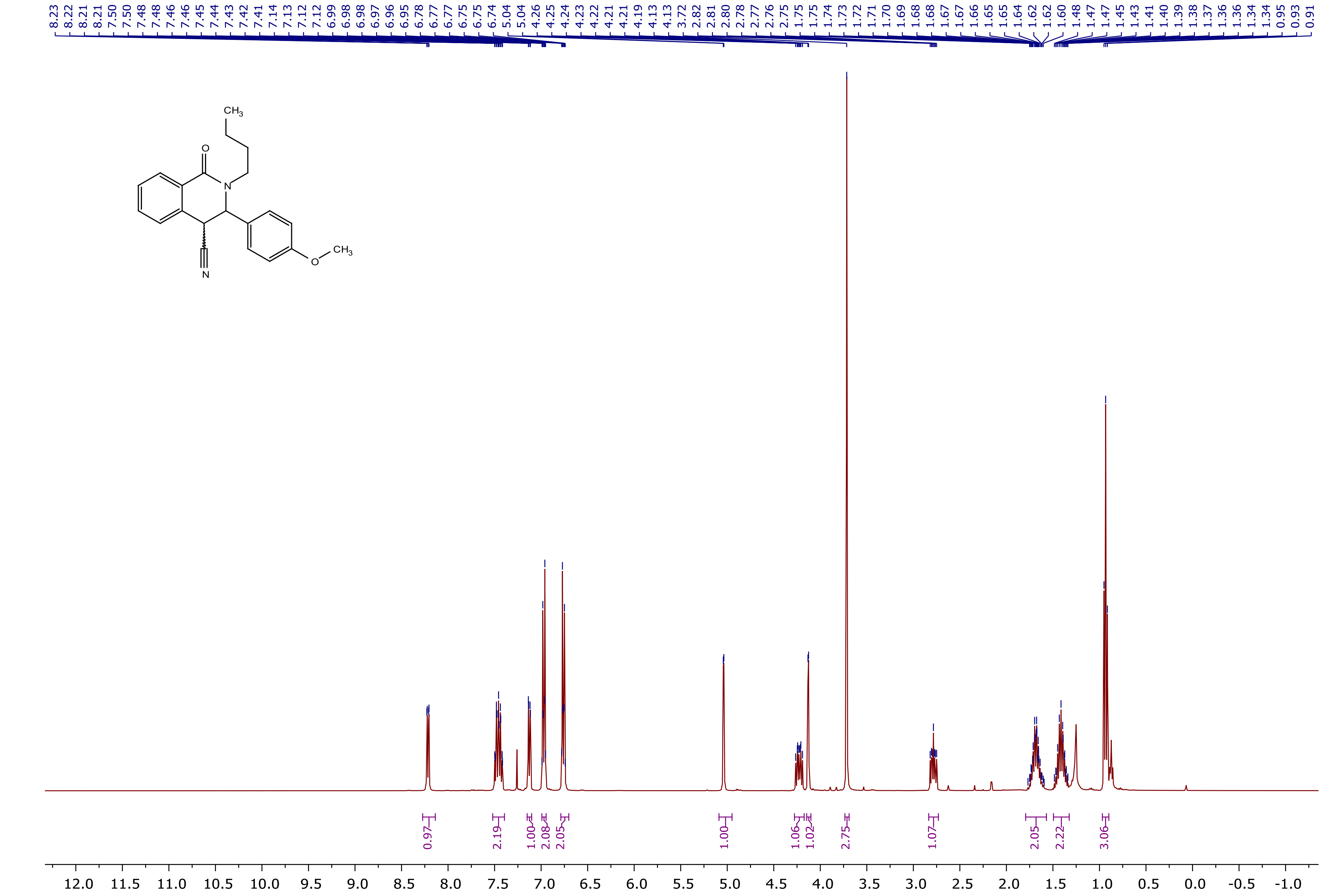

^{13}C NMR spectrum of compound 18a

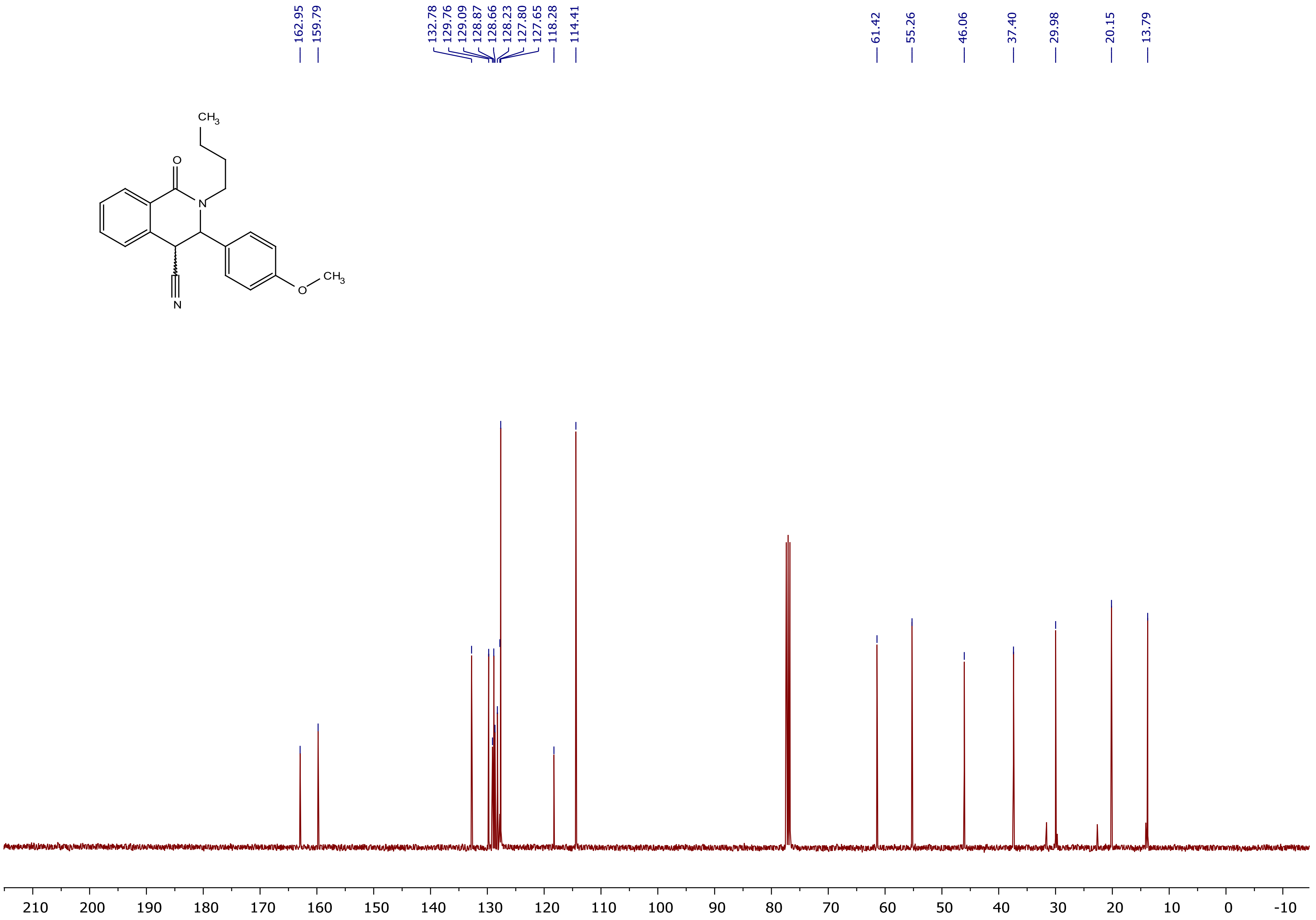

^{1}H NMR spectrum of compound 18b, trans-isomer

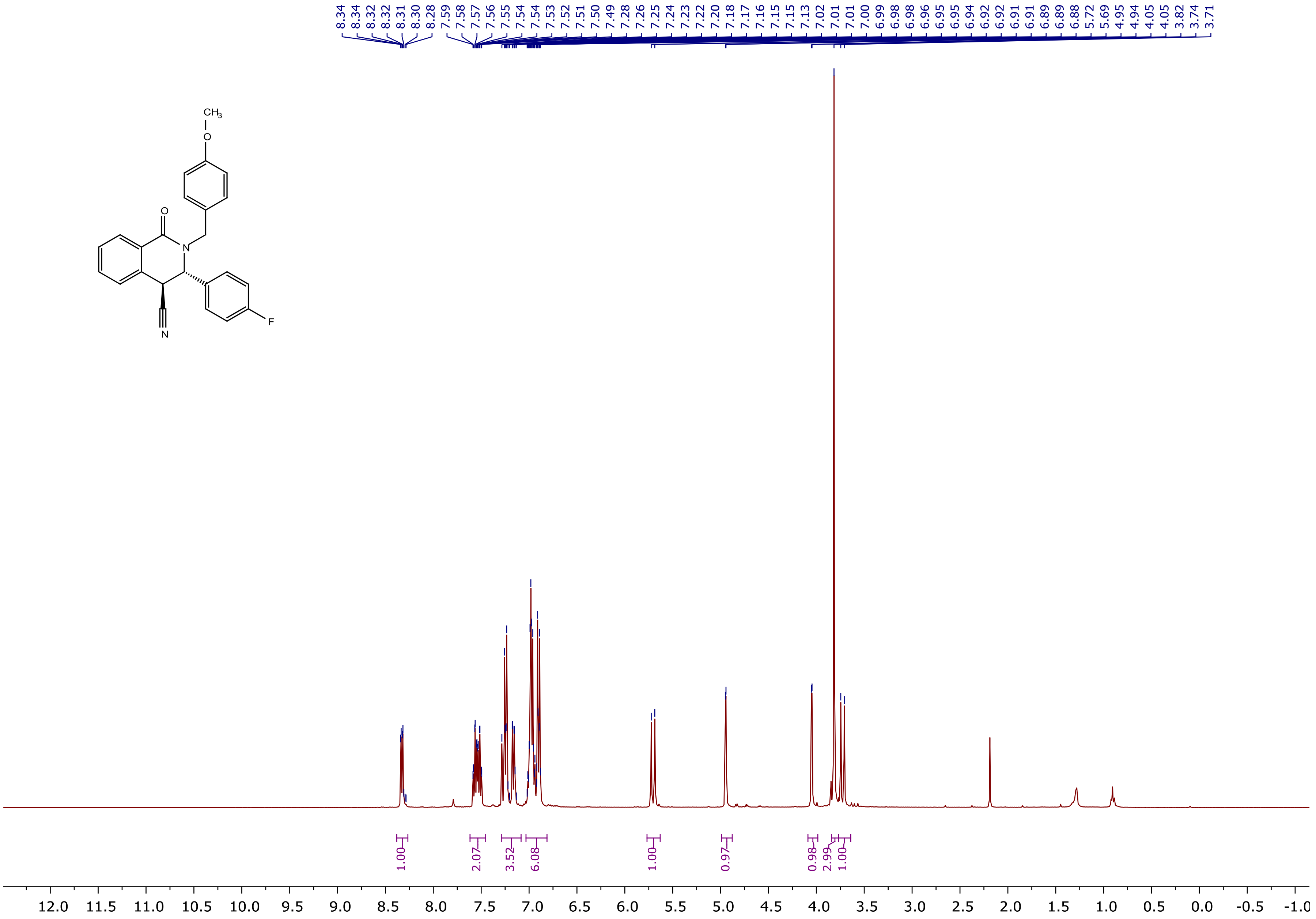

[13] C NMR spectrum of compound 18b, trans-isomer

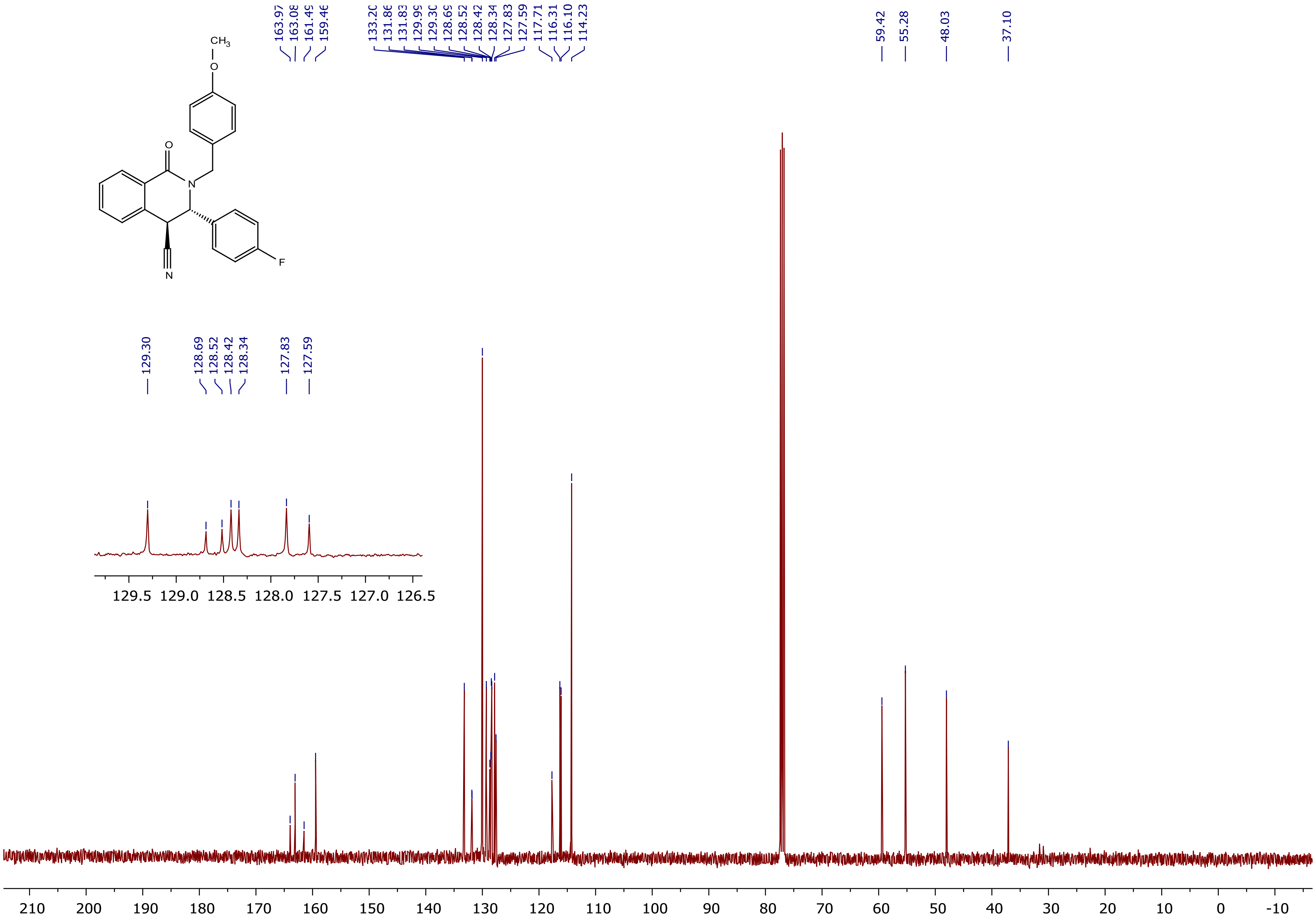

^{19}F NMR spectrum of compound 18b, trans-isomer

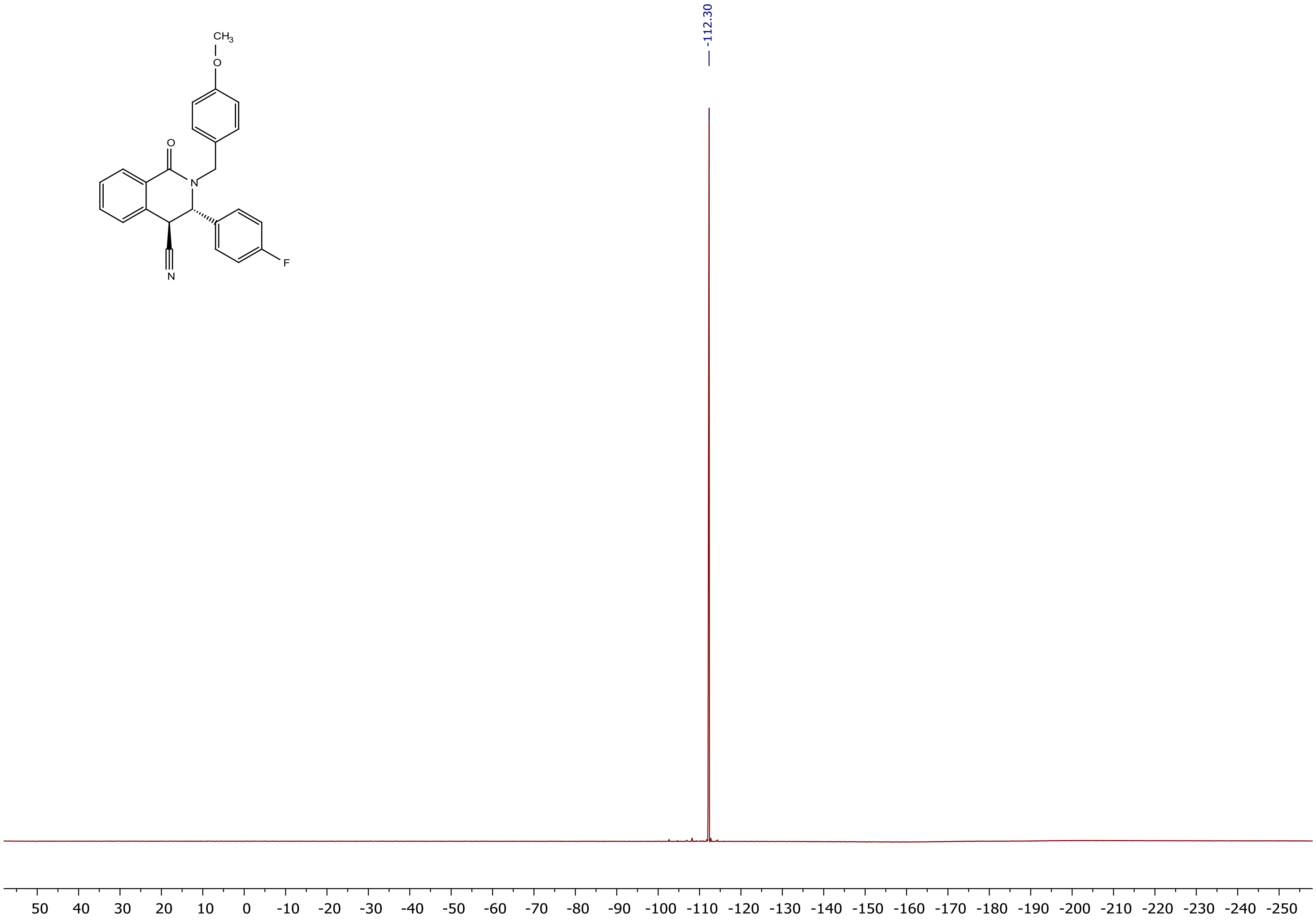

^{1}H NMR spectrum of compound compound 18b, cis-isomer

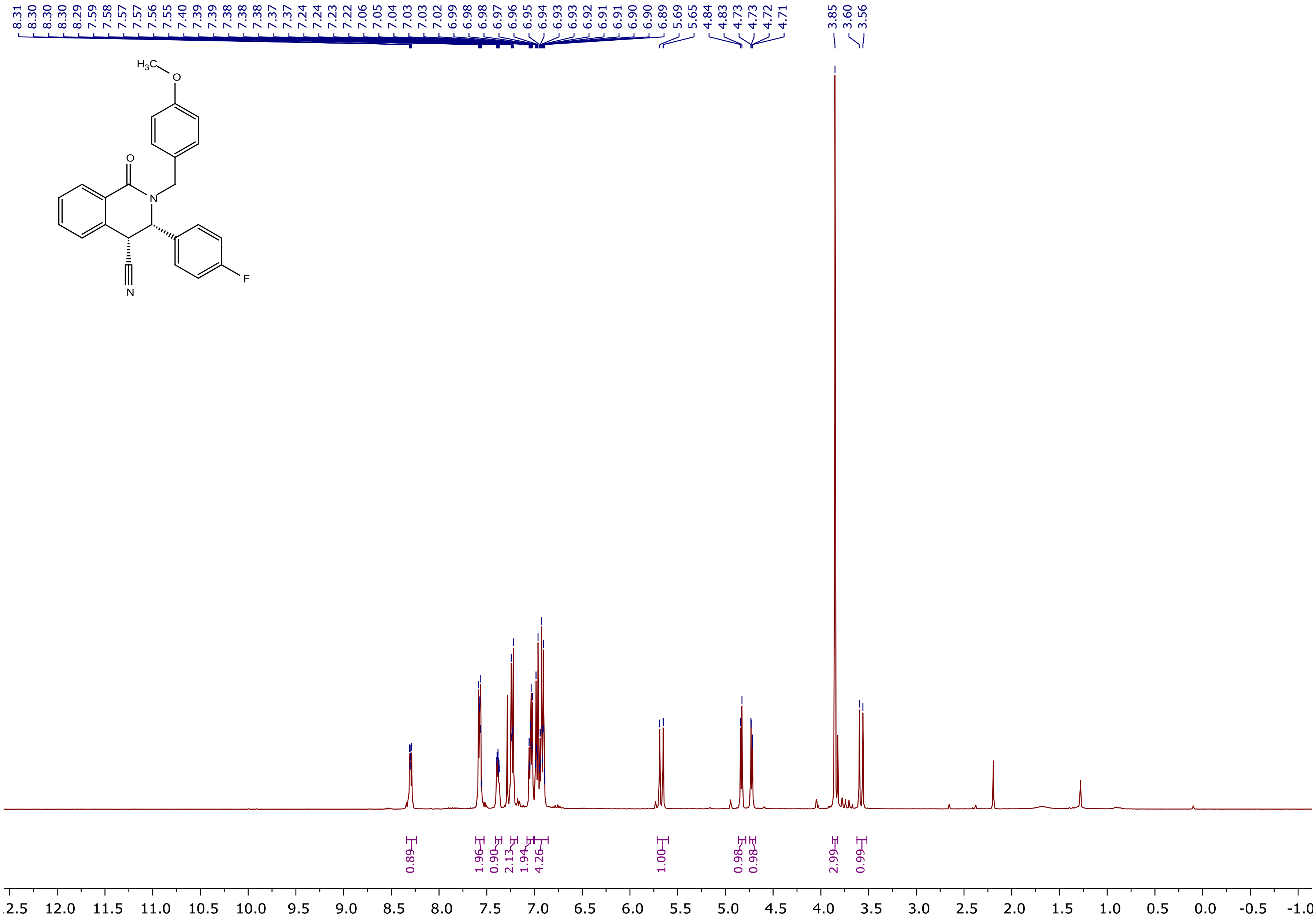

^{13}C NMR spectrum of compound 18b, cis-isomer

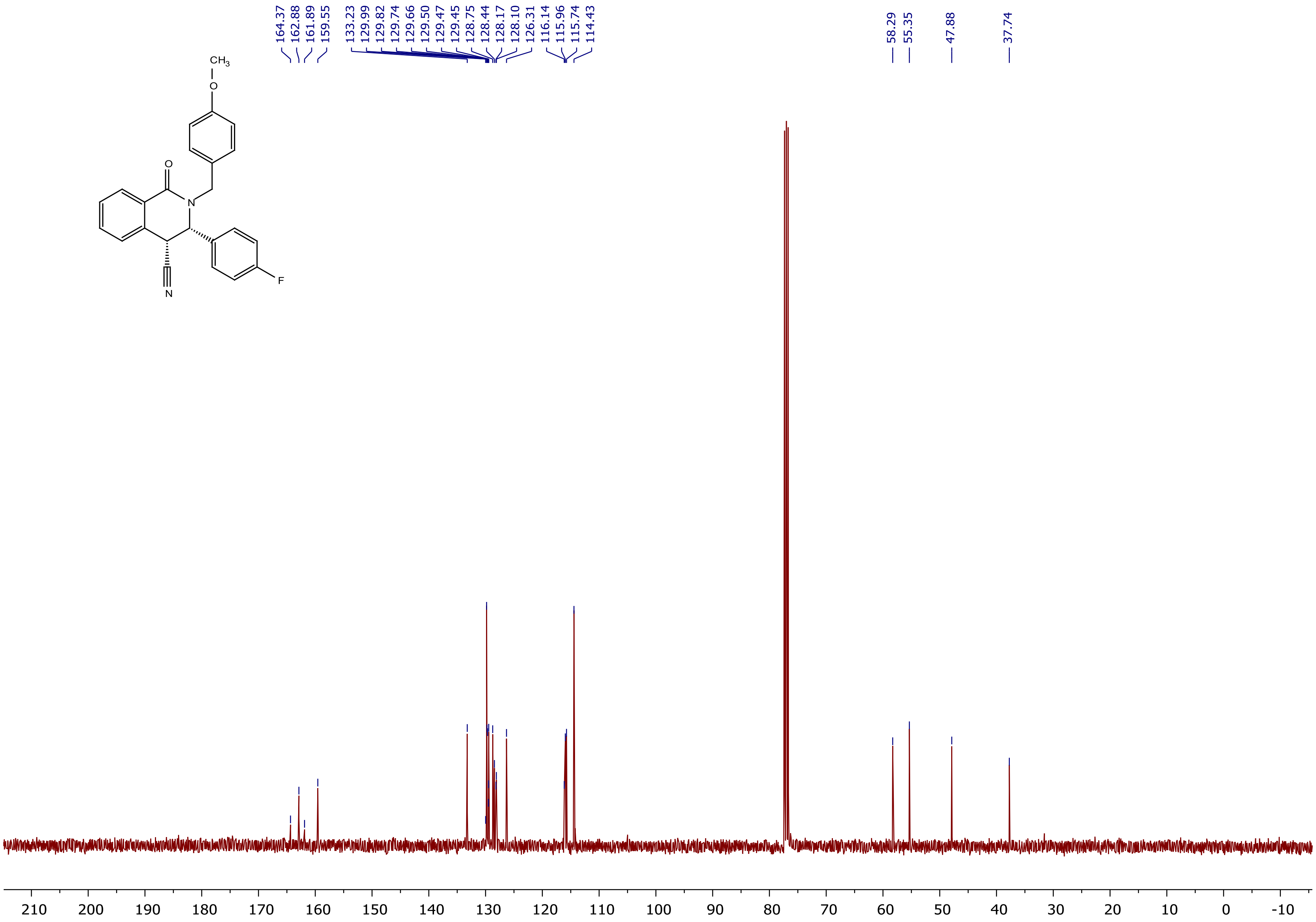

^{19}F NMR spectrum of compound 18b, cis-isomer

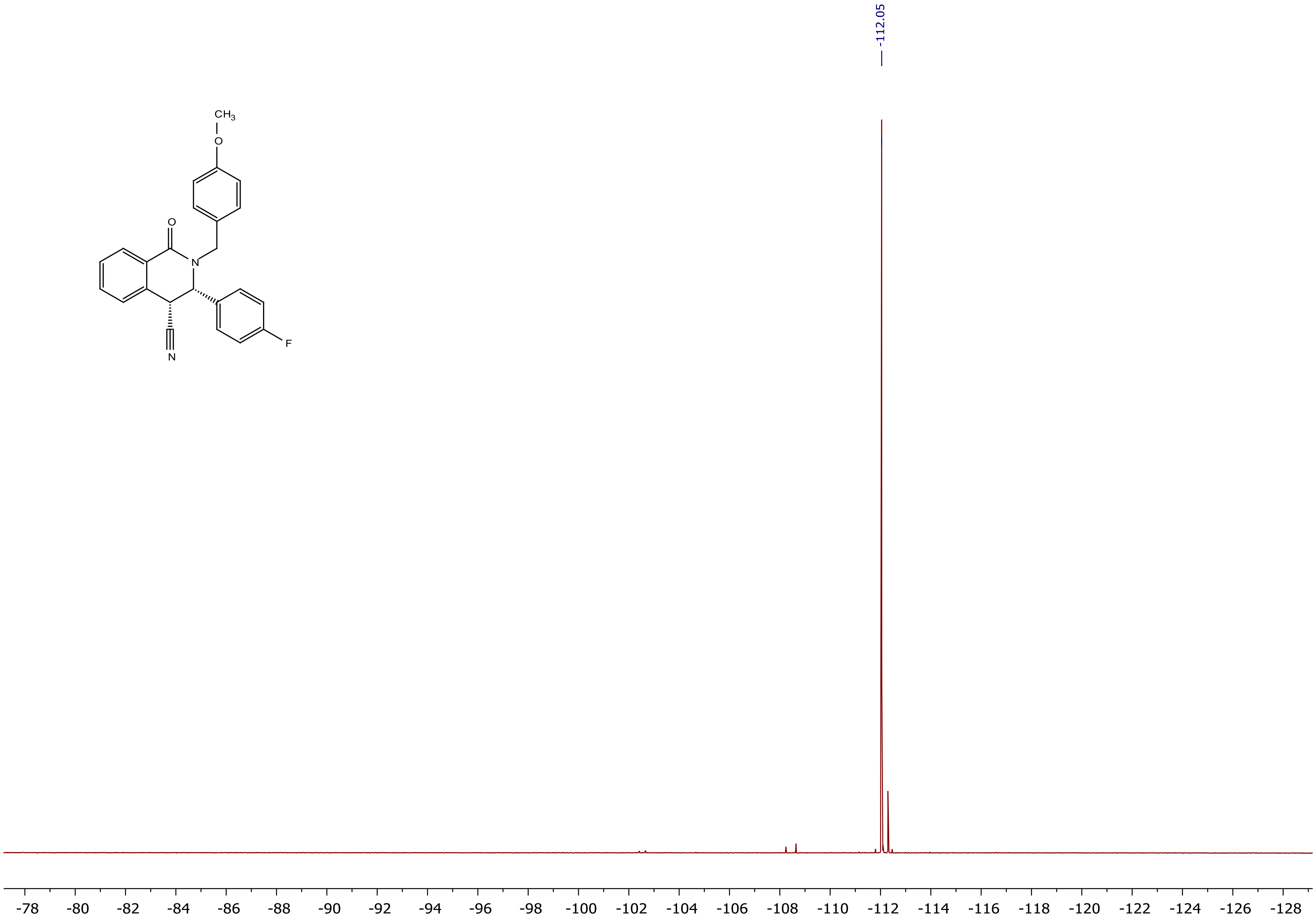

^{1}H NMR spectrum of compound 18c

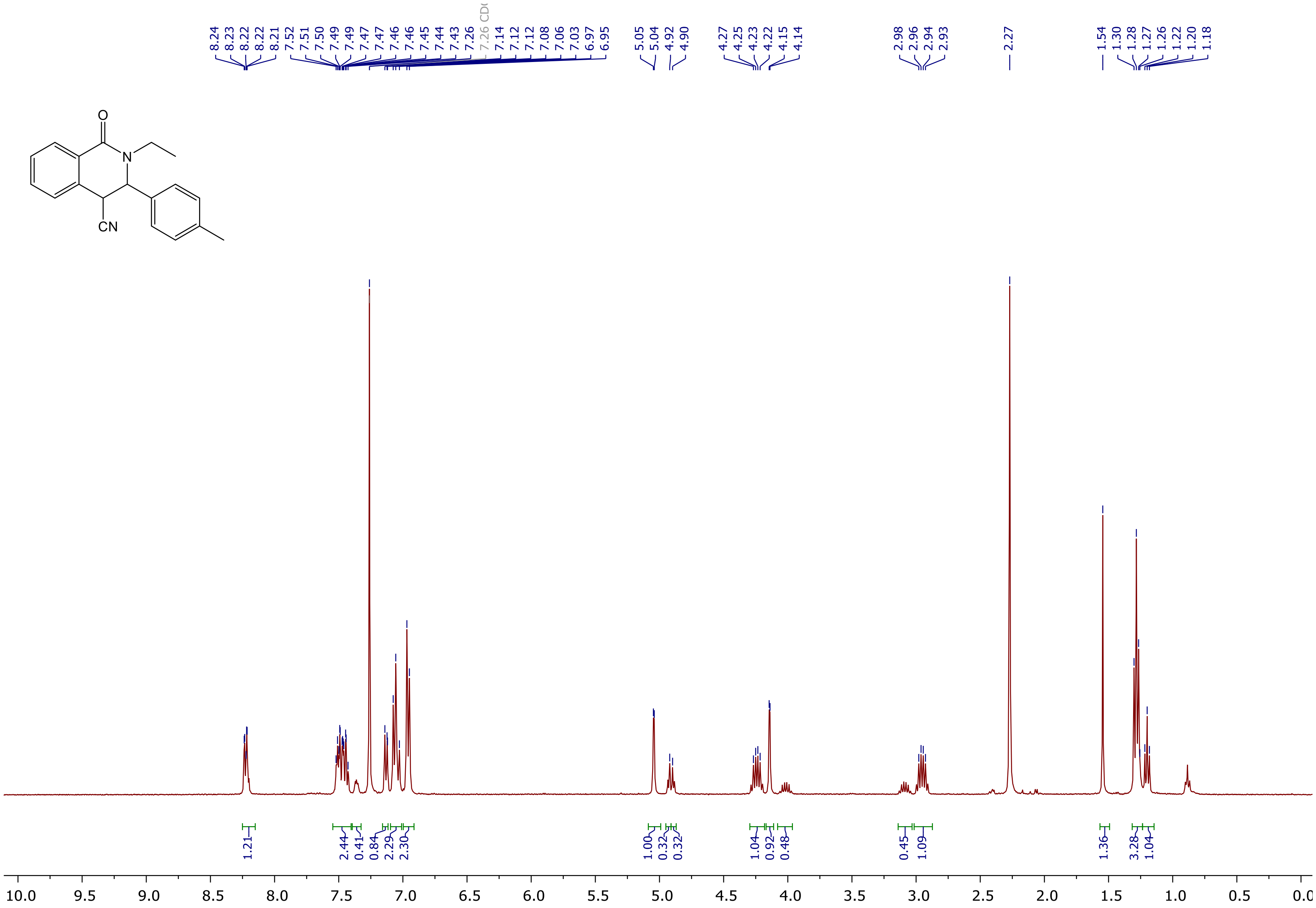

[1]13C NMR spectrum of compound 18c

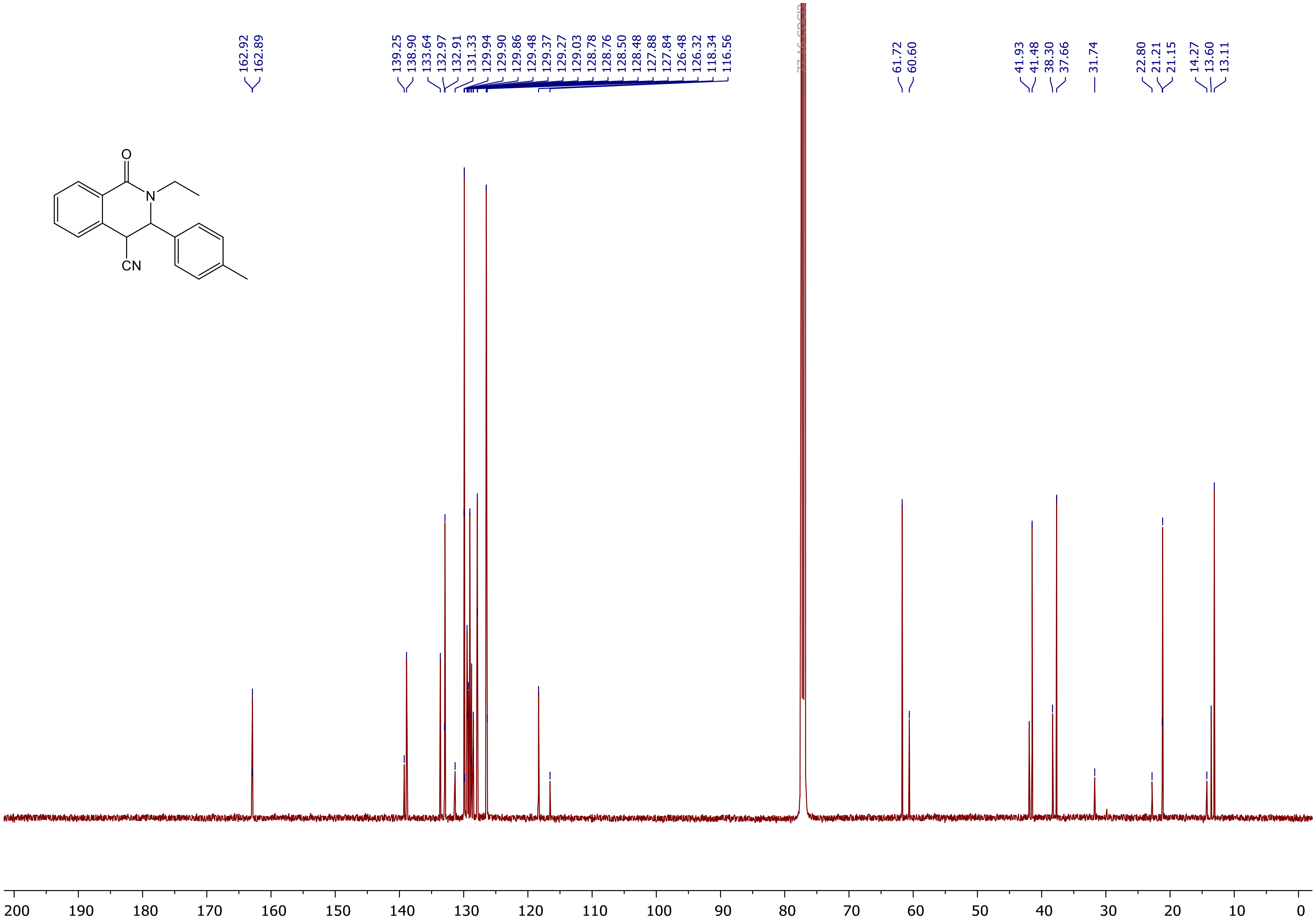

^{1}H NMR spectrum of compound 18d

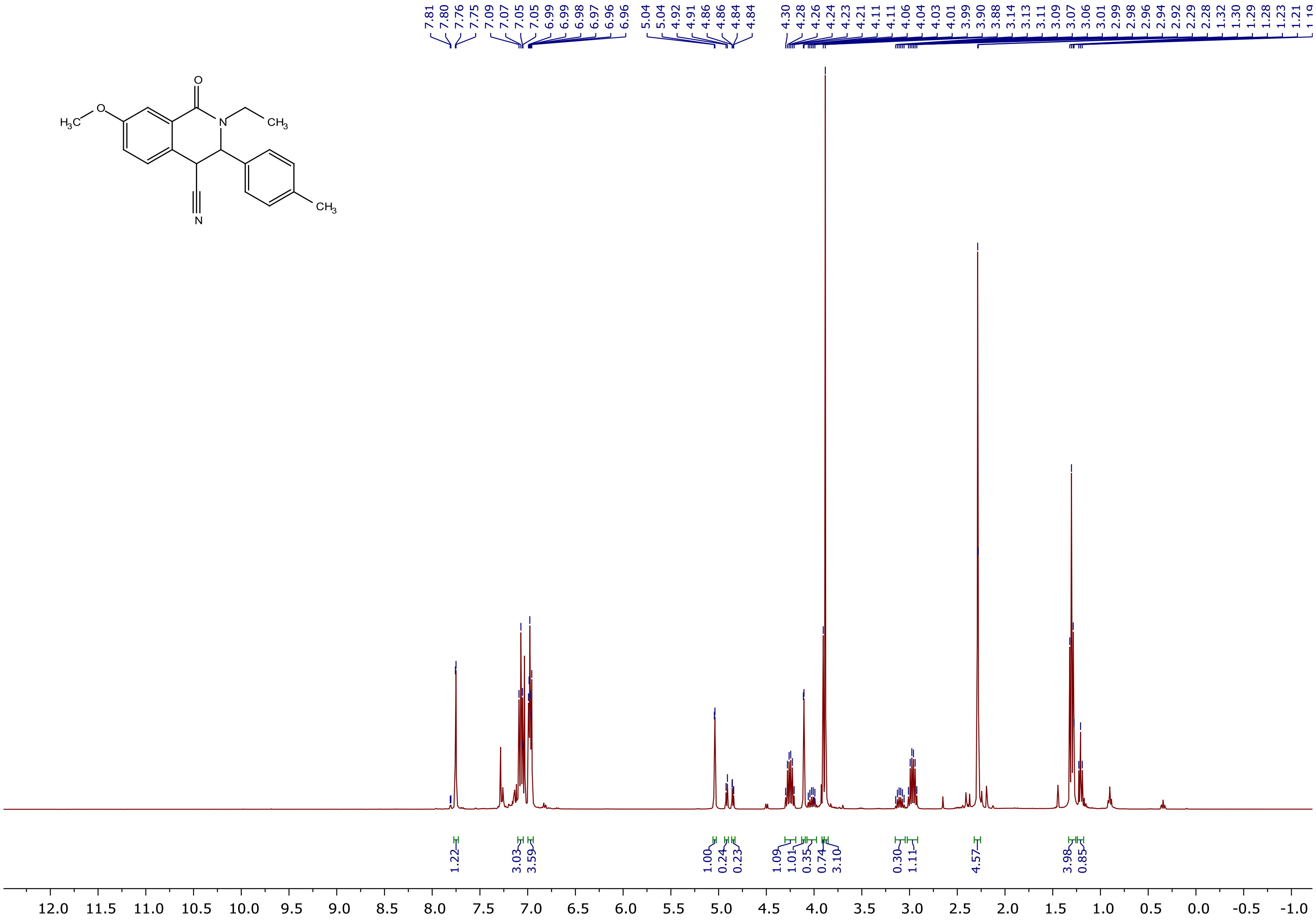

^{13}C NMR spectrum of compound 18d

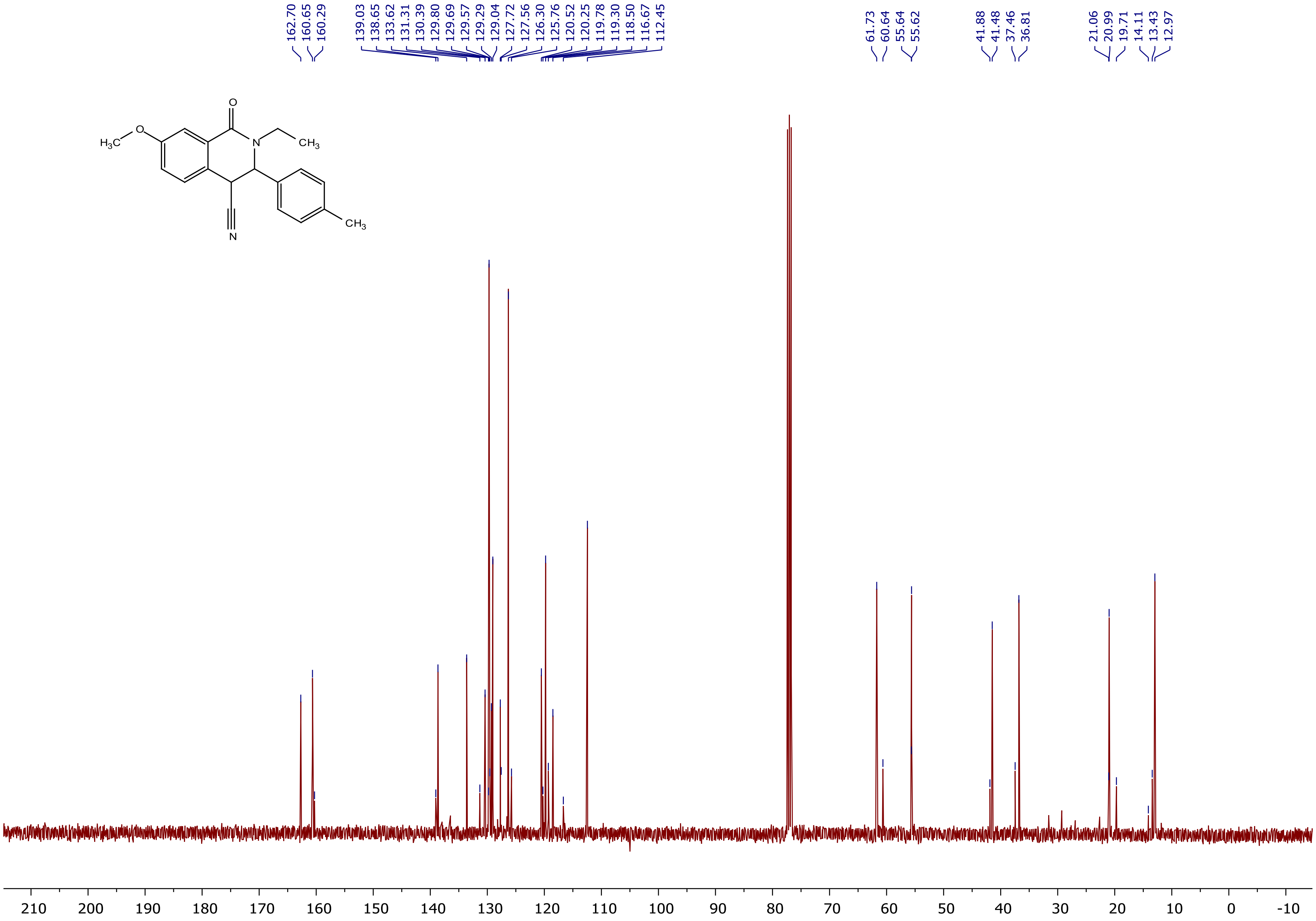

^{1}H NMR spectrum of compound 18e

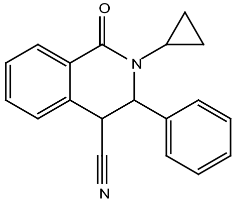

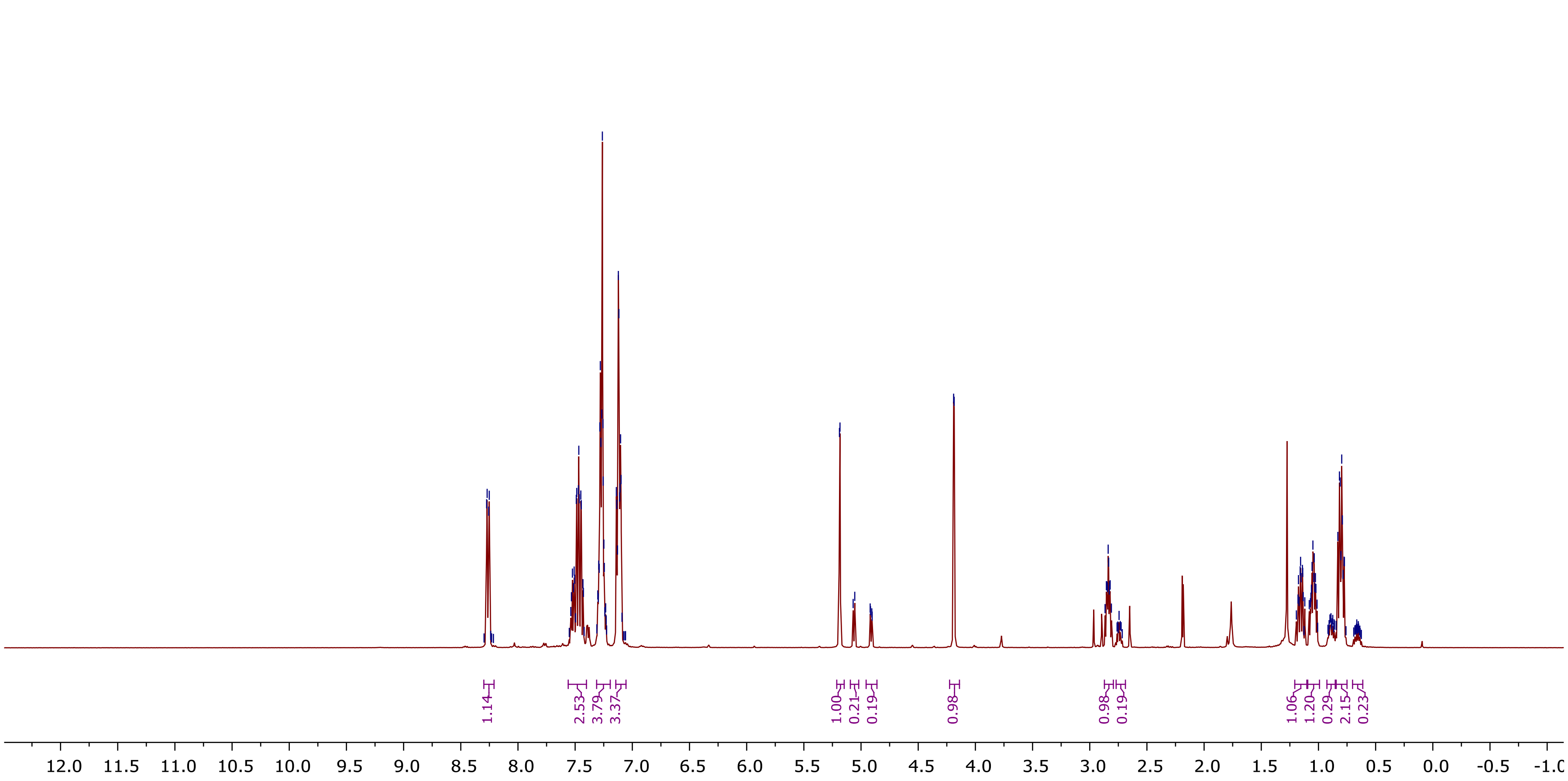

^{13}C NMR spectrum of compound 18e

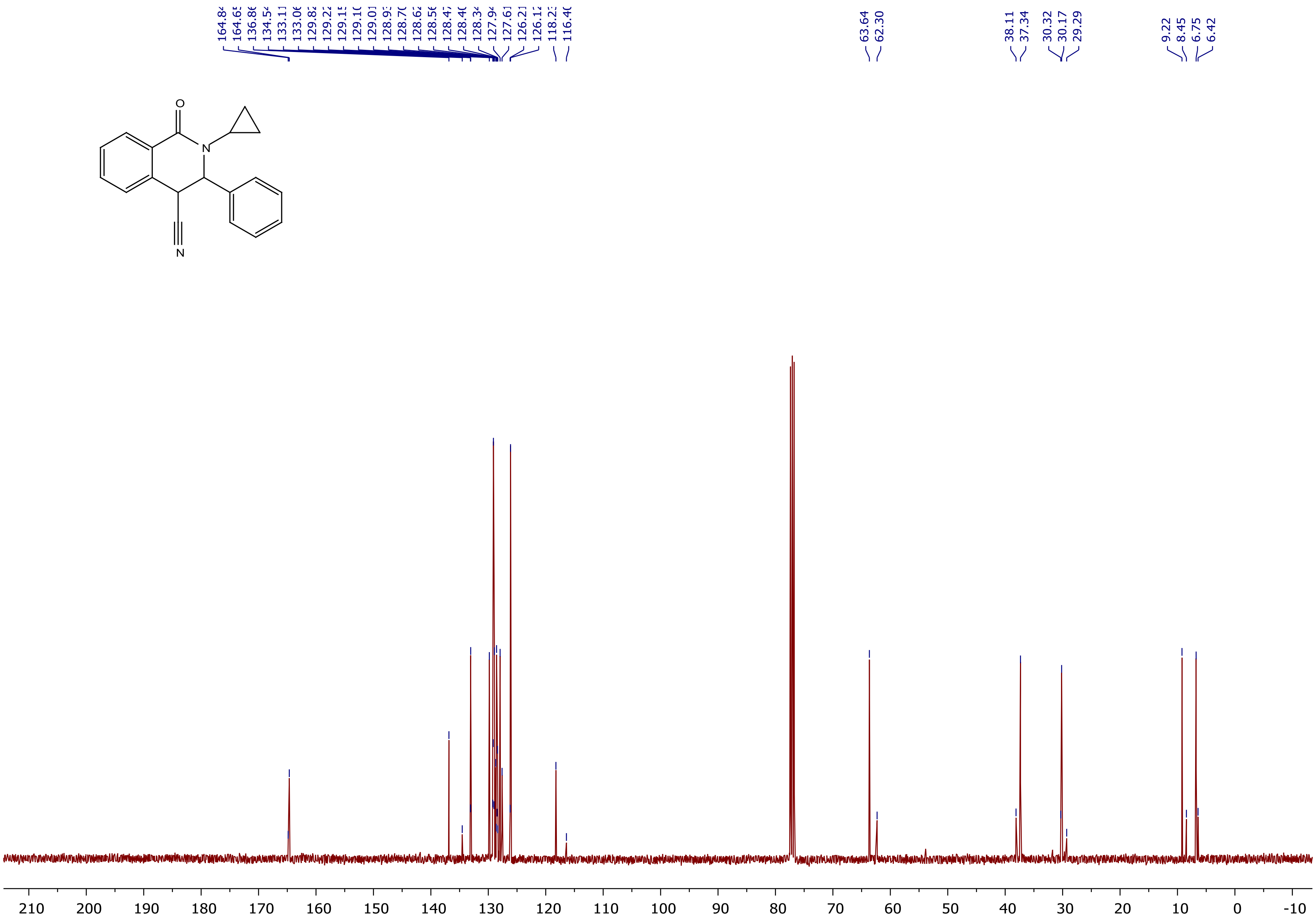

^{1}H NMR spectrum of compound 18f

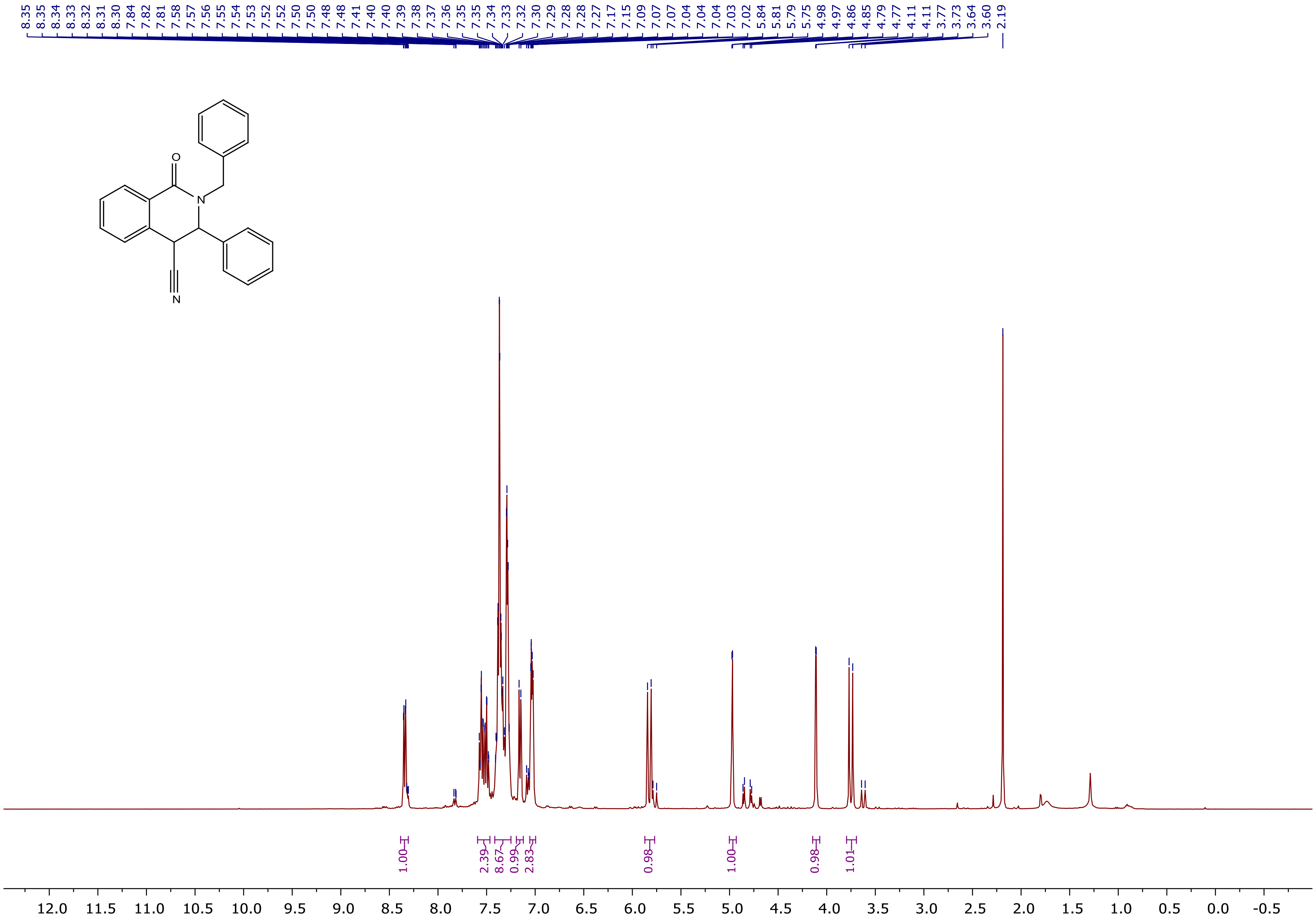

^{13}C NMR spectrum of compound 18f

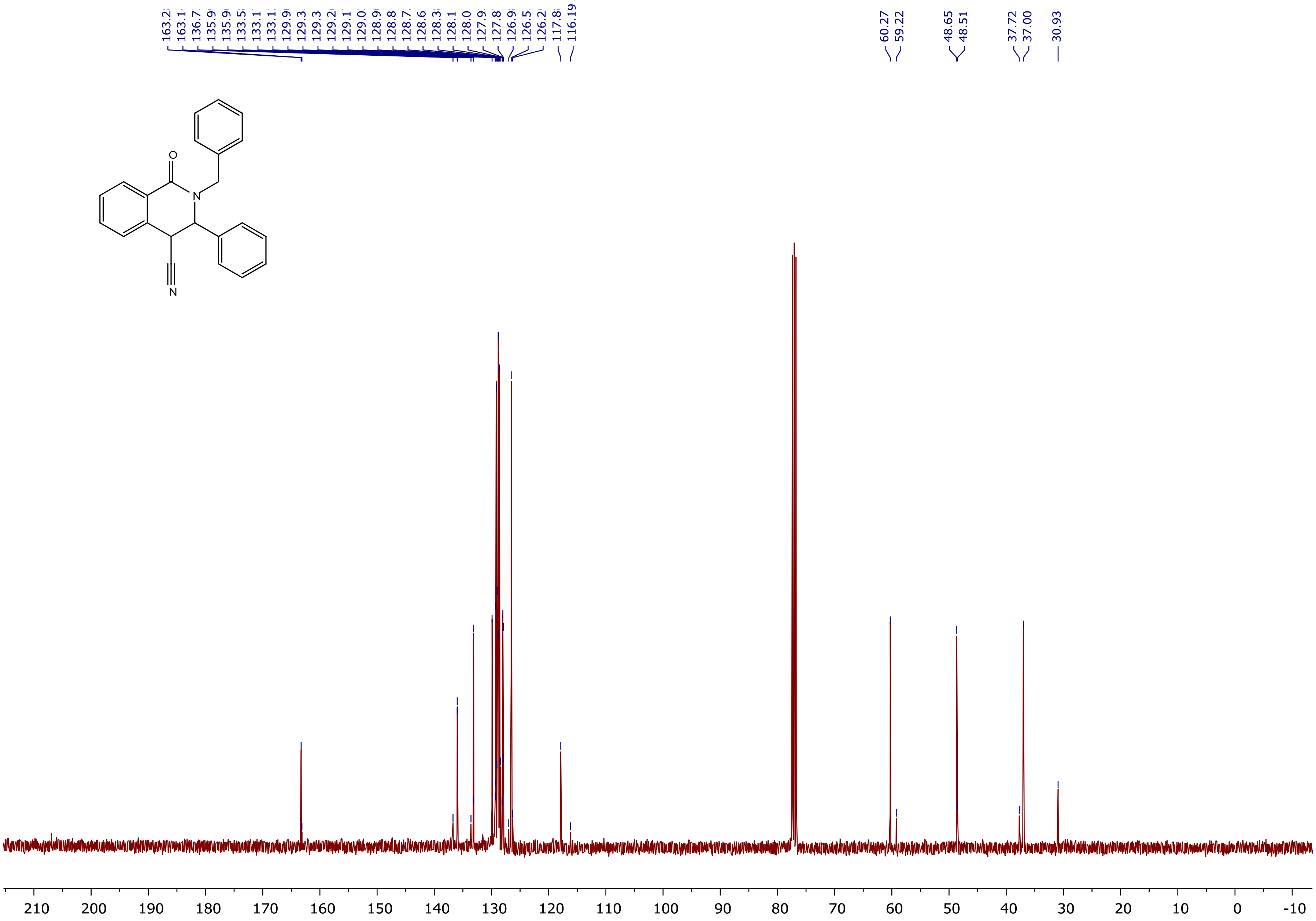

^{1}H NMR spectrum of compound 18g

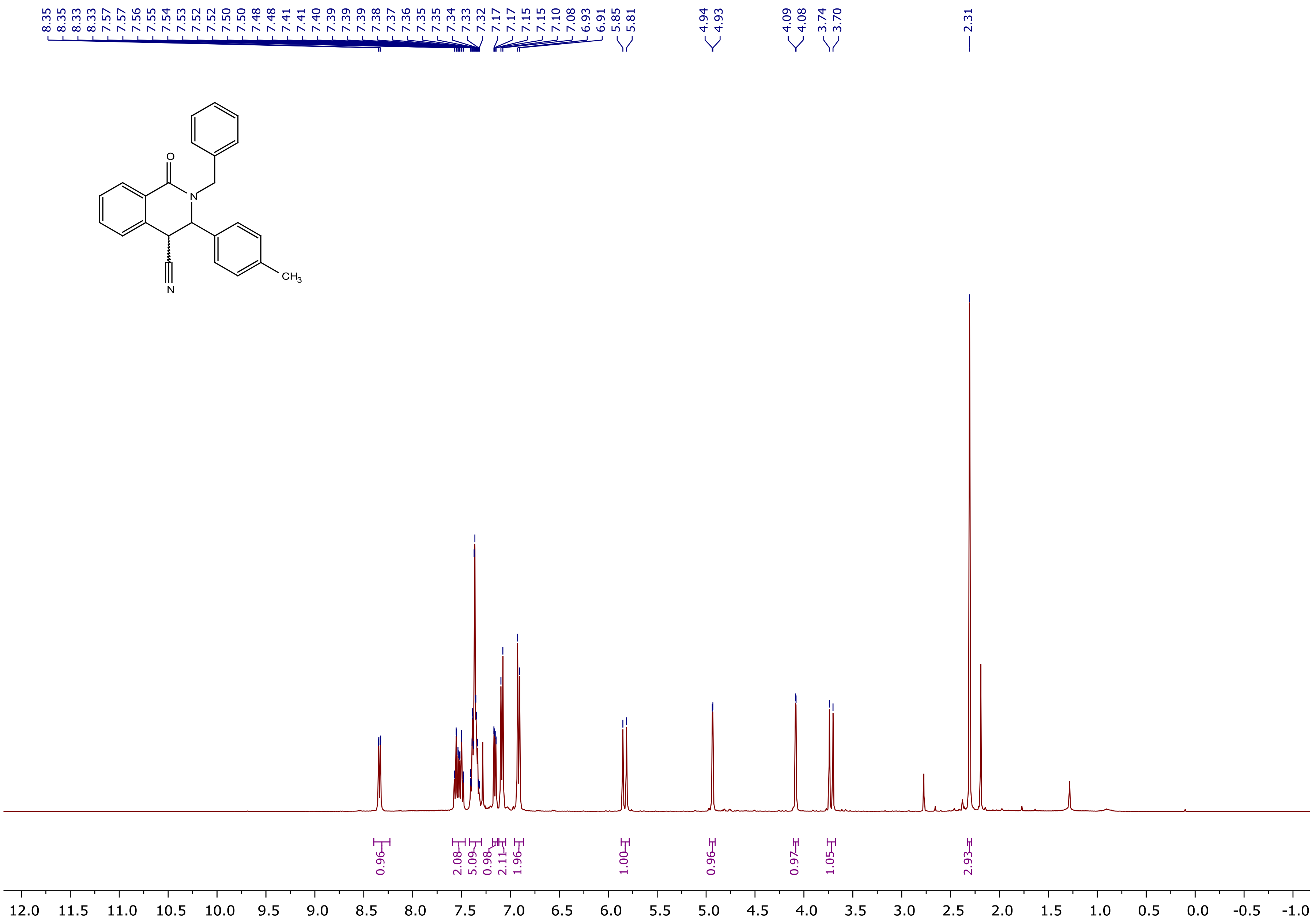

^{13}C NMR spectrum of compound 18g

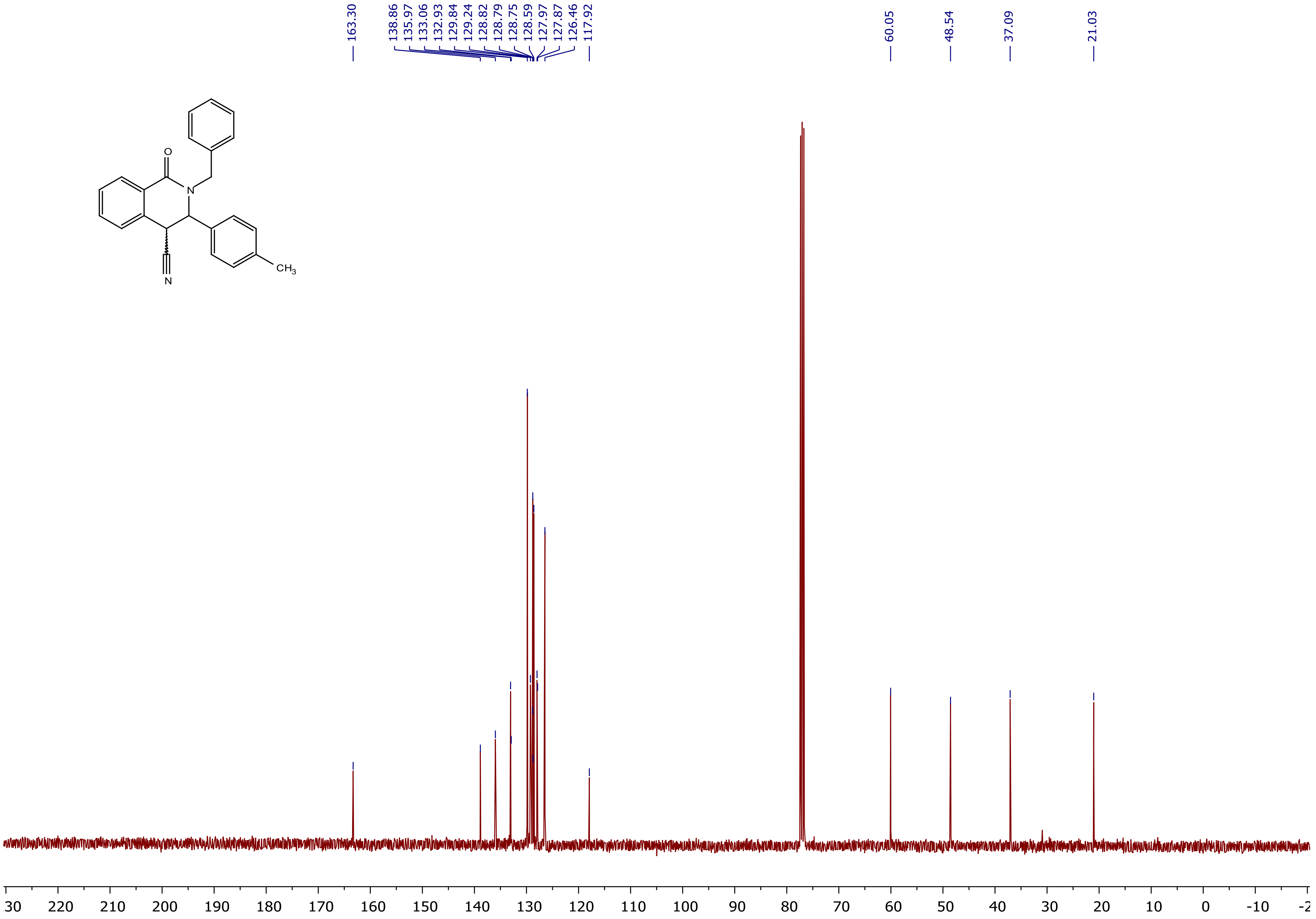

^{1}H NMR spectrum of compound 18h

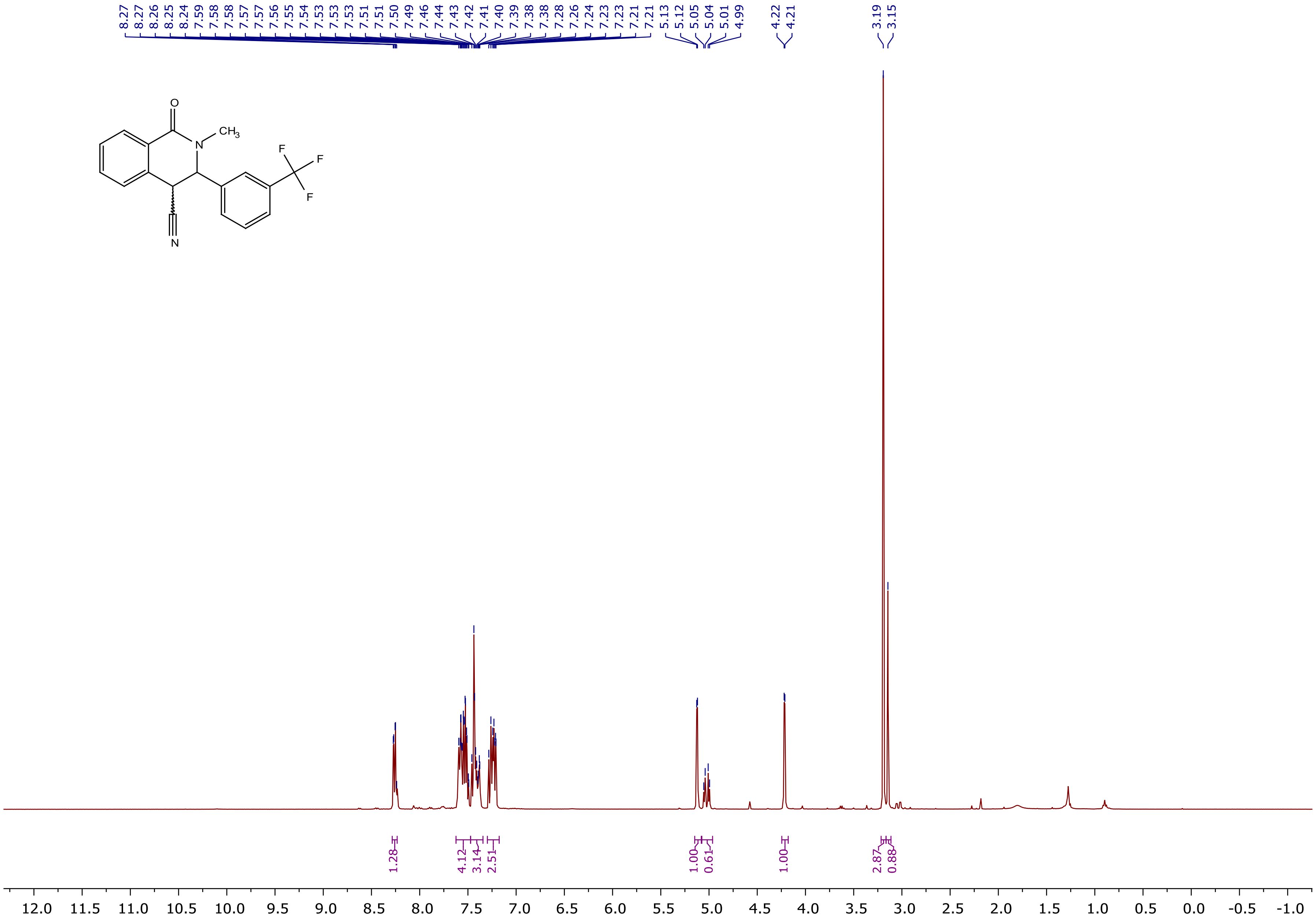

^{13}C NMR spectrum of compound 18h

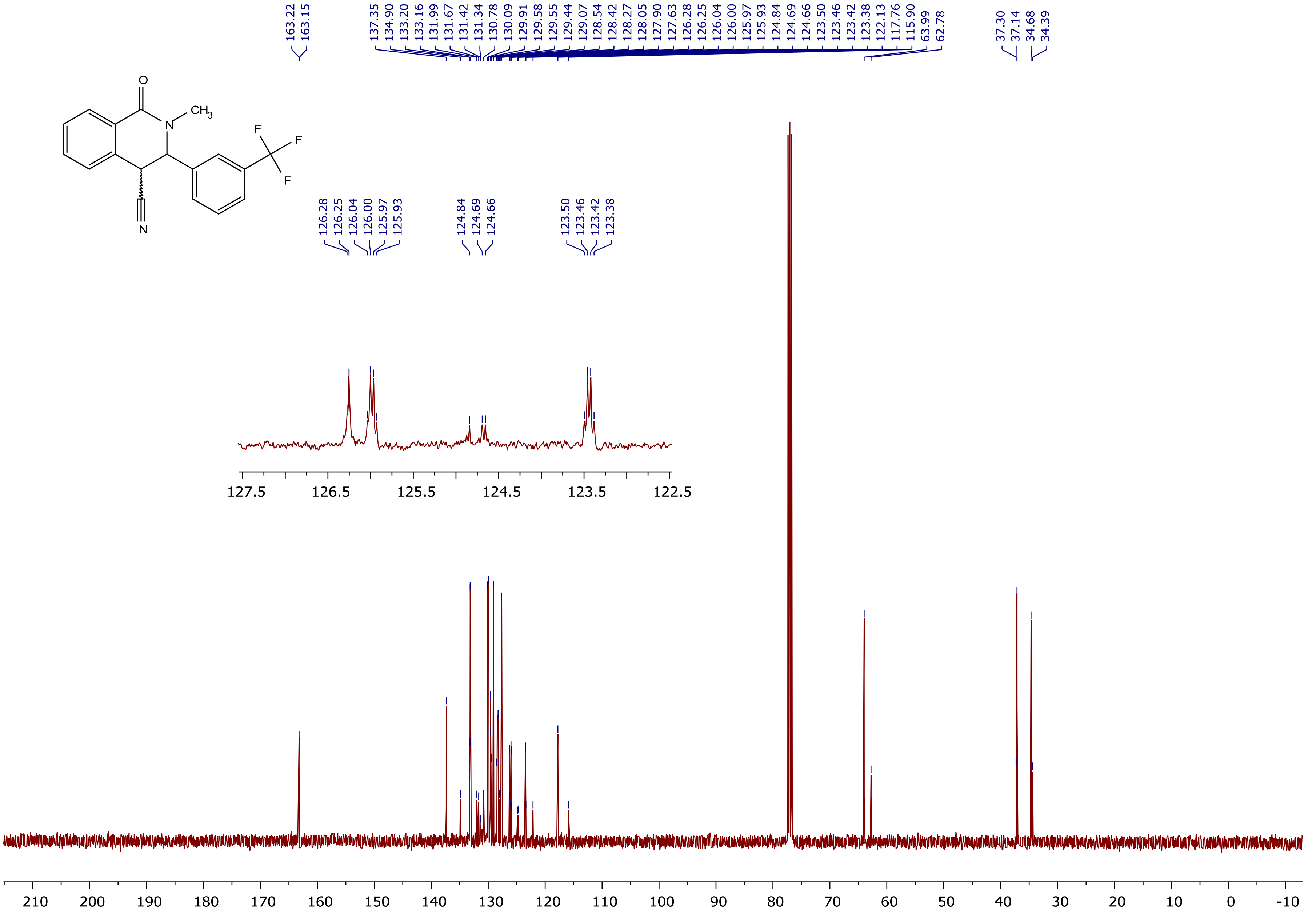

^{19}F NMR spectrum of compound 18h

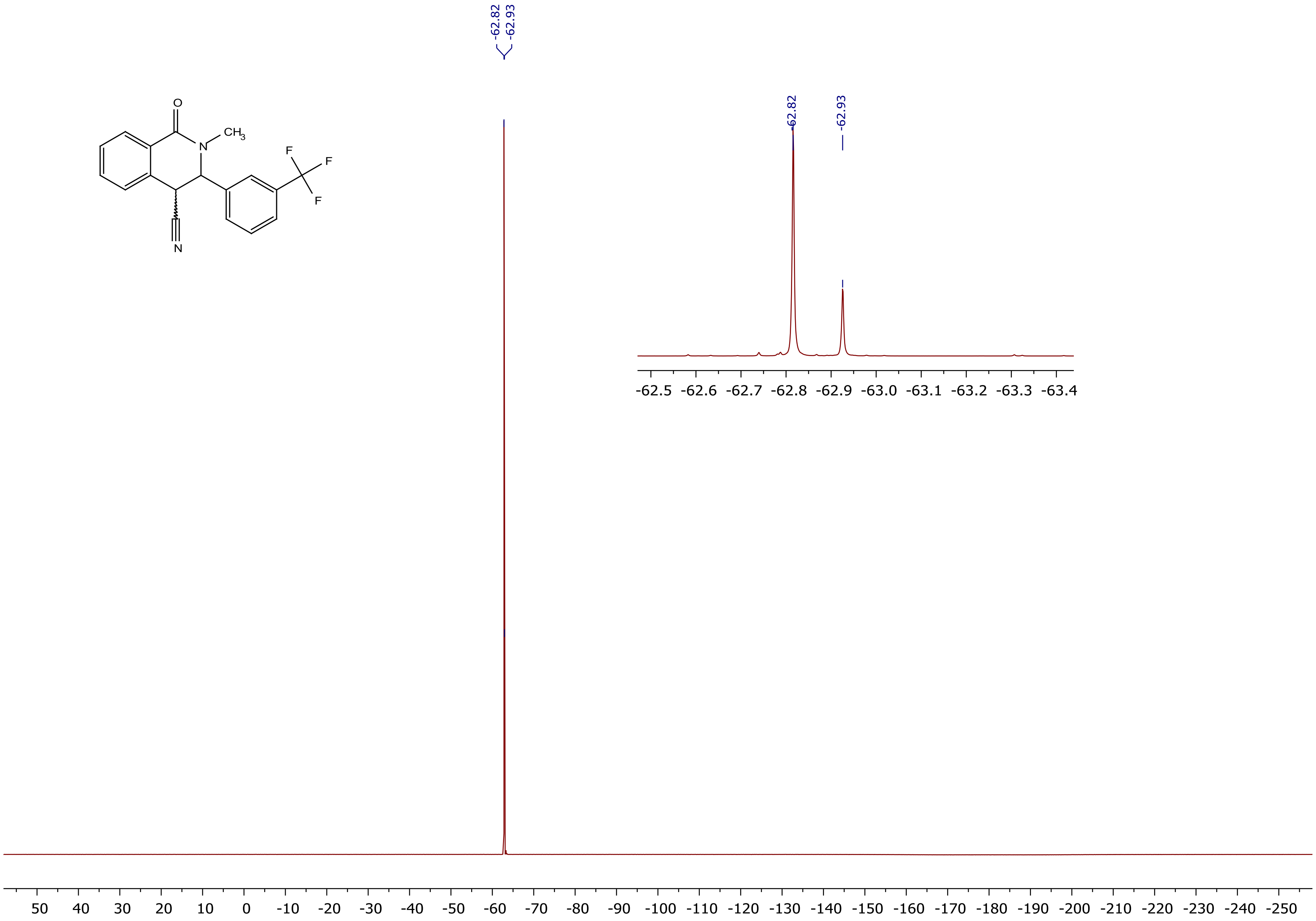

^{1}H NMR spectrum of compound 18i

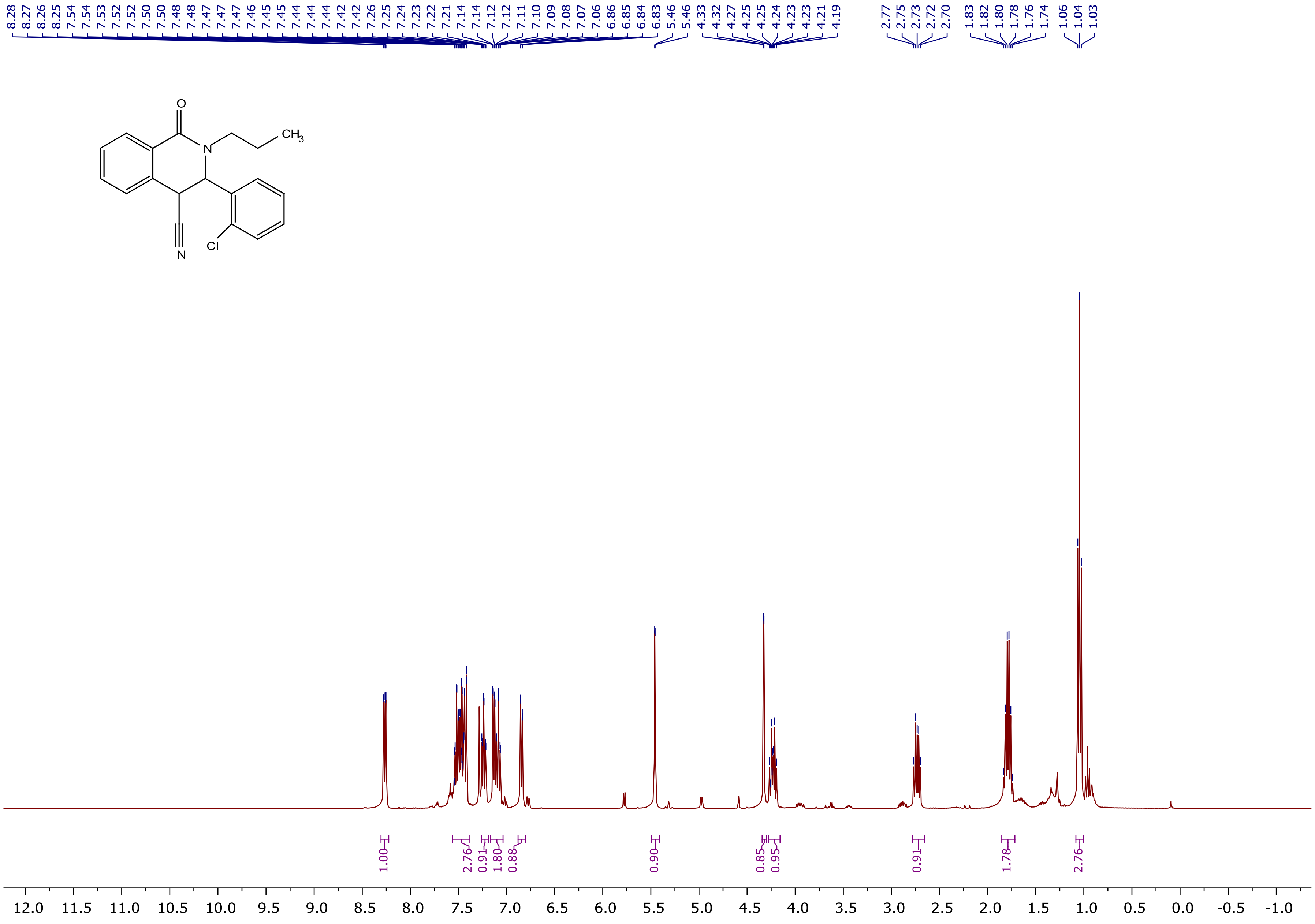

^{13}C NMR spectrum of compound 18i

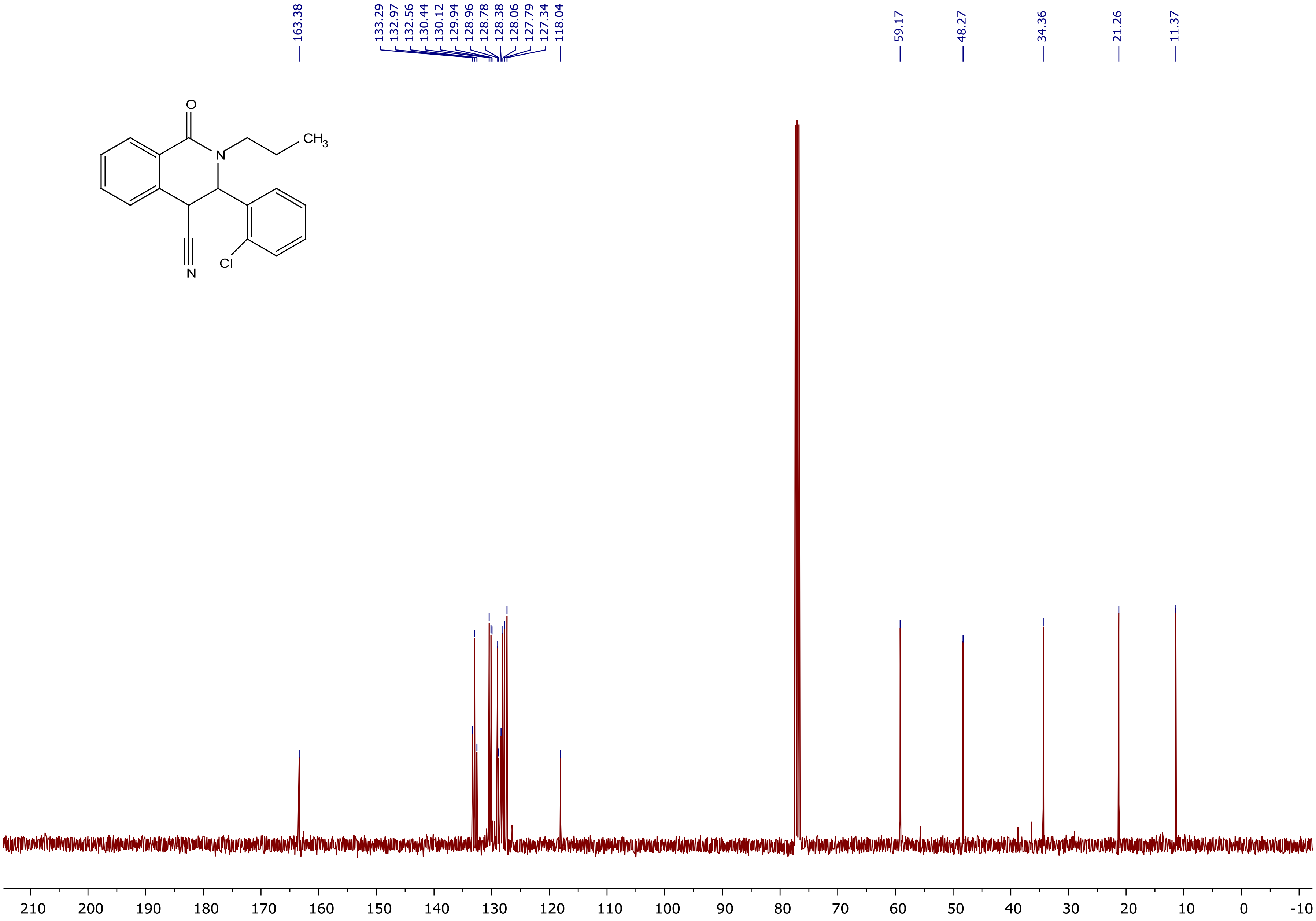

^{1}H NMR spectrum of compound 18j

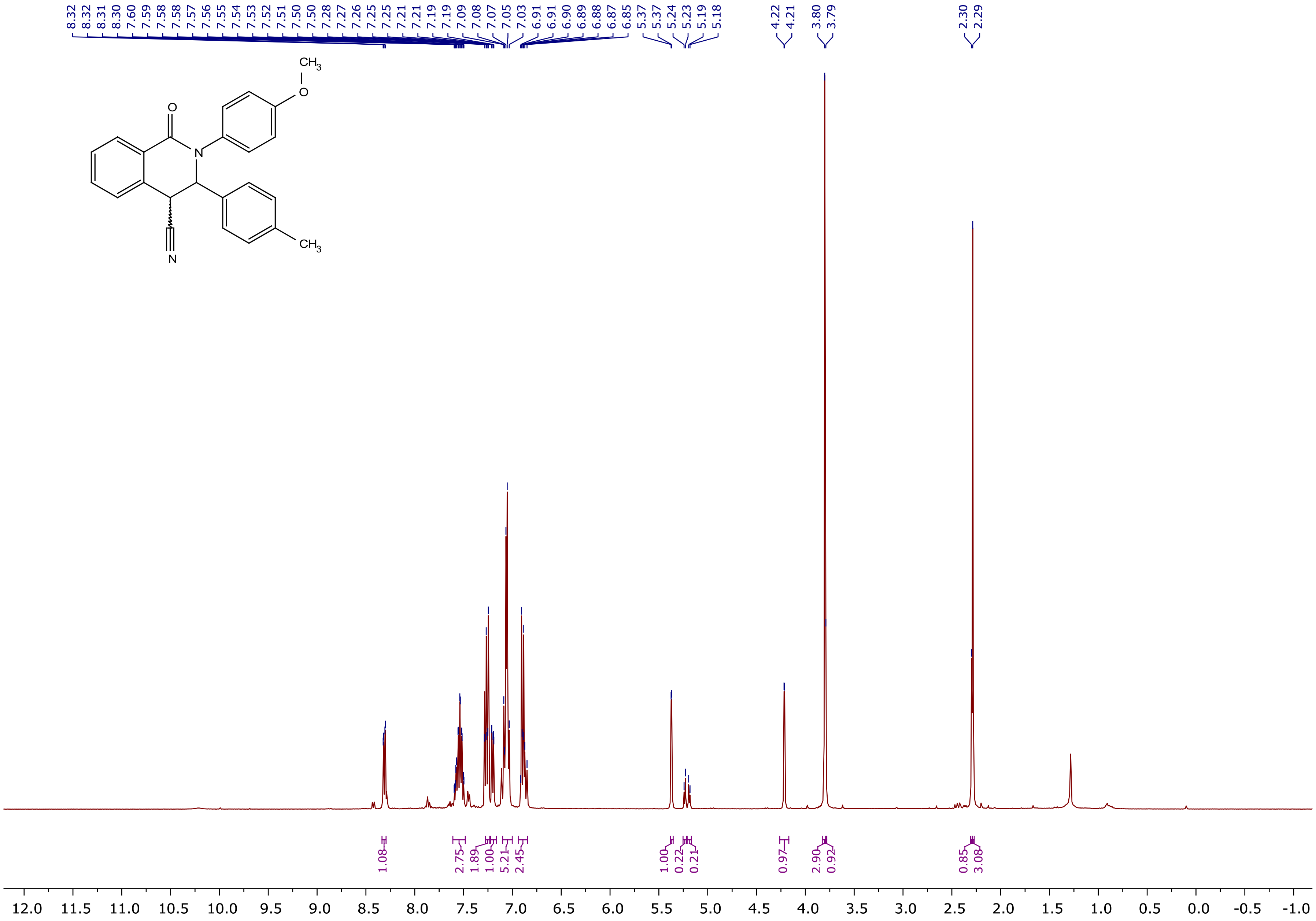

^{13}C NMR spectrum of compound 18j

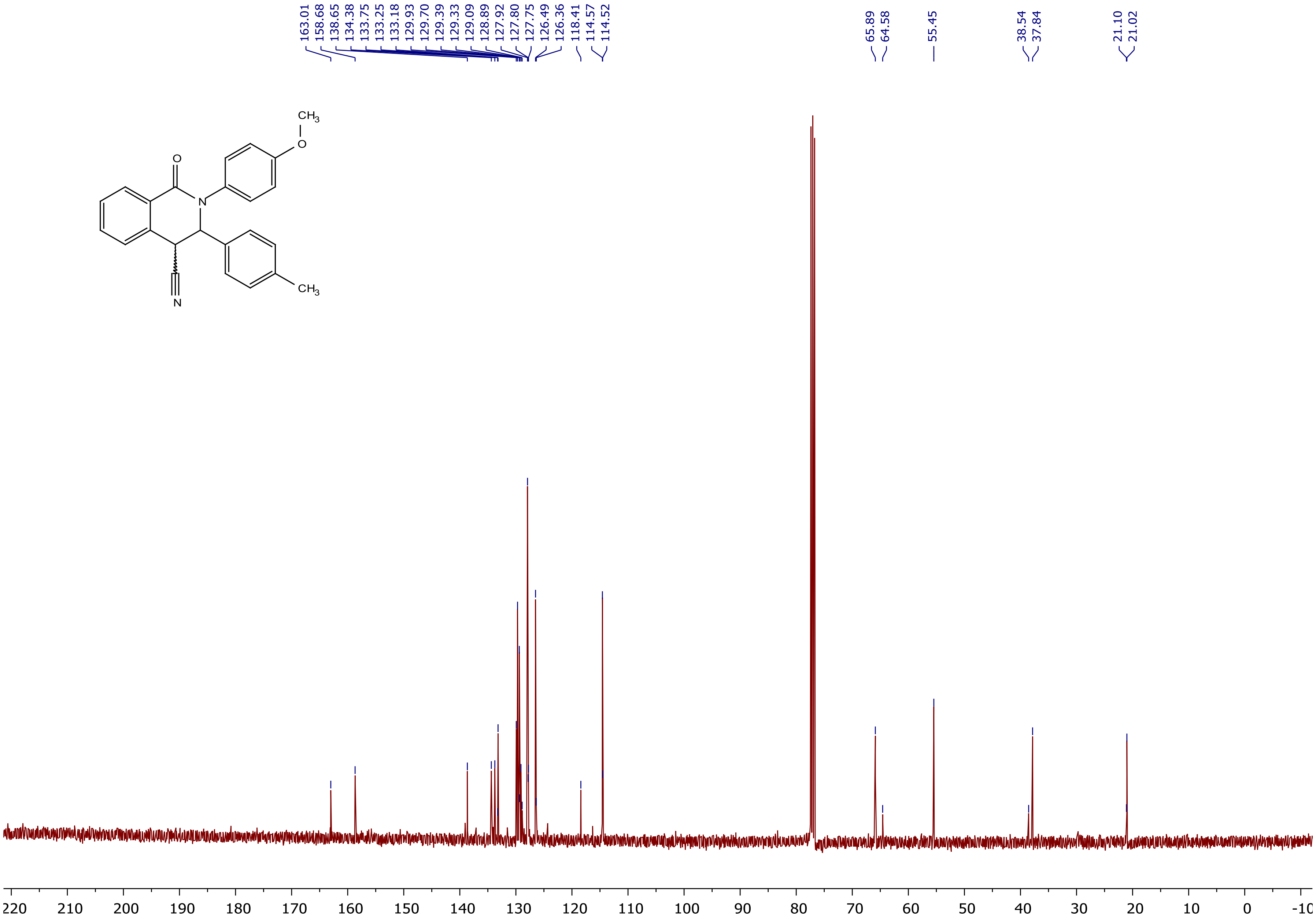

^{1}H NMR spectrum of compound 18k

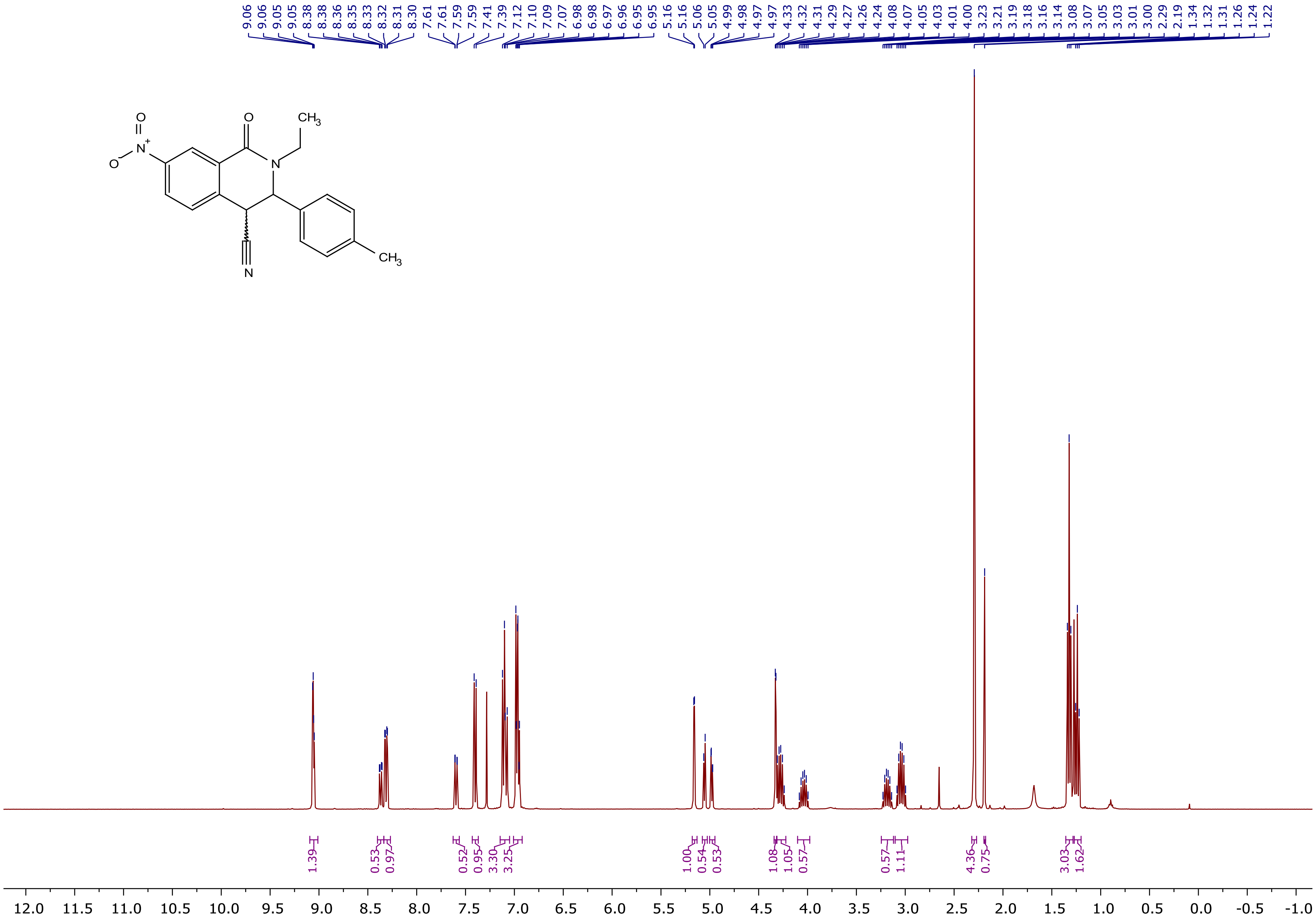

^{13}C NMR spectrum of compound 18k

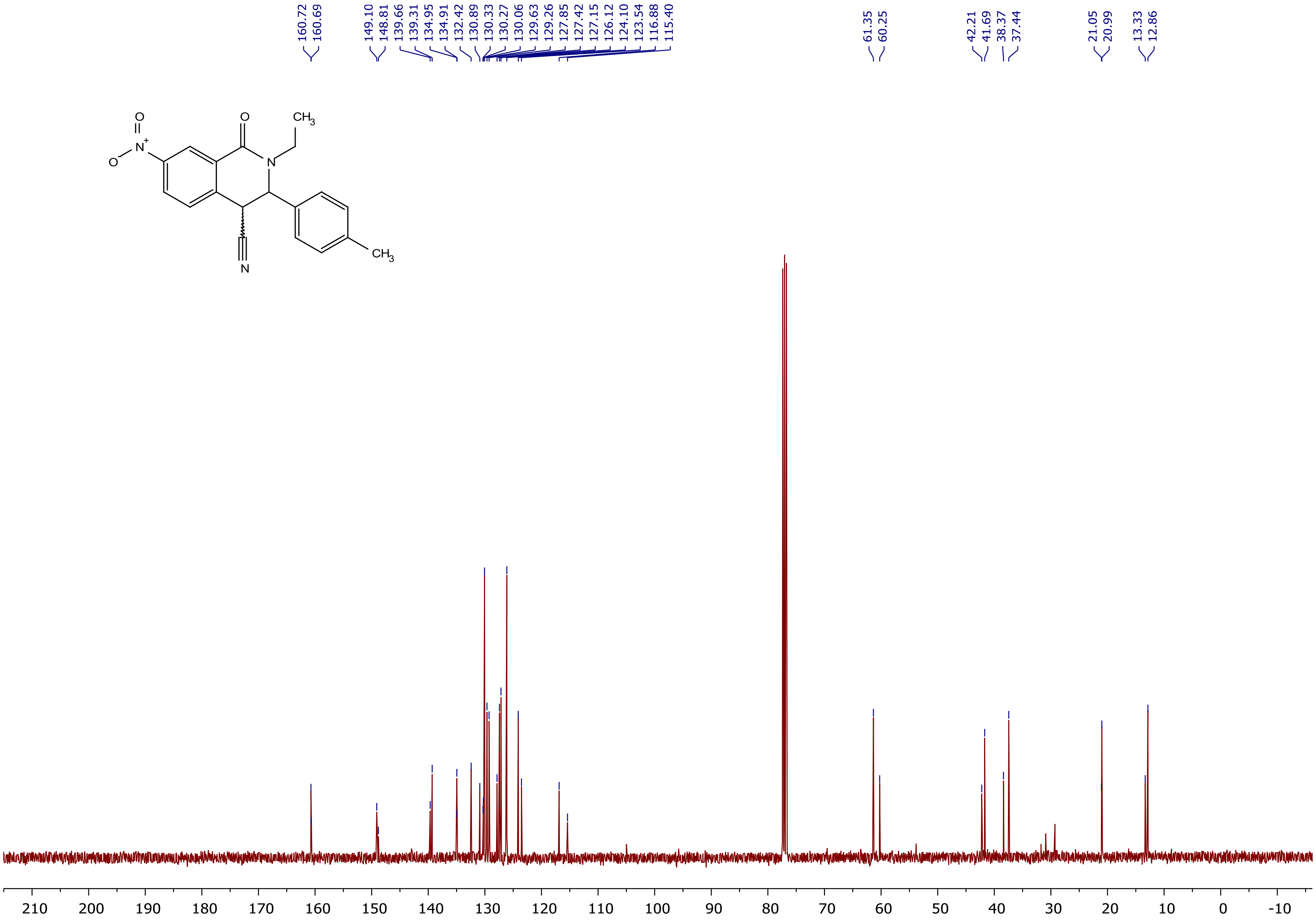

^{1}H NMR spectrum of compound 18l

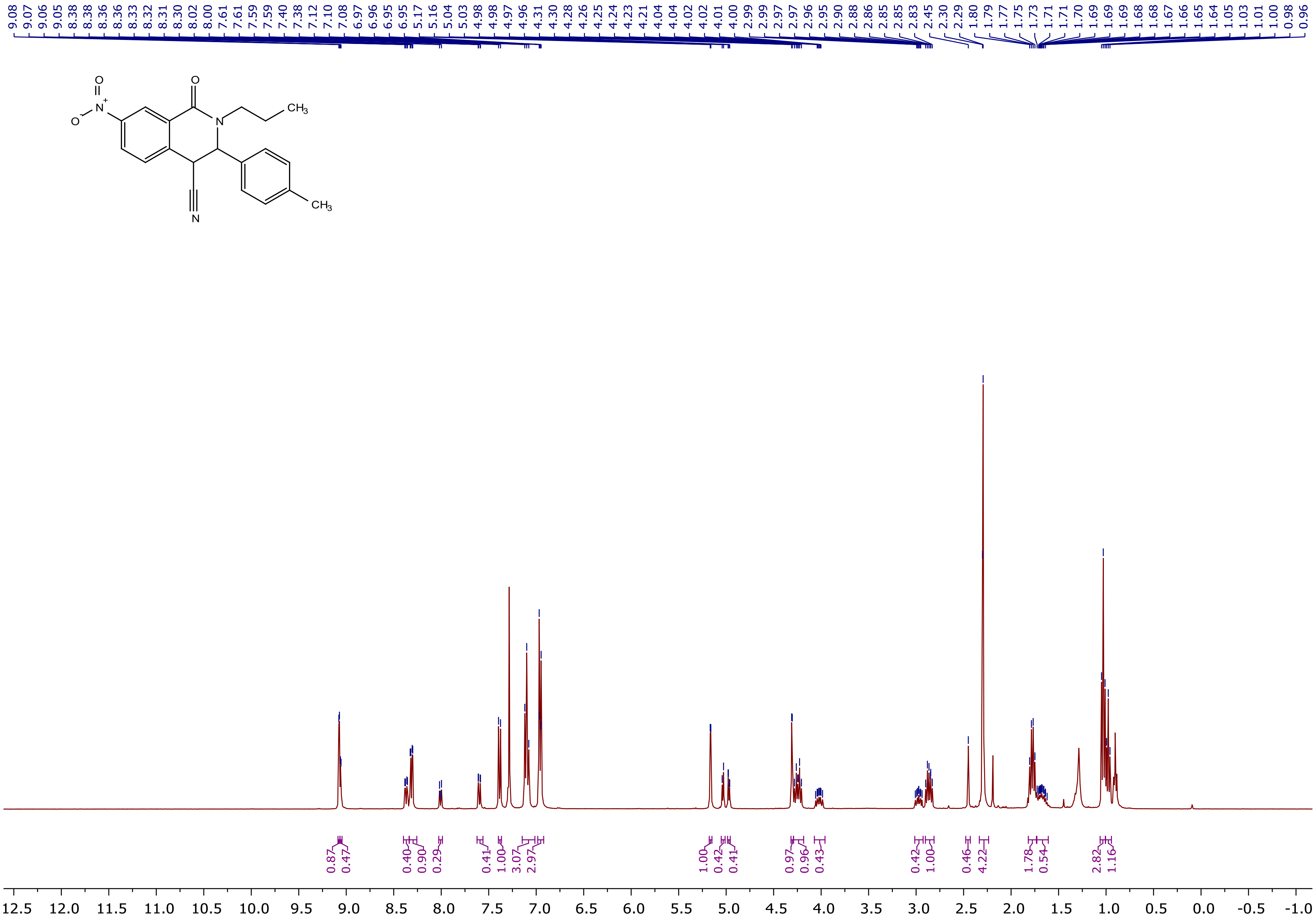

^{13}C NMR spectrum of compound 18l

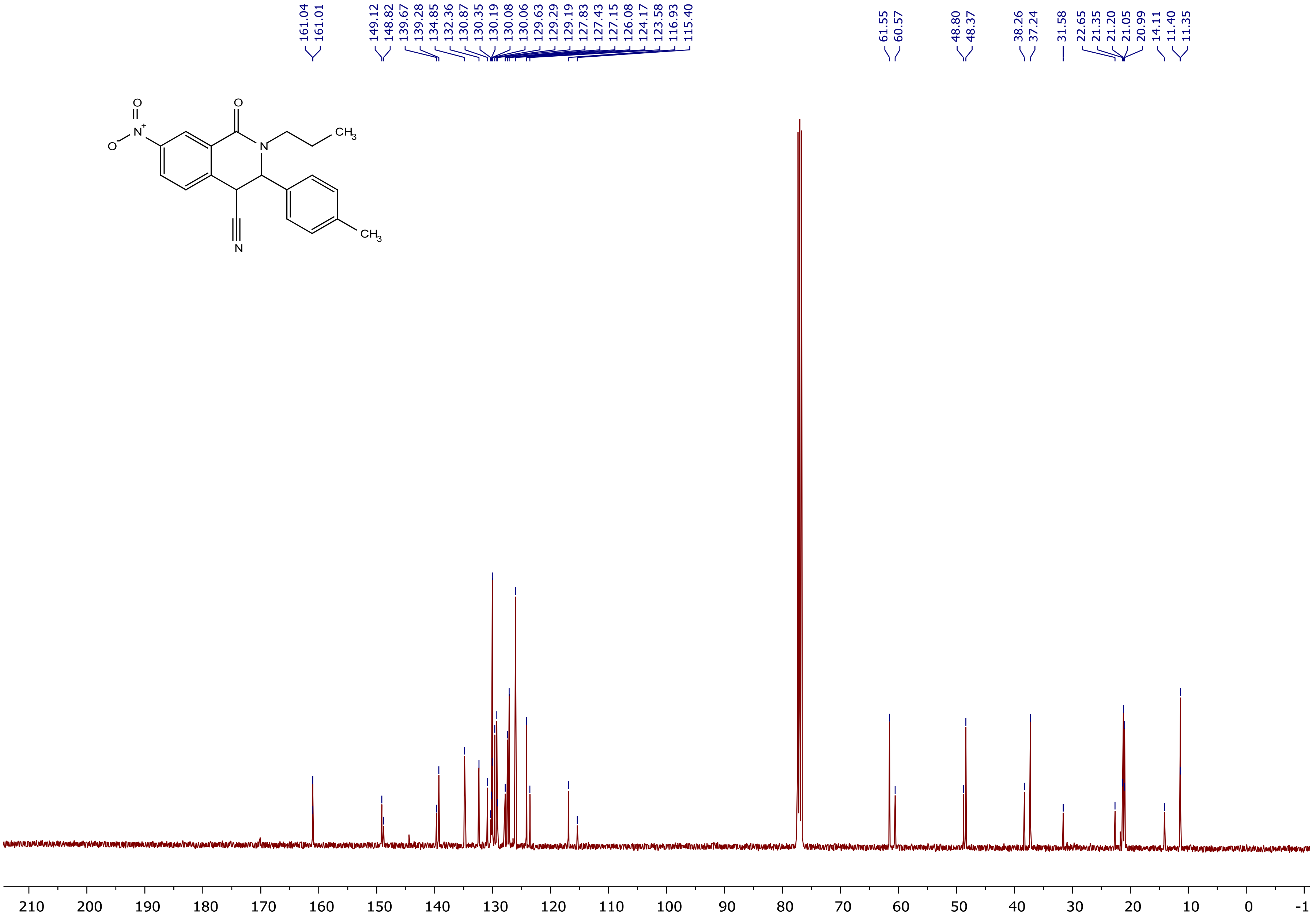

^{1}H NMR spectrum of compound 18m

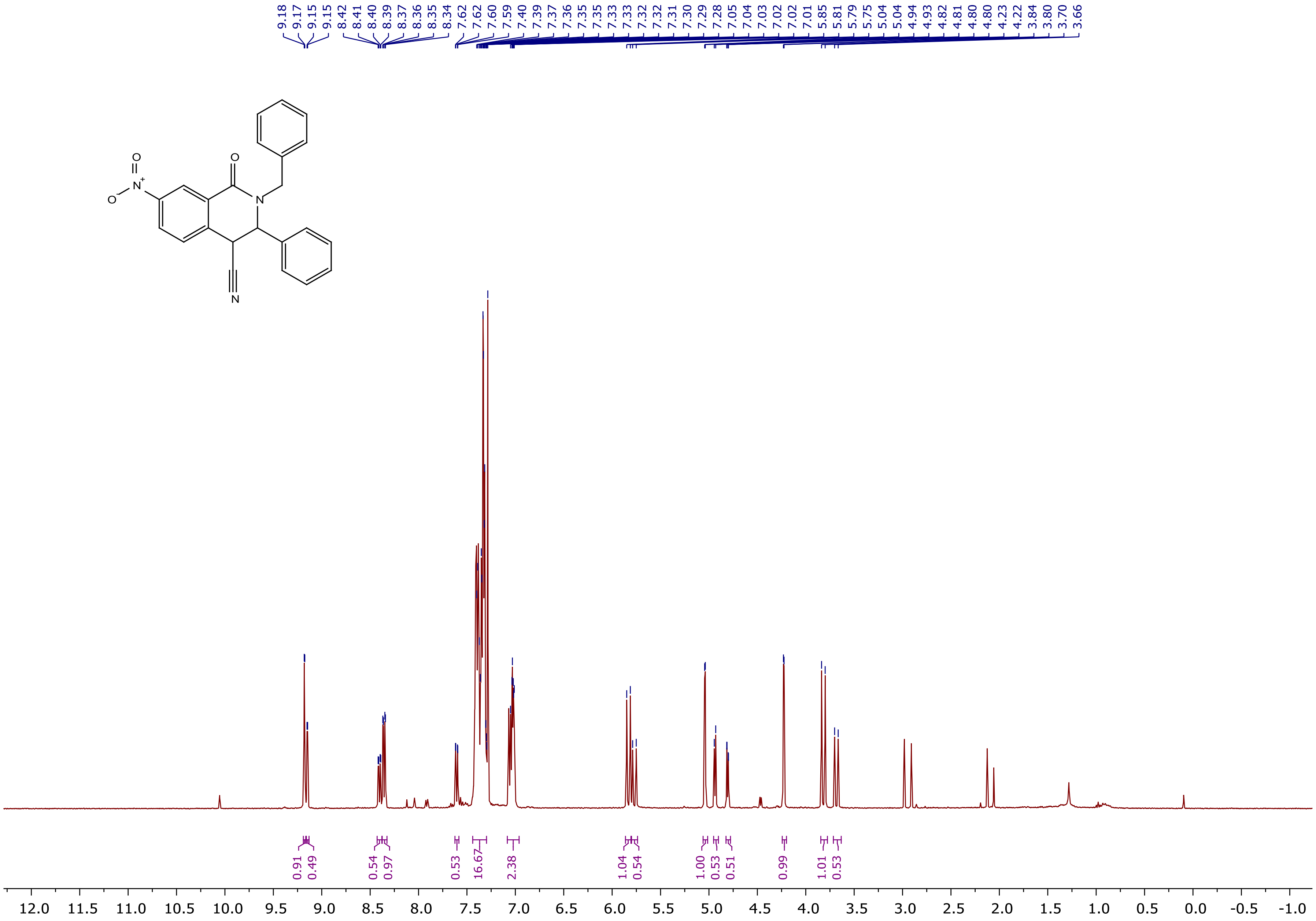

^{13}C NMR spectrum of compound 18m

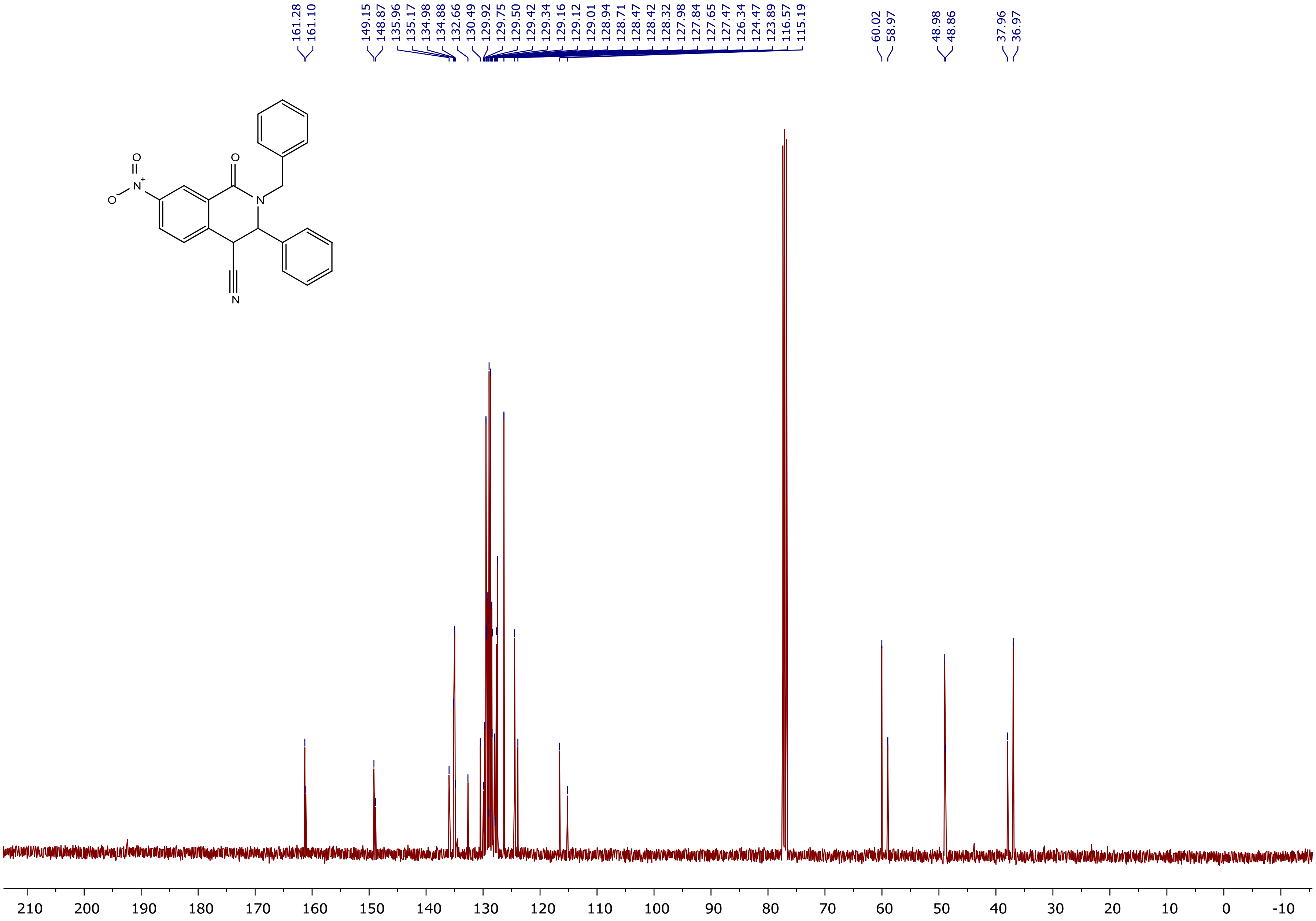

^{1}H NMR spectrum of compound 18n

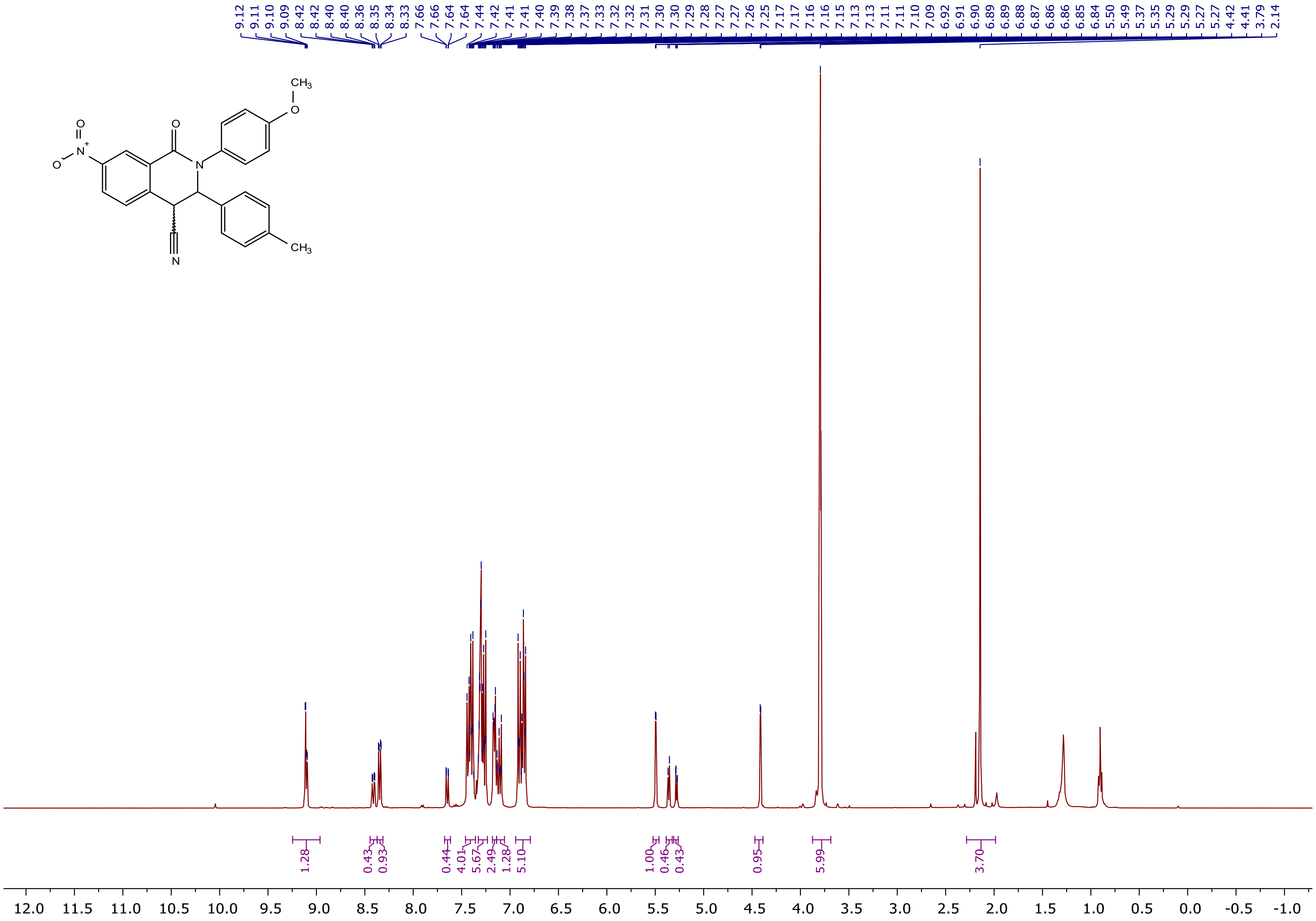

^{13}C NMR spectrum of compound 18n

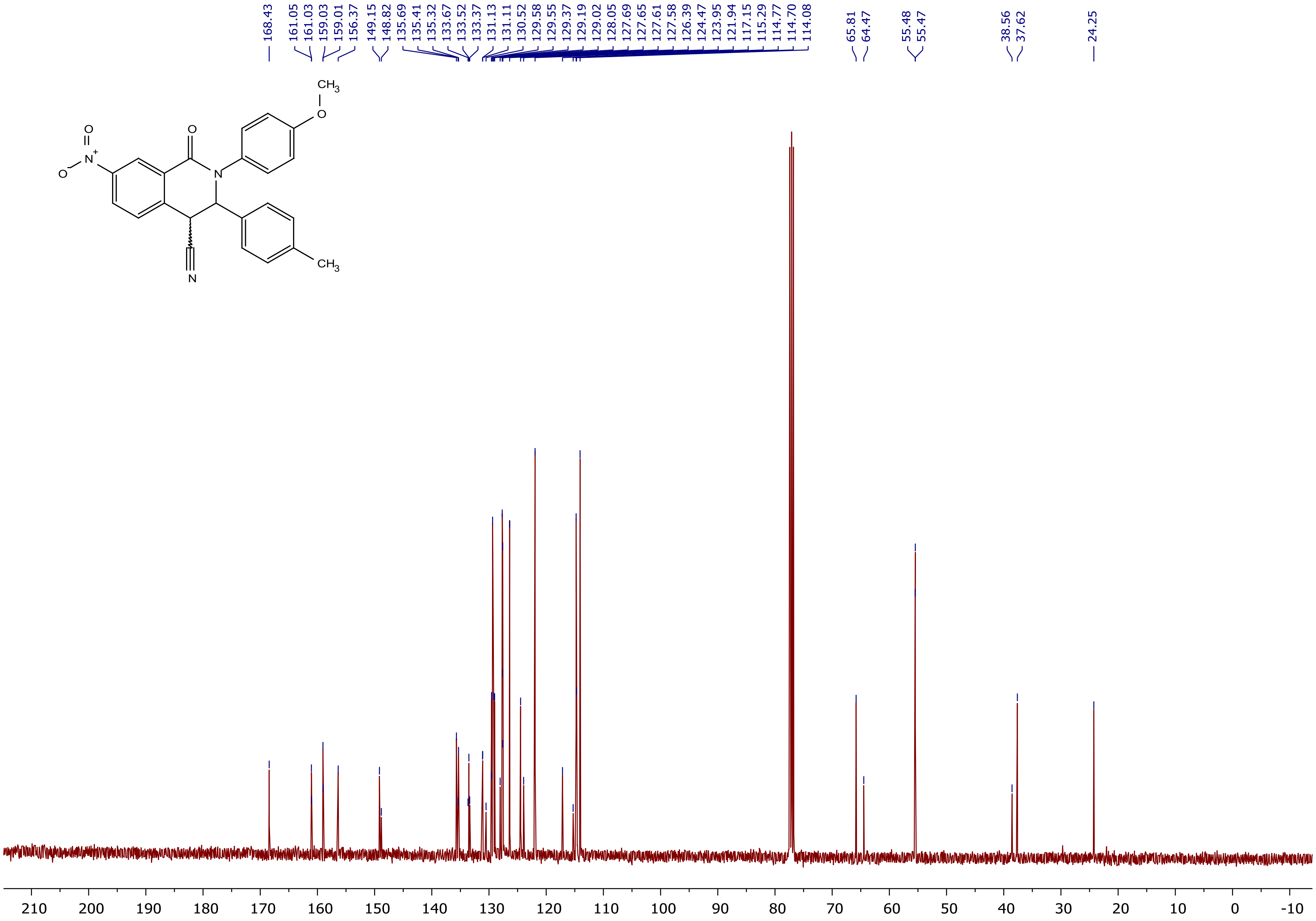

^{1}H NMR spectrum of compound 18o

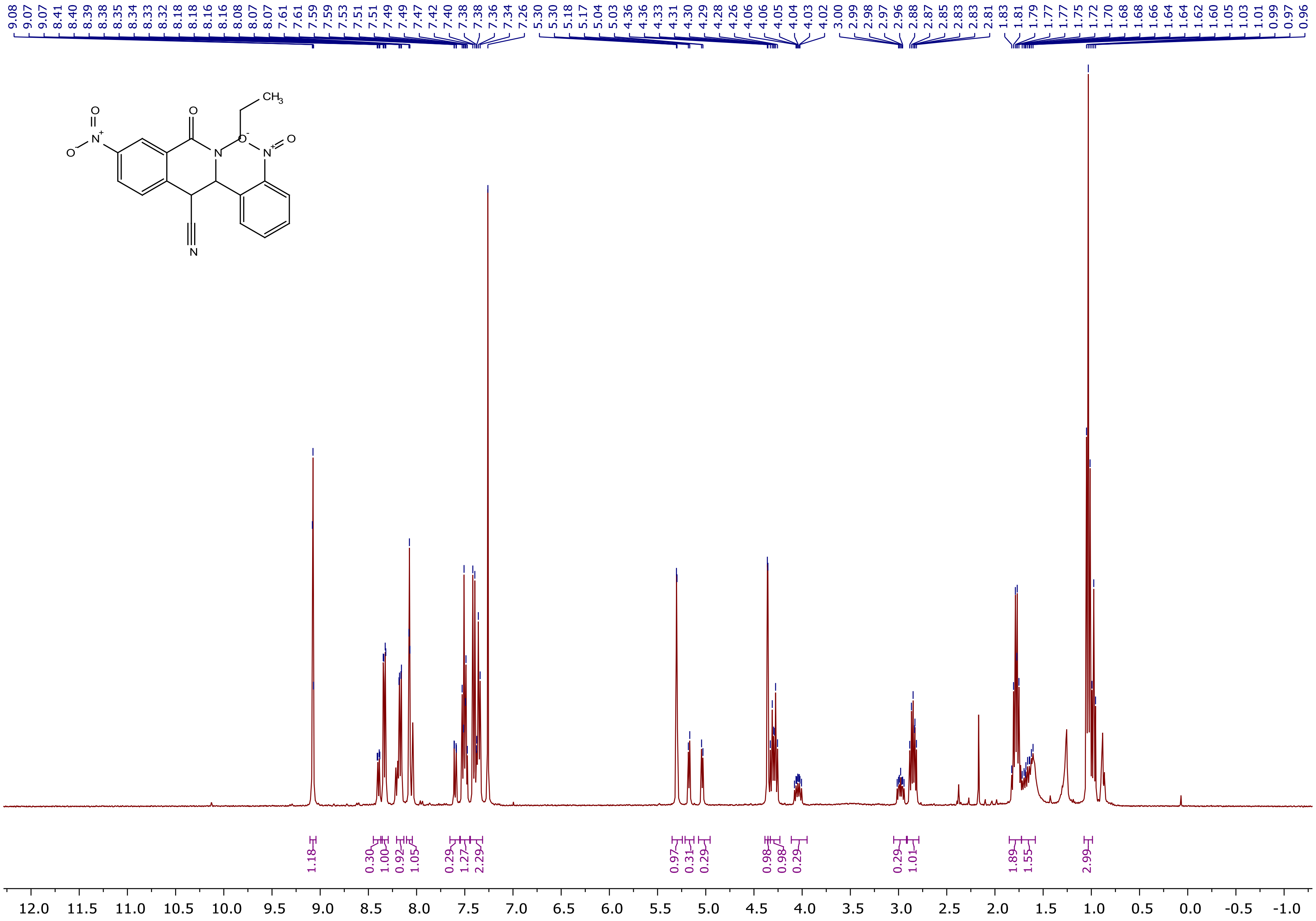

^{13}C NMR spectrum of compound 18o

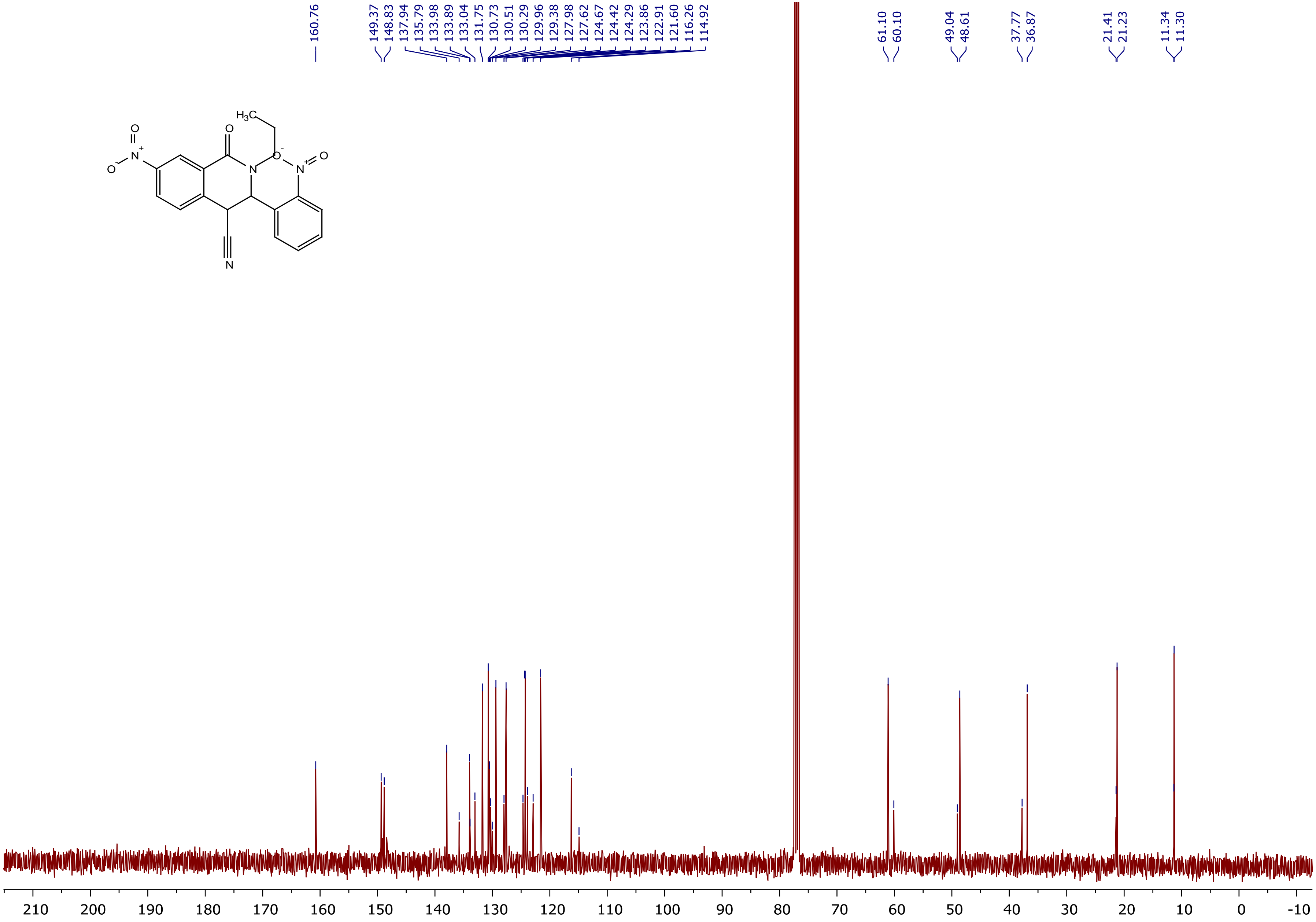

^{1}H NMR spectrum of compound 18p

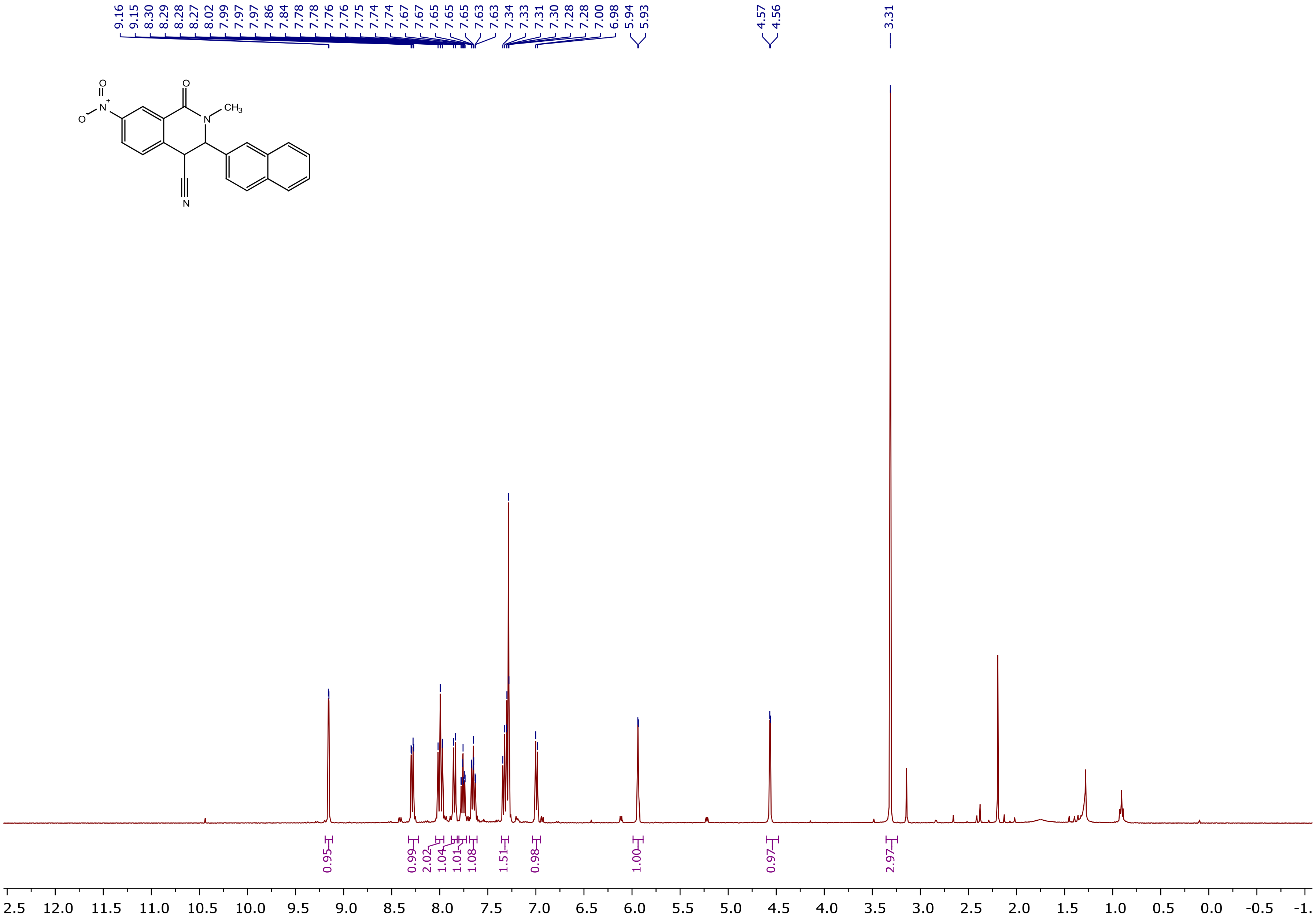

^{13}C NMR spectrum of compound 18p

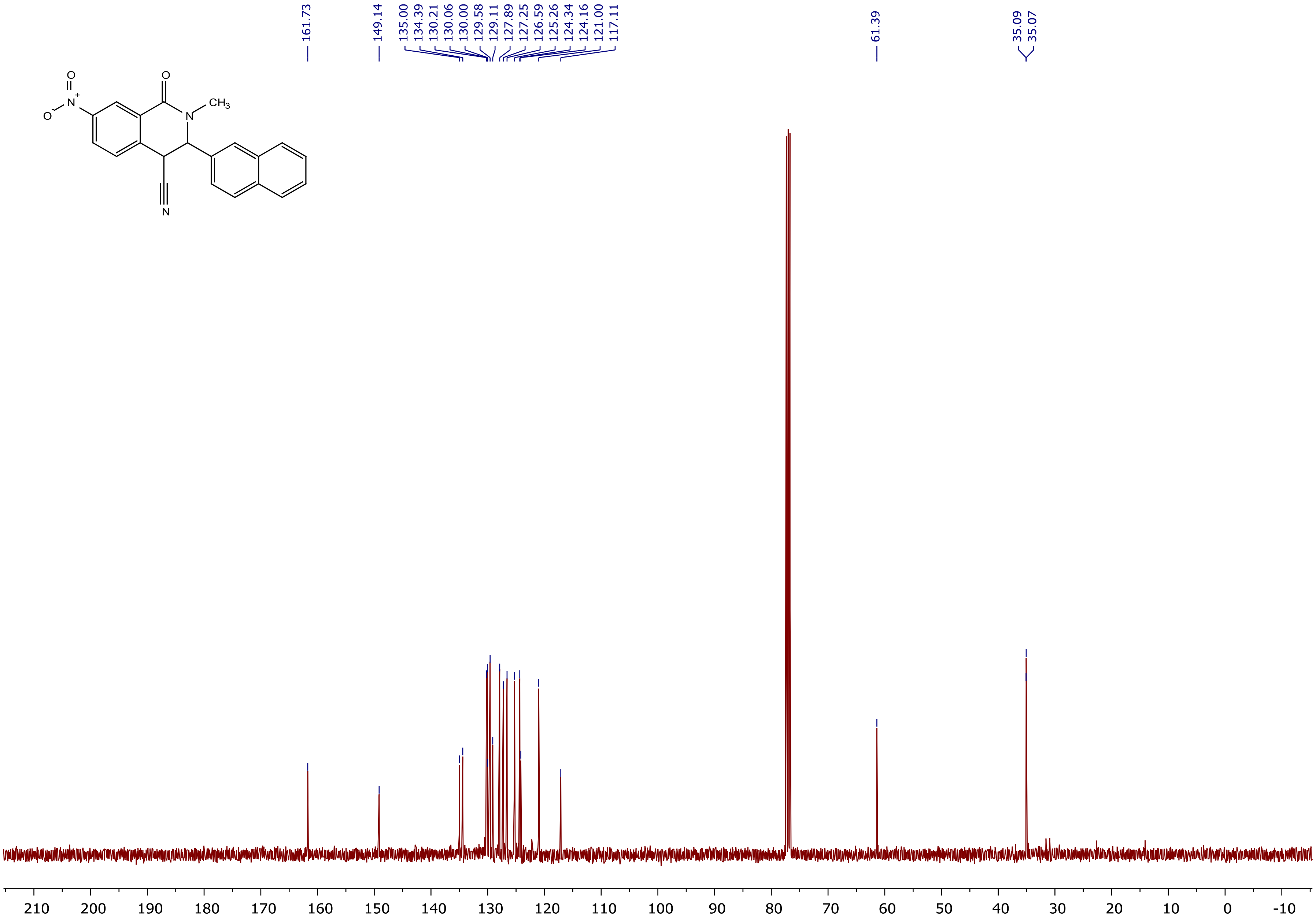

^{1}H NMR spectrum of compound 18q

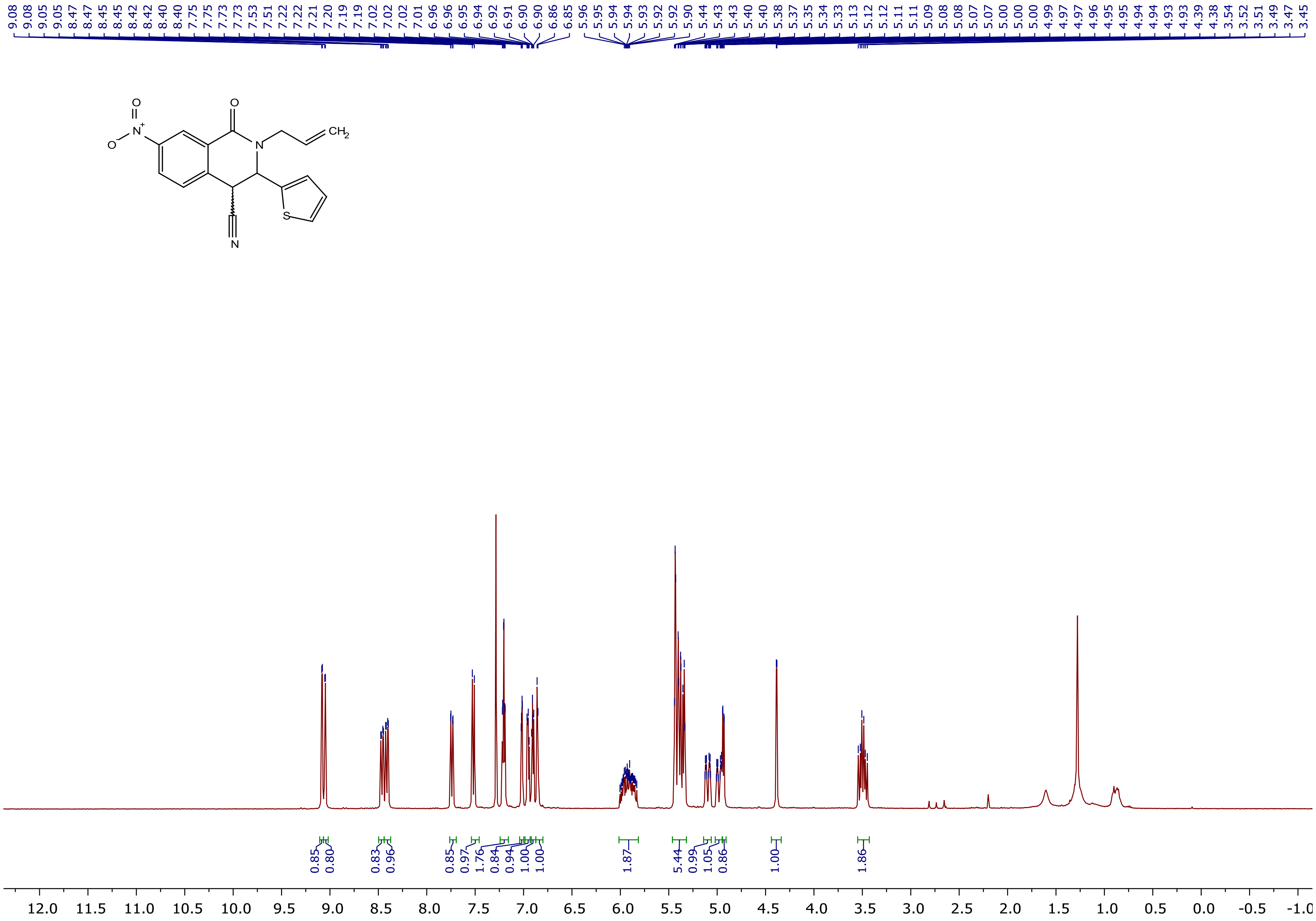

^{13}C NMR spectrum of compound 18q

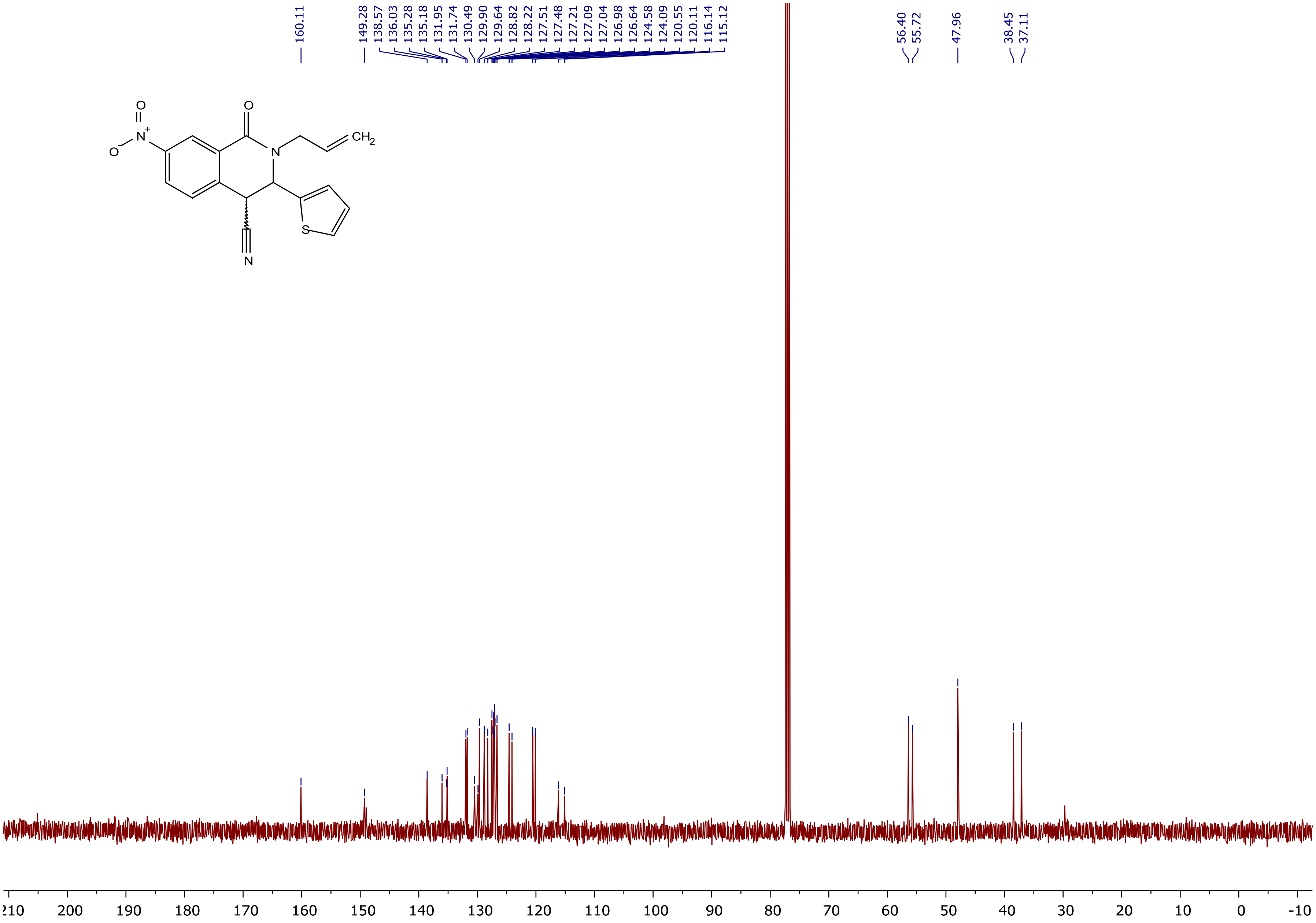

^{1}H NMR spectrum of compound 19a

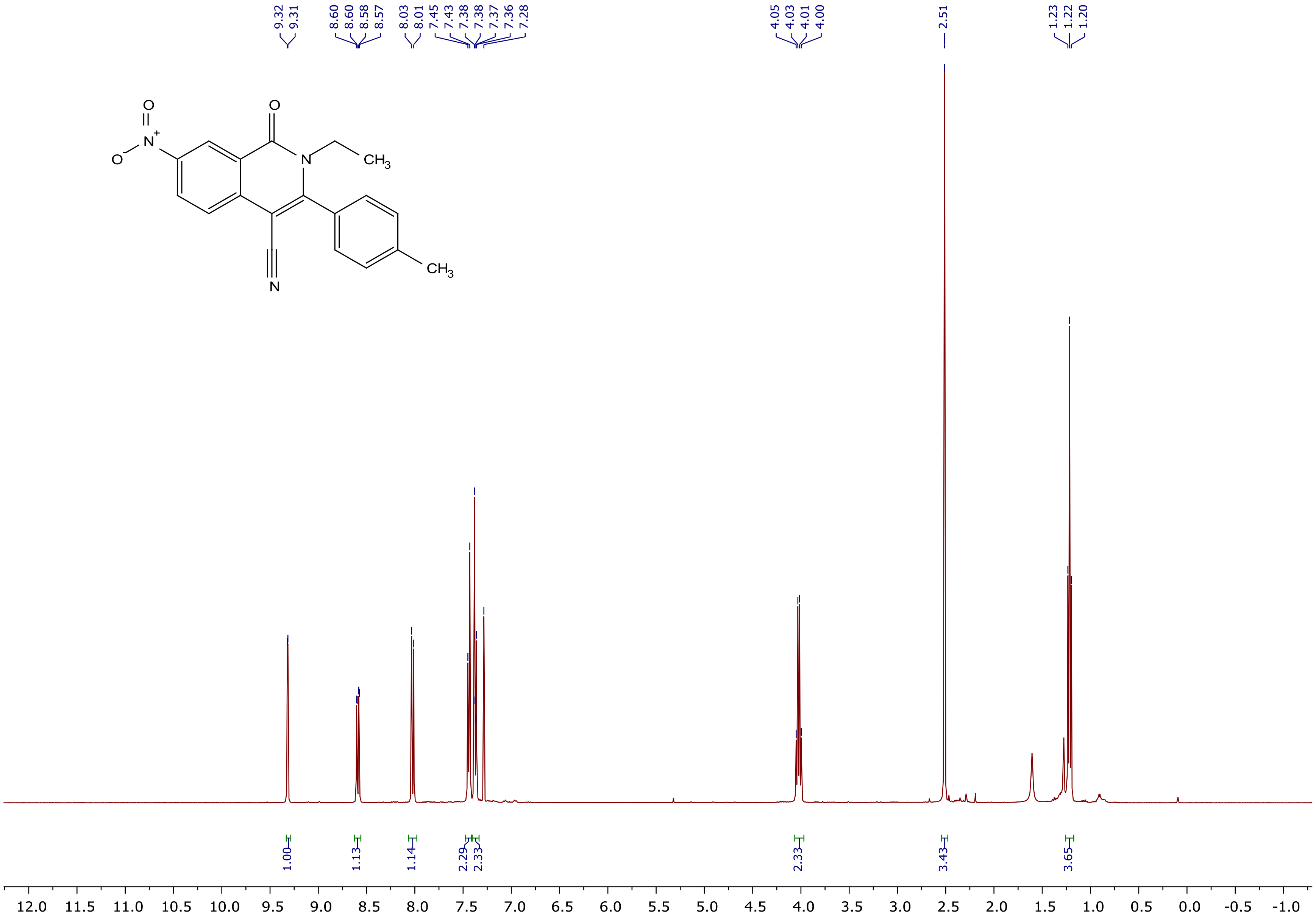

^{13}C NMR spectrum of compound 19a

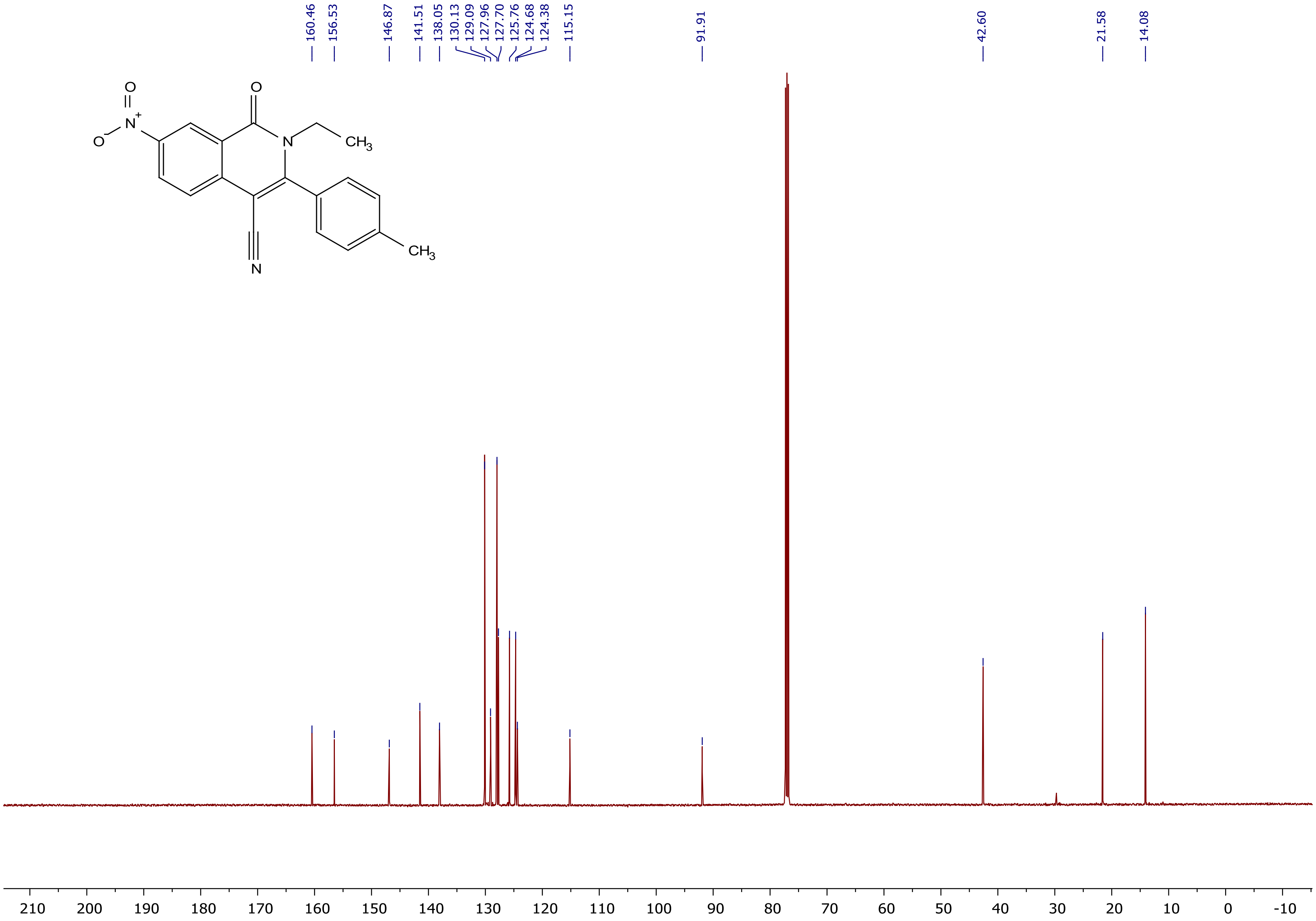

^{1}H NMR spectrum of compound 19b

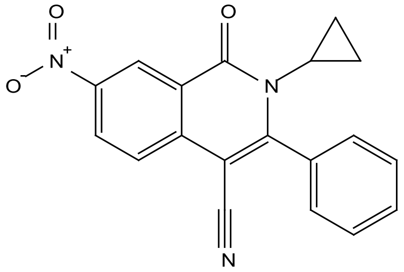

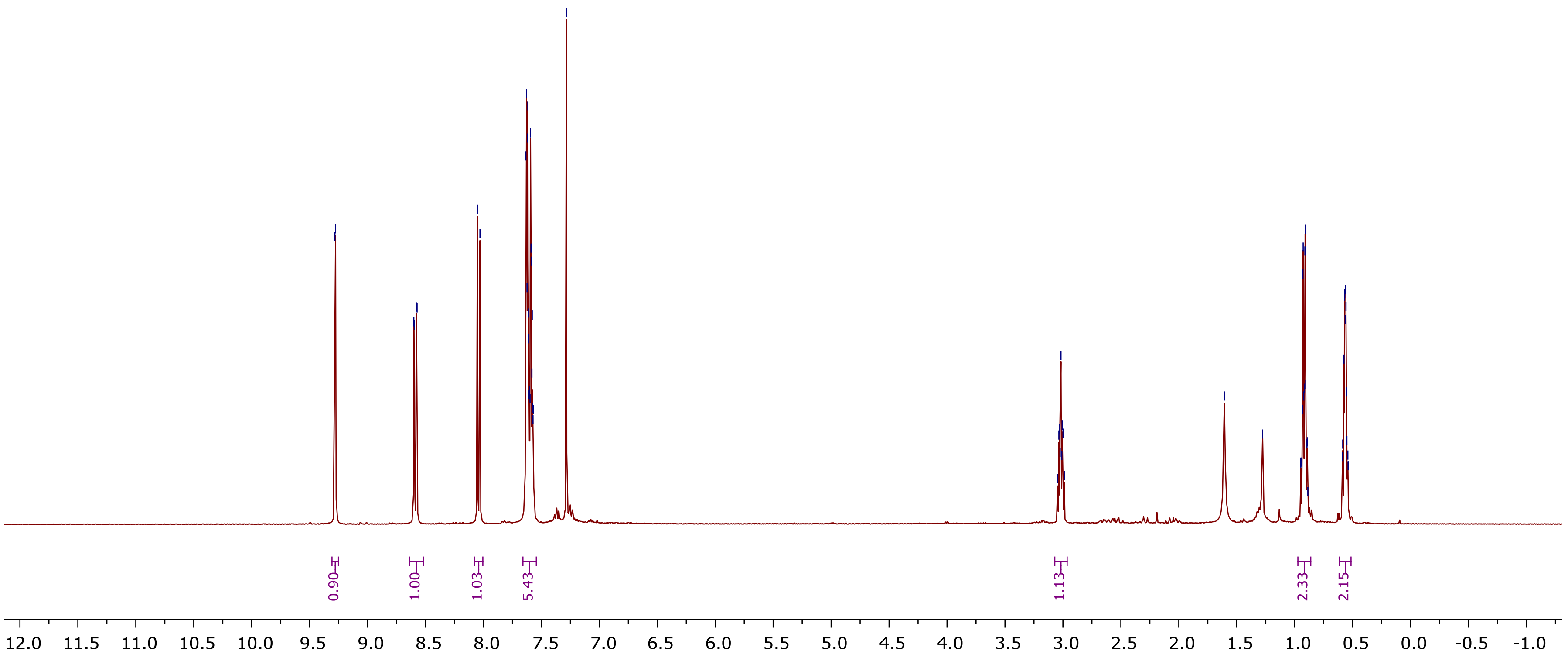

^{13}C NMR spectrum of compound 19b

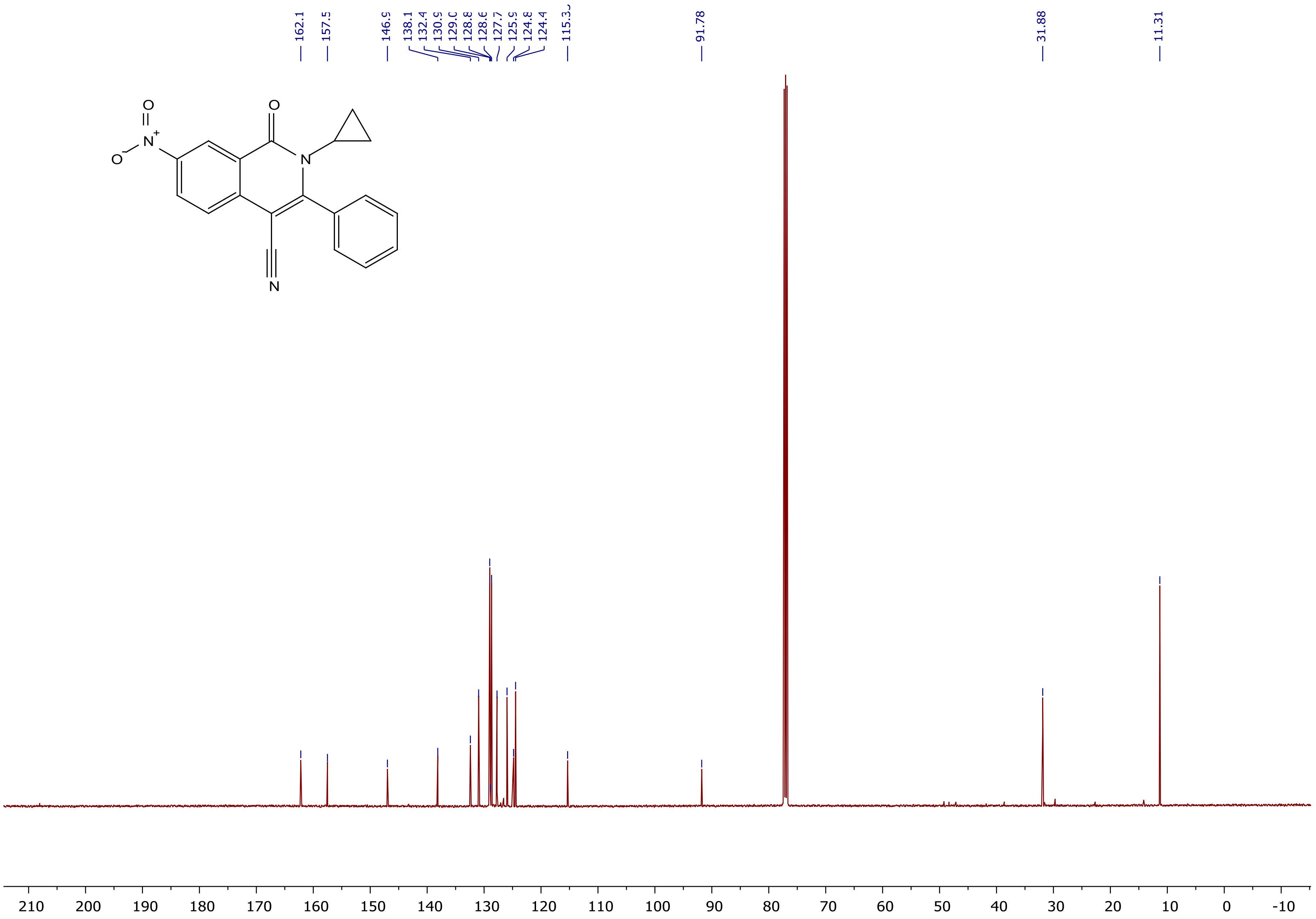

^{1}H NMR spectrum of compound 20

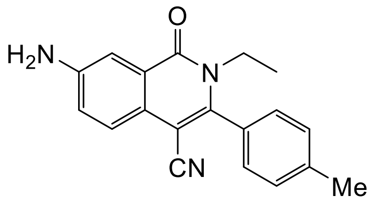

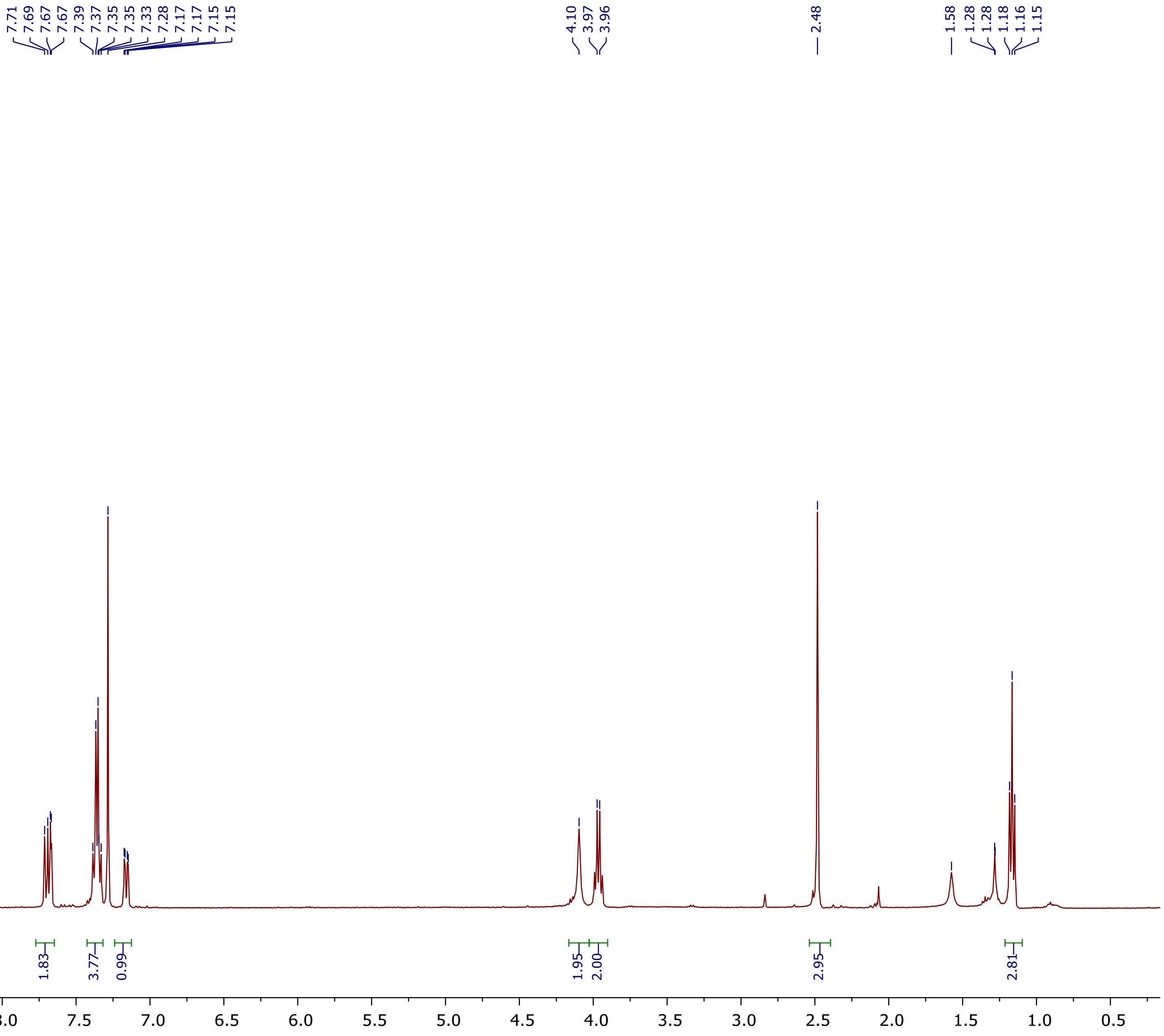

^{13}C NMR spectrum of compound 20

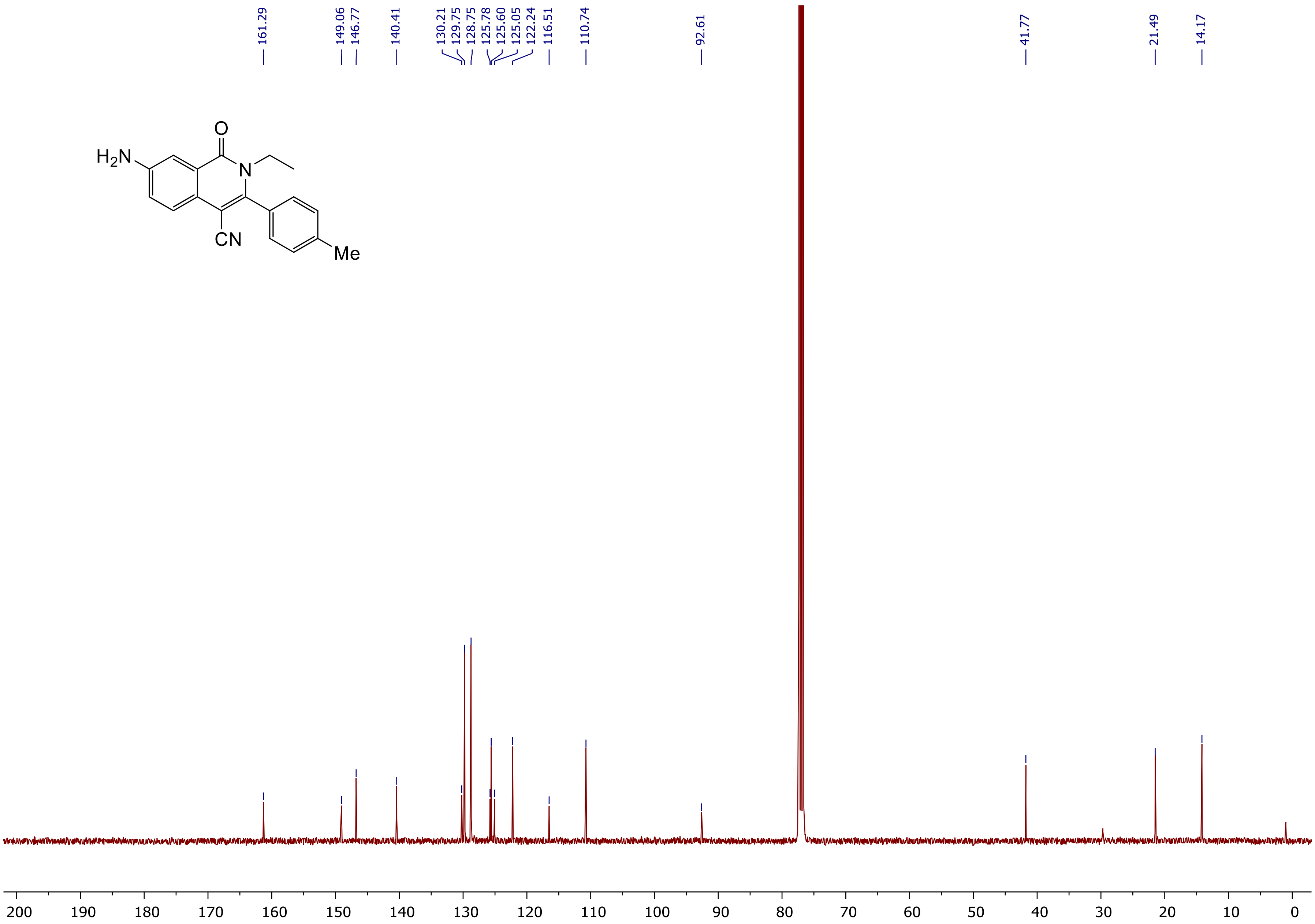

^{1}H NMR spectrum of compound 21

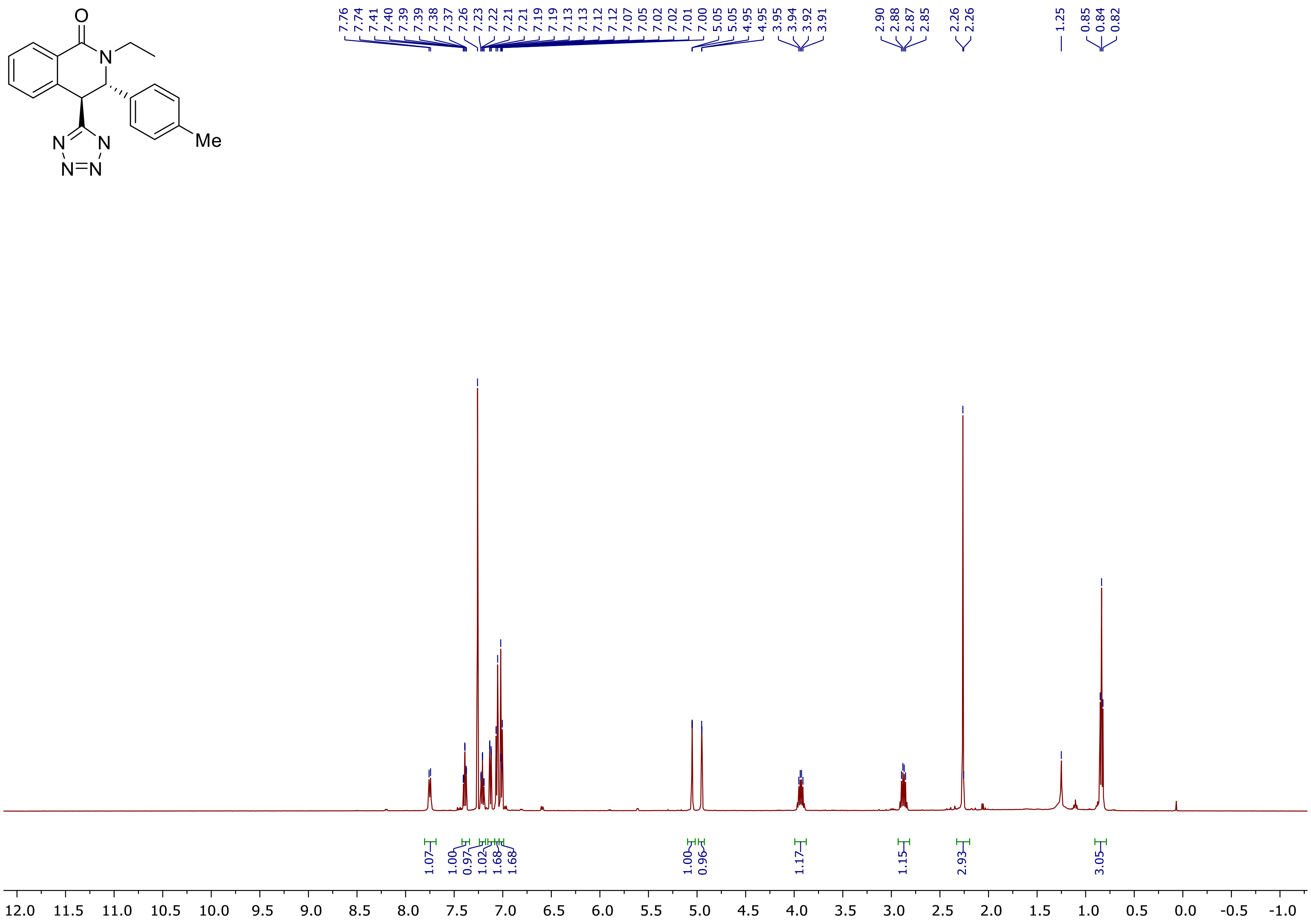

^{13}C NMR spectrum of compound 21

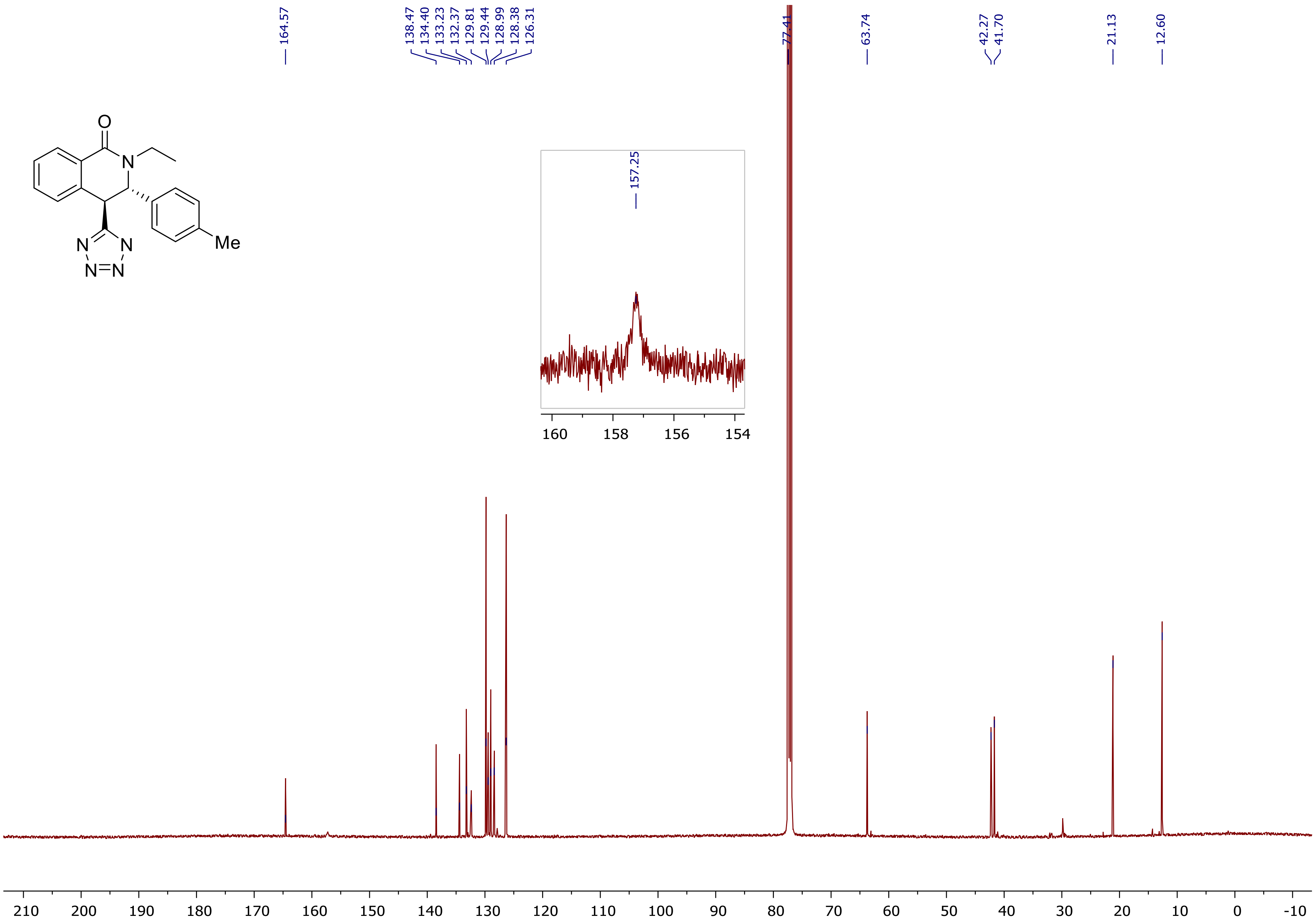

^{1}H NMR spectrum of compound 22a

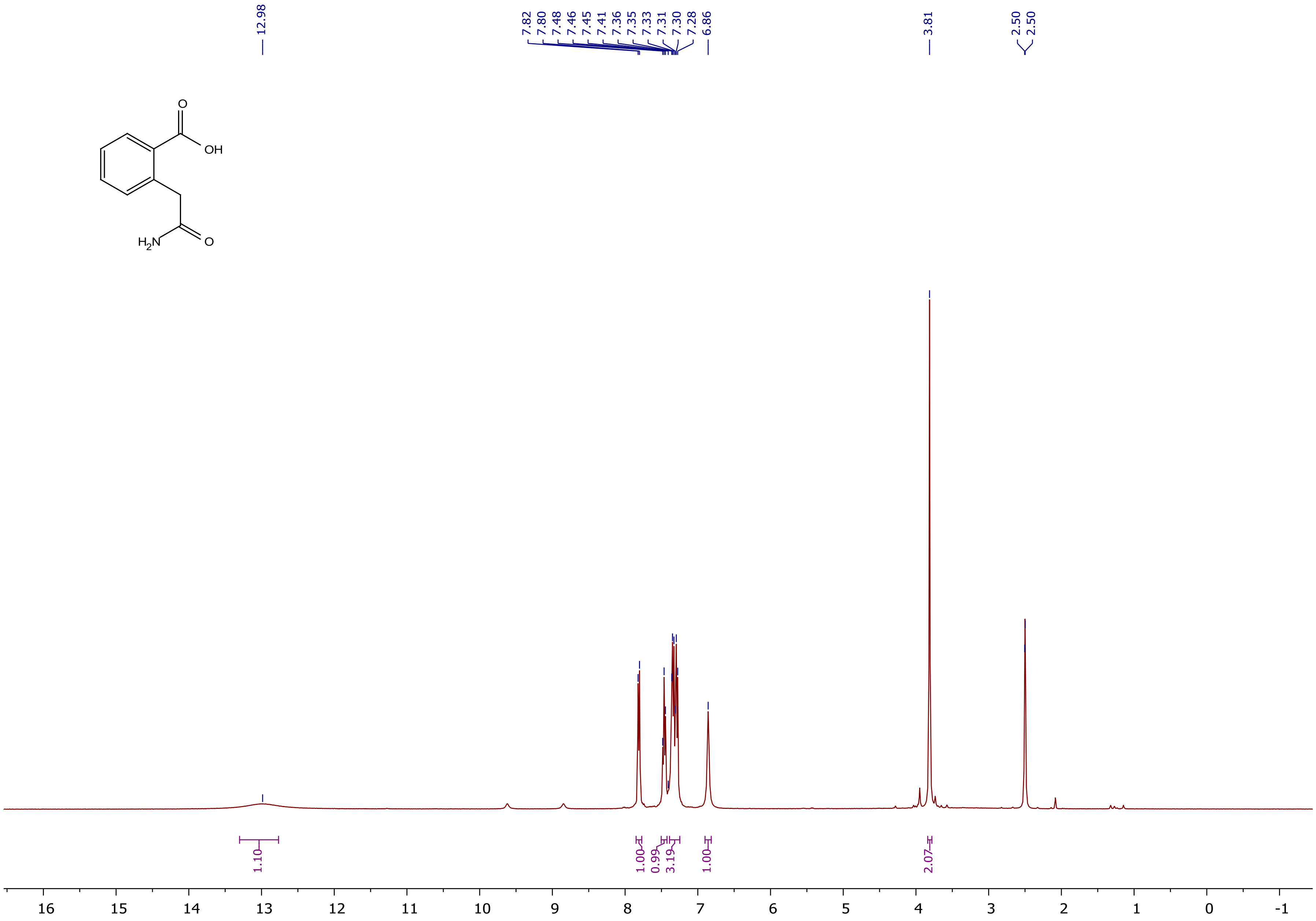

^{13}C NMR spectrum of compound 22a

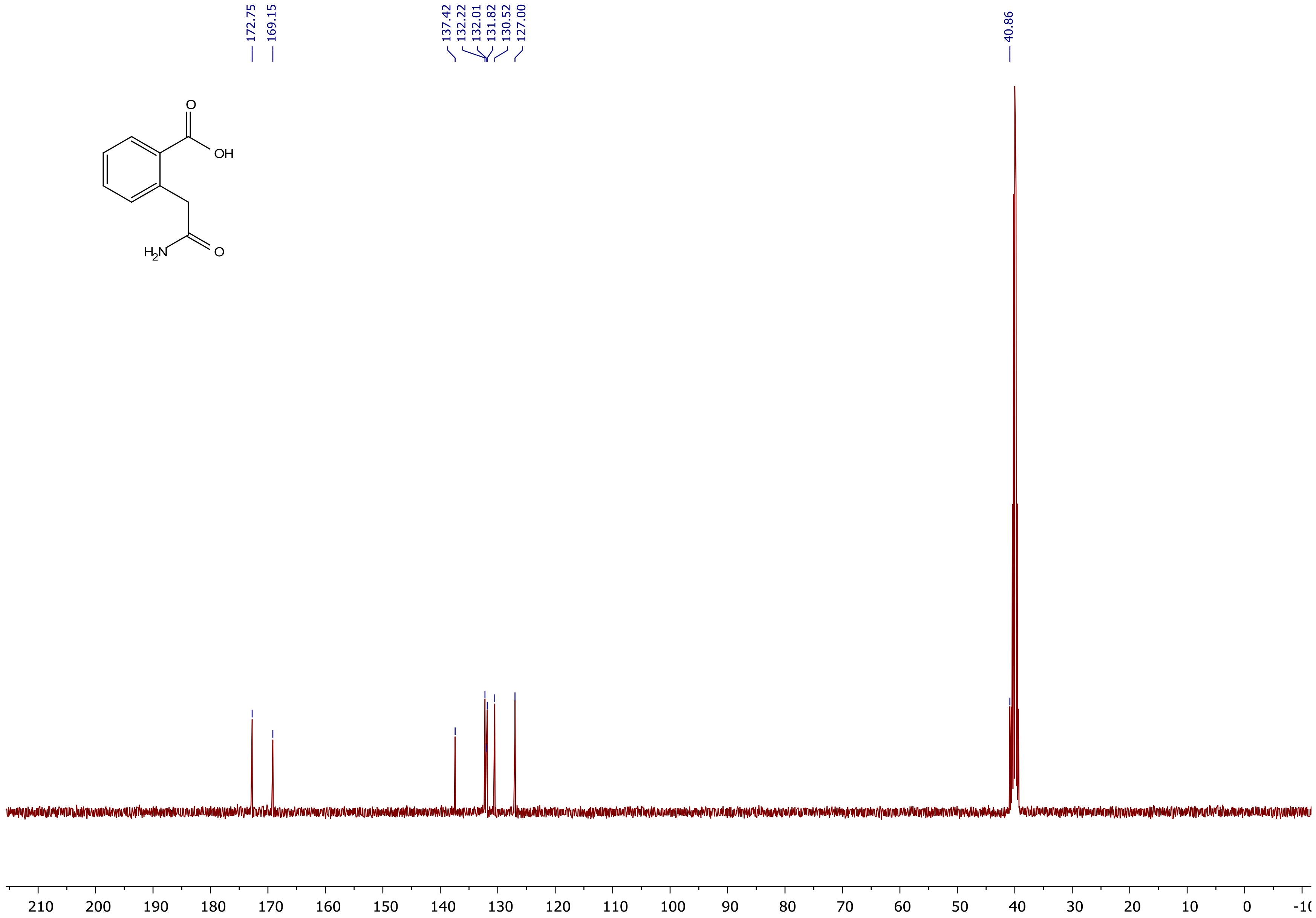

^{1}H NMR spectrum of compound 22b

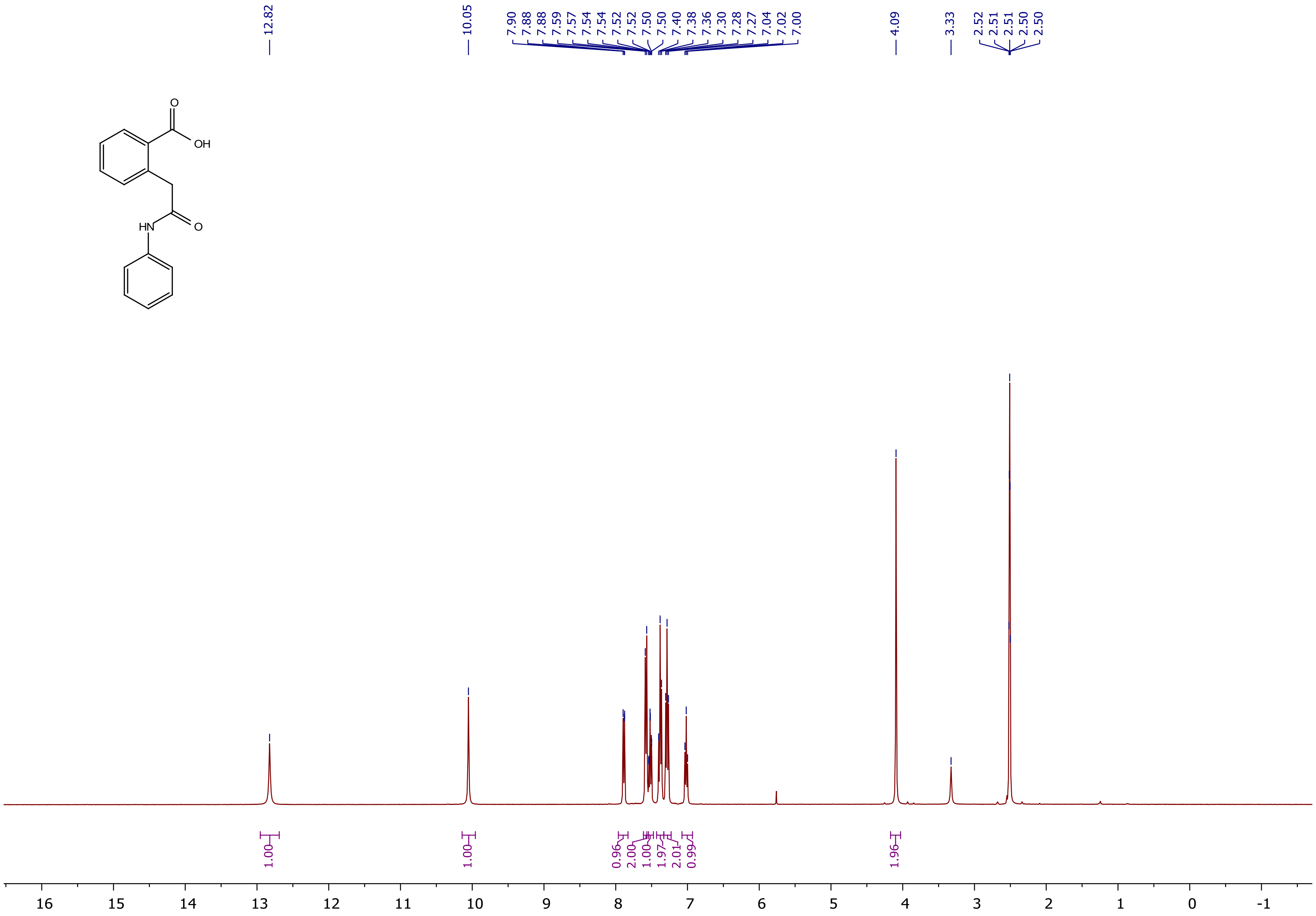

^{13}C NMR spectrum of compound 22b

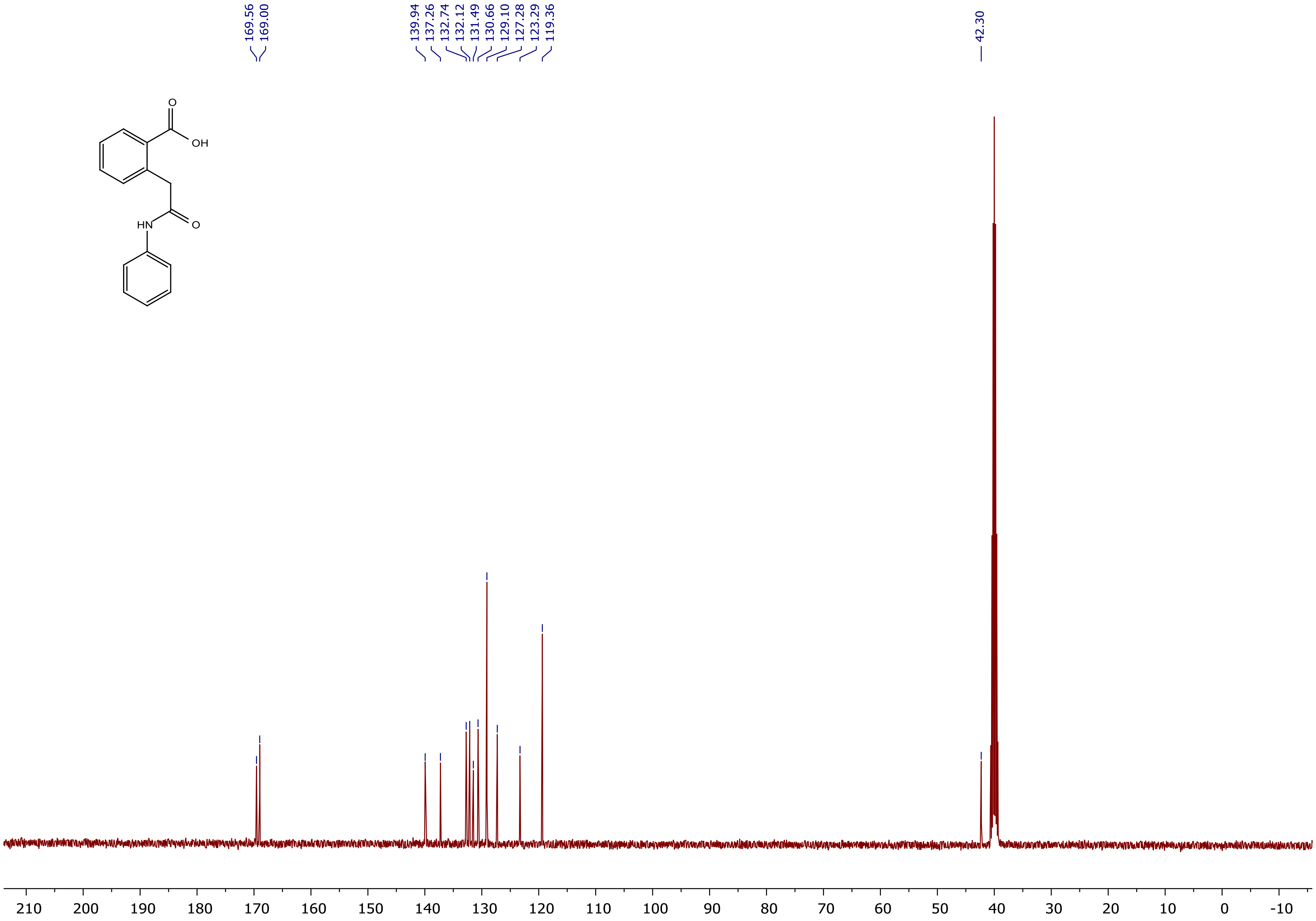

^{1}H NMR spectrum of compound 22c

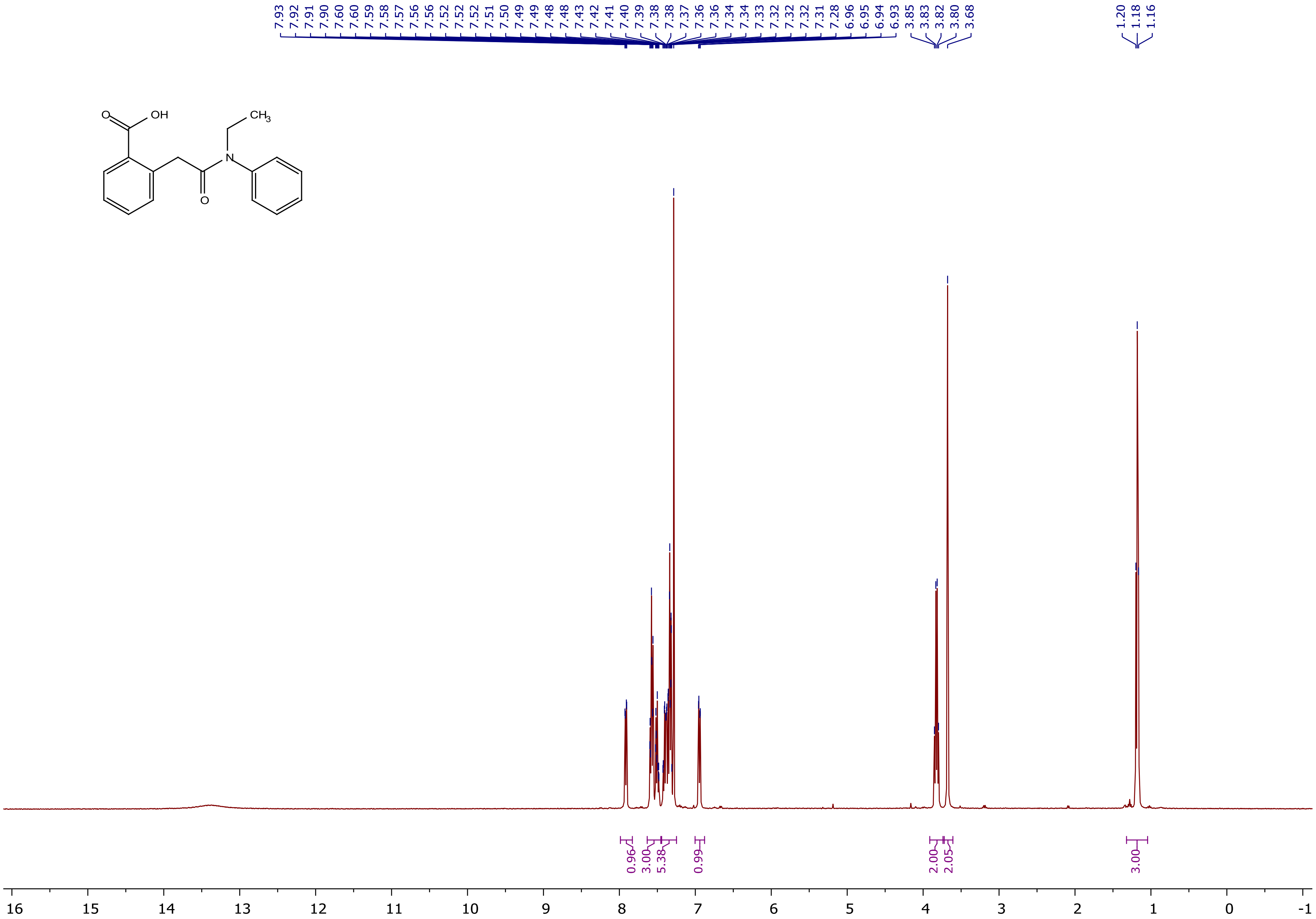

^{13}C NMR spectrum of compound 22c

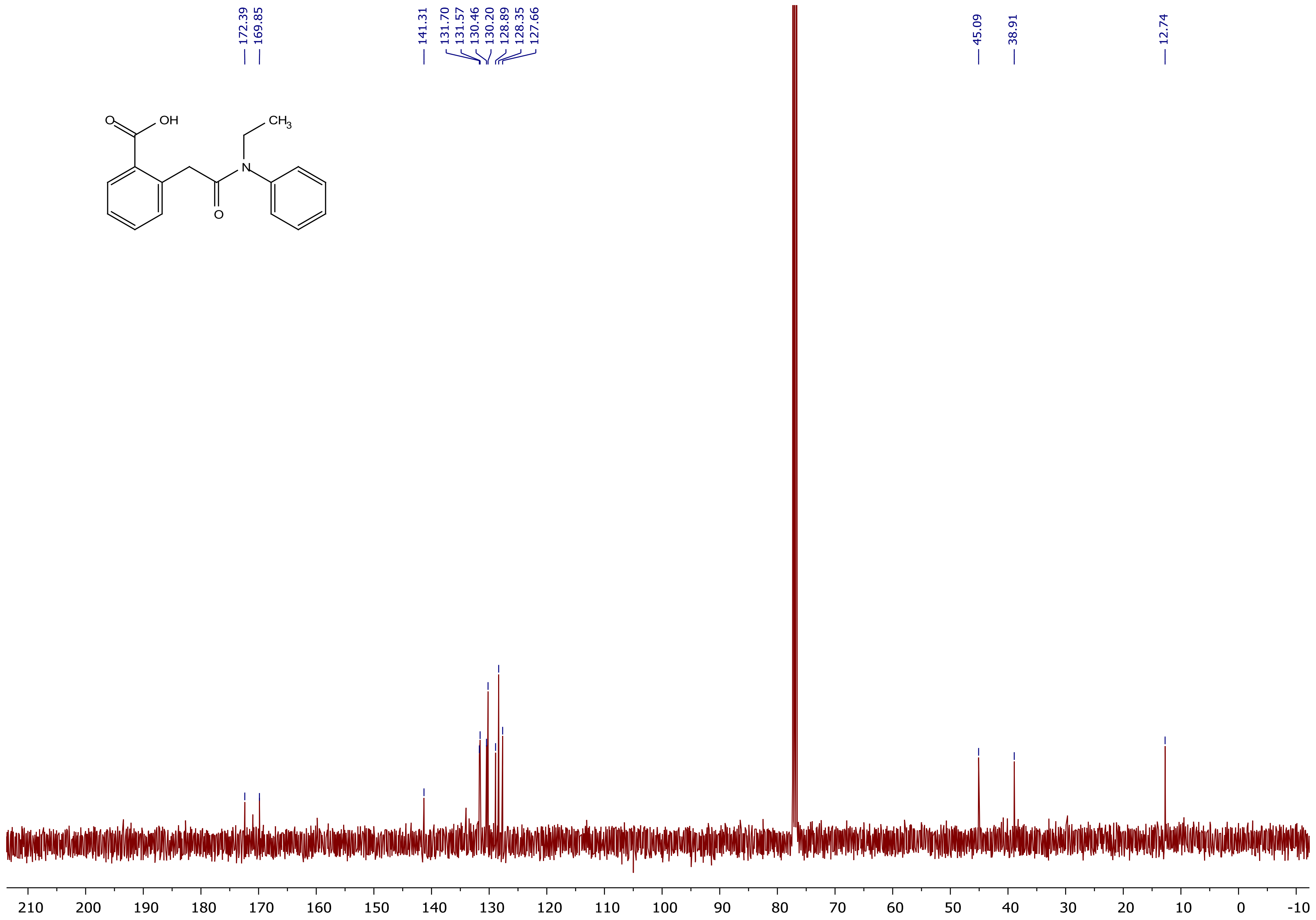

^{1}H NMR spectrum of compound 22d

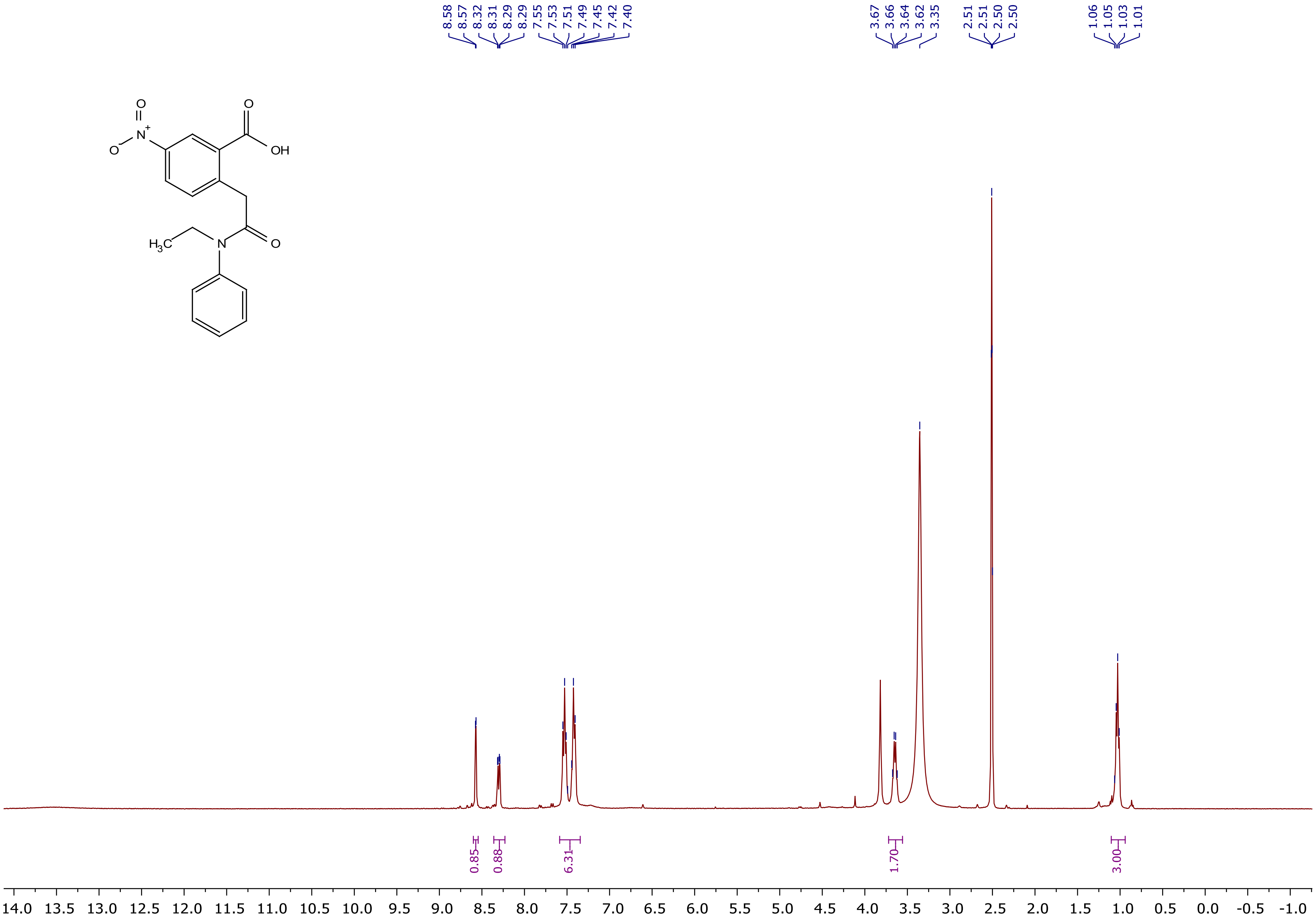

^{13}C NMR spectrum of compound 22d

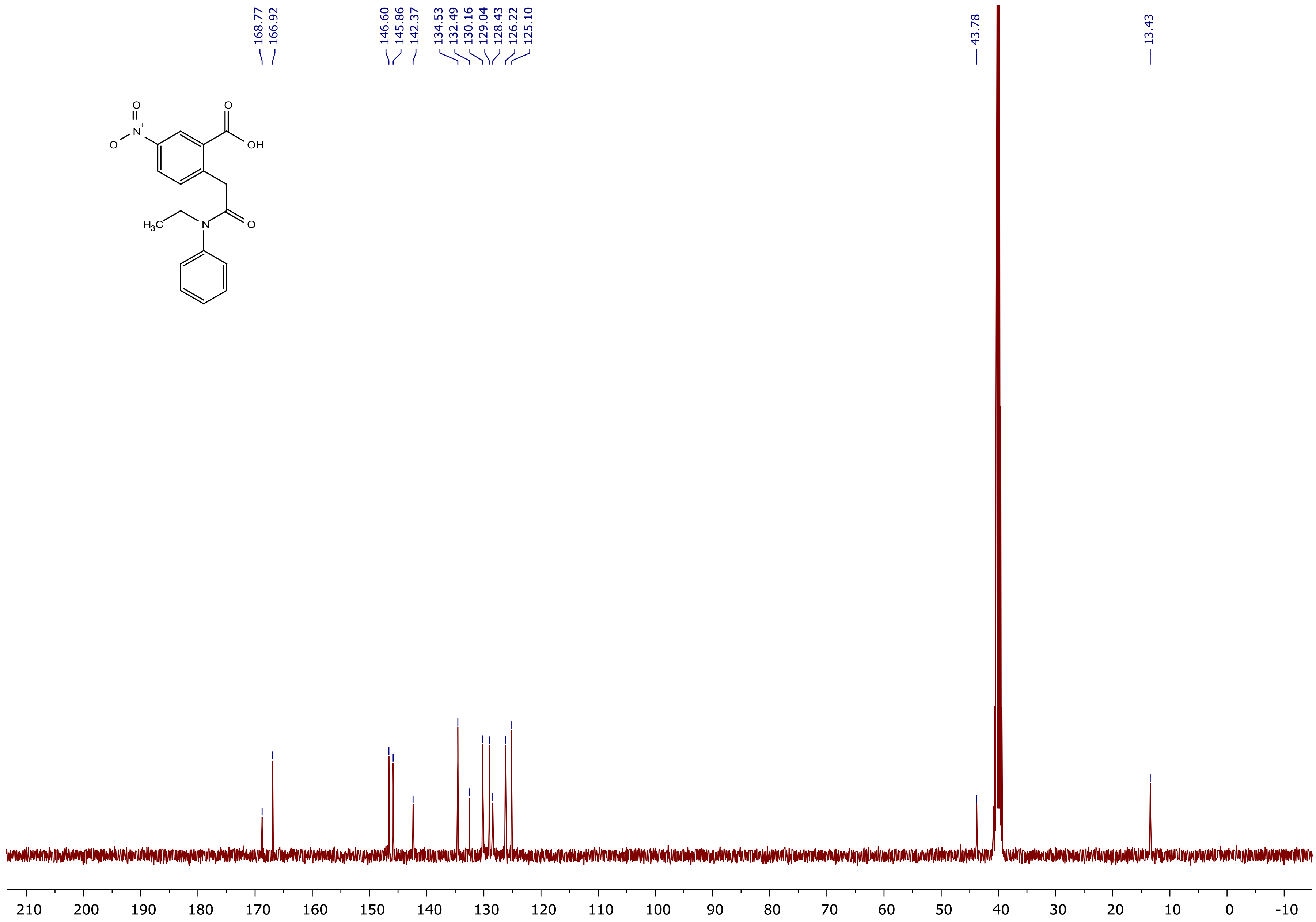

^{1}H NMR spectrum of compound 23a

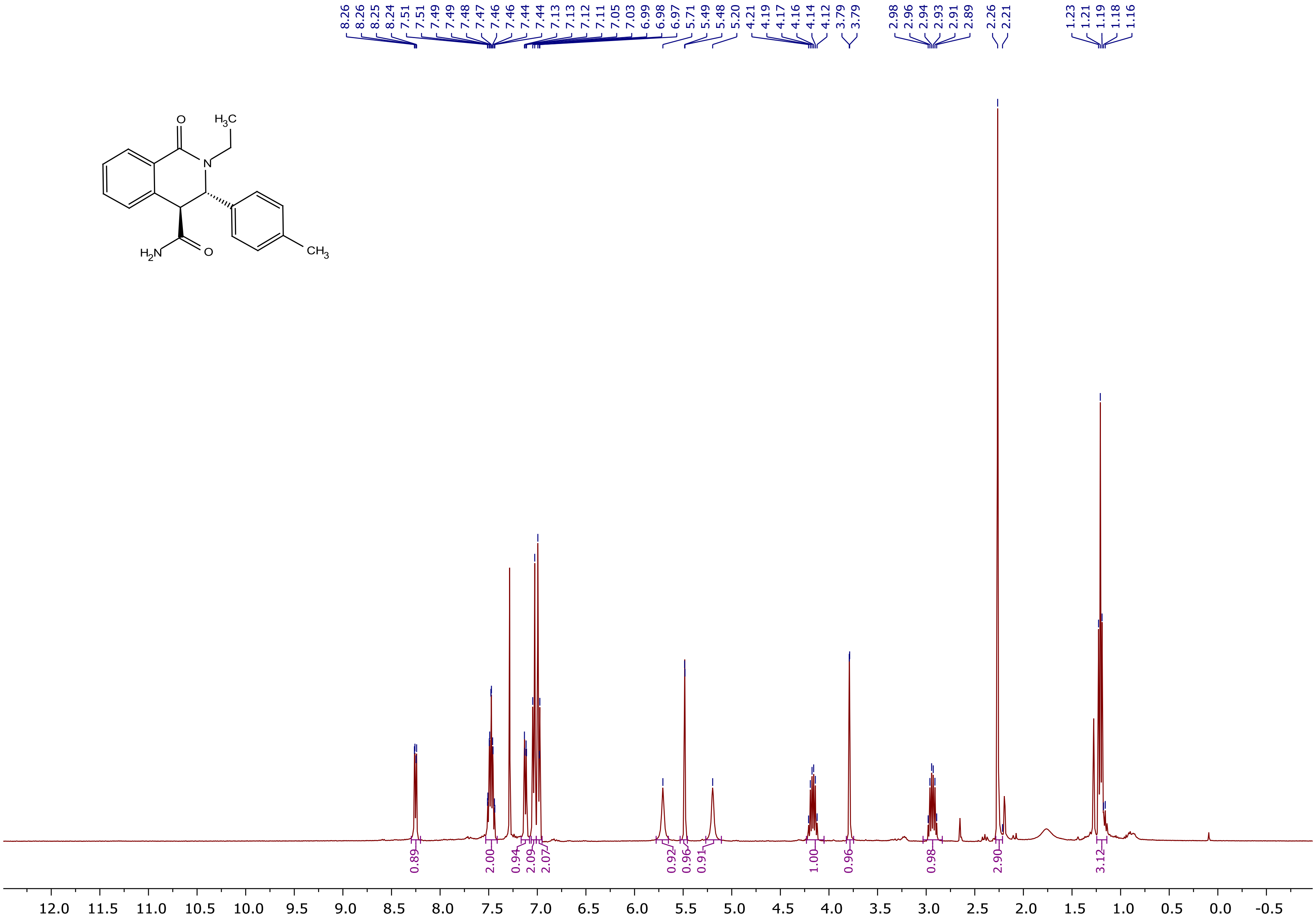

^{13}C NMR spectrum of compound 23a

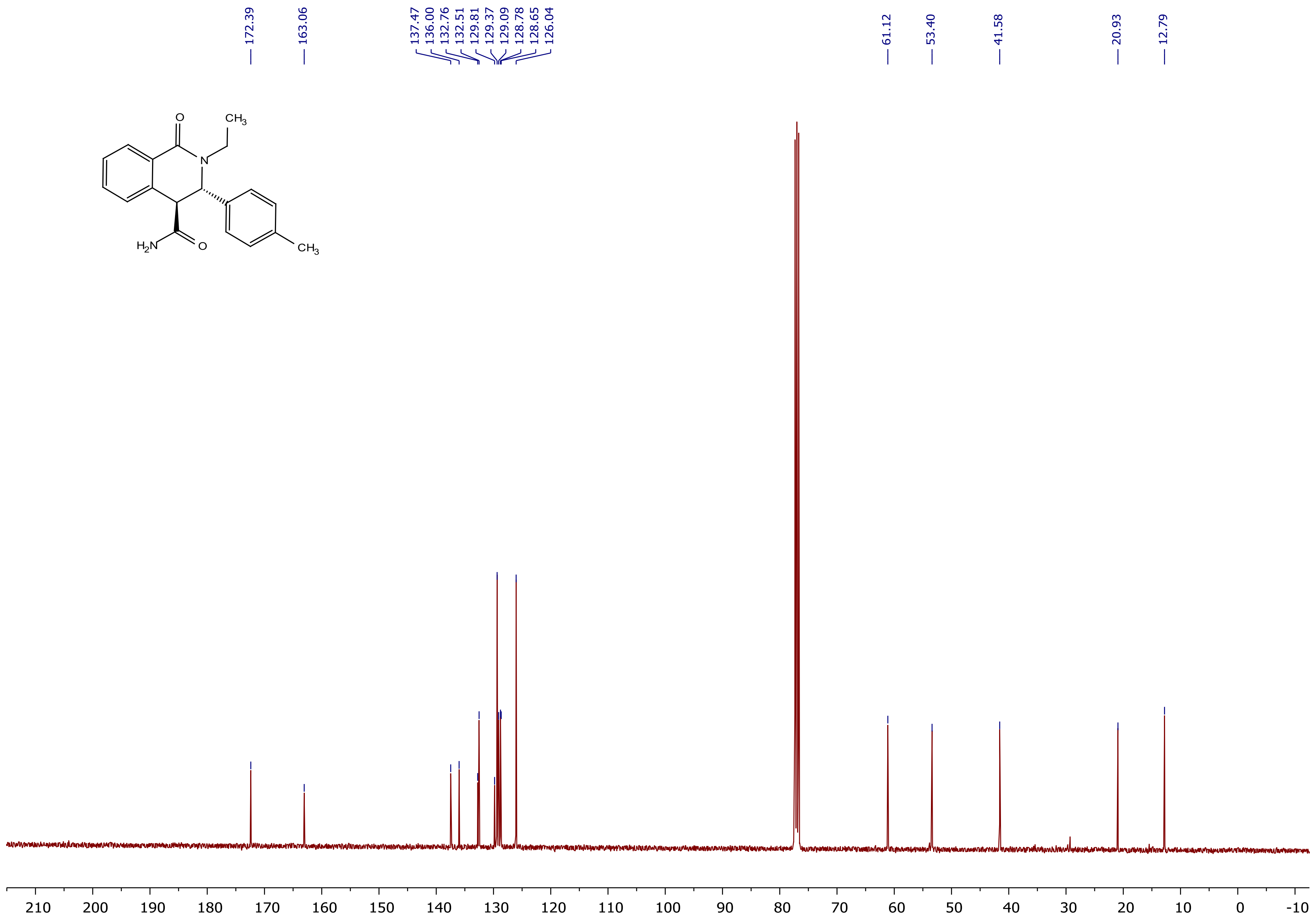

^{1}H NMR spectrum of compound 23b

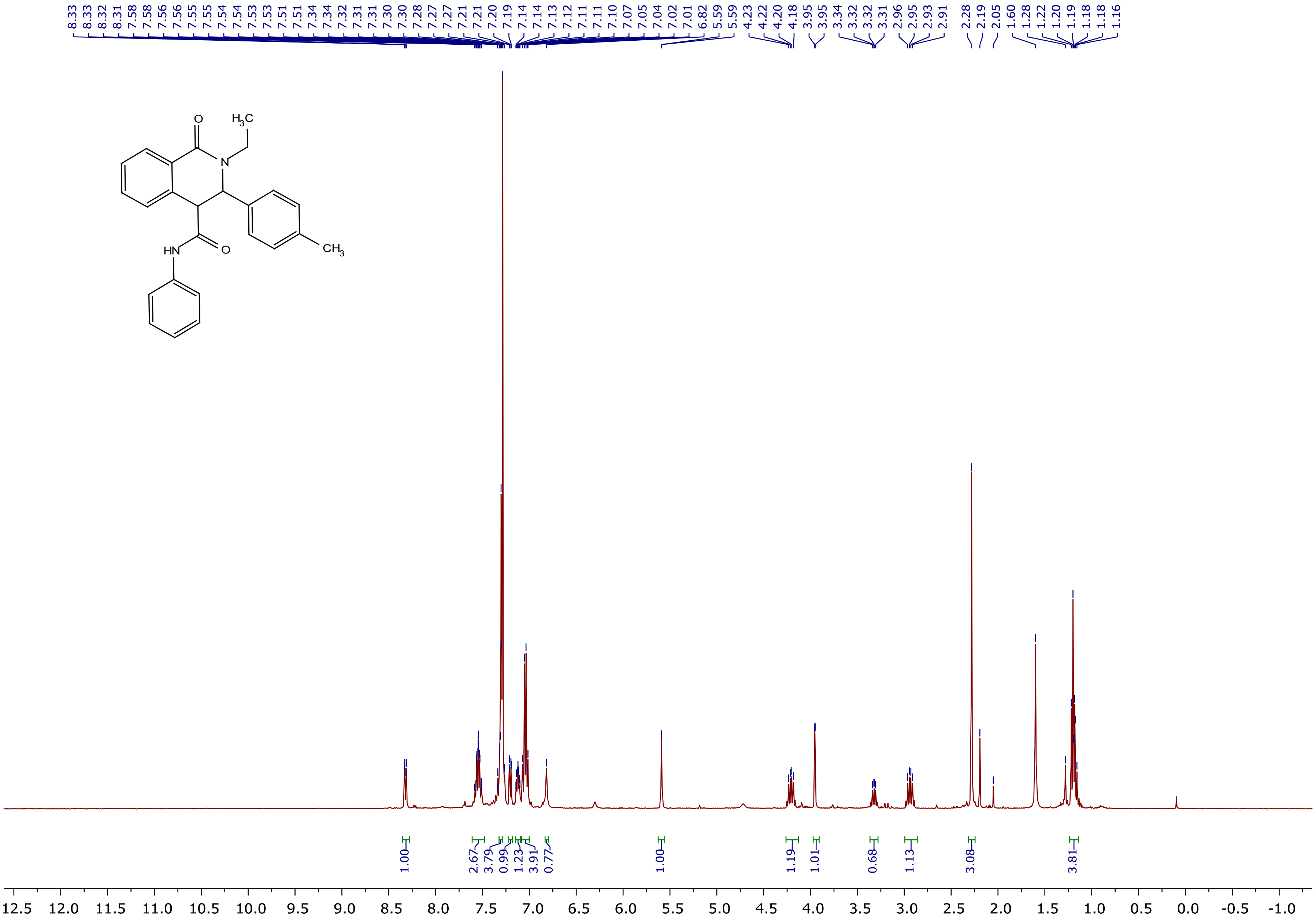

^{13}C NMR spectrum of compound 23b

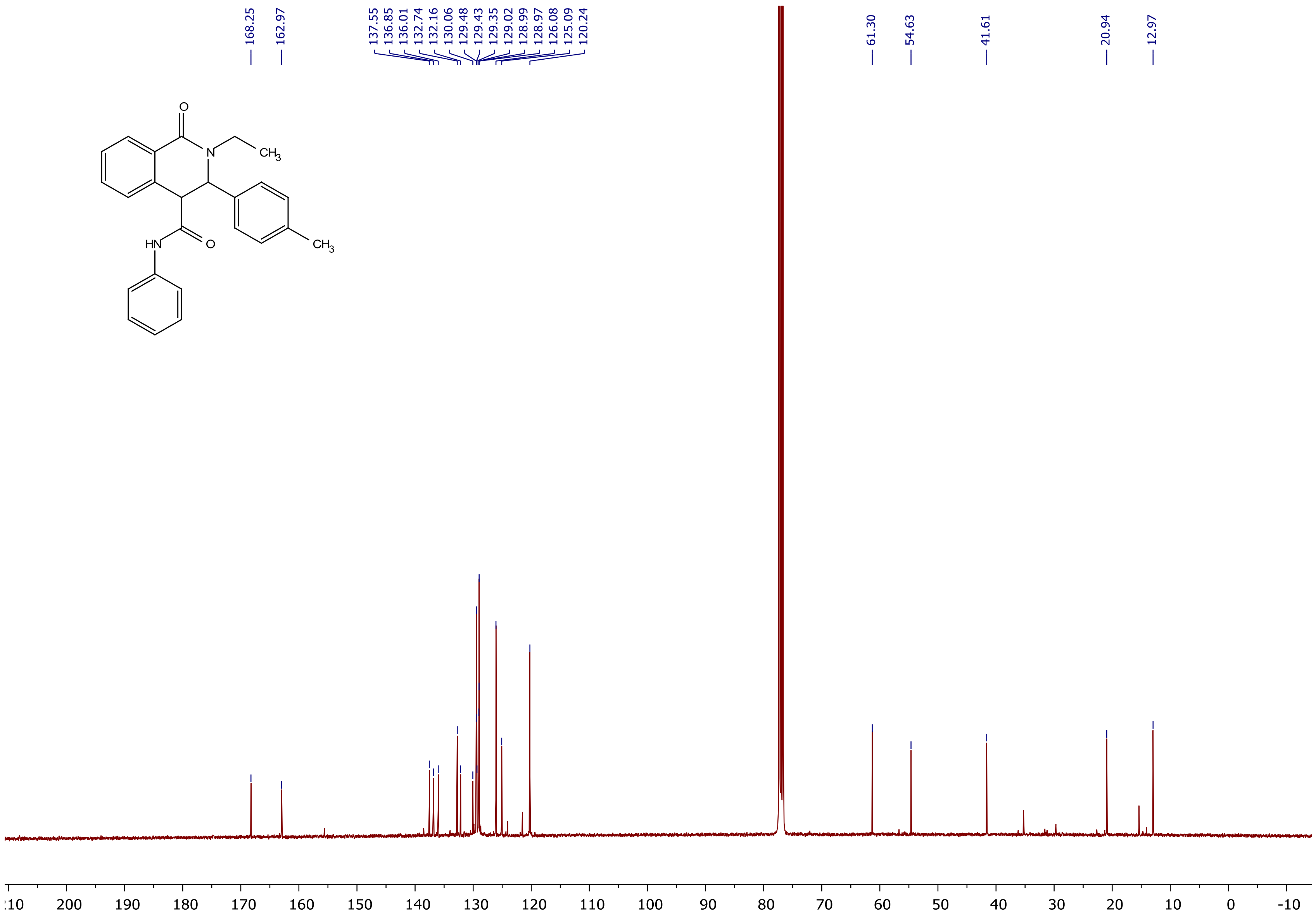

^{1}H NMR spectrum of compound 24a

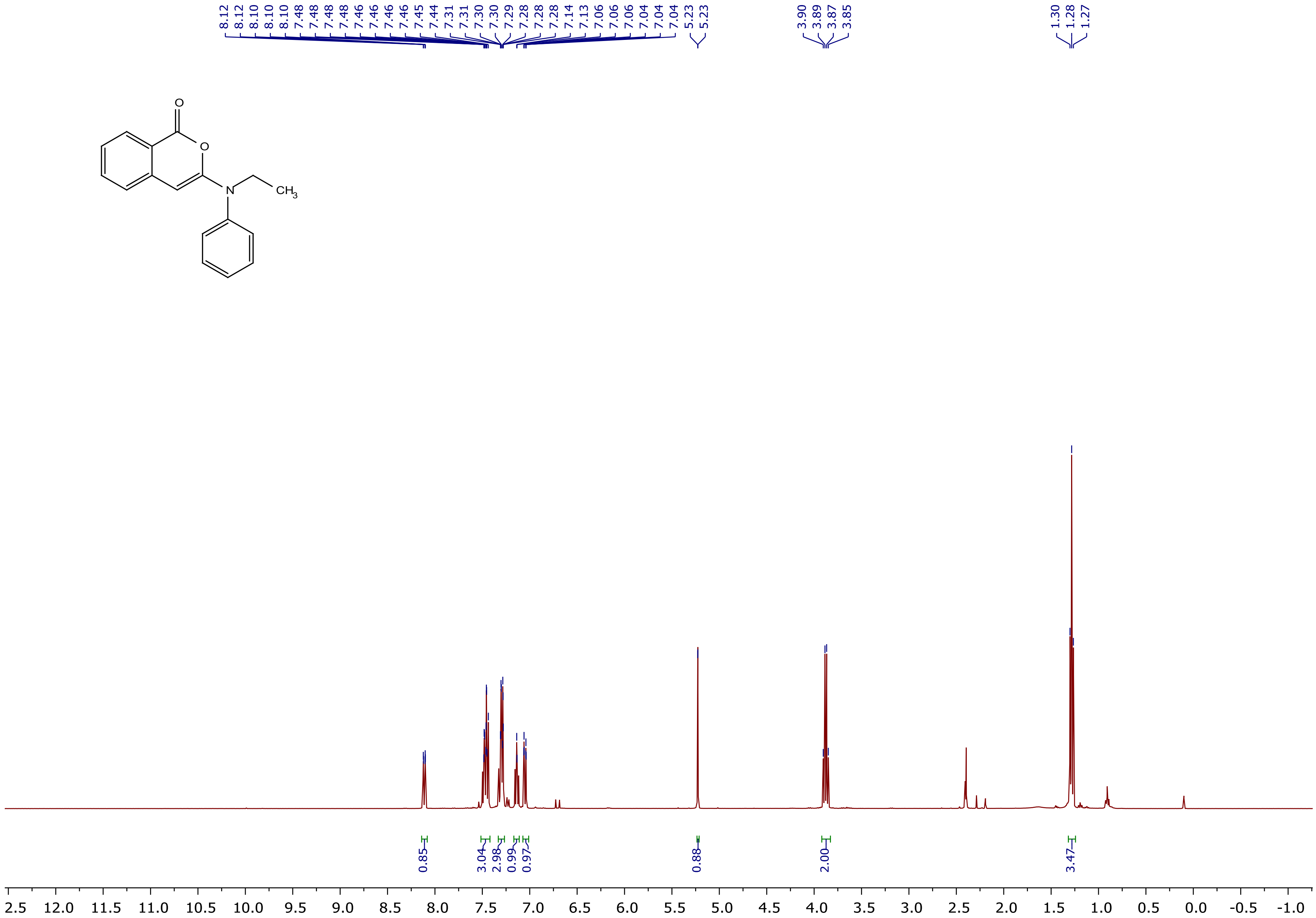

^{13}C NMR spectrum of compound 24a

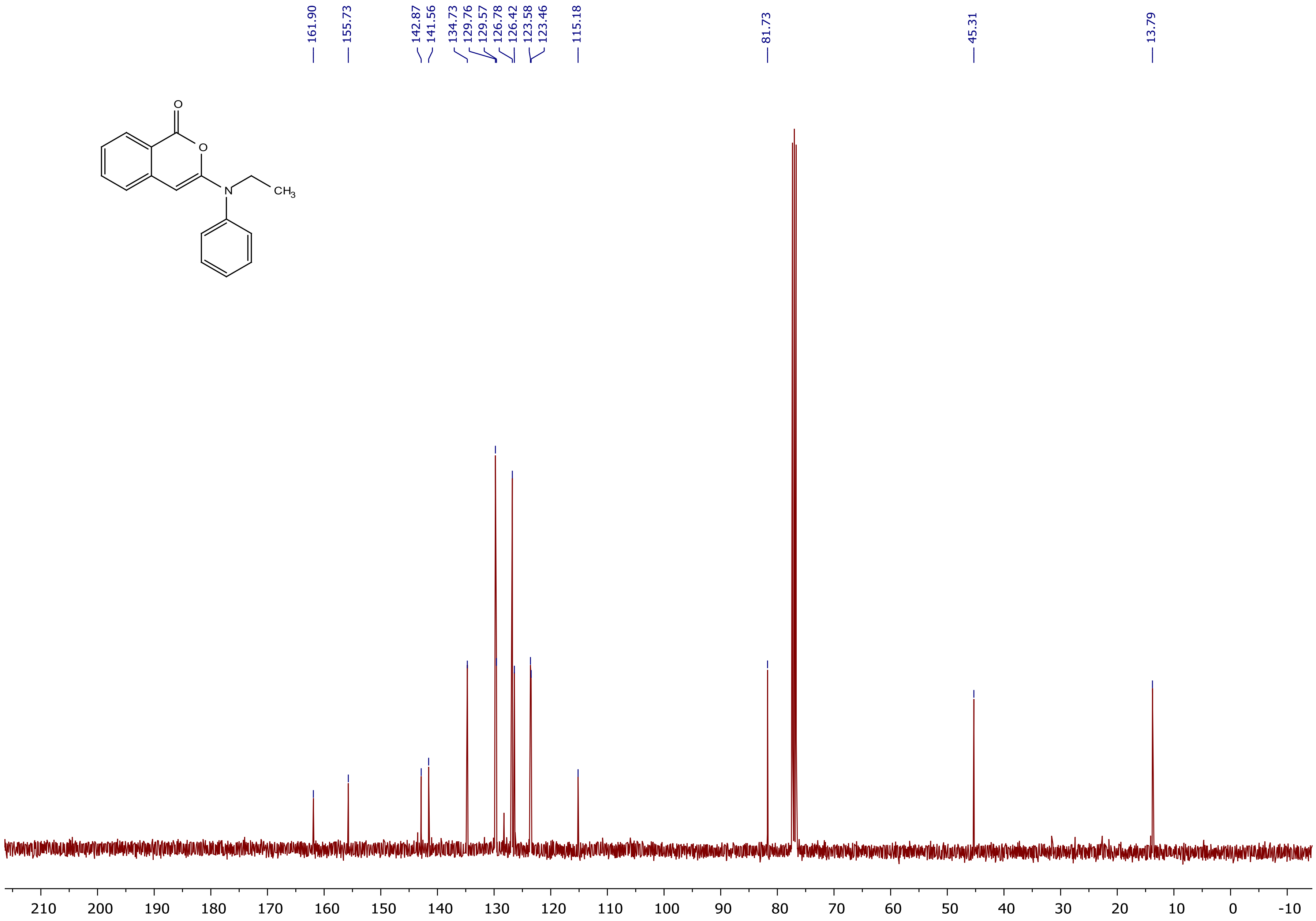

^{1}H NMR spectrum of compound 24b

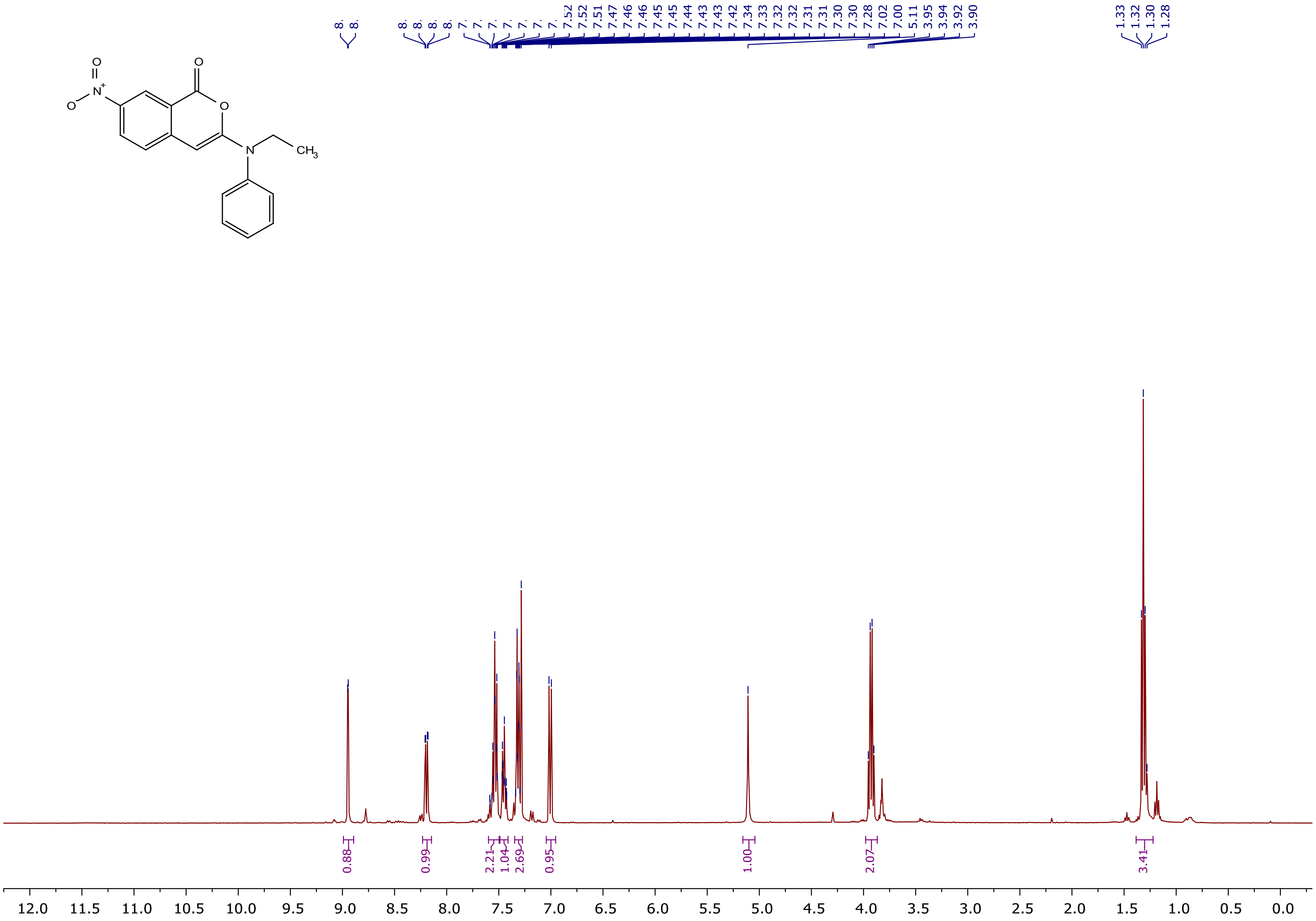

^{13}C NMR spectrum of compound 24b

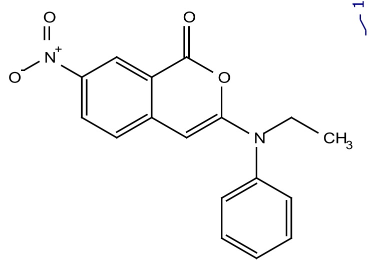

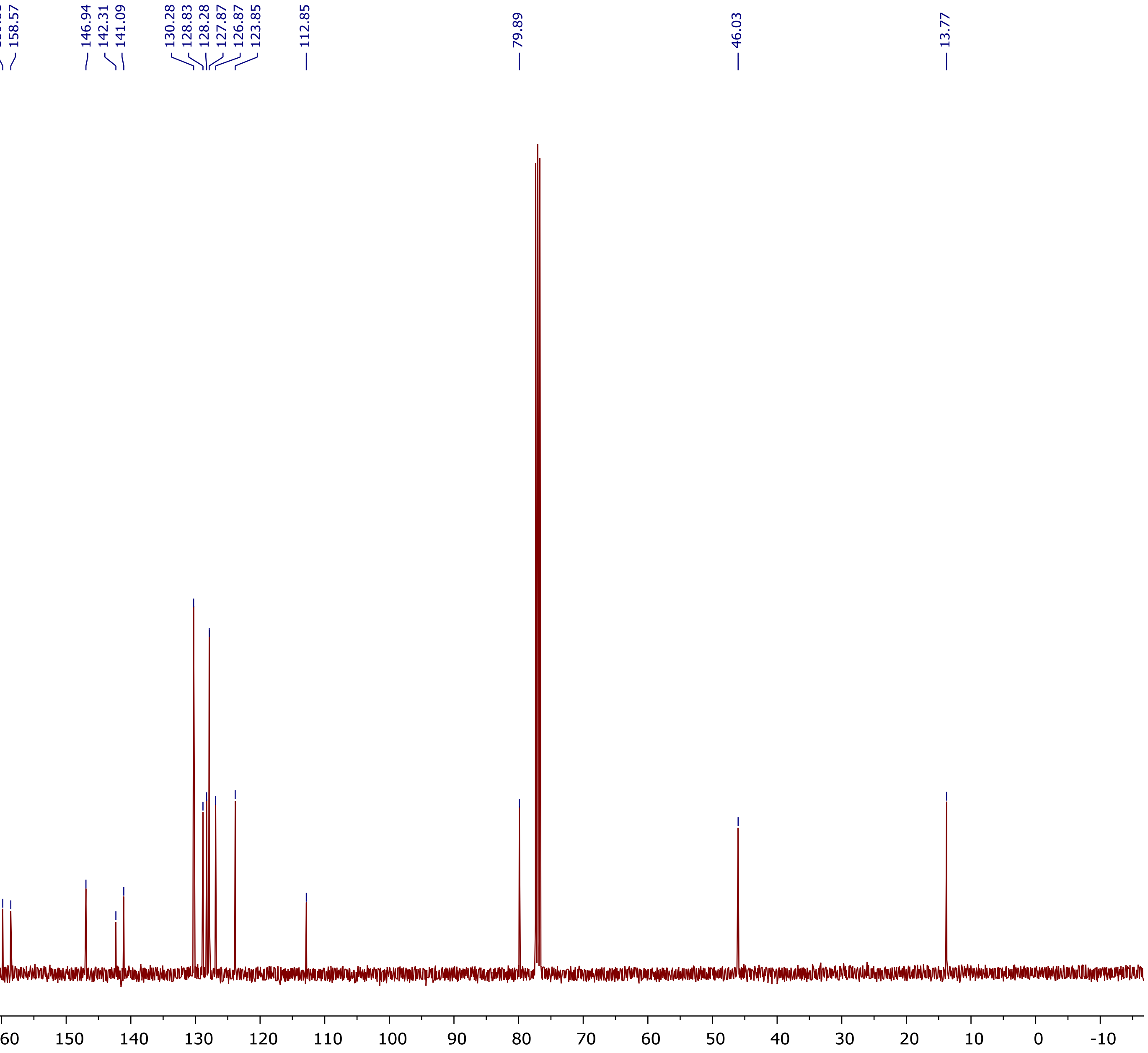

^{1}H NMR spectrum of compound 26

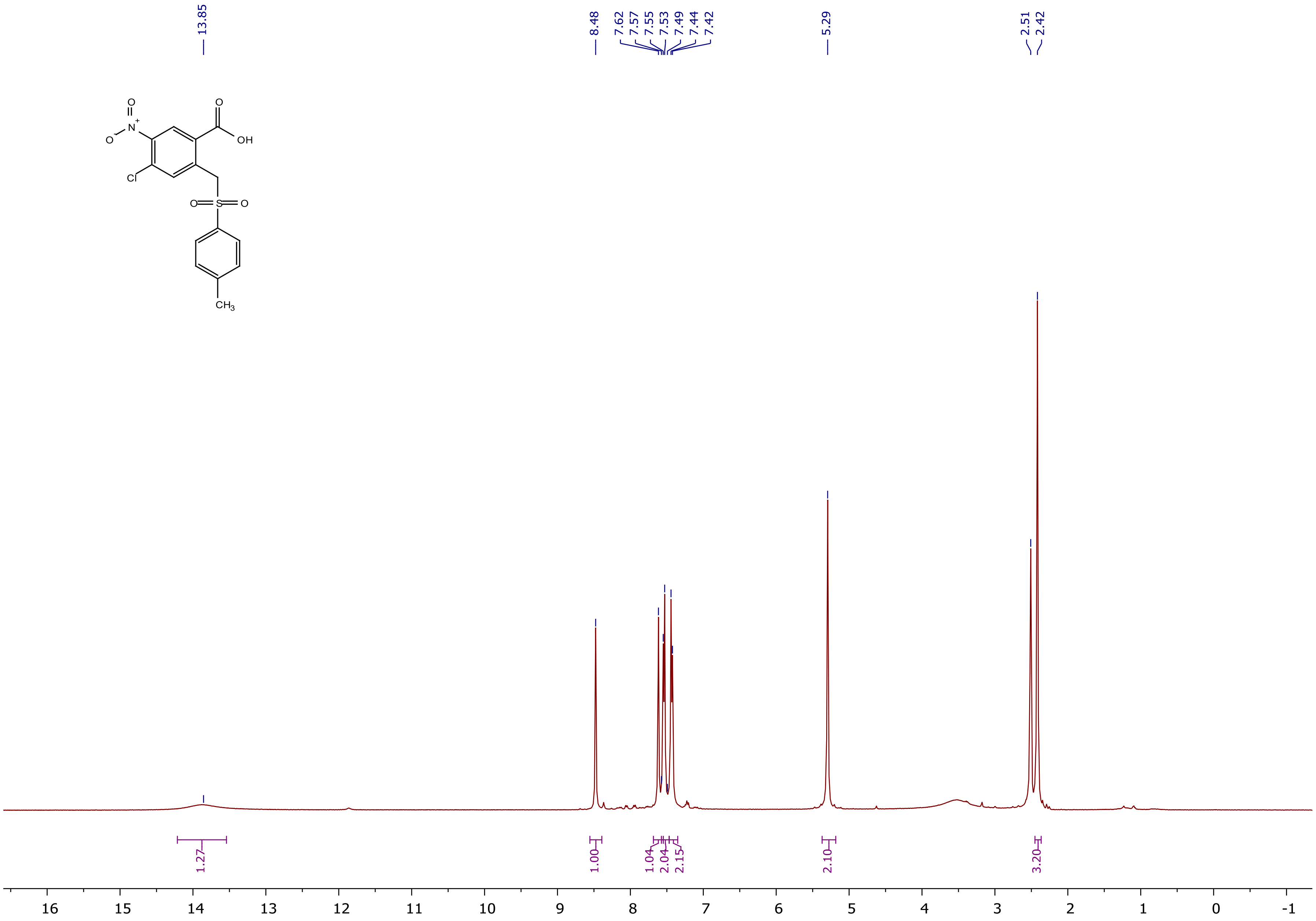

^{13}C NMR spectrum of compound 26

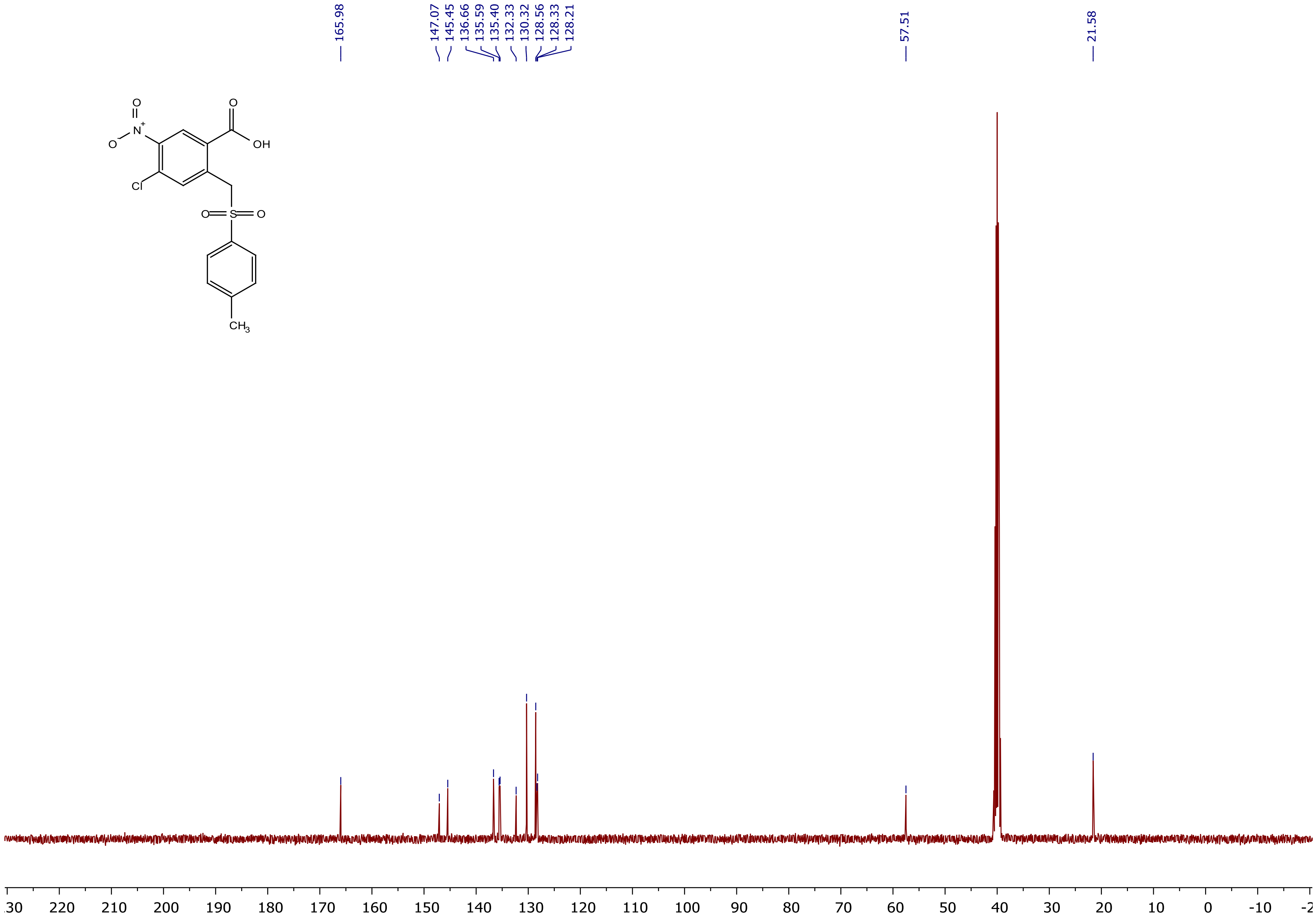

^{1}H NMR spectrum of compound 29

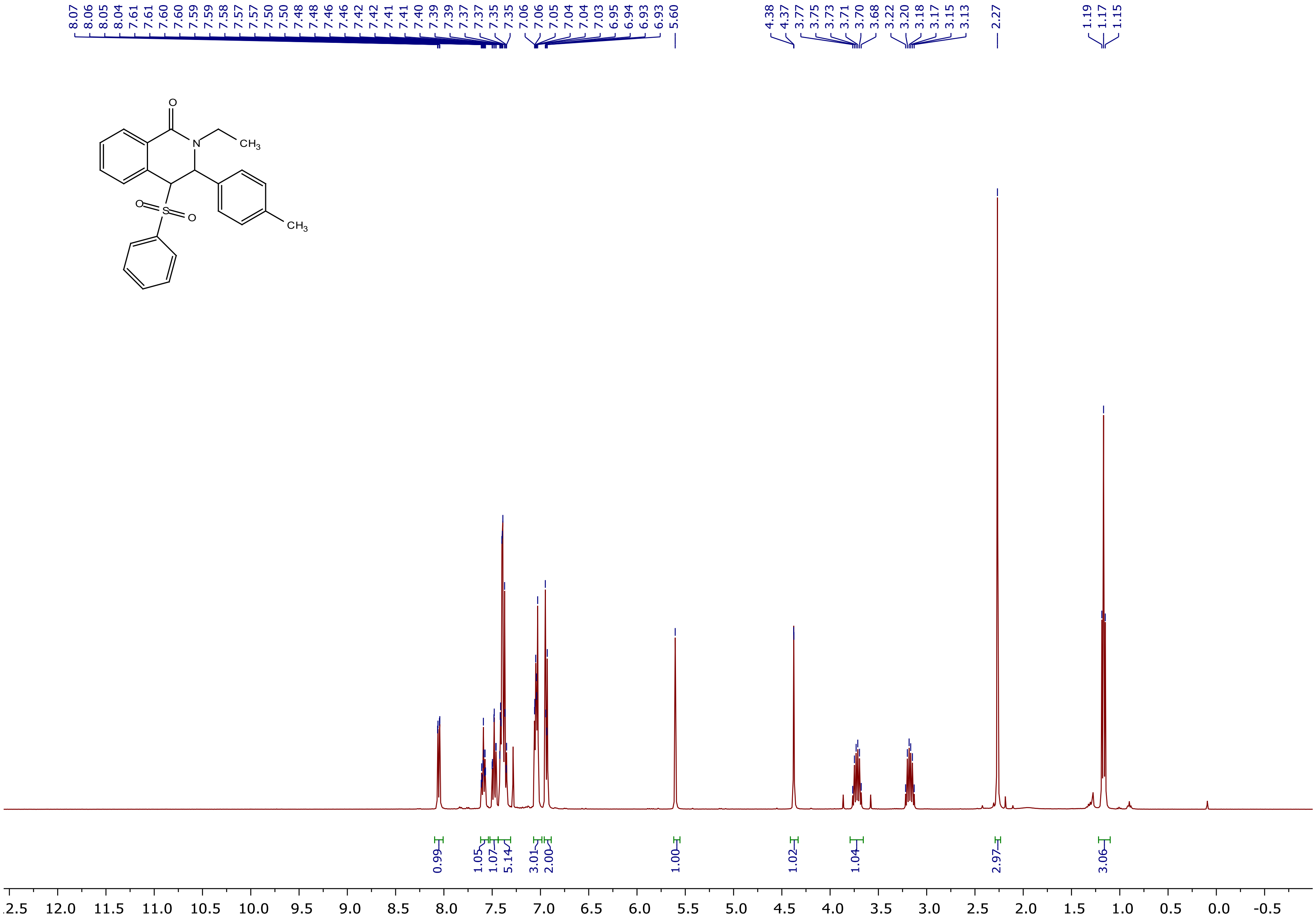

^{13}C NMR spectrum of compound 29

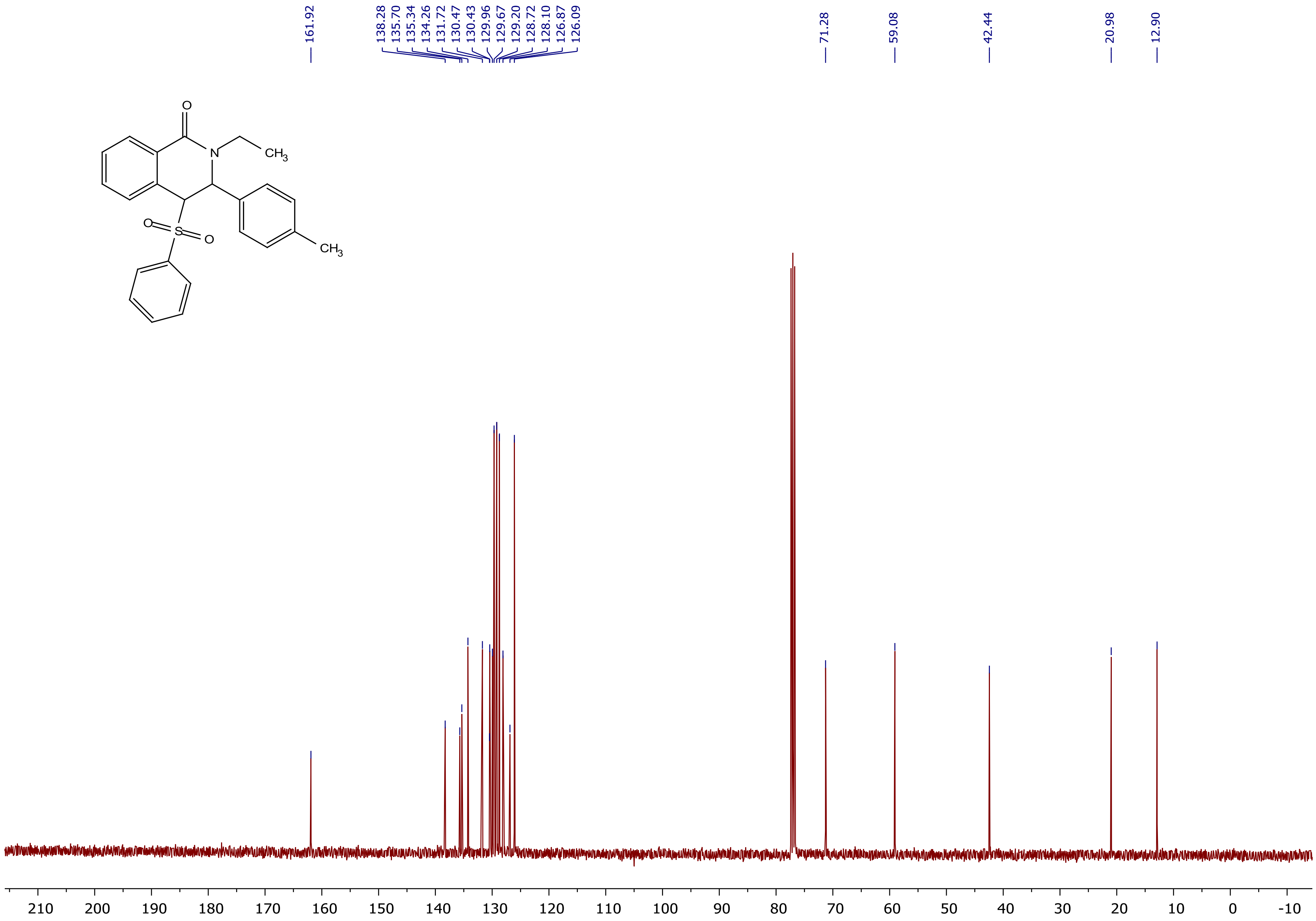

^{1}H NMR spectrum of compound 30

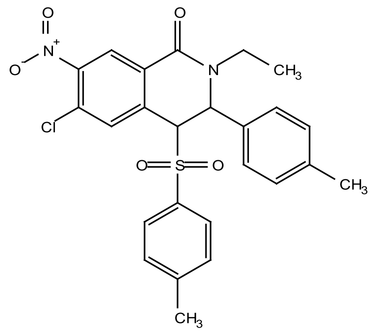

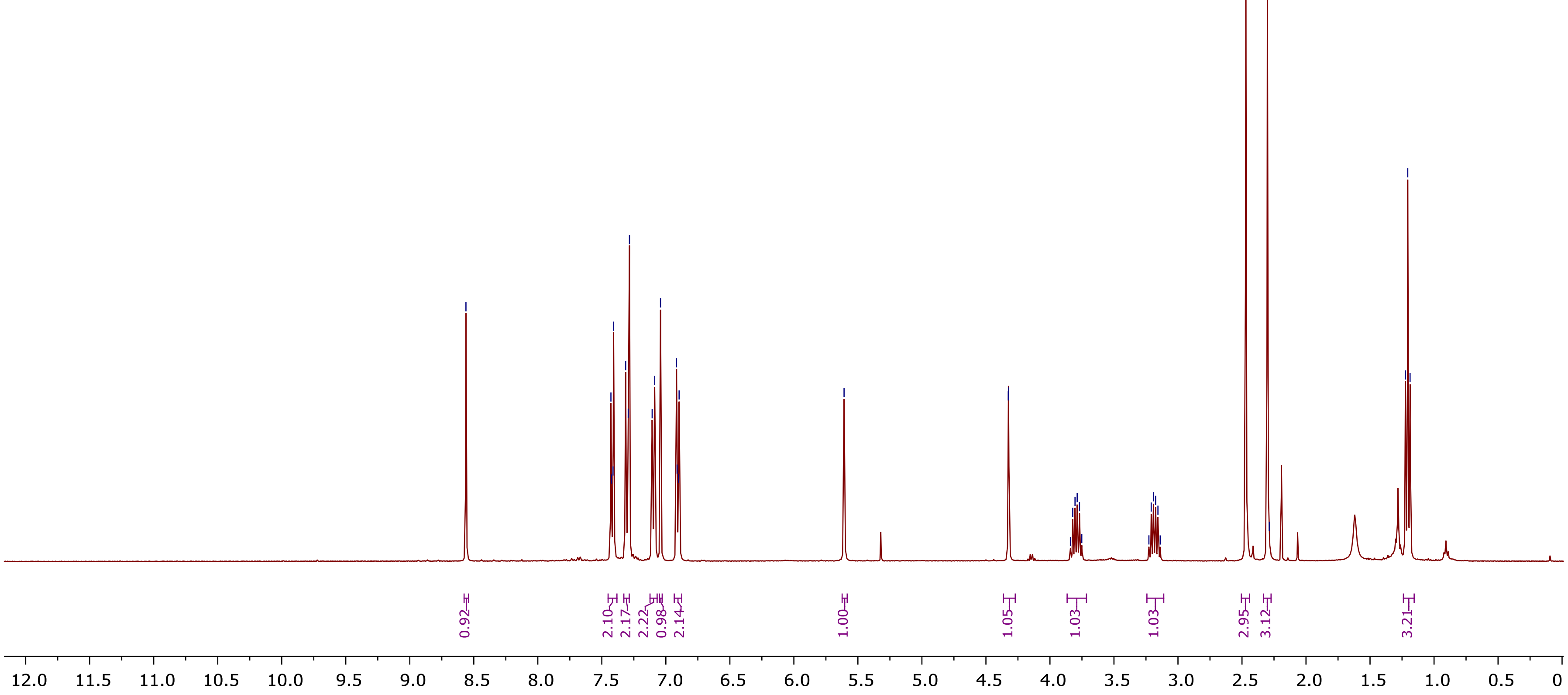

^{13}C NMR spectrum of compound 30

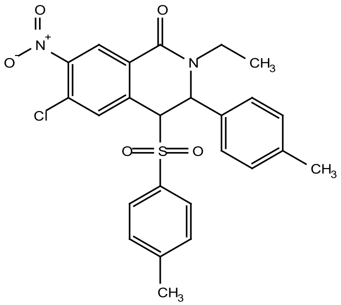

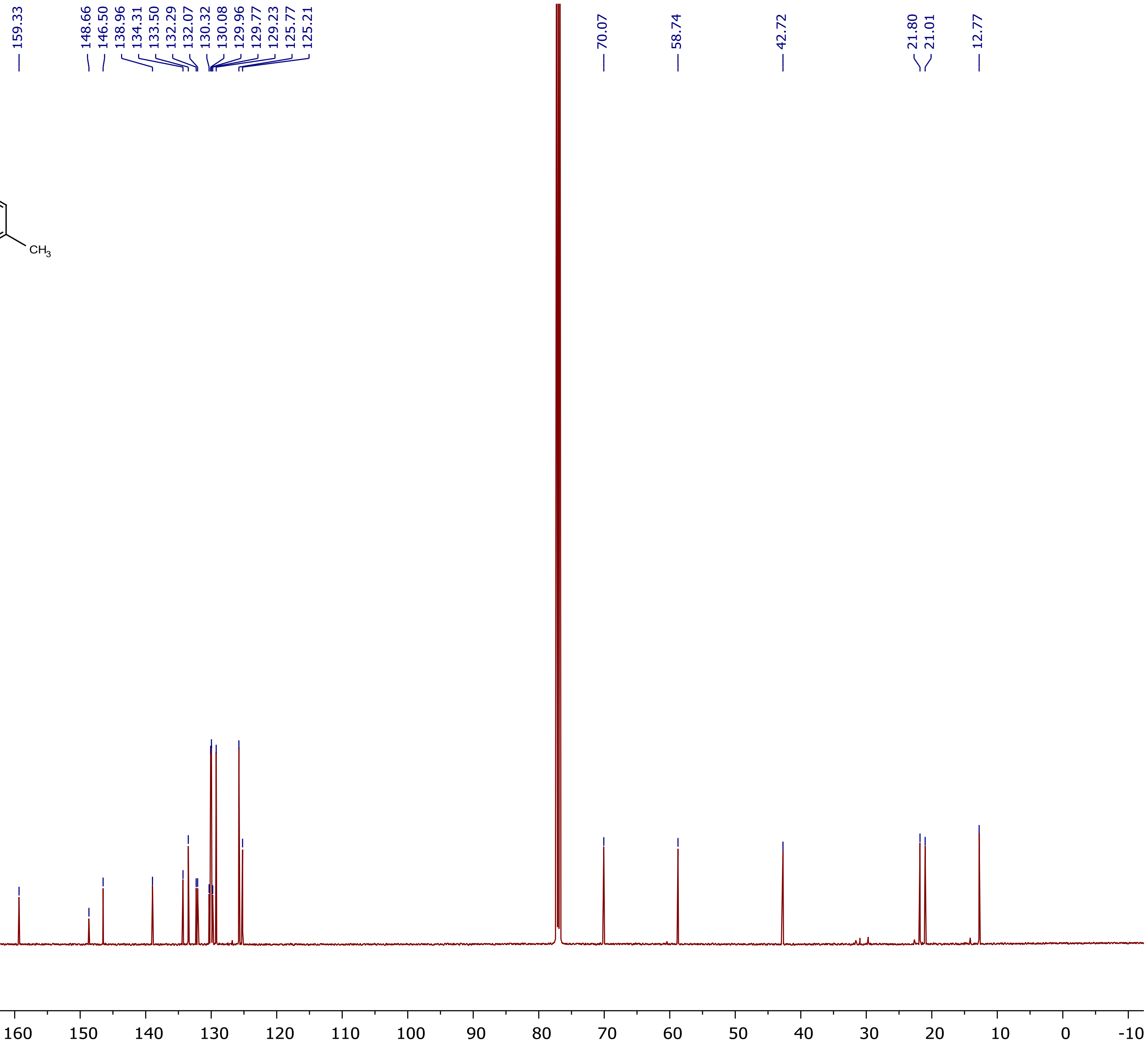

Supplement: Supplementary file 1 [file molecules-27-07211-s001.zip › molecules-1958585-supplementary.pdf]
